# Supplementary material for: Catalytic enantioselective reductive domino alkyl arylation of acrylates via nickel/photoredox catalysis
Source: Nat Commun. 2021 Nov 16;12:6613. doi: 10.1038/s41467-021-26794-8 (PMC8595378; doi:10.1038/s41467-021-26794-8)
Supplement: Supplementary file 1 — Supplementary Information [file 41467_2021_26794_MOESM1_ESM.pdf]

Catalytic Enantioselective Reductive Domino Alkylarylation of Acrylates via Nickel/Photoredox Catalysis

Qian *et al.*

NMR Spectra

(*R*)-*tert*-Butyl 2-(4-acetylphenyl)-4,4-dimethylpentanoate (4a)

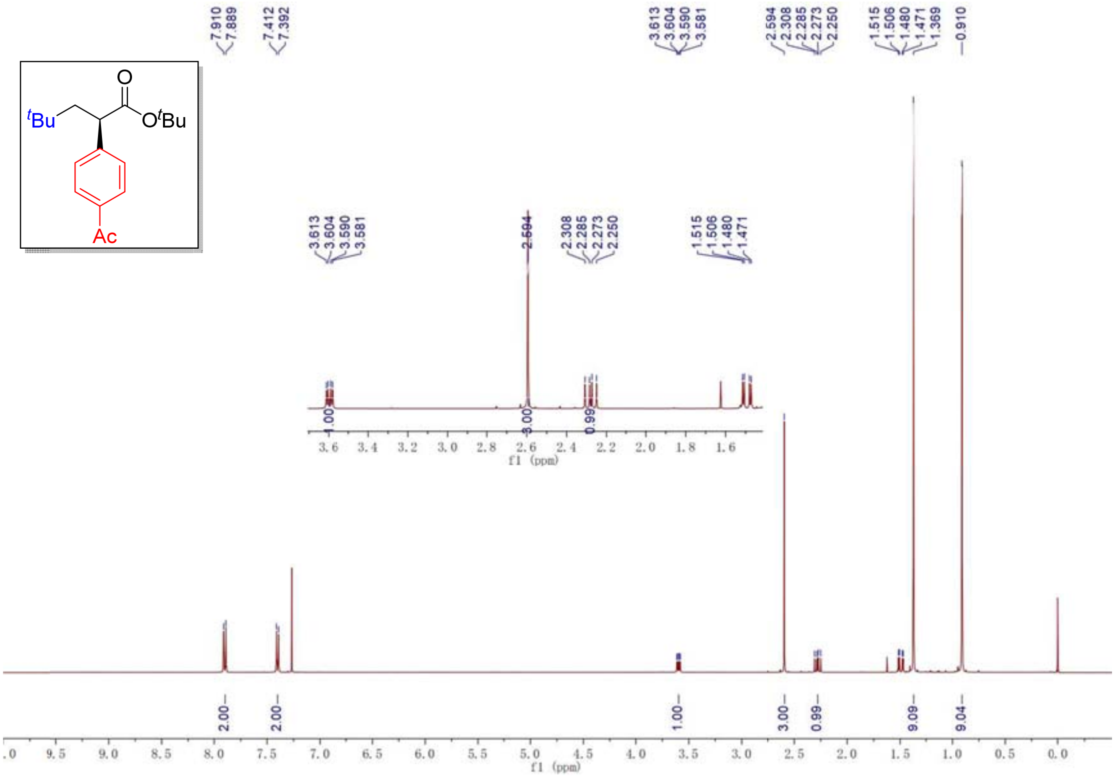

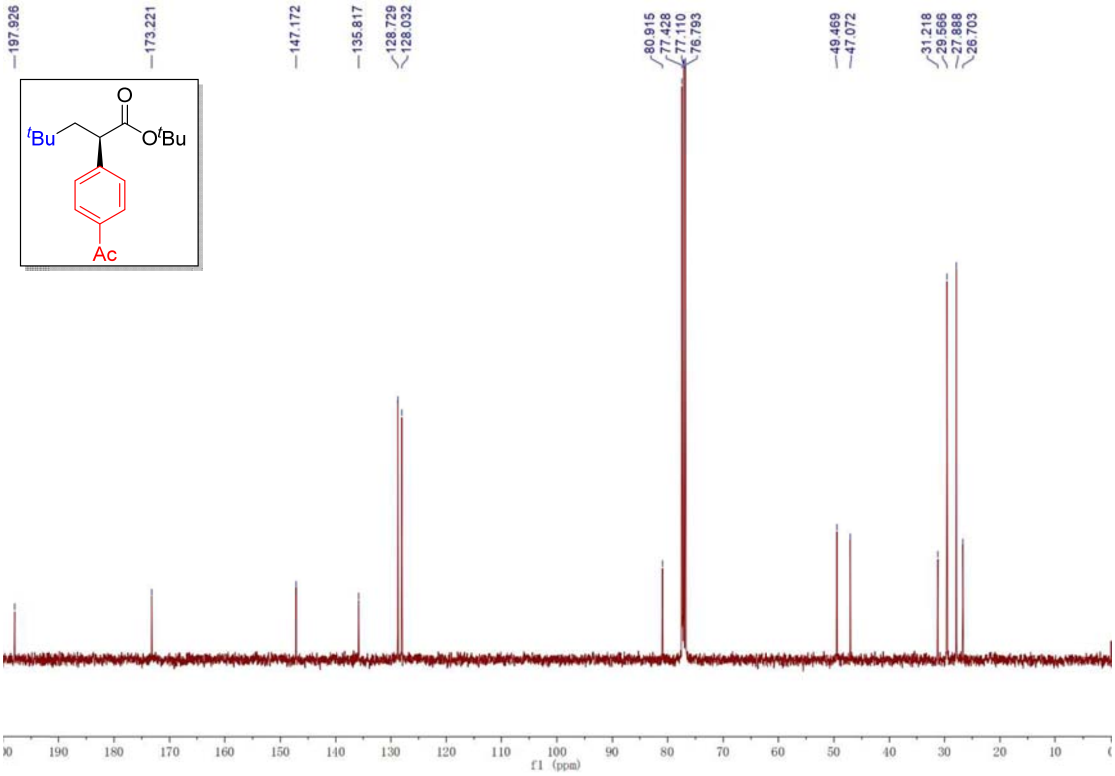

Supplementary Figure 1. ^{1}**H (400 MHz) and** ^{13}**C {**^{1}**H} (101 MHz) NMR spectra of 4a in** $CDCl_3$

(*R*)-methyl 4-(1-(*tert*-butoxy)-4,4-dimethyl-1-oxopentan-2-yl)benzoate (4b)

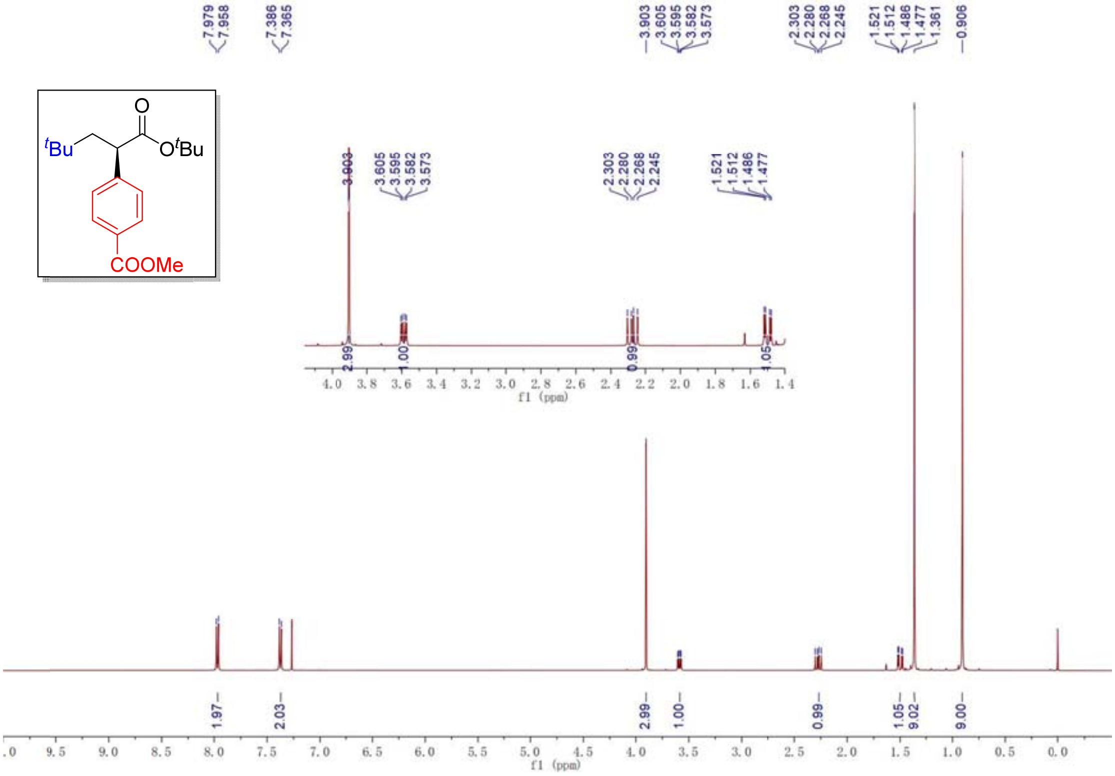

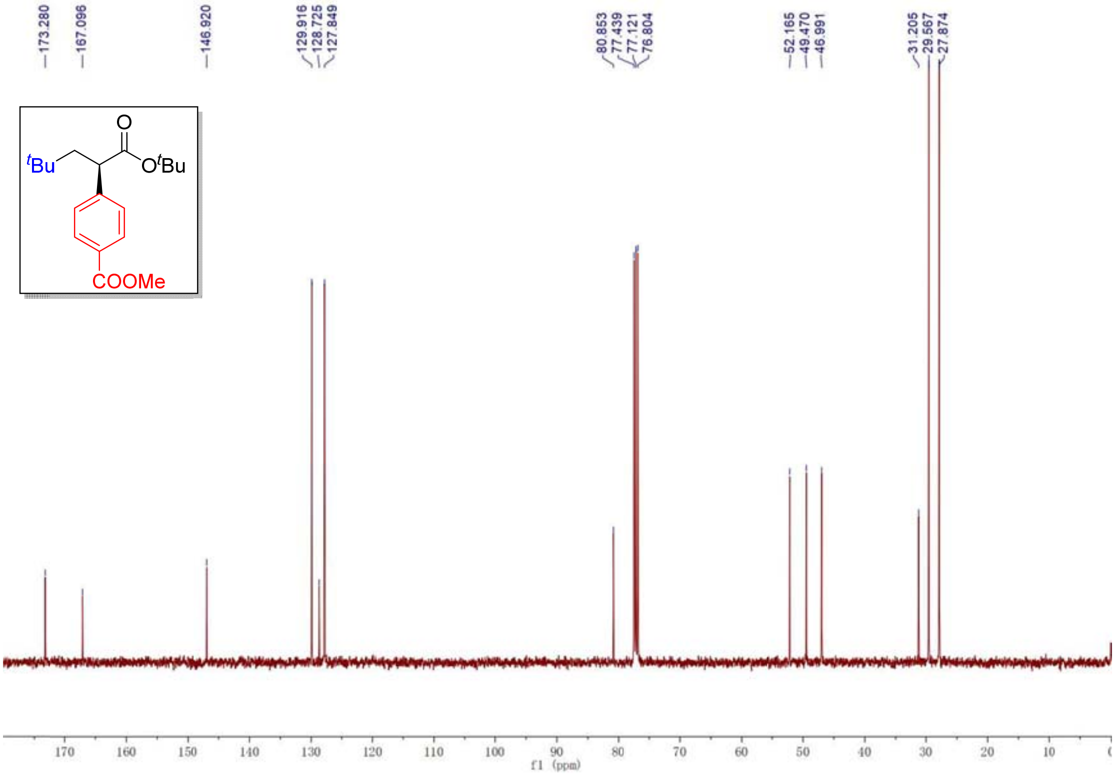

Supplementary Figure 2. **^{1}H (400 MHz) and ^{13}C {^{1}H} (101 MHz) NMR spectra of 4b in $CDCl_3$**

(*R*)-*tert*-butyl 2-(4-cyanophenyl)-4,4-dimethylpentanoate (4c)

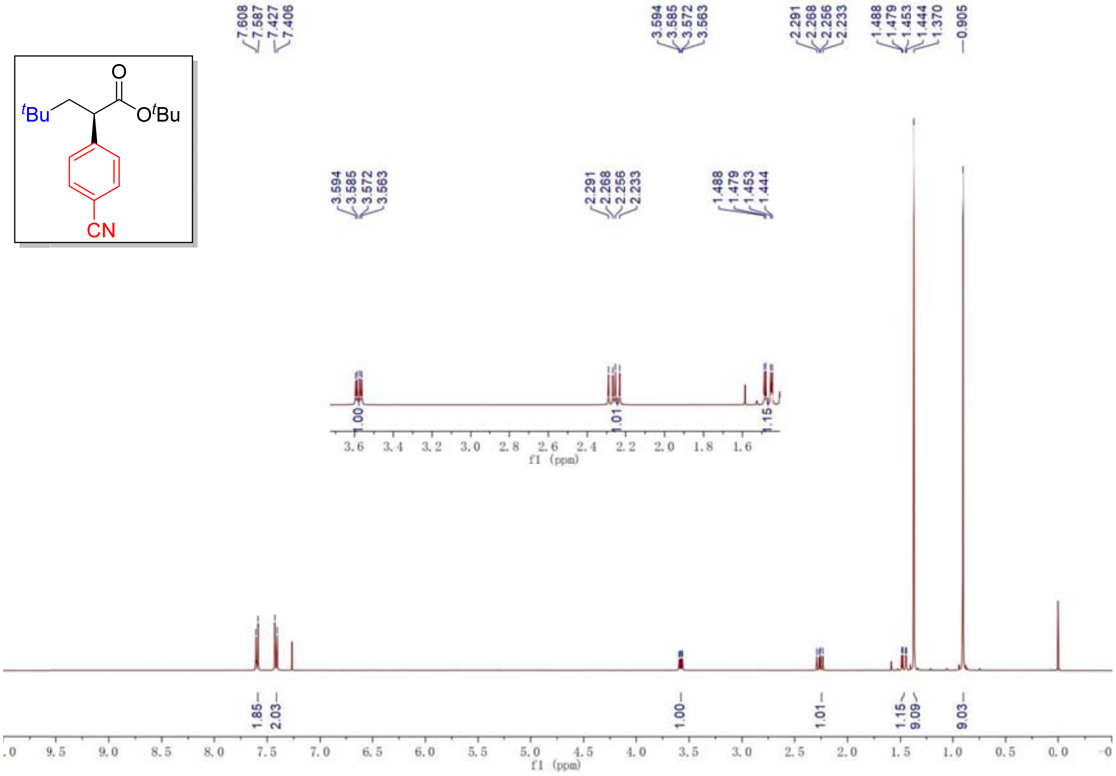

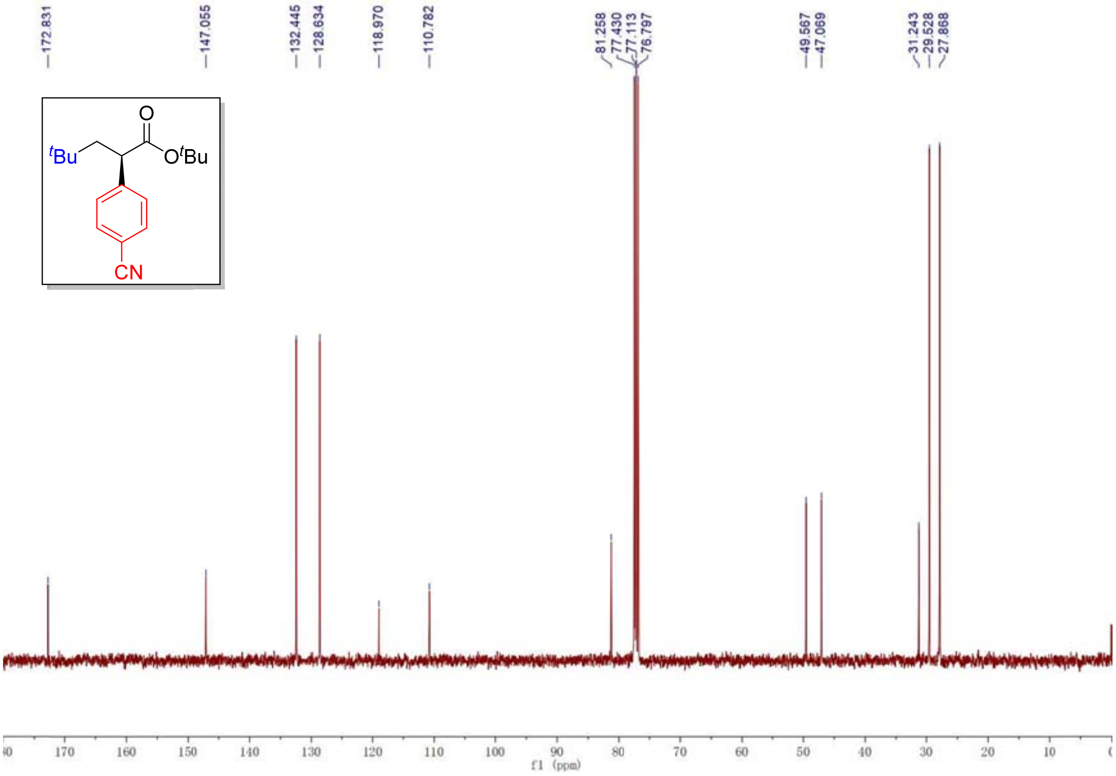

Supplementary Figure 3. ^{1}**H (400 MHz) and** ^{13}**C {**^{1}**H} (101 MHz) NMR spectra of 4c in** $CDCl_3$

(*R*)-*tert*-butyl 2-(4-formylphenyl)-4,4-dimethylpentanoate (4d)

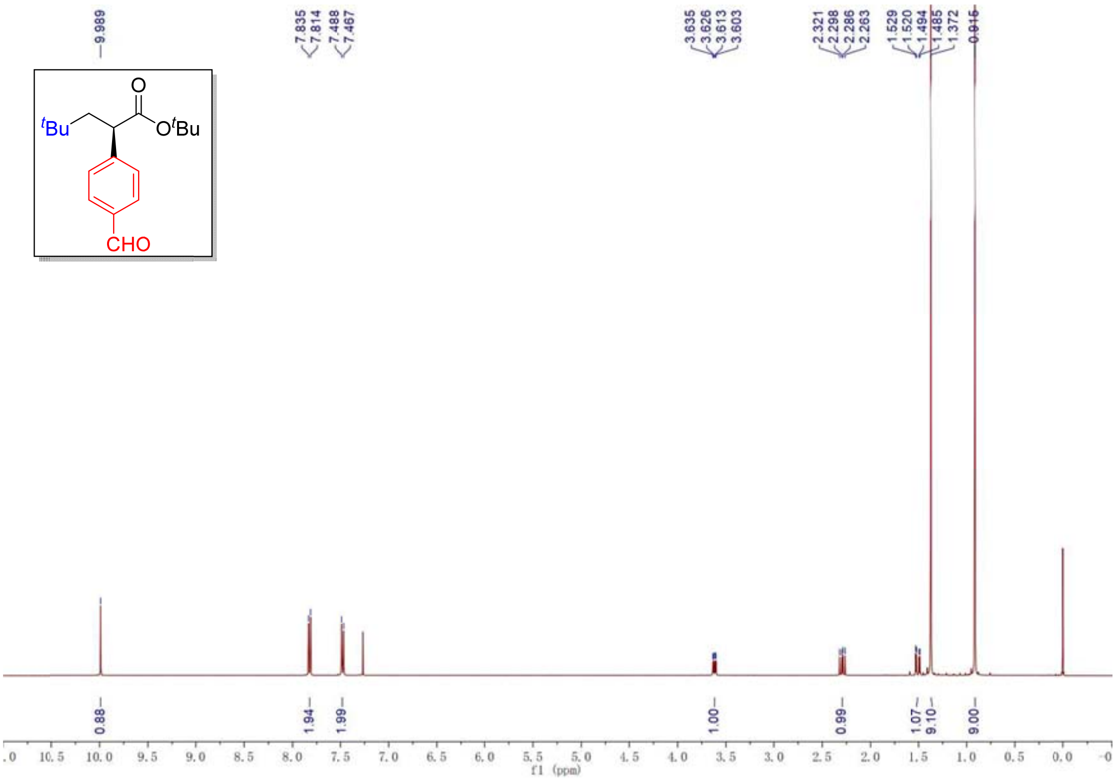

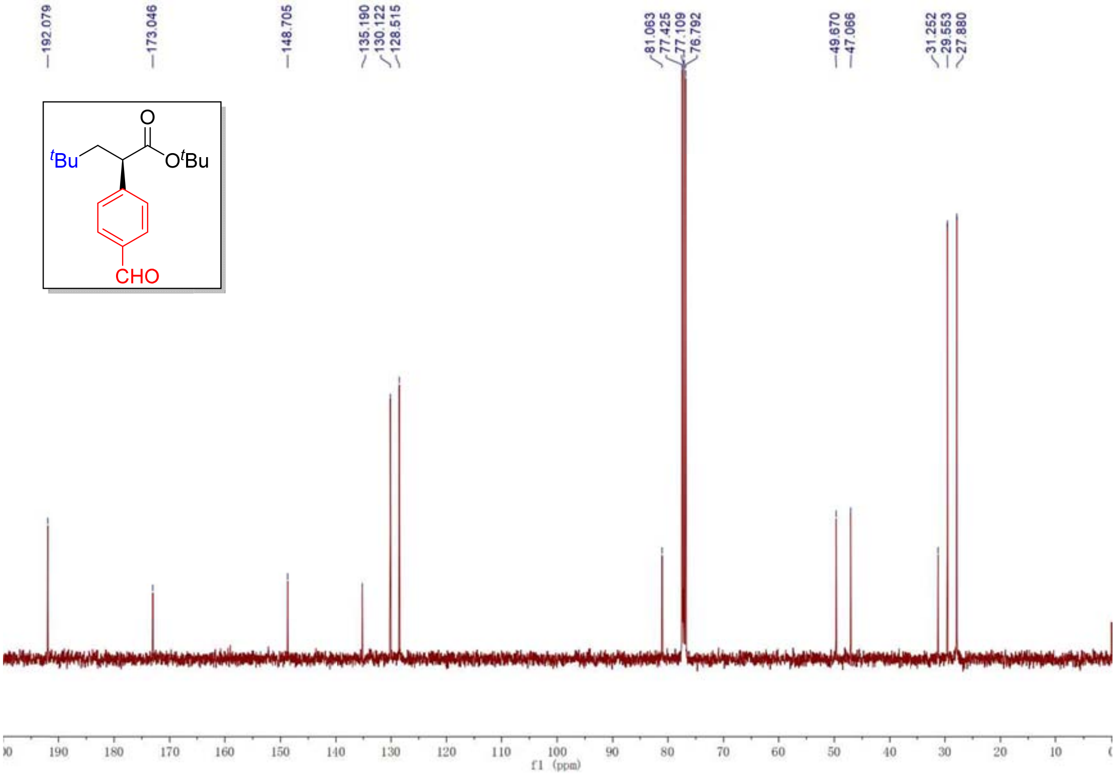

Supplementary Figure 4. ^{1}H (400 MHz) and ^{13}C {^{1}H} (101 MHz) NMR spectra of 4d in $CDCl_3$

(*R*)-*tert*-Buty 4,4-dimethyl-2-(4-(trifluoromethyl)phenyl)pentanoate (4e)

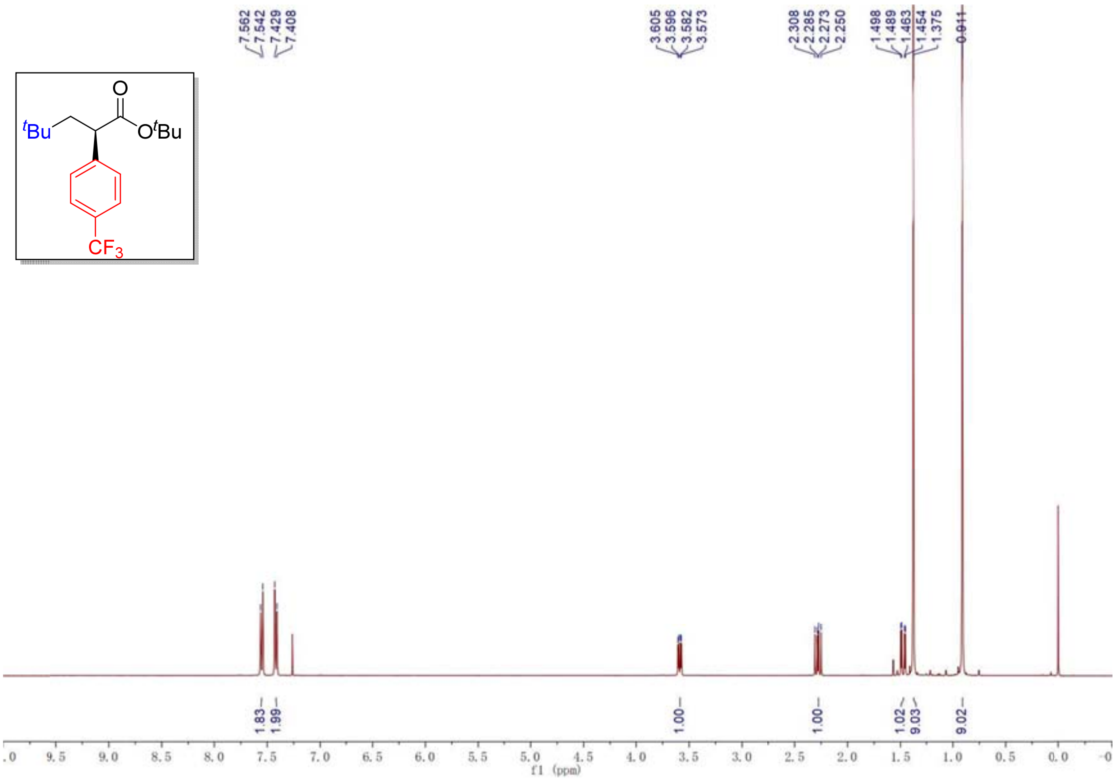

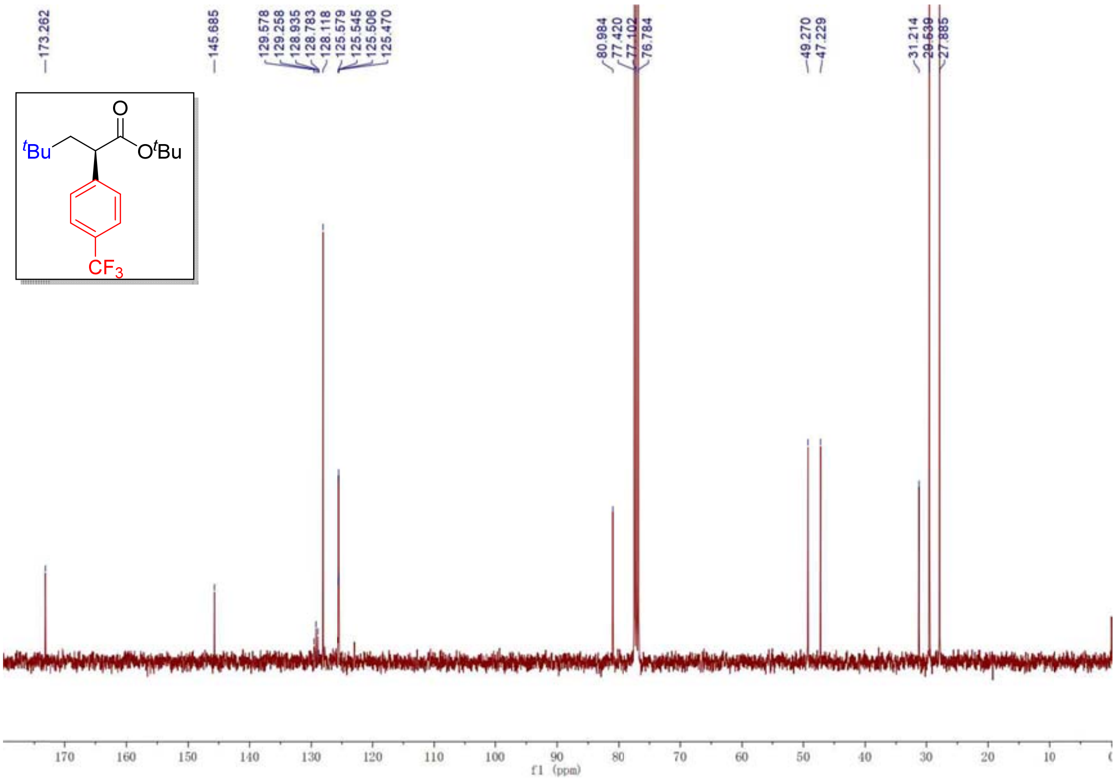

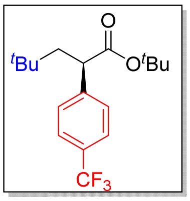

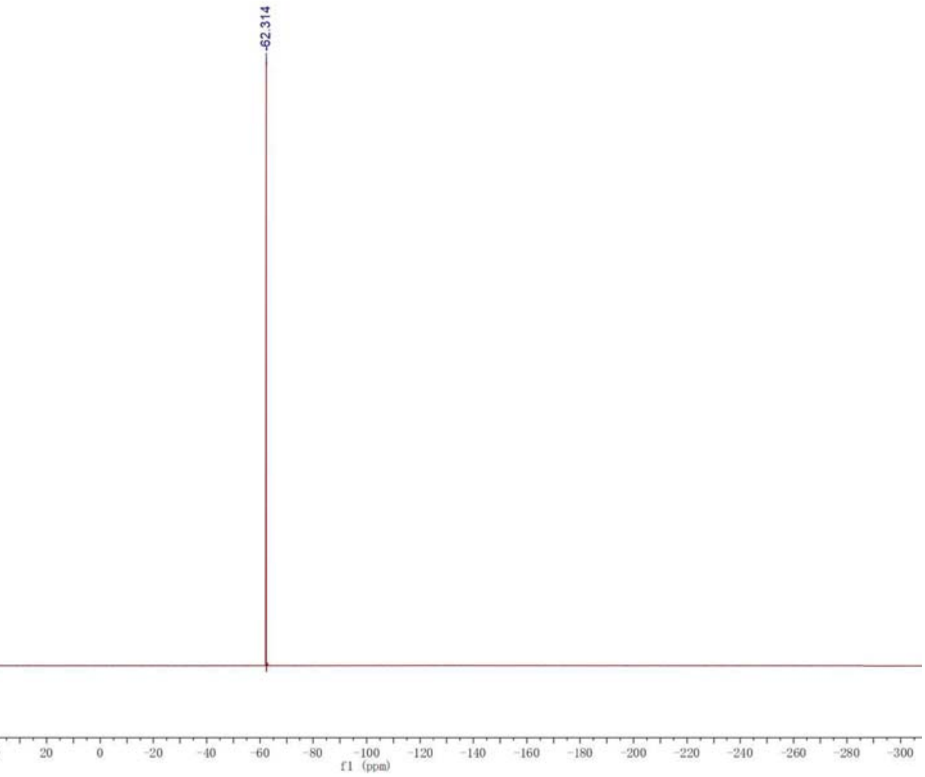

Supplementary Figure 5. ^{1}**H (400 MHz),** ^{13}**C {**^{1}**H} (101 MHz) and** ^{19}**F NMR spectra of 4e in** $CDCl_3$

(*R*)-*tert*-butyl 4,4-dimethyl-2-(4-(trifluoromethoxy)phenyl)pentanoate (4f)

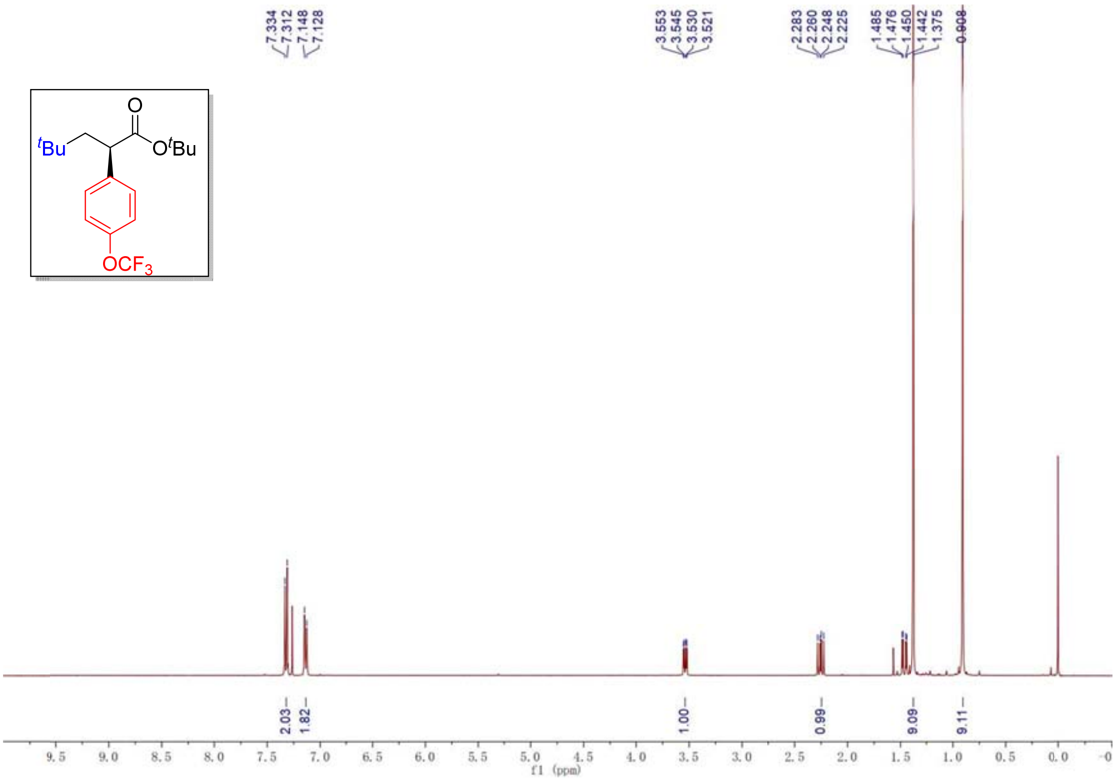

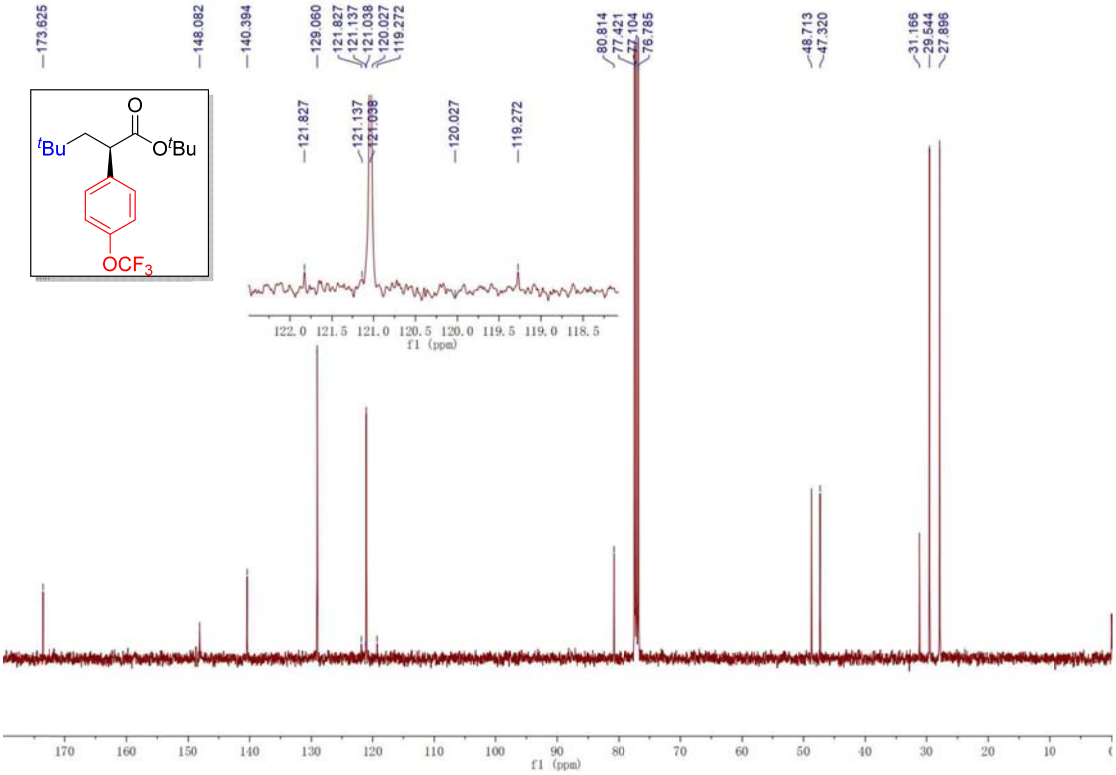

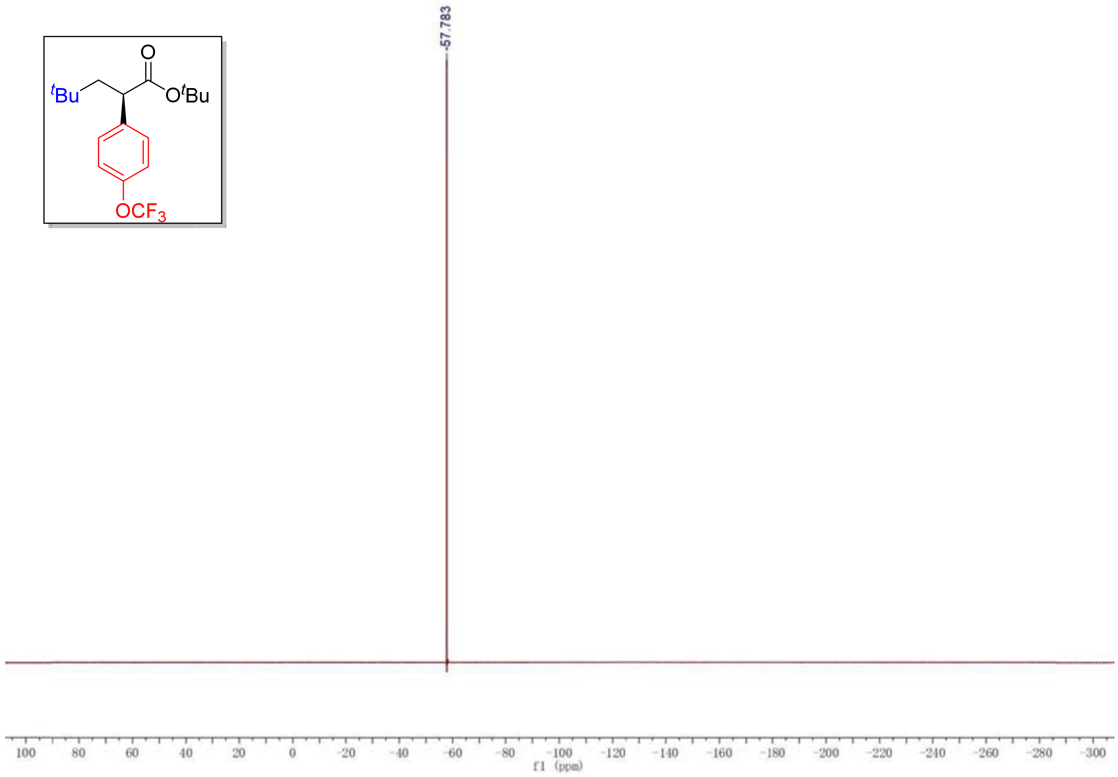

Supplementary Figure 6. ^{1}**H (400 MHz),** ^{13}**C {**^{1}**H} (101 MHz) and** ^{19}**F NMR spectra of 4f in** $CDCl_3$

(*R*)-*tert*-butyl 2-(4-fluorophenyl)-4,4-dimethylpentanoate (4g)

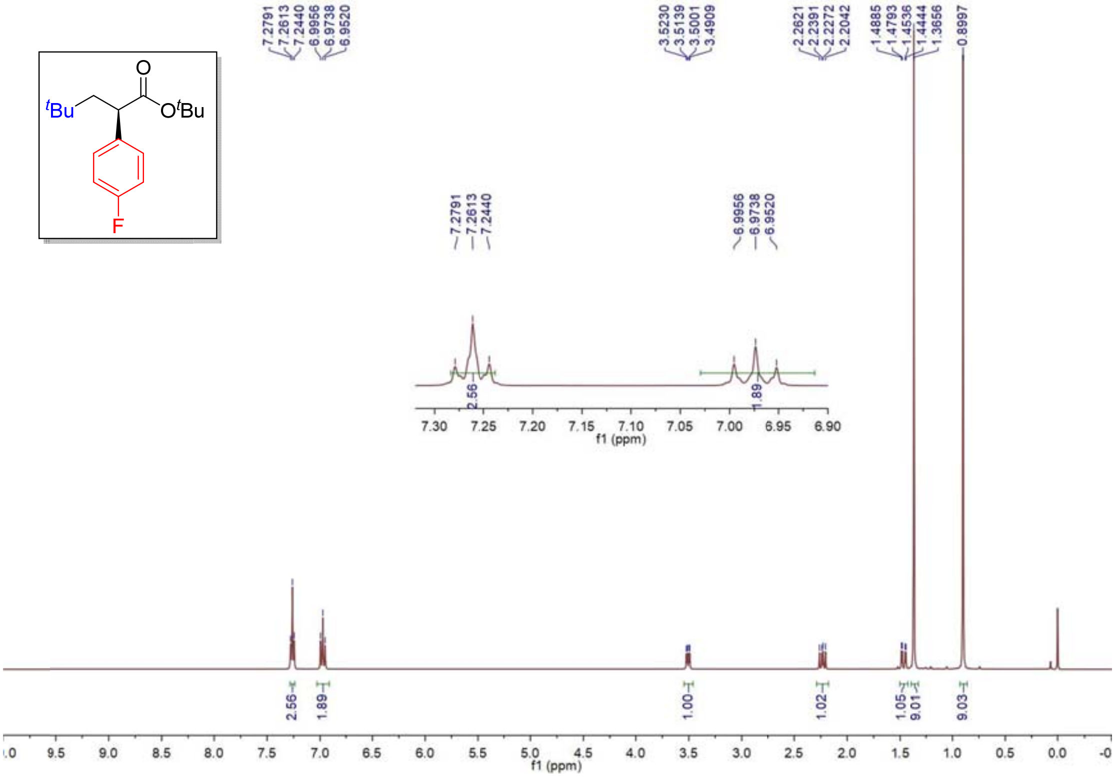

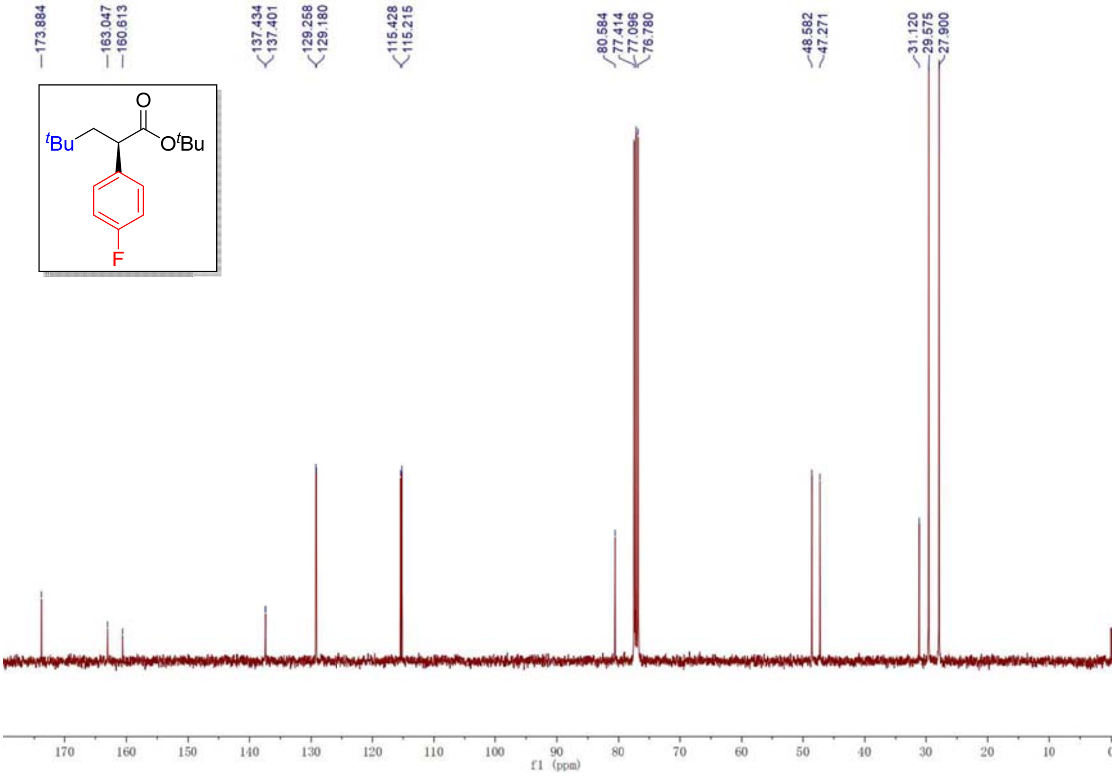

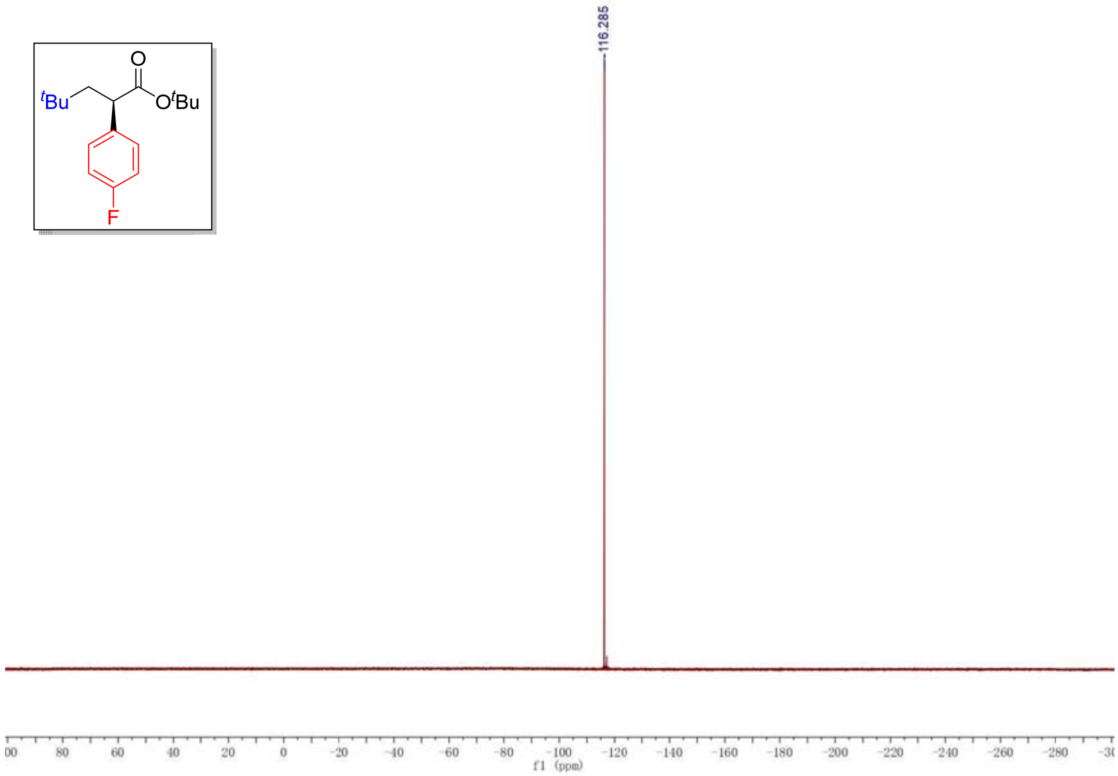

Supplementary Figure 7. ^{1}**H (400 MHz),** ^{13}**C {**^{1}**H} (101 MHz) and** ^{19}**F NMR spectra of 4g in** $CDCl_3$

(*R*)-*tert*-butyl 2-(4-chlorophenyl)-4,4-dimethylpentanoate (4h)

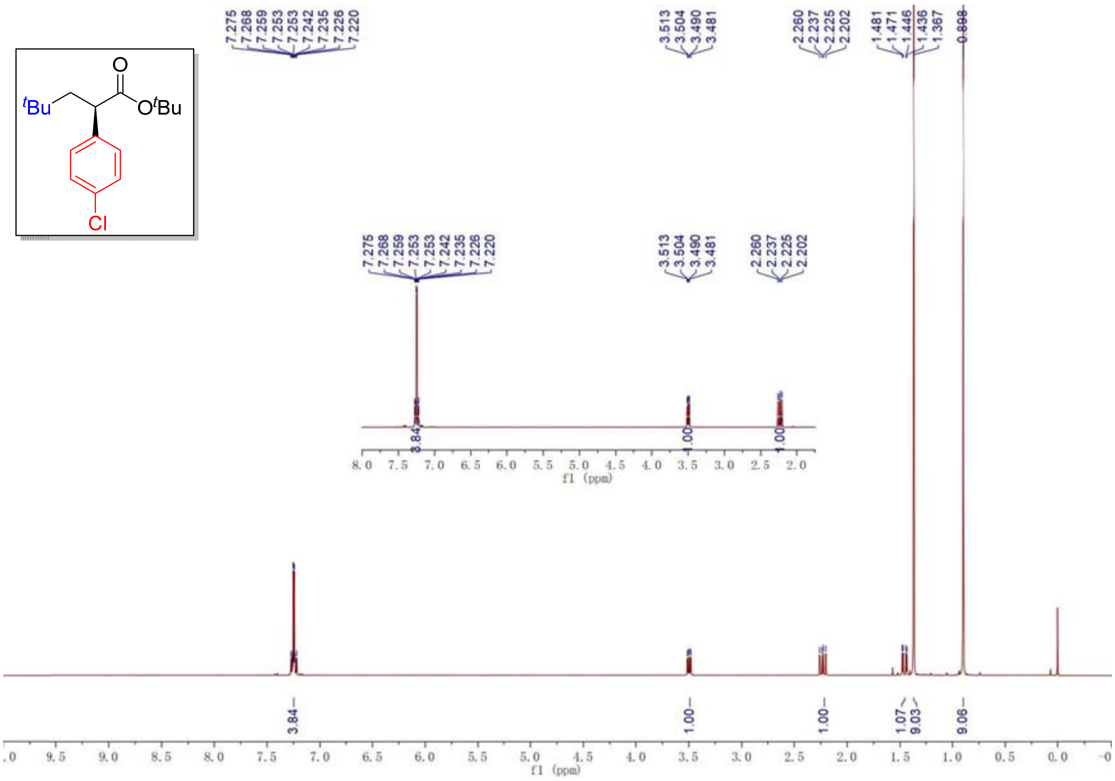

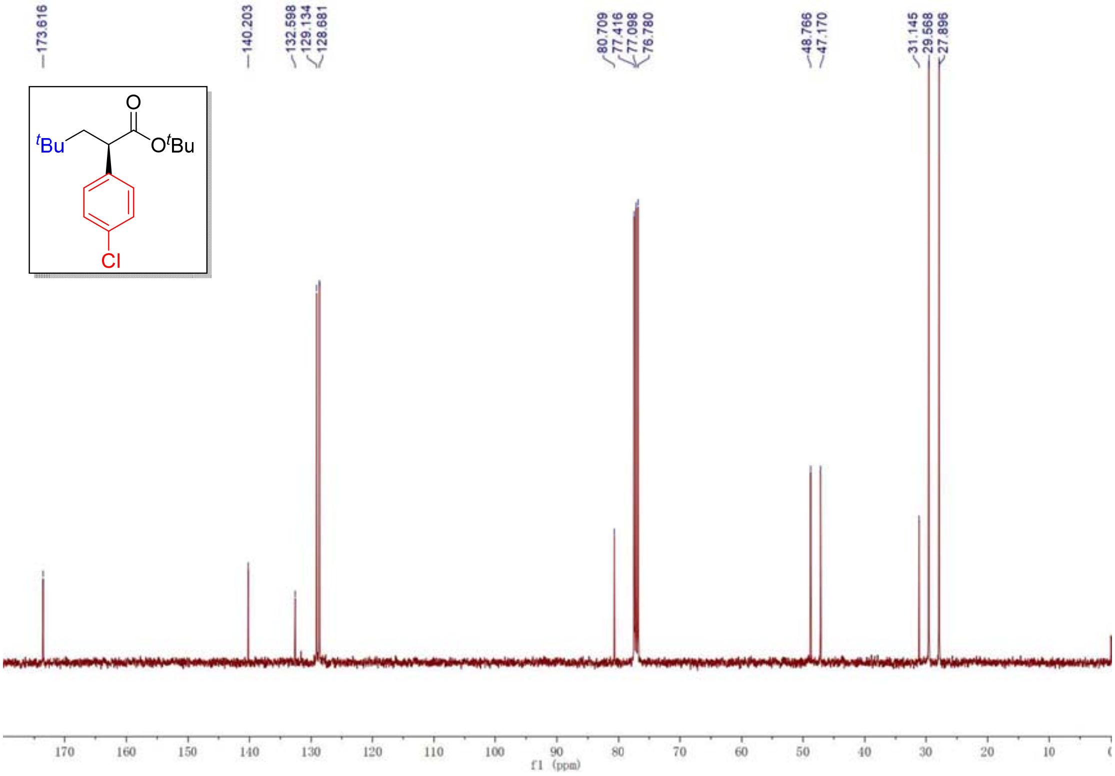

Supplementary Figure 8. ^{1}**H (400 MHz) and** ^{13}**C {**^{1}**H} (101 MHz) NMR spectra of 4h in** $CDCl_3$

(*R*)-tert-butyl 4,4-dimethyl-2-phenylpentanoate (4i)

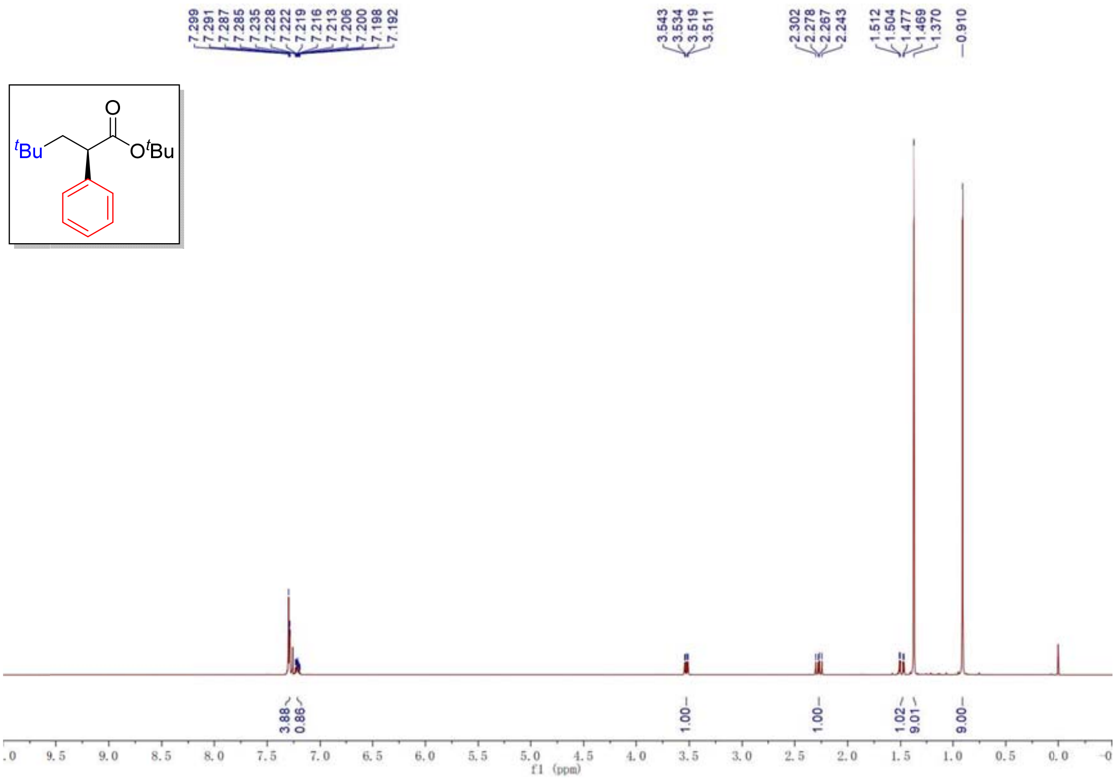

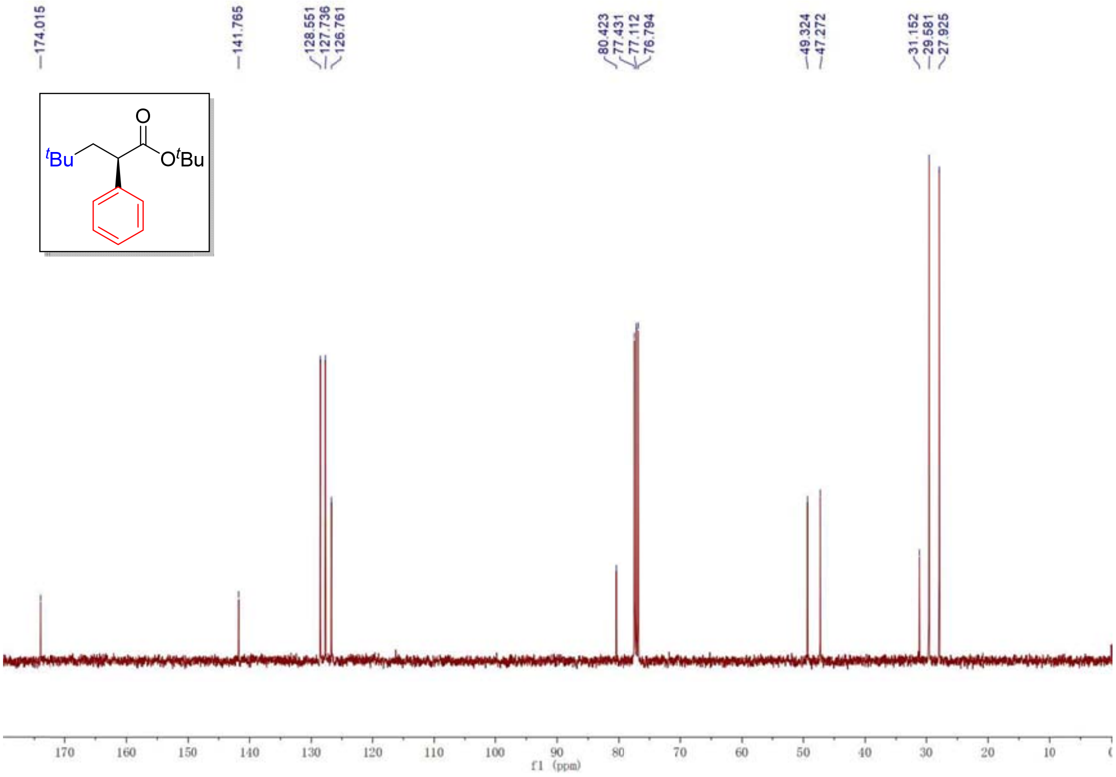

Supplementary Figure 9. ^{1}**H (400 MHz) and** ^{13}**C {**^{1}**H} (101 MHz) NMR spectra of 4i in** $CDCl_3$

(*R*)-*tert*-butyl 2-([1,1'-biphenyl]-4-yl)-4,4-dimethylpentanoate (4j)

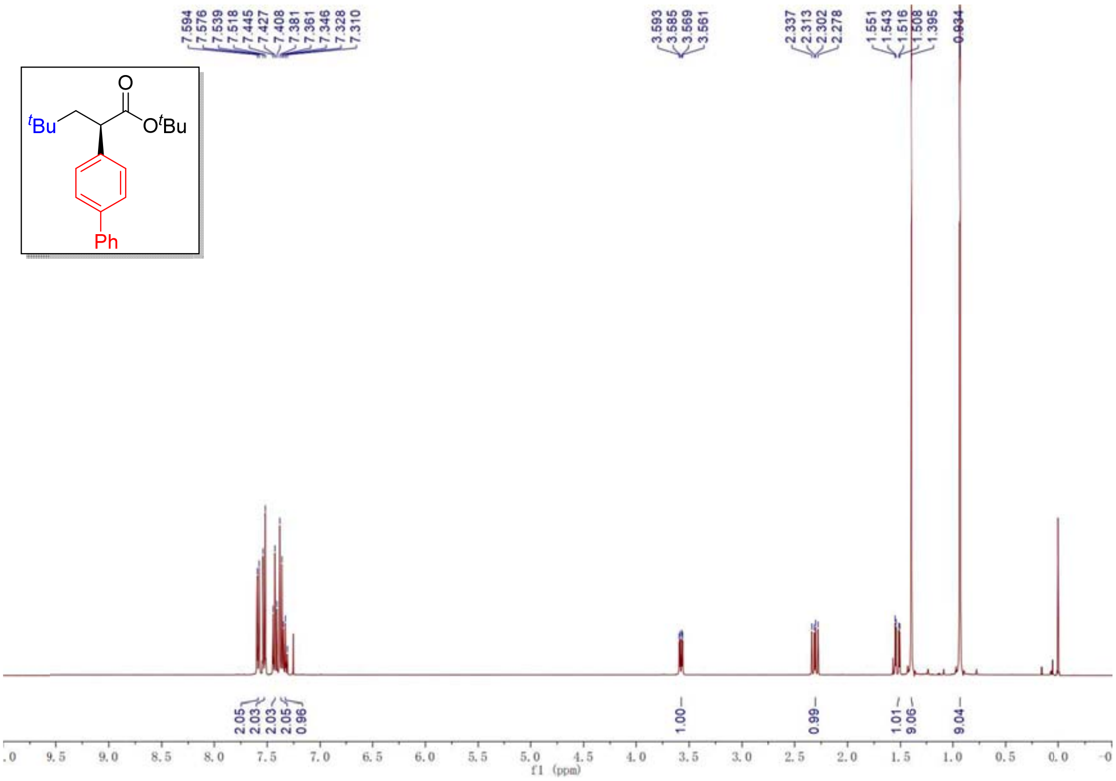

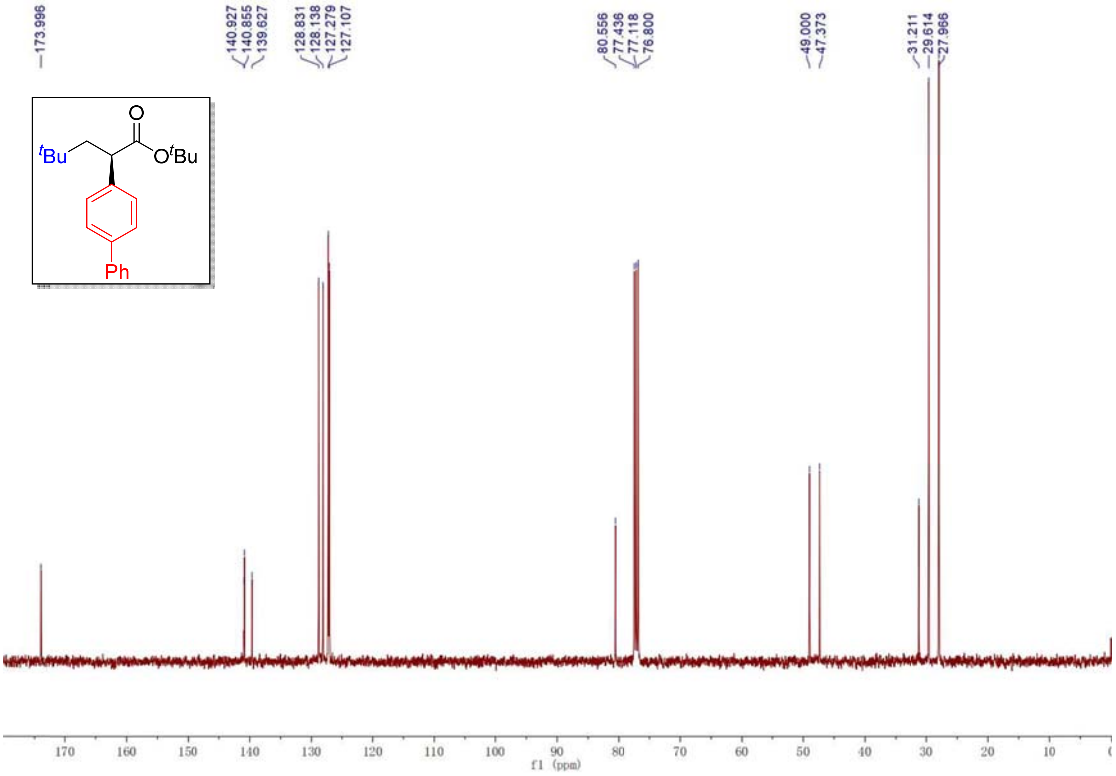

Supplementary Figure 10. ^{1}**H (400 MHz) and** ^{13}**C {**^{1}**H} (101 MHz) NMR spectra of 4j in CDCl**$_3$

(*R*)-*tert*-butyl 2-(4-(tert-butyl)phenyl)-4,4-dimethylpentanoate (4k)

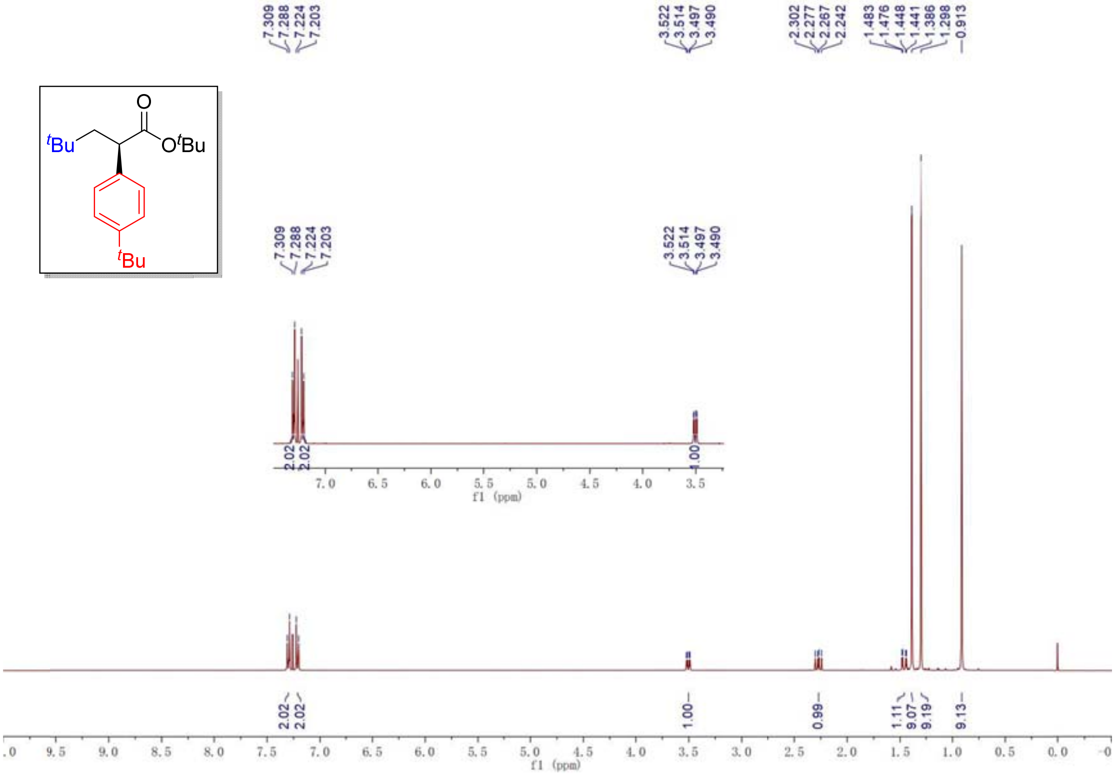

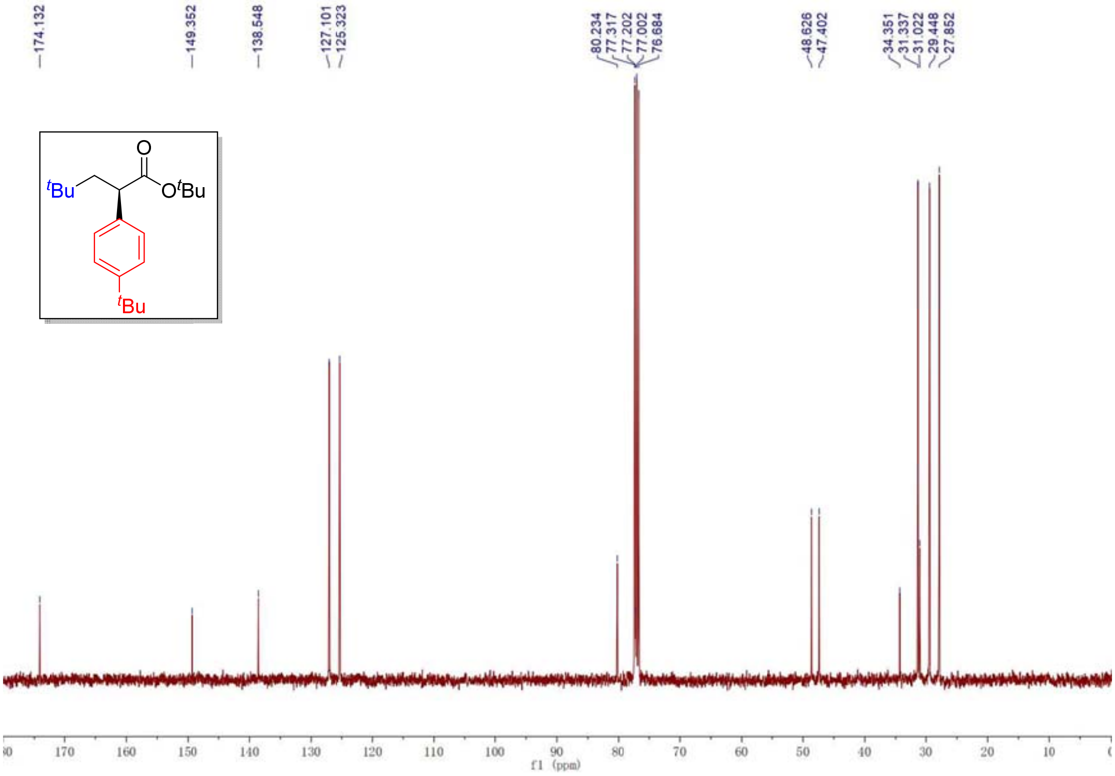

Supplementary Figure 11. ^{1}H **(400 MHz) and** ^{13}C **{**^{1}H**} (101 MHz) NMR spectra of 4k in** $CDCl_3$

(*R*)-*tert*-butyl 2-(4-methoxyphenyl)-4,4-dimethylpentanoate (4l)

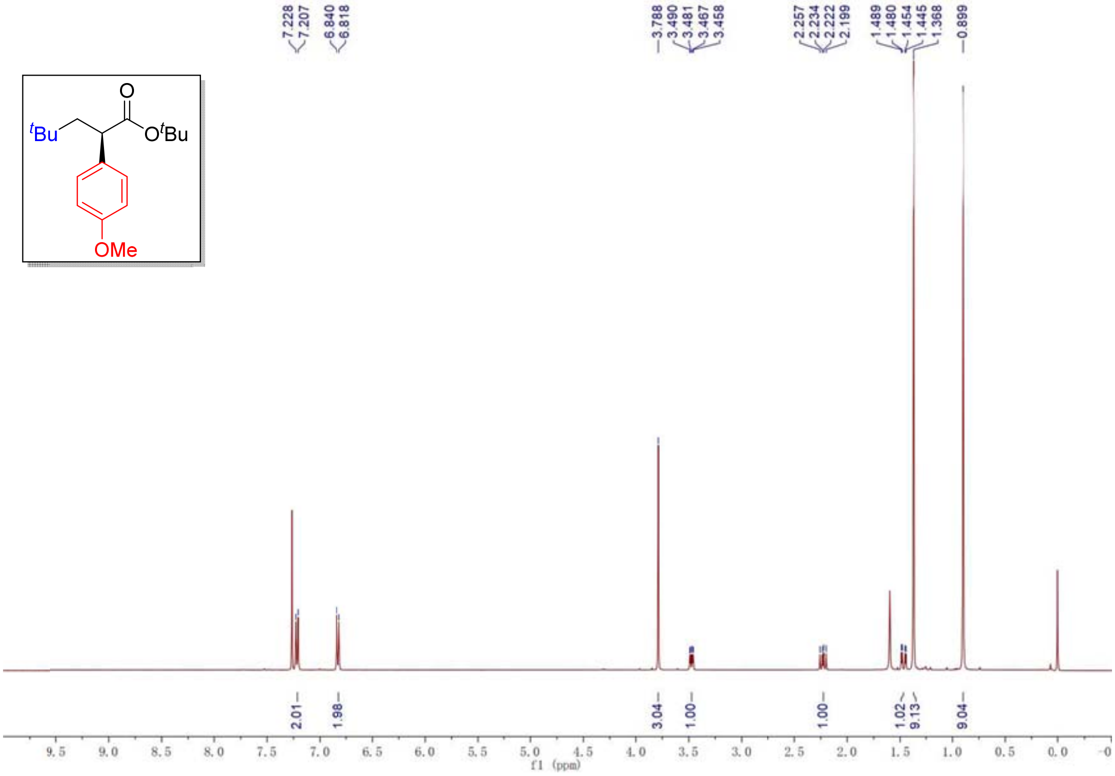

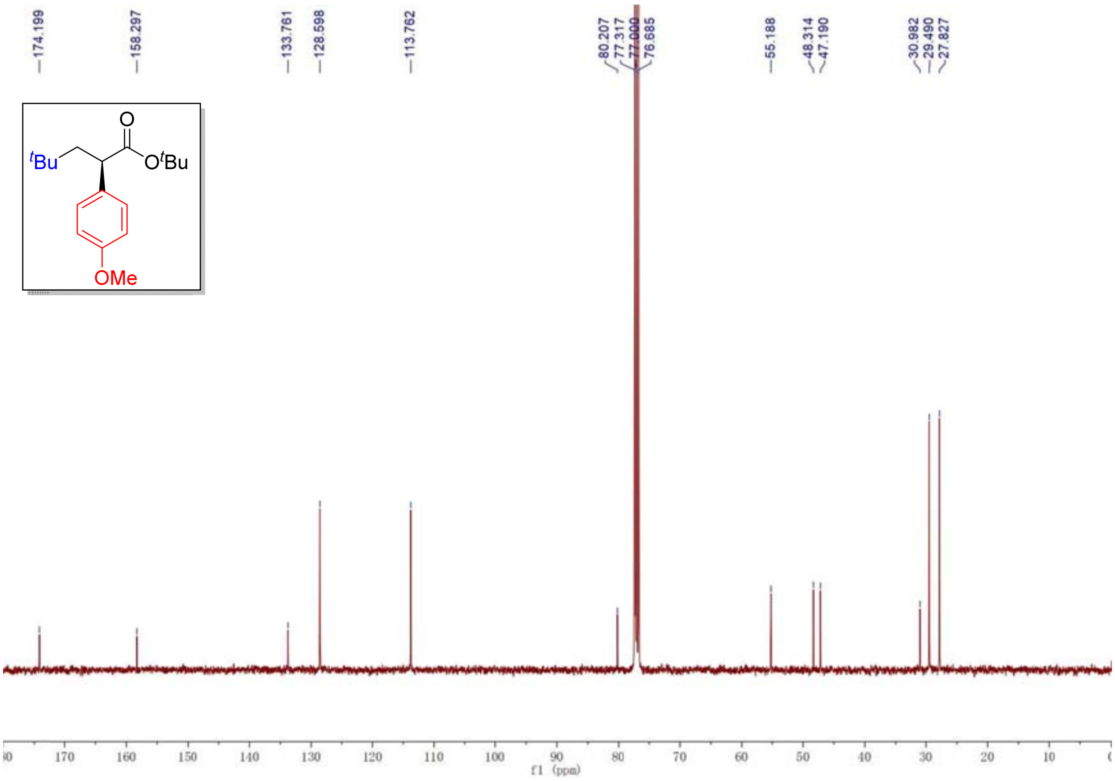

Supplementary Figure 12. **^{1}H (400 MHz) and ^{13}C {^{1}H} (101 MHz) NMR spectra of 4l in $CDCl_3$**

(*R*)-*tert*-butyl 4,4-dimethyl-2-(4-(methylthio)phenyl)pentanoate (4m)

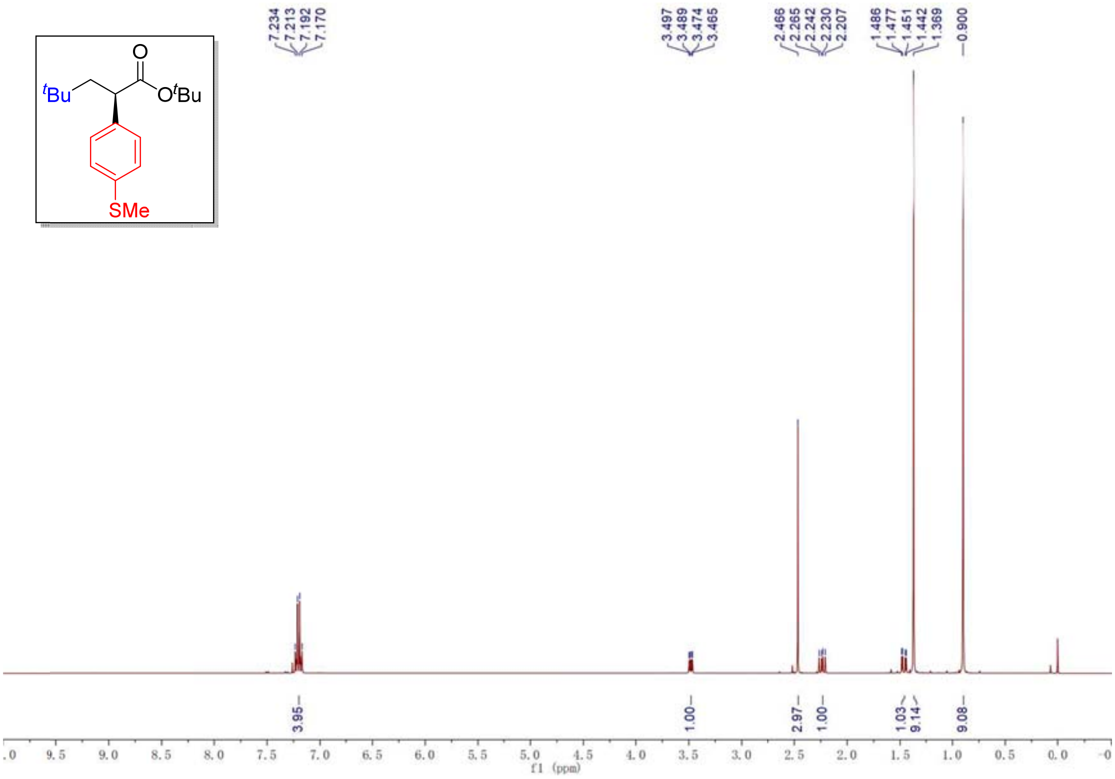

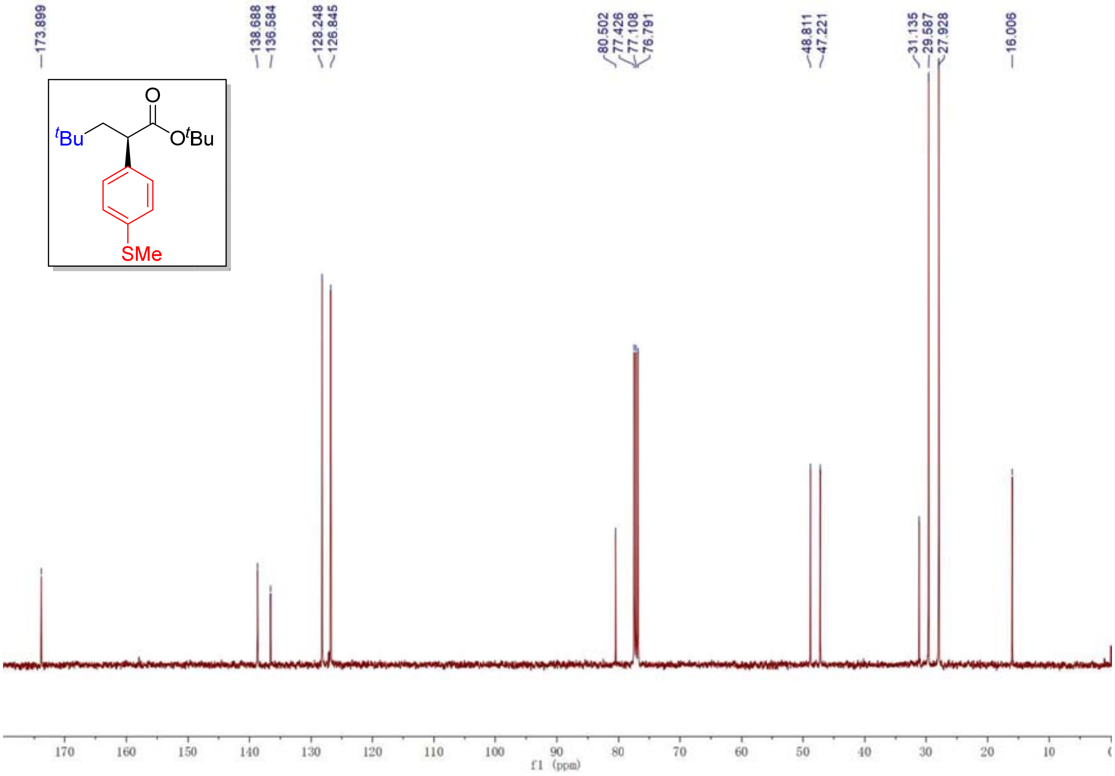

Supplementary Figure 13. ^{1}**H (400 MHz) and** ^{13}**C {**^{1}**H} (101 MHz) NMR spectra of 4m in** $CDCl_3$

(*R*)-*tert*-butyl 2-(4-(dimethylamino)phenyl)-4,4-dimethylpentanoate (4n)

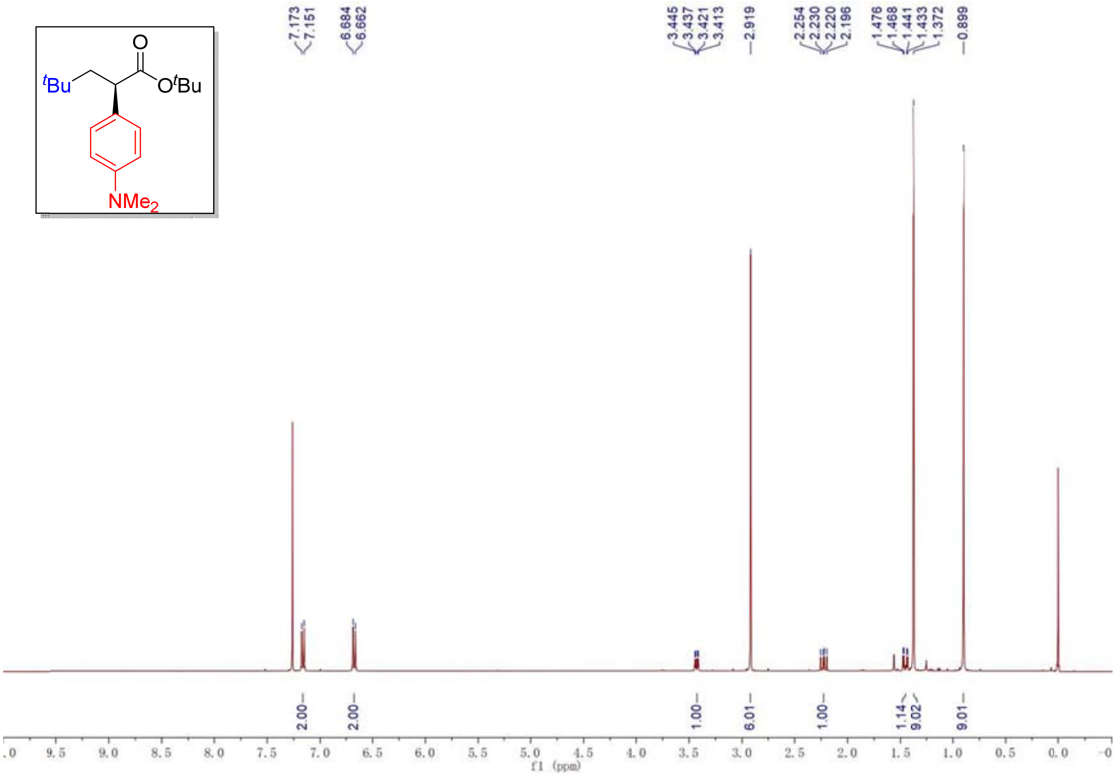

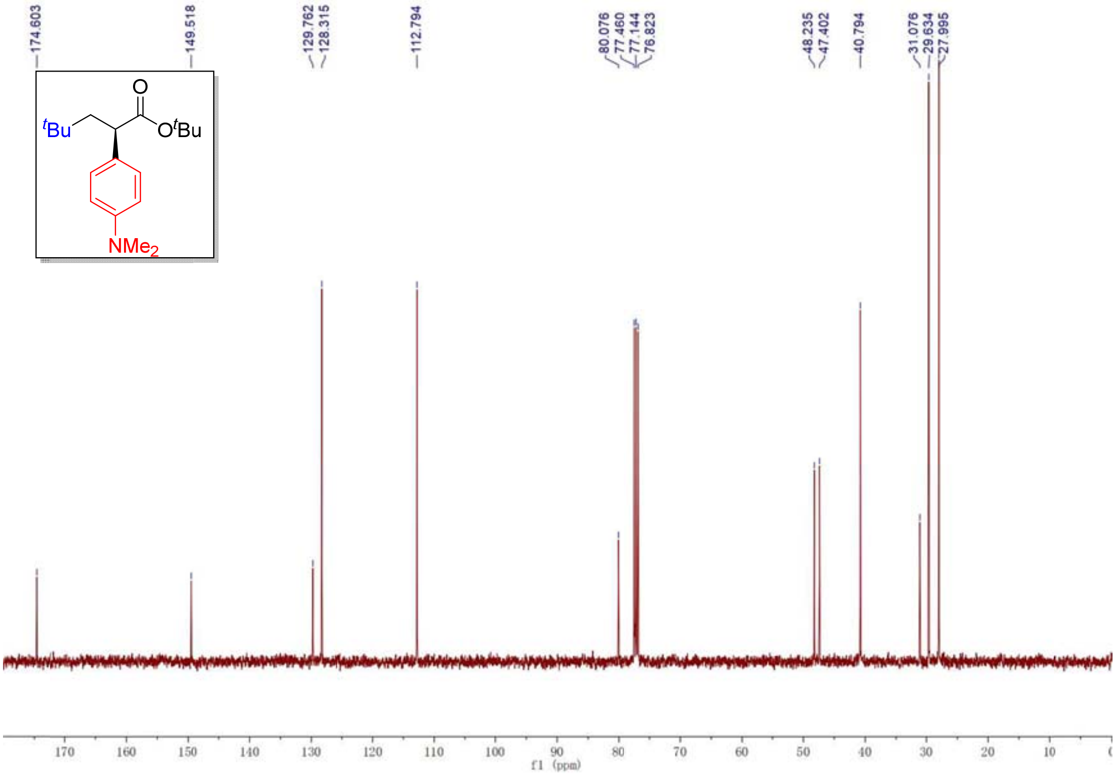

Supplementary Figure 14. ^{1}H **(400 MHz) and** ^{13}C **{**^{1}H**} (101 MHz) NMR spectra of 4n in** $CDCl_3$

(*R*)-*tert*-butyl 4,4-dimethyl-2-(4-(4,4,5,5-tetramethyl-1,3,2-dioxaborolan-2-yl)phenyl)pentanoate (4o)

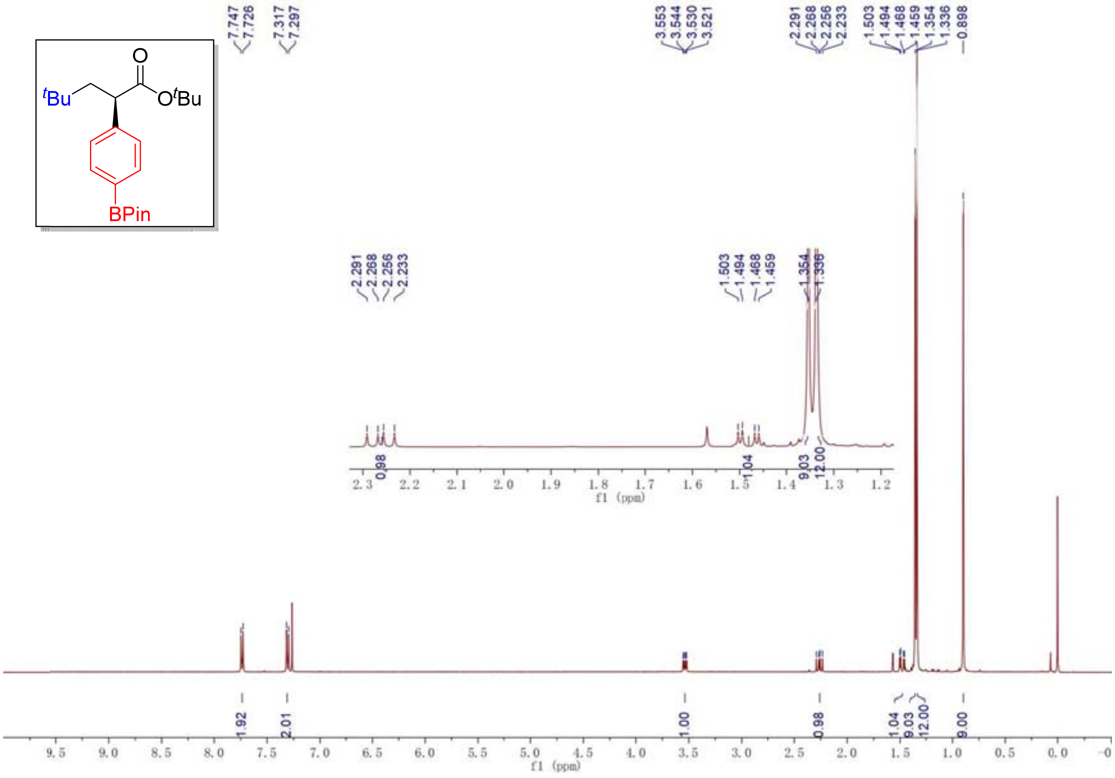

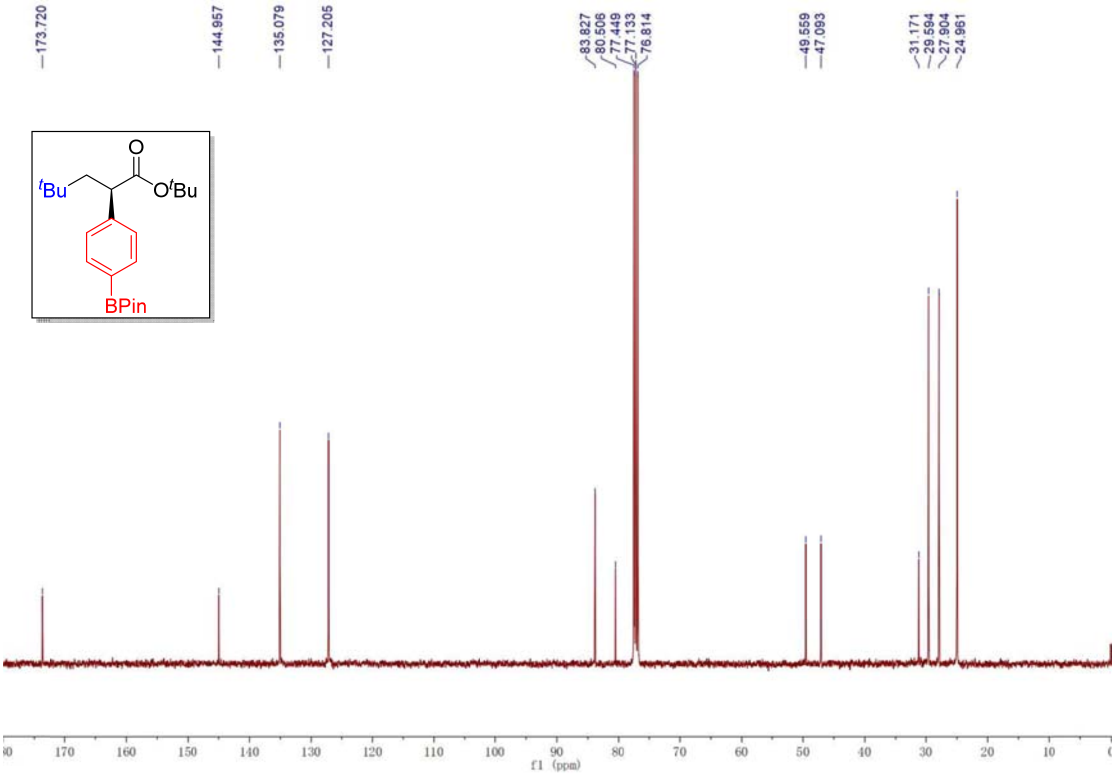

Supplementary Figure 15. ^{1}**H (400 MHz) and** ^{13}**C {**^{1}**H} (101 MHz) NMR spectra of 4o in** $CDCl_3$

(*R*)-*tert*-butyl2-(3-acetylphenyl)-4,4-dimethylpentanoate (4p)

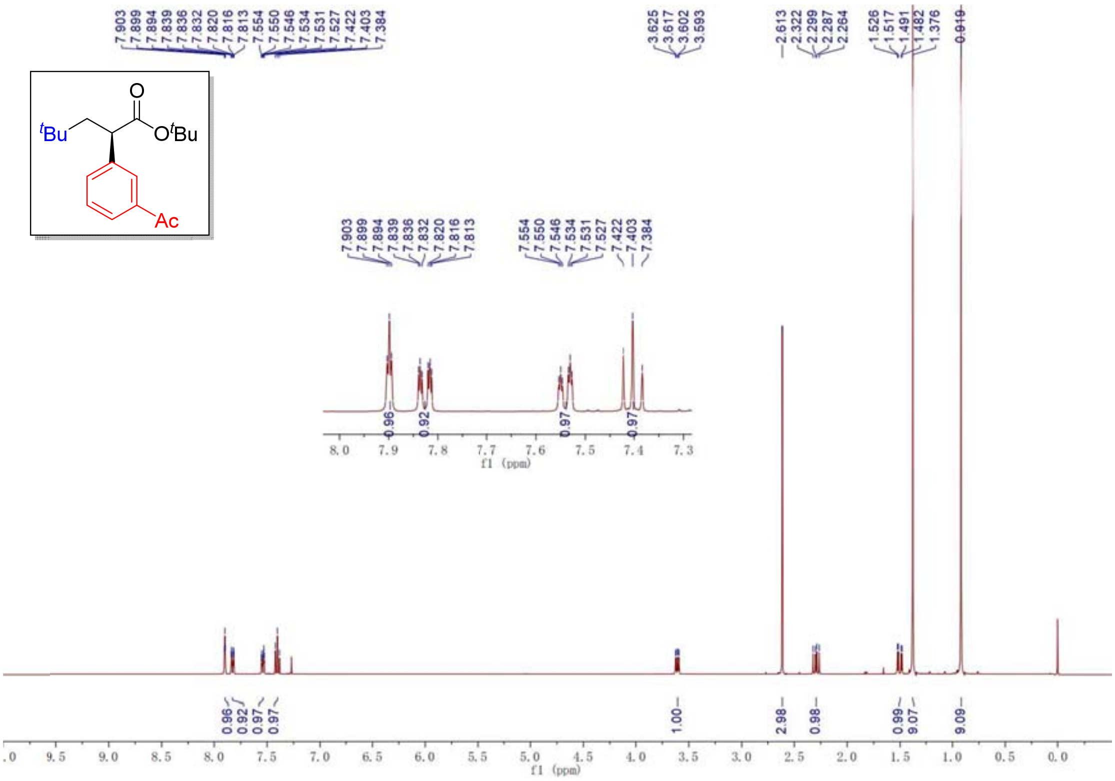

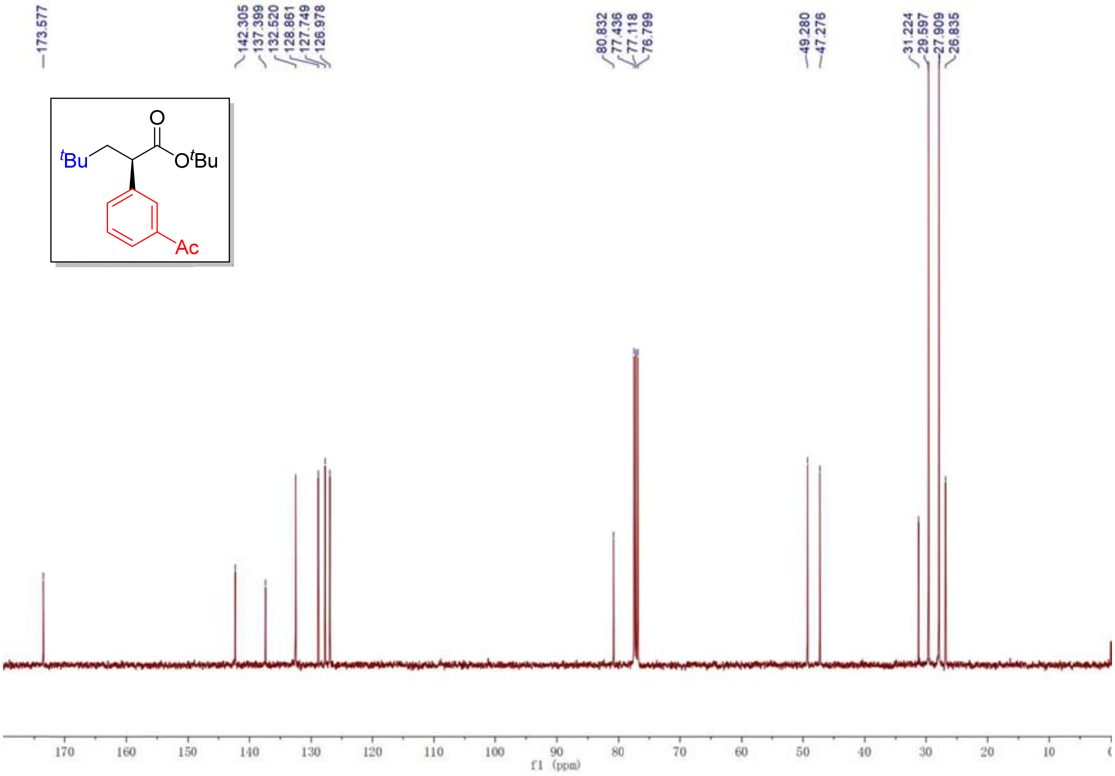

Supplementary Figure 16. ^{1}H (400 MHz) and ^{13}C {^{1}H} (101 MHz) NMR spectra of 4p in $CDCl_3$

(*R*)-*tert*-butyl 2-(3-methoxyphenyl)-4,4-dimethylpentanoate (4q)

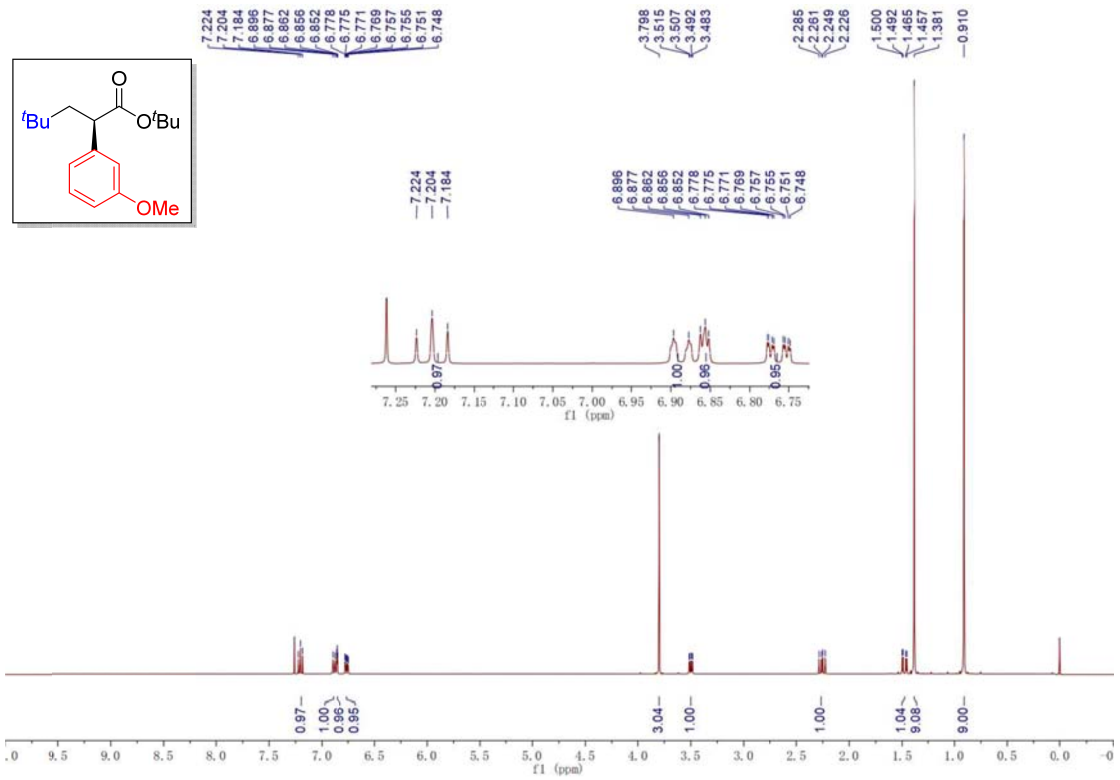

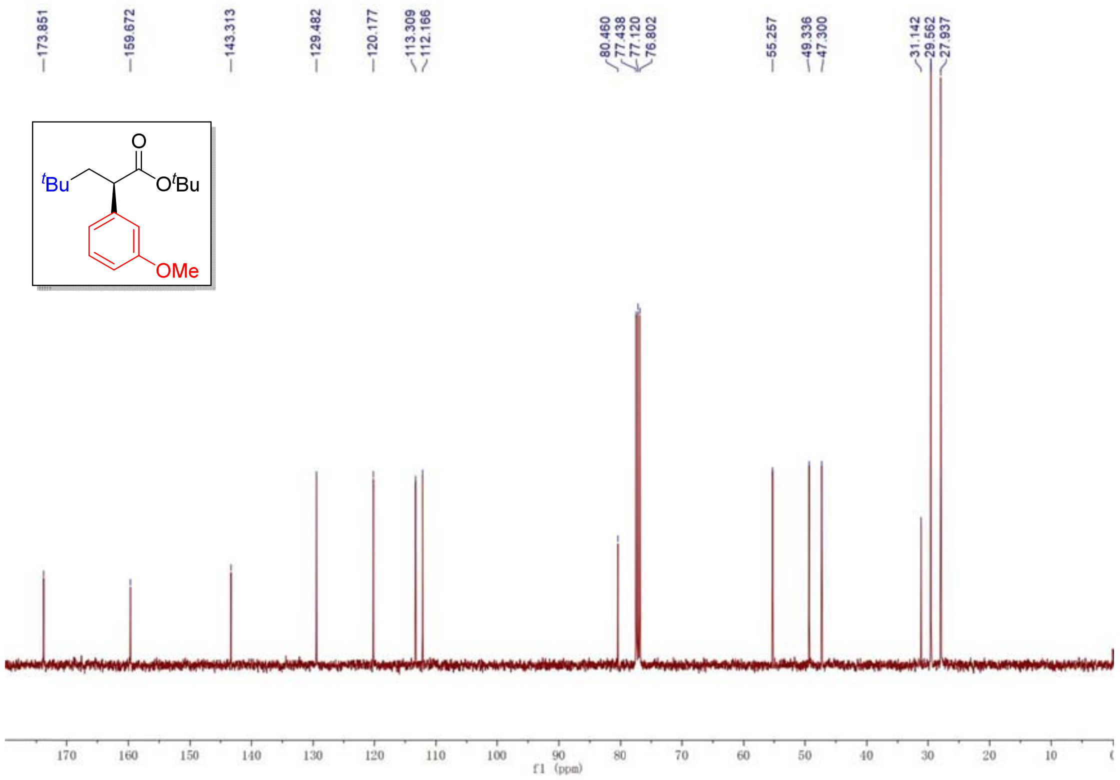

Supplementary Figure 17. ^{1}**H (400 MHz) and** ^{13}**C {**^{1}**H} (101 MHz) NMR spectra of 4q in** $CDCl_3$

(*R*)-*tert*-butyl 2-(3-fluoro-4-methylphenyl)-4,4-dimethylpentanoate (4r)

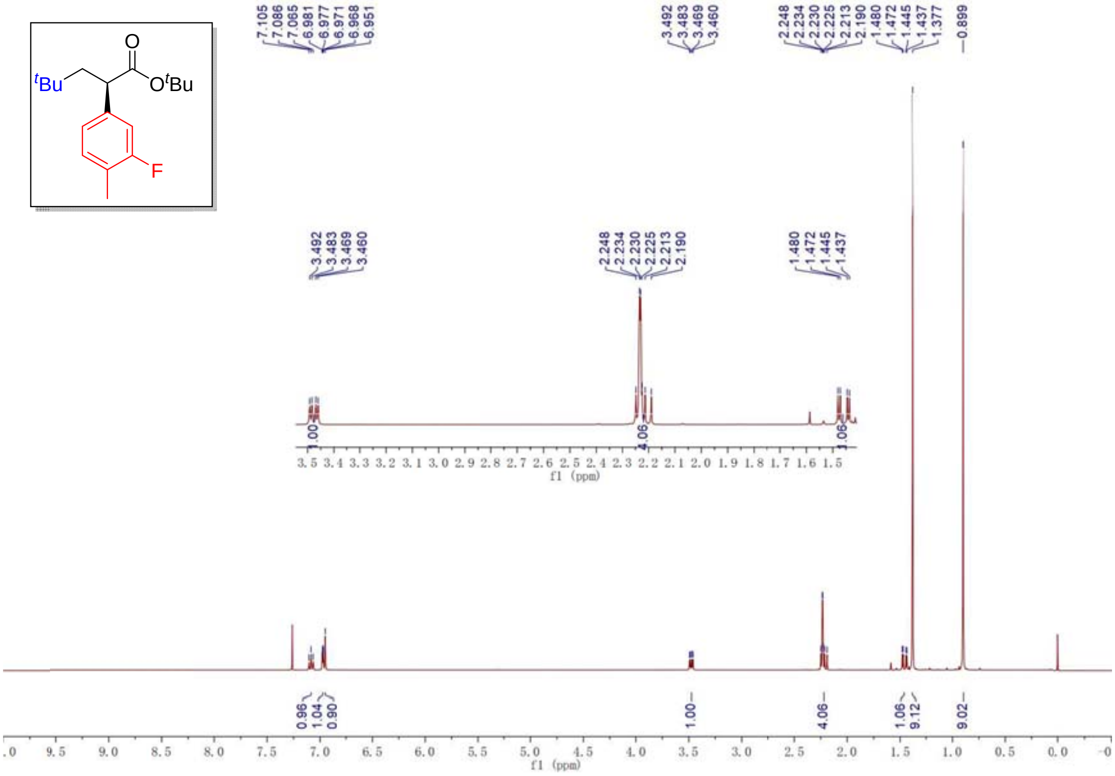

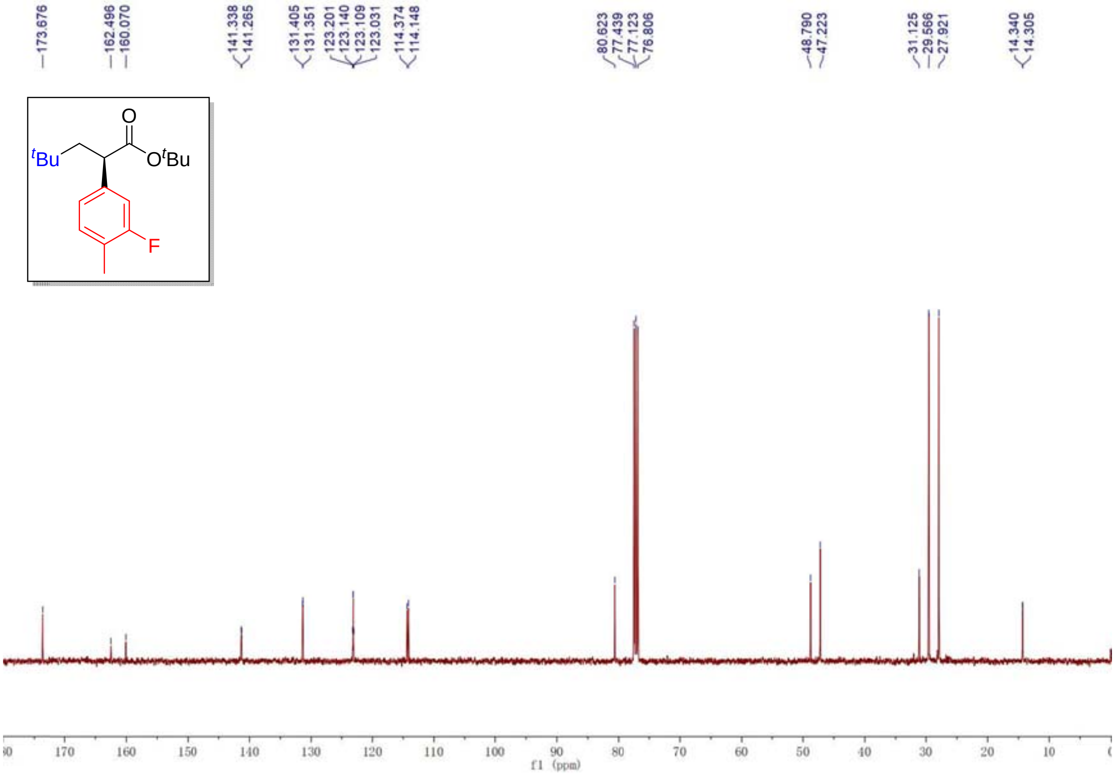

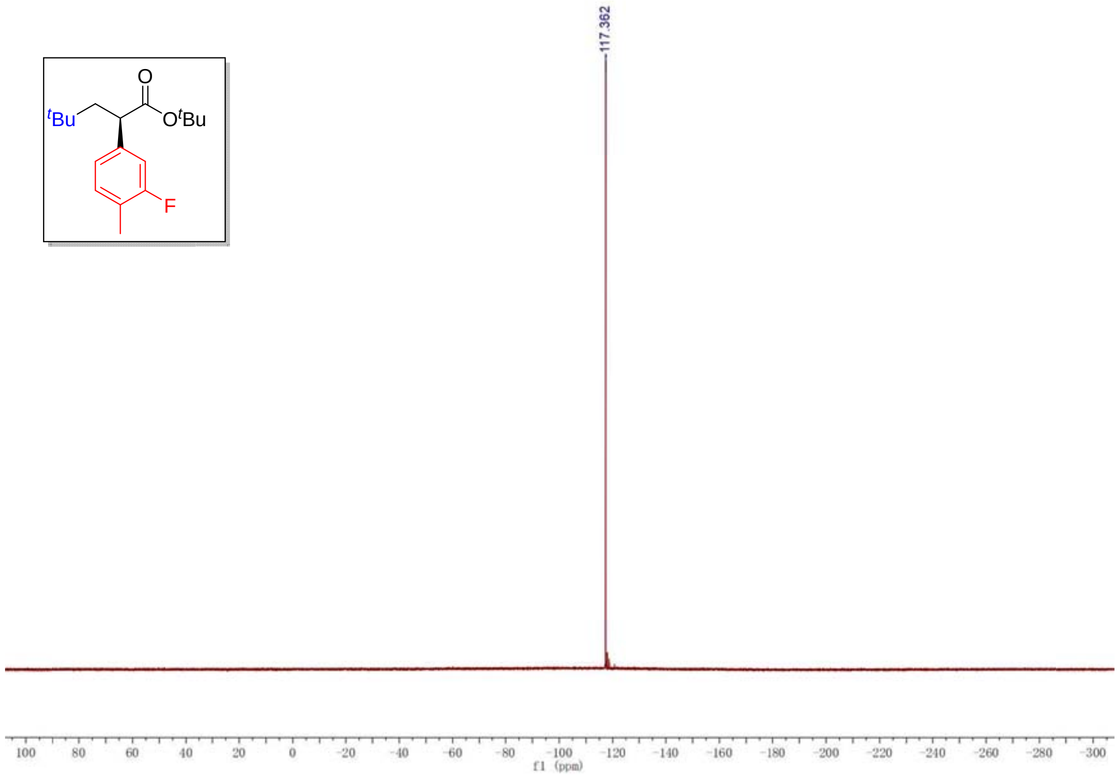

Supplementary Figure 18. ^{1}**H (400 MHz),** ^{13}**C {**^{1}**H} (101 MHz) and** ^{19}**F NMR spectra of 4r in** $CDCl_3$

(*R*)-*tert*-butyl 2-(2-fluoro-[1,1'-biphenyl]-4-yl)-4,4-dimethylpentanoate (4s)

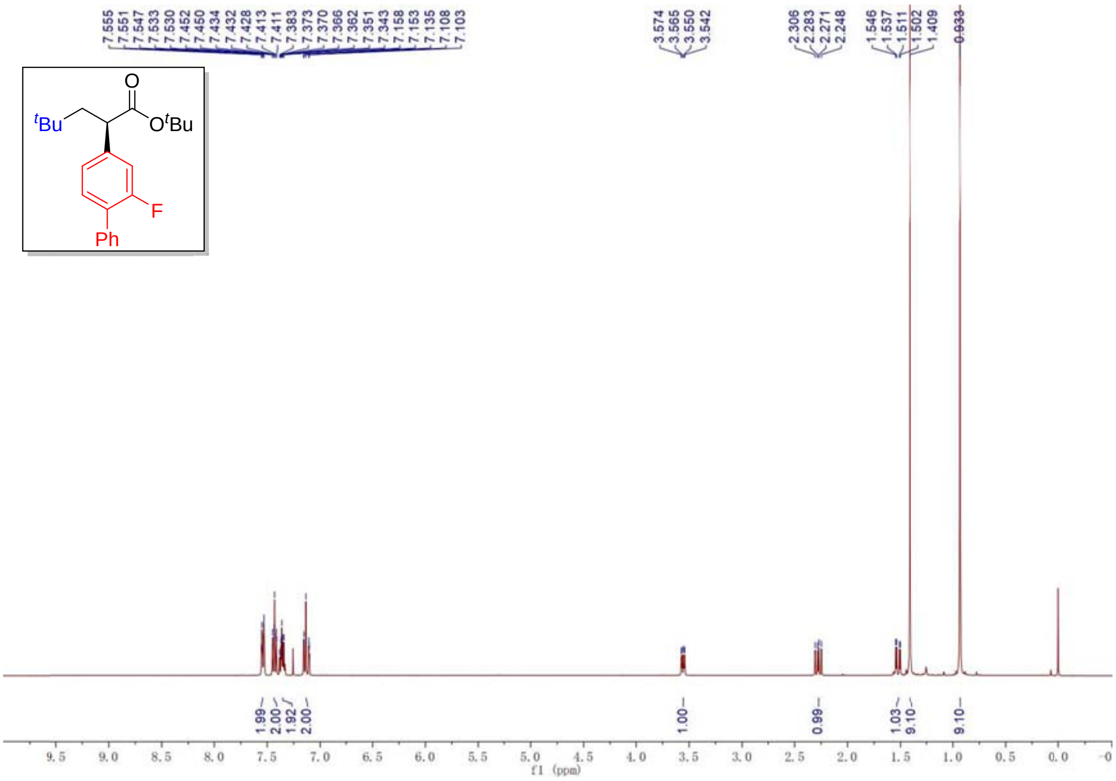

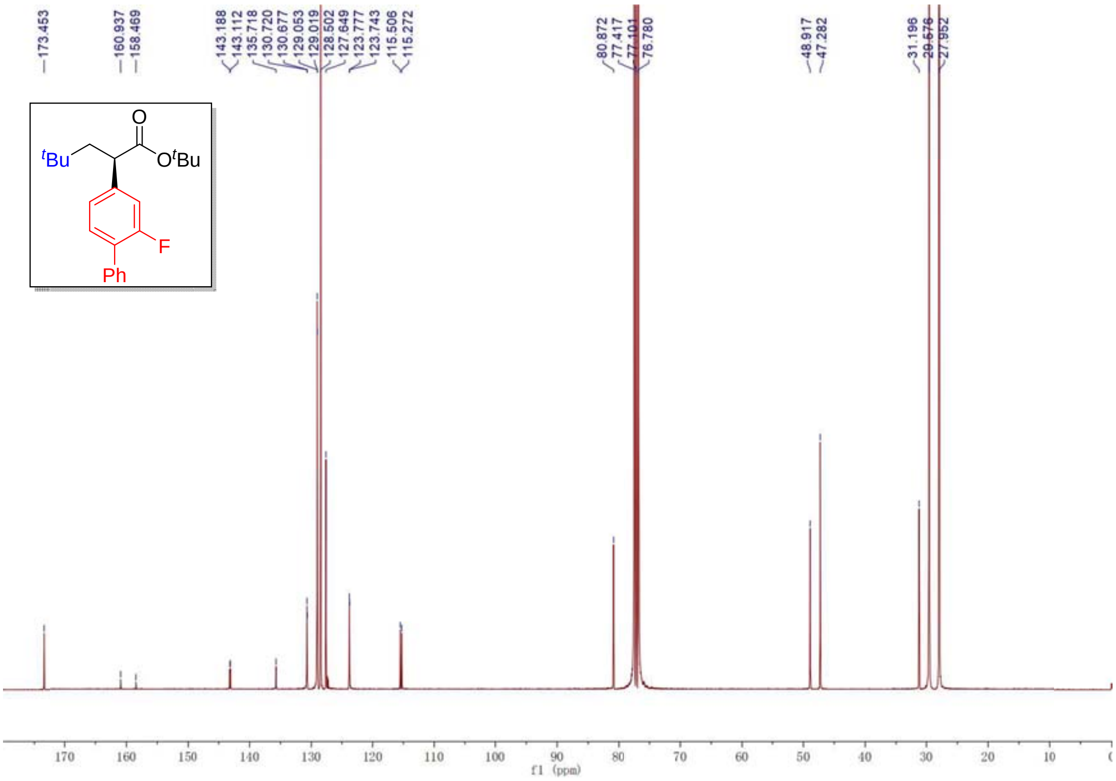

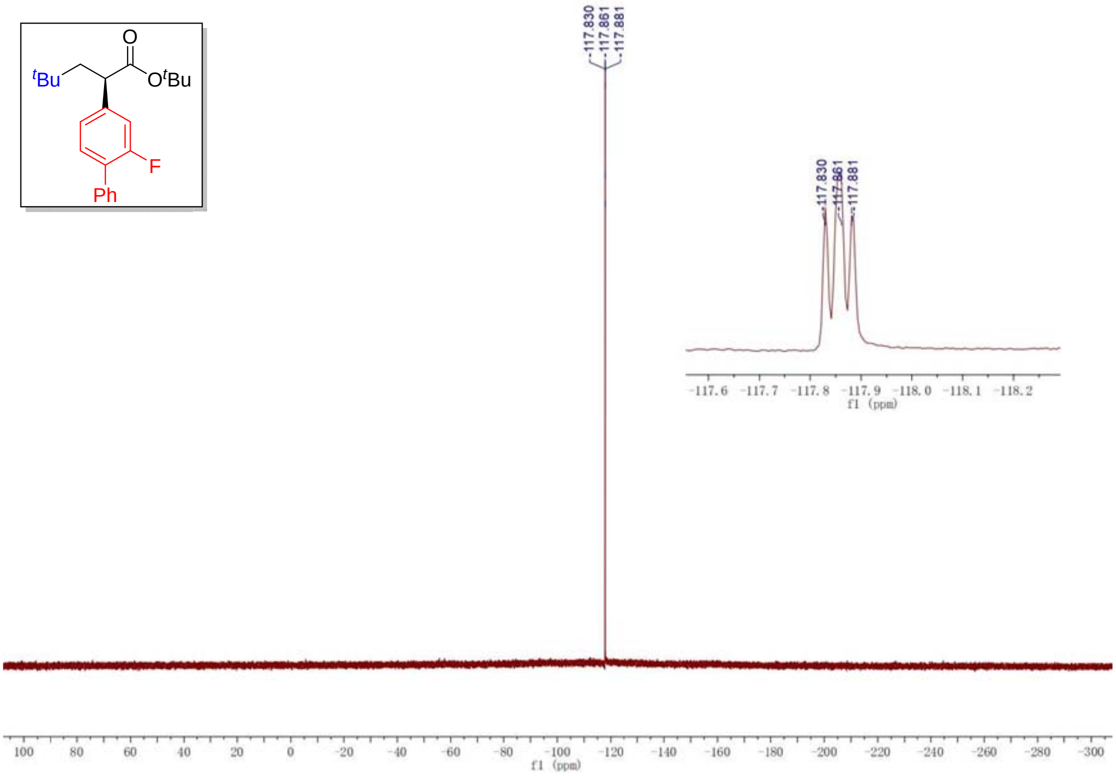

Supplementary Figure 19. ^{1}H (400 MHz), ^{13}C {^{1}H} (101 MHz) and ^{19}F NMR spectra of 4s in $CDCl_3$

(*R*)-*tert*-butyl 2-(6-methoxynaphthalen-2-yl)-4,4-dimethylpentanoate (4t)

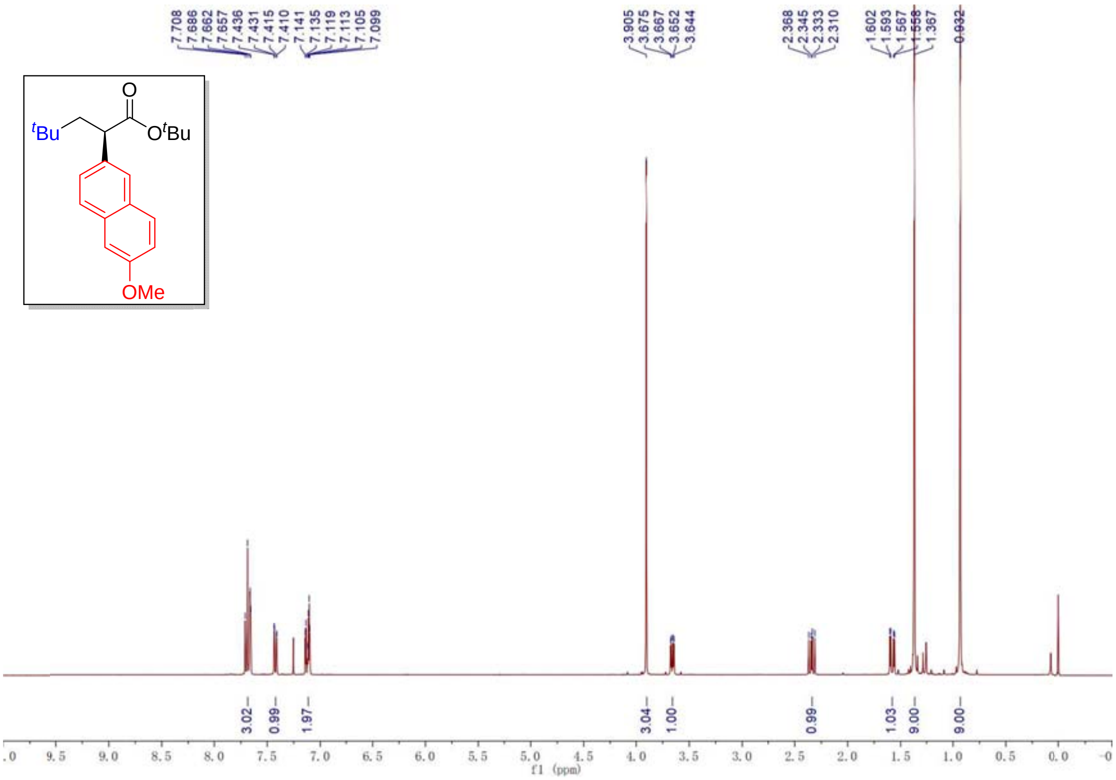

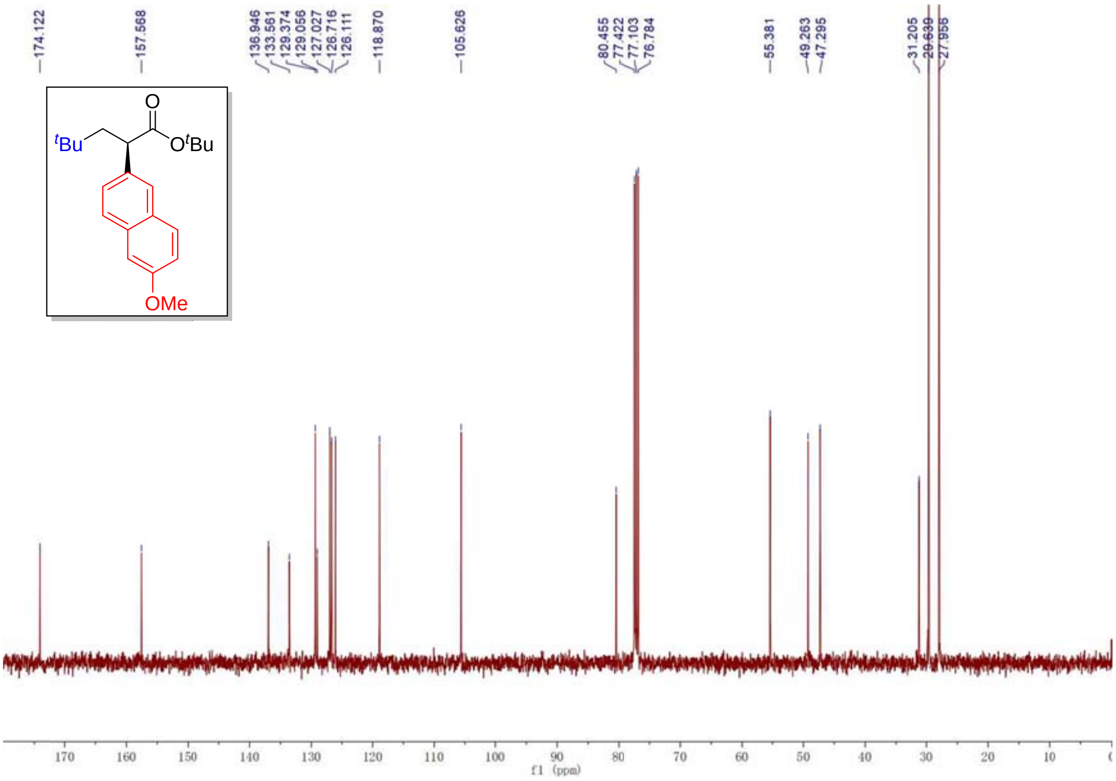

Supplementary Figure 20. ^{1}H (400 MHz) and ^{13}C {^{1}H} (101 MHz) NMR spectra of 4t in $CDCl_3$

(*R*)-*tert*-butyl 4,4-dimethyl-2-(naphthalen-2-yl)pentanoate (4u)

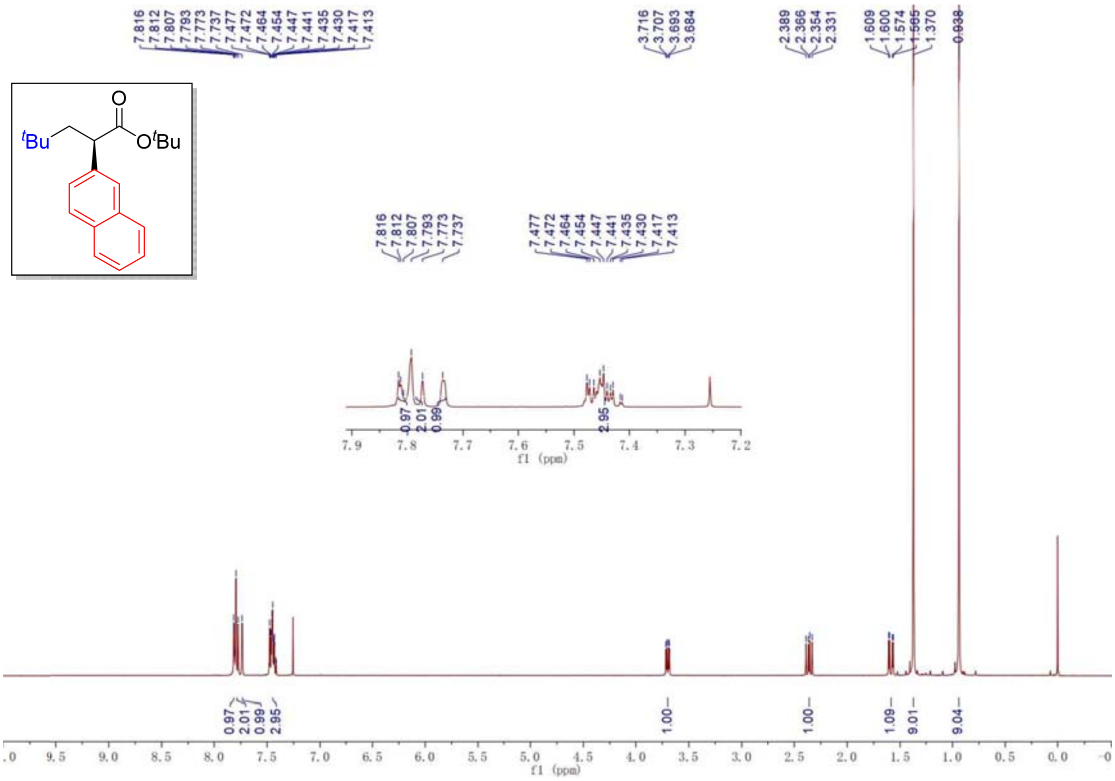

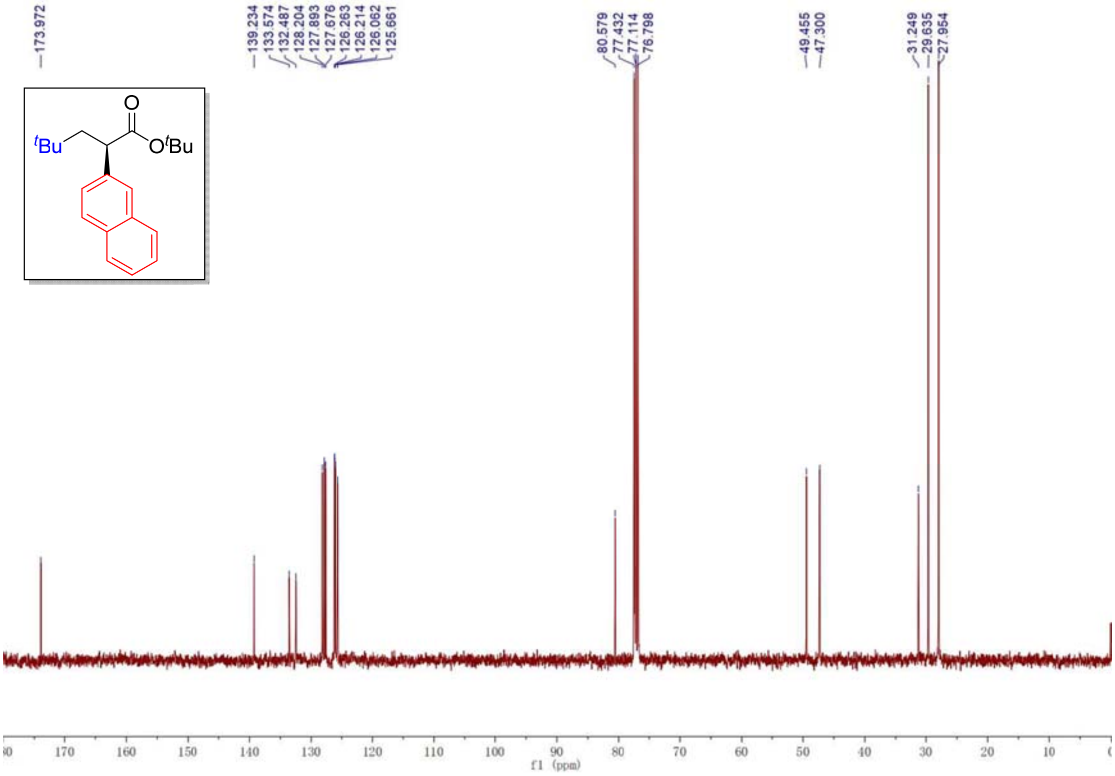

Supplementary Figure 21. **^{1}H (400 MHz) and ^{13}C {^{1}H} (101 MHz) NMR spectra of 4u in $CDCl_3$**

(*R*)-*tert*-butyl 2-(dibenzo[b,d]furan-2-yl)-4,4-dimethylpentanoate (4v)

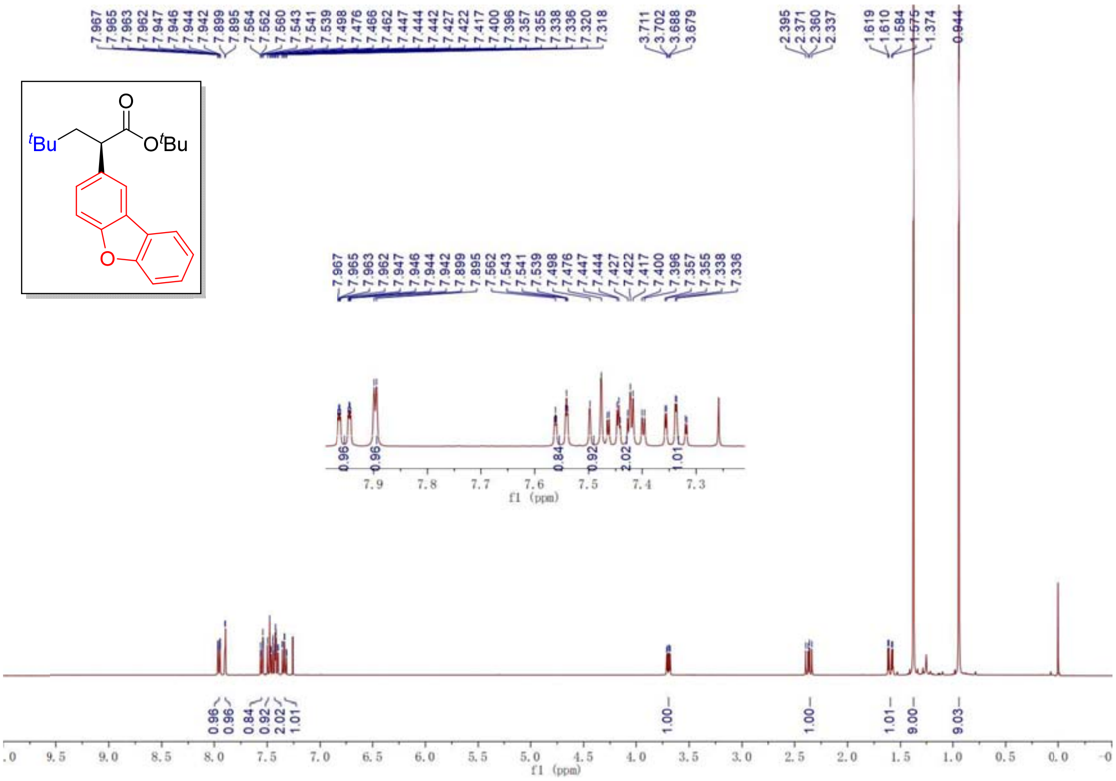

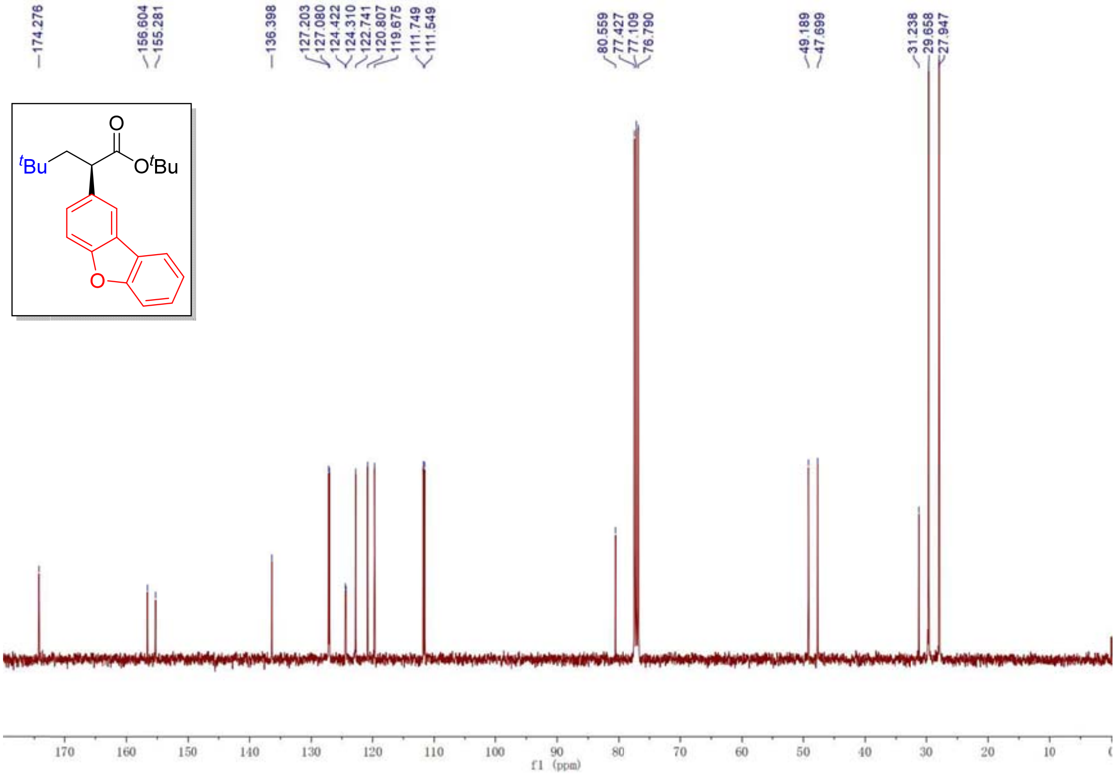

Supplementary Figure 22. ^{1}**H (400 MHz) and** ^{13}**C {**^{1}**H} (101 MHz) NMR spectra of 4v in** $CDCl_3$

(*R*)-*tert*-butyl 2-(2,3-dihydrobenzofuran-5-yl)-4,4-dimethylpentanoate (4w)

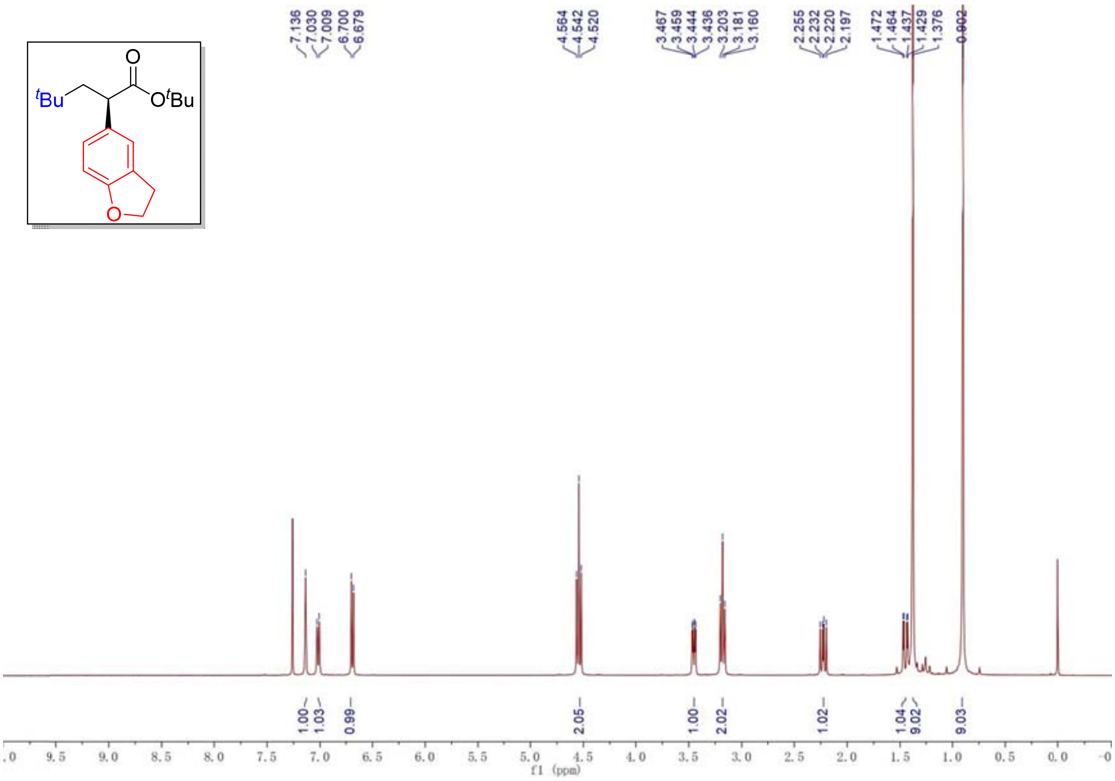

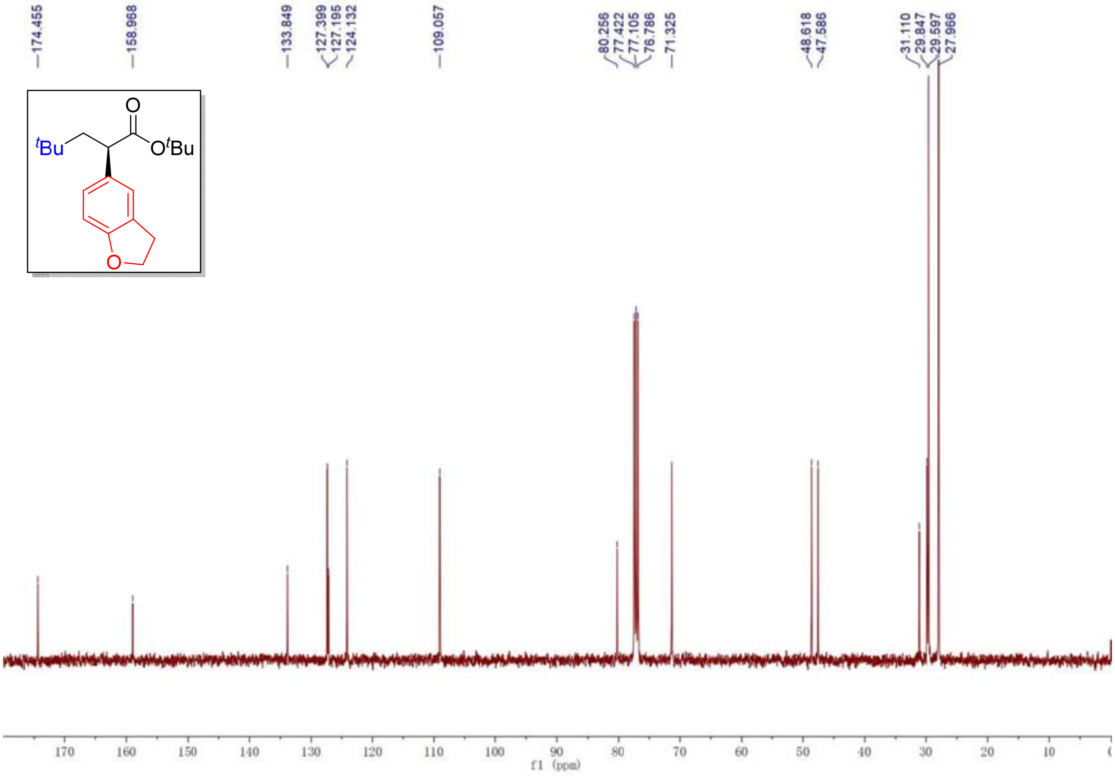

Supplementary Figure 23. ^{1}**H (400 MHz) and** ^{13}**C {**^{1}**H} (101 MHz) NMR spectra of 4w in** $CDCl_3$

(*R*)-*tert*-butyl 2-(benzo[d][1,3]dioxol-5-yl)-4,4-dimethylpentanoate (4x)

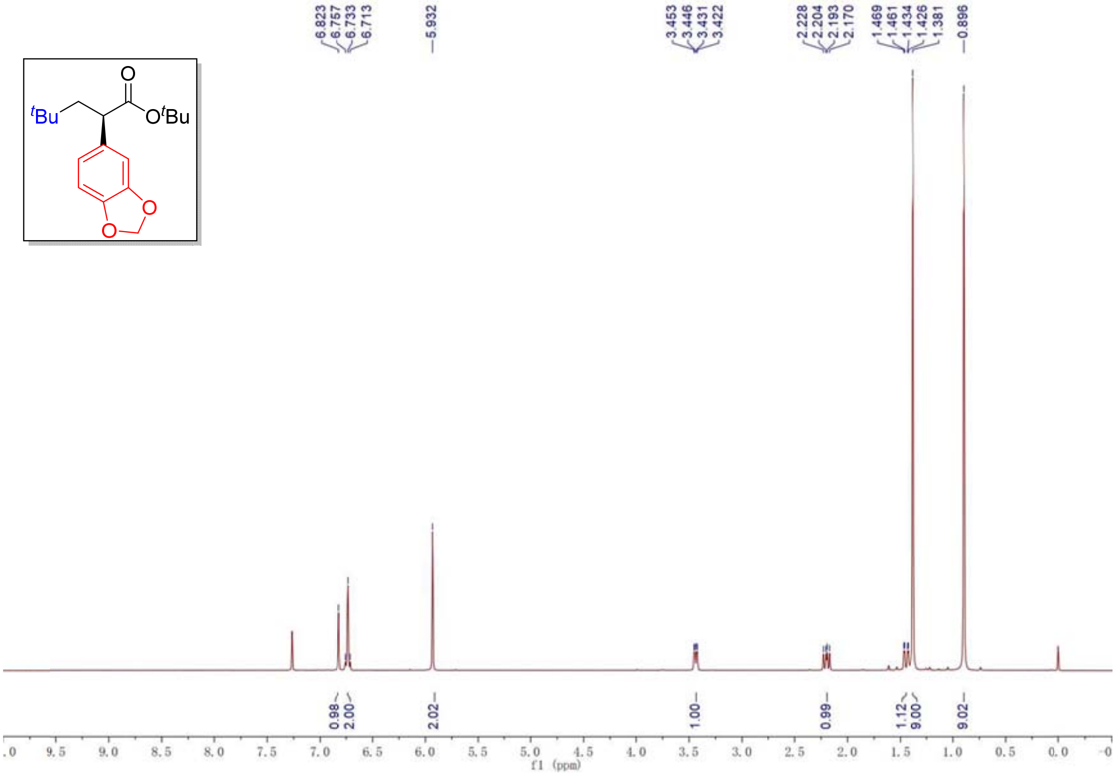

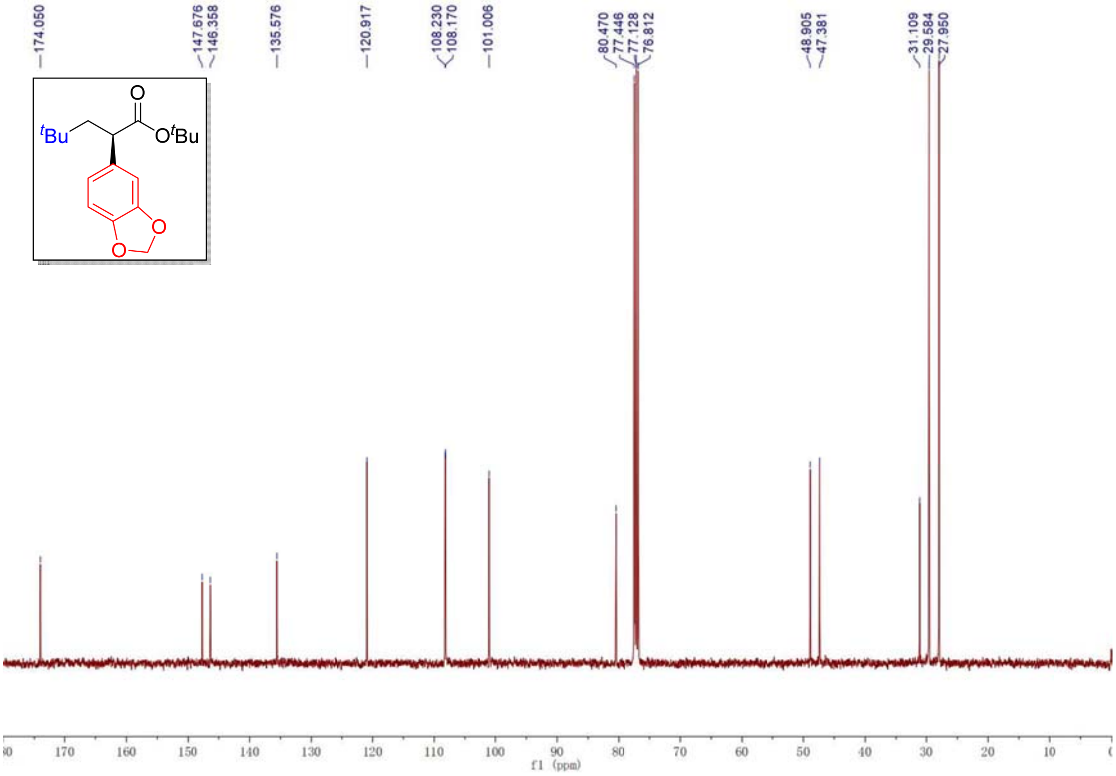

Supplementary Figure 24. **^{1}H (400 MHz) and ^{13}C {^{1}H} (101 MHz) NMR spectra of 4x in $CDCl_3$**

(*R*)-*tert*-butyl 2-(benzofuran-5-yl)-4,4-dimethylpentanoate (4y)

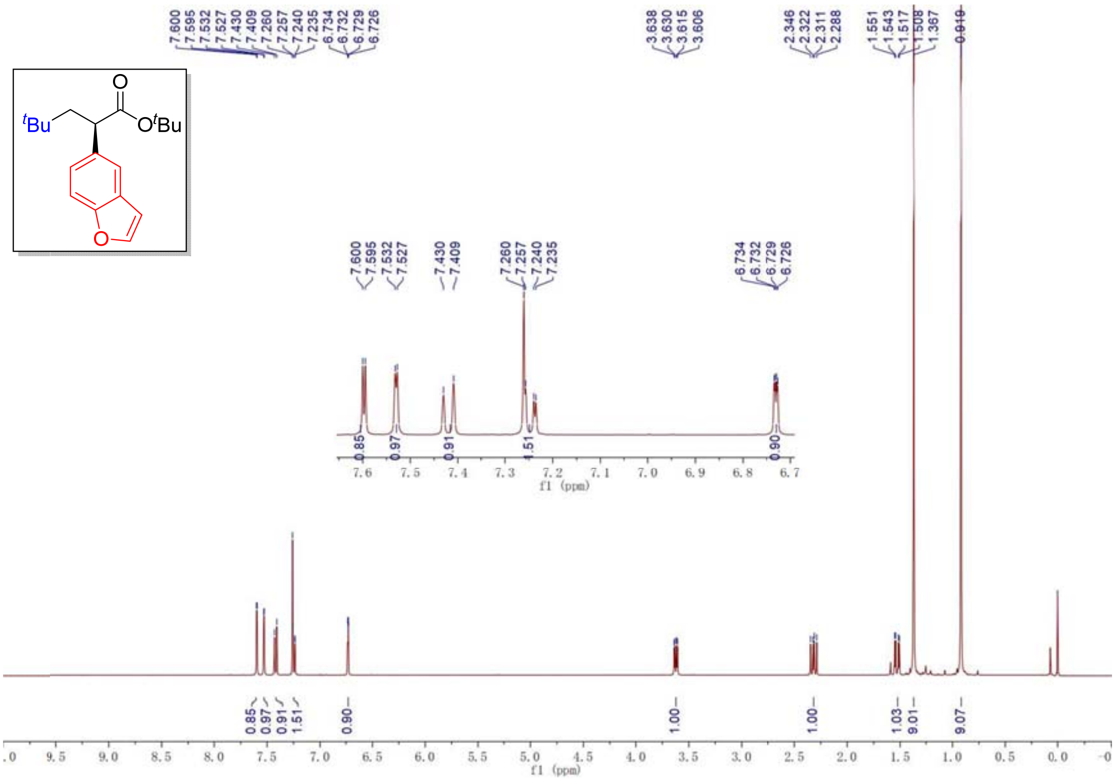

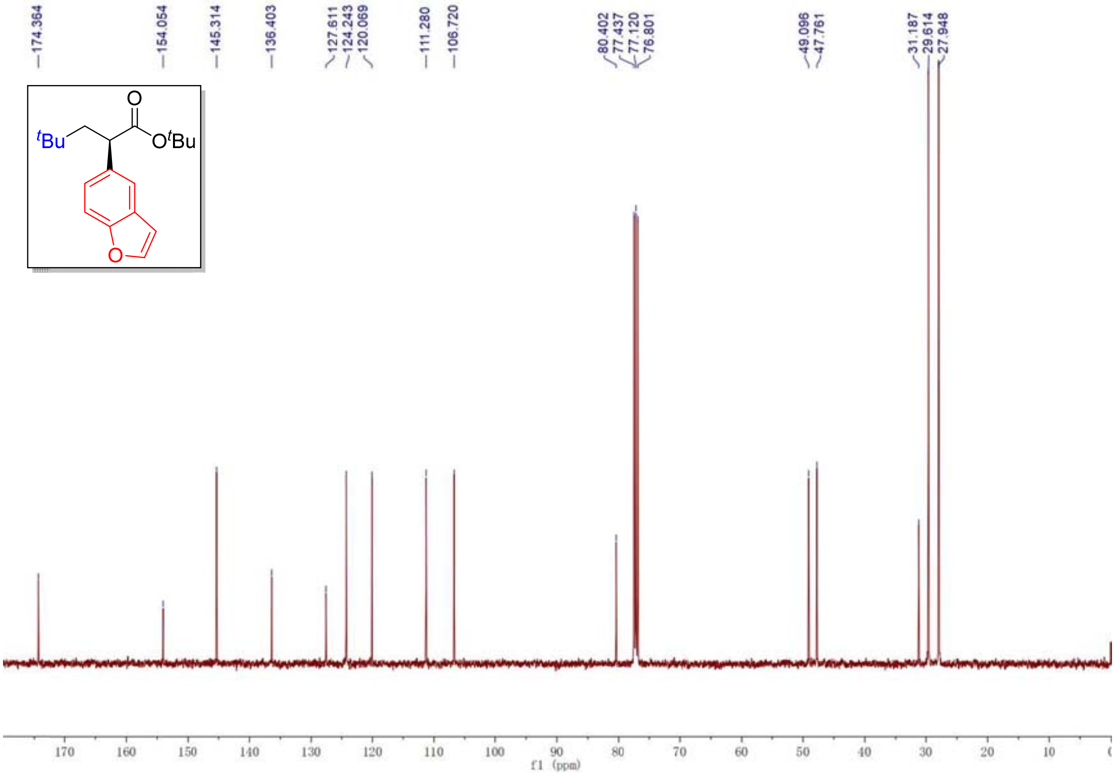

Supplementary Figure 25. ^{1}H (400 MHz) and ^{13}C {^{1}H} (101 MHz) NMR spectra of 4y in $CDCl_3$

(*R*)-*tert*-butyl 2-(benzo[b]thiophen-5-yl)-4,4-dimethylpentanoate (4z)

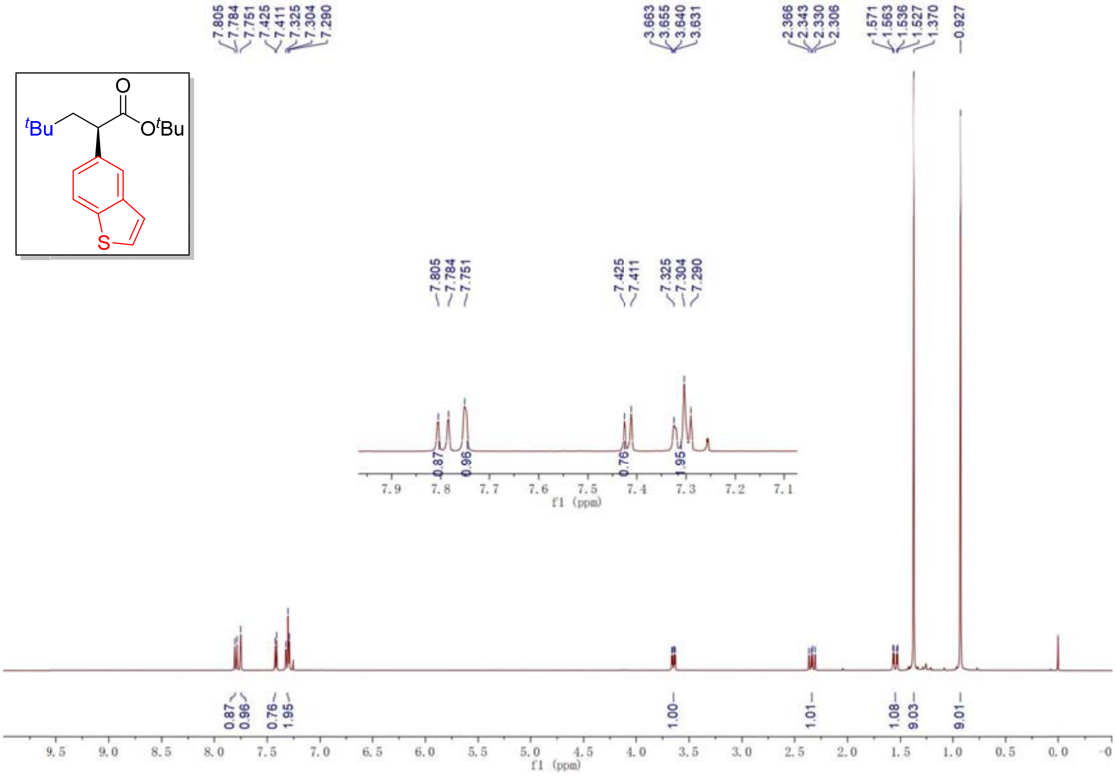

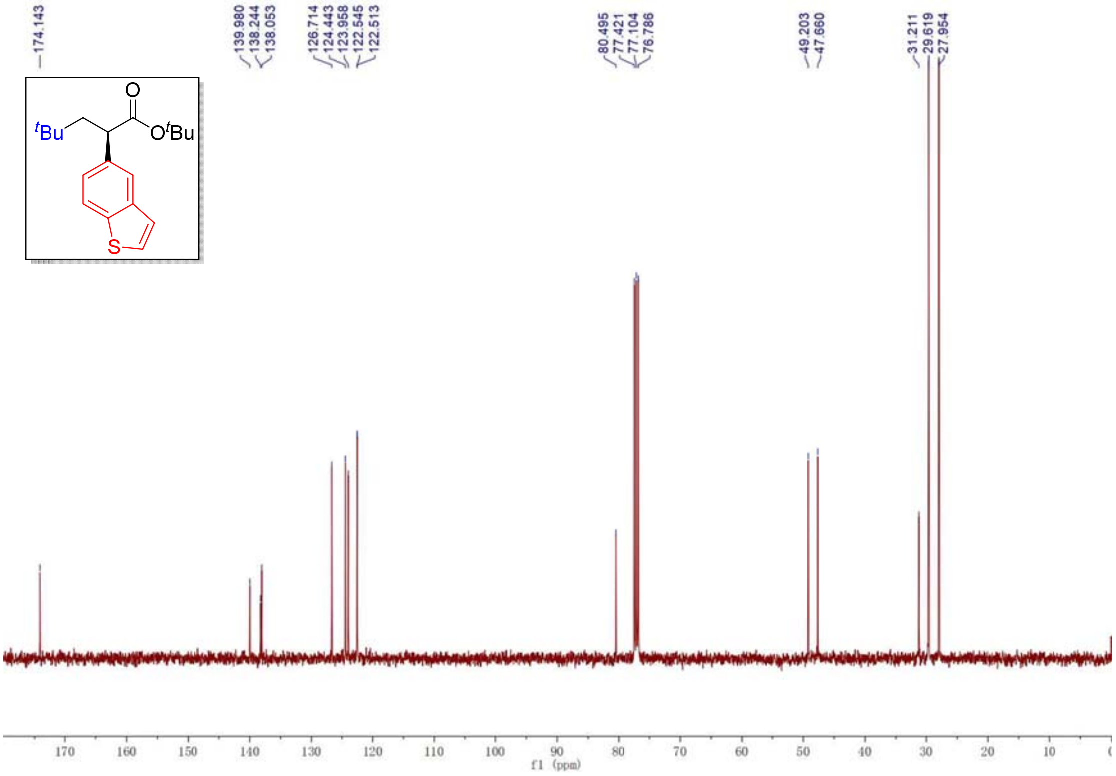

Supplementary Figure 26. **^{1}H (400 MHz) and ^{13}C {^{1}H} (101 MHz) NMR spectra of 4z in $CDCl_3$**

(*R*)-*tert*-butyl 4,4-dimethyl-2-(1-methyl-1*H*-indol-5-yl)pentanoate (4aa)

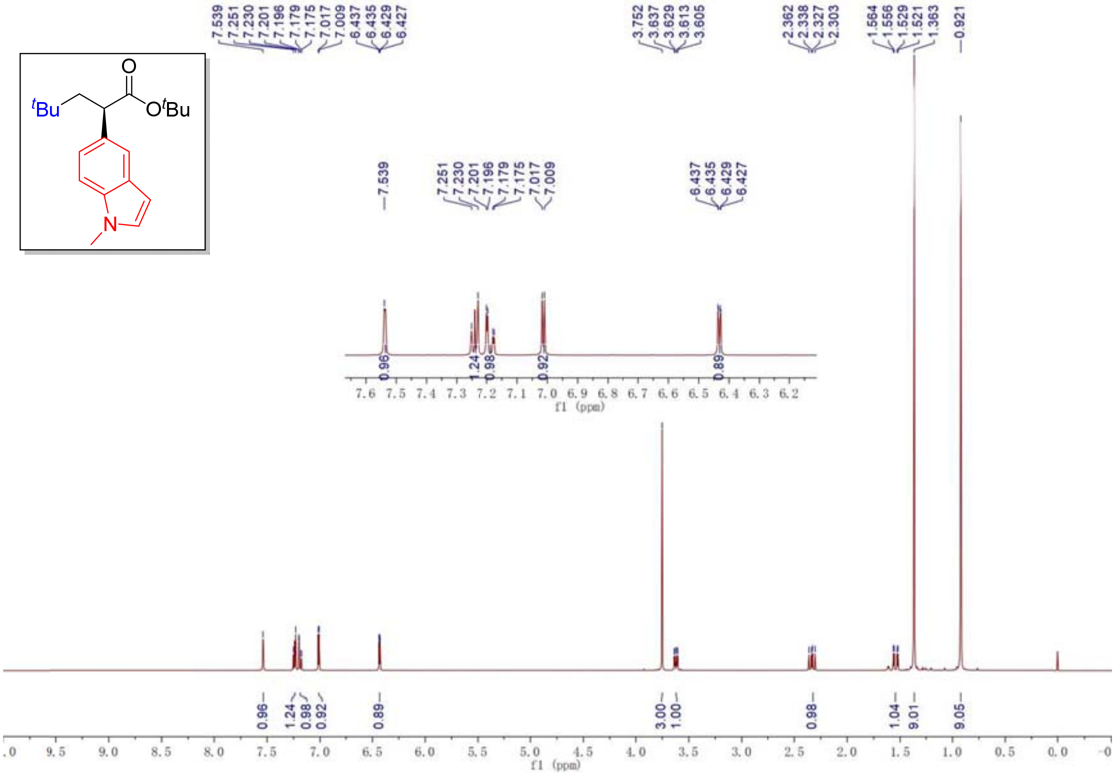

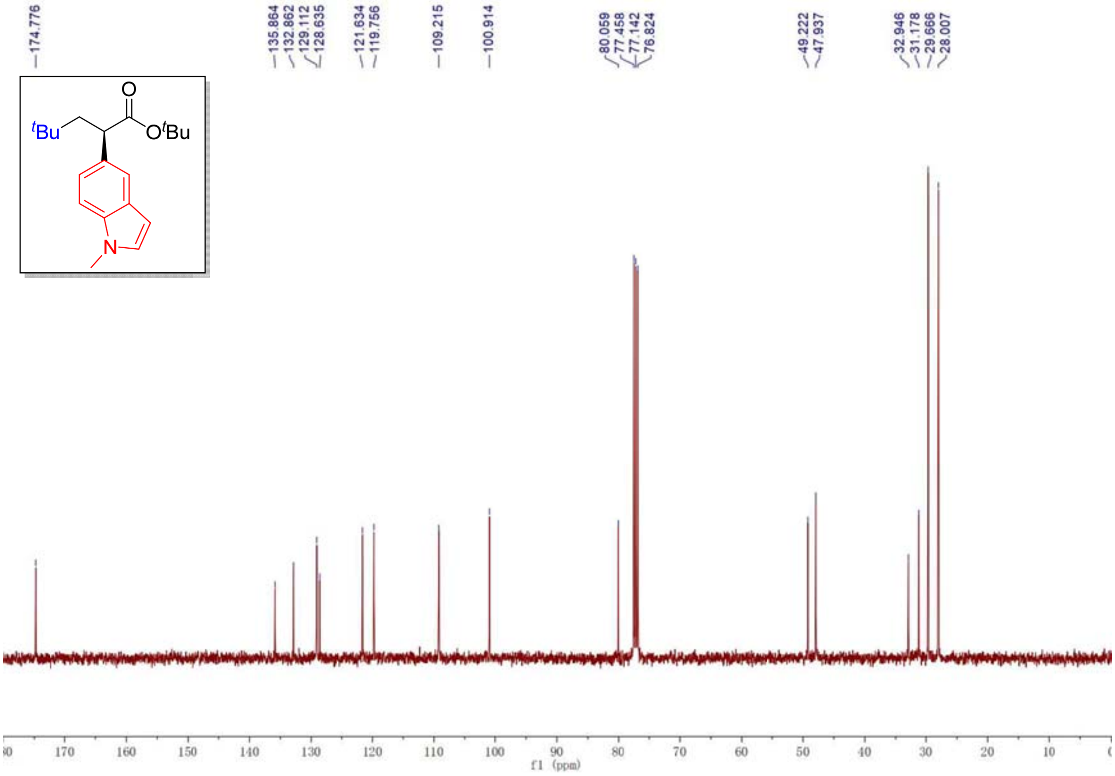

Supplementary Figure 27. ^{1}H **(400 MHz) and** ^{13}C **{**^{1}H**} (101 MHz) NMR spectra of 4aa in** $CDCl_3$

(*R*)-*tert*-butyl 4,4-dimethyl-2-(9-phenyl-9*H*-carbazol-3-yl)pentanoate (4ab)

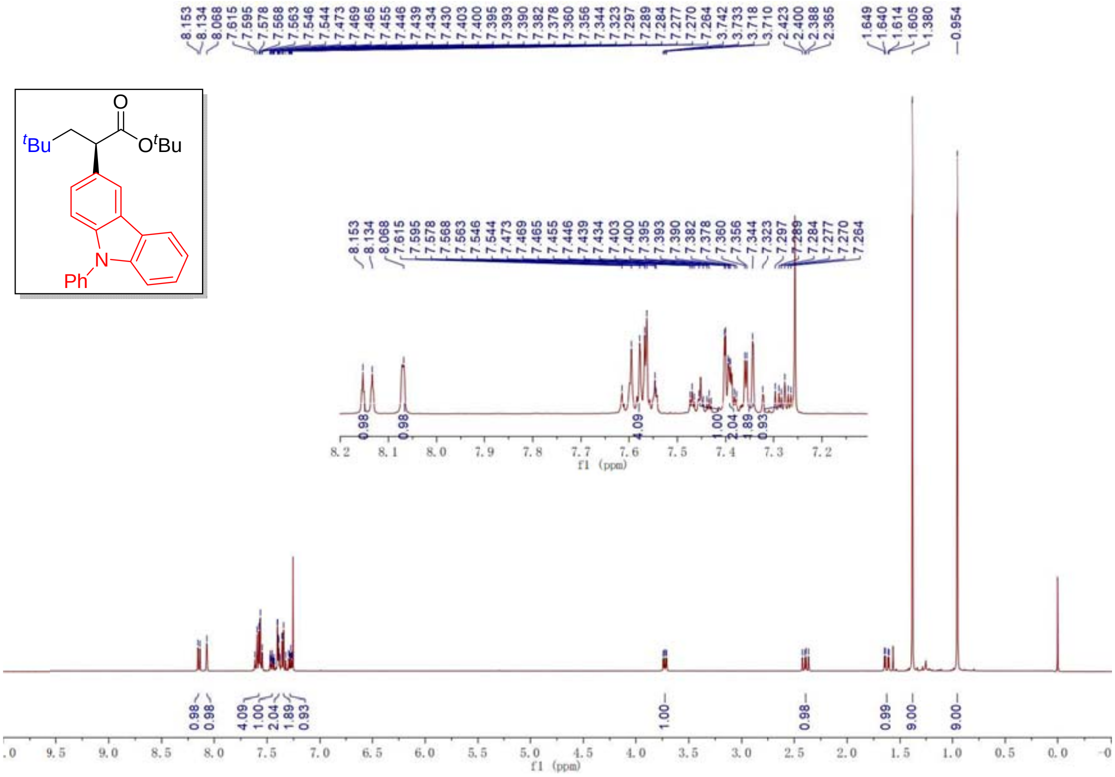

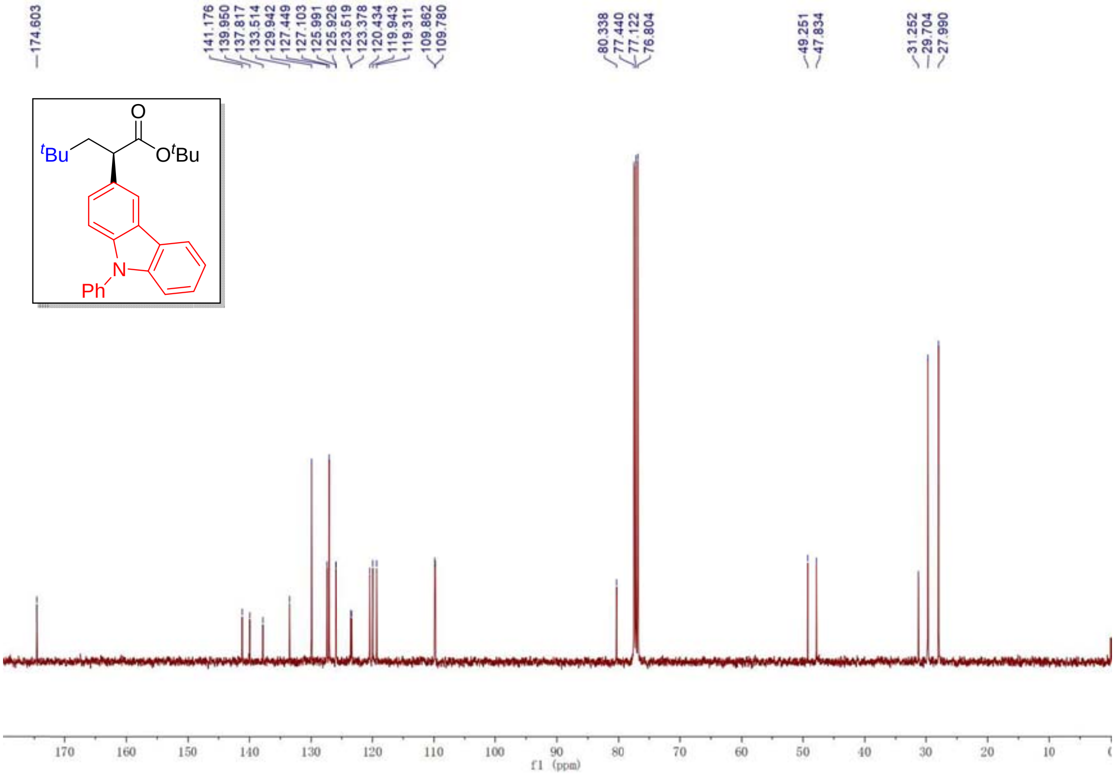

Supplementary Figure 28. ^{1}**H (400 MHz) and** ^{13}**C {**^{1}**H} (101 MHz) NMR spectra of 4ab in** $CDCl_3$

(*R*)-*tert*-butyl 2-(4-((((*S*)-2-(4-isobutylphenyl)propanoyl)oxy)methyl)phenyl)-4,4-dimethylpentanoate (4ac)

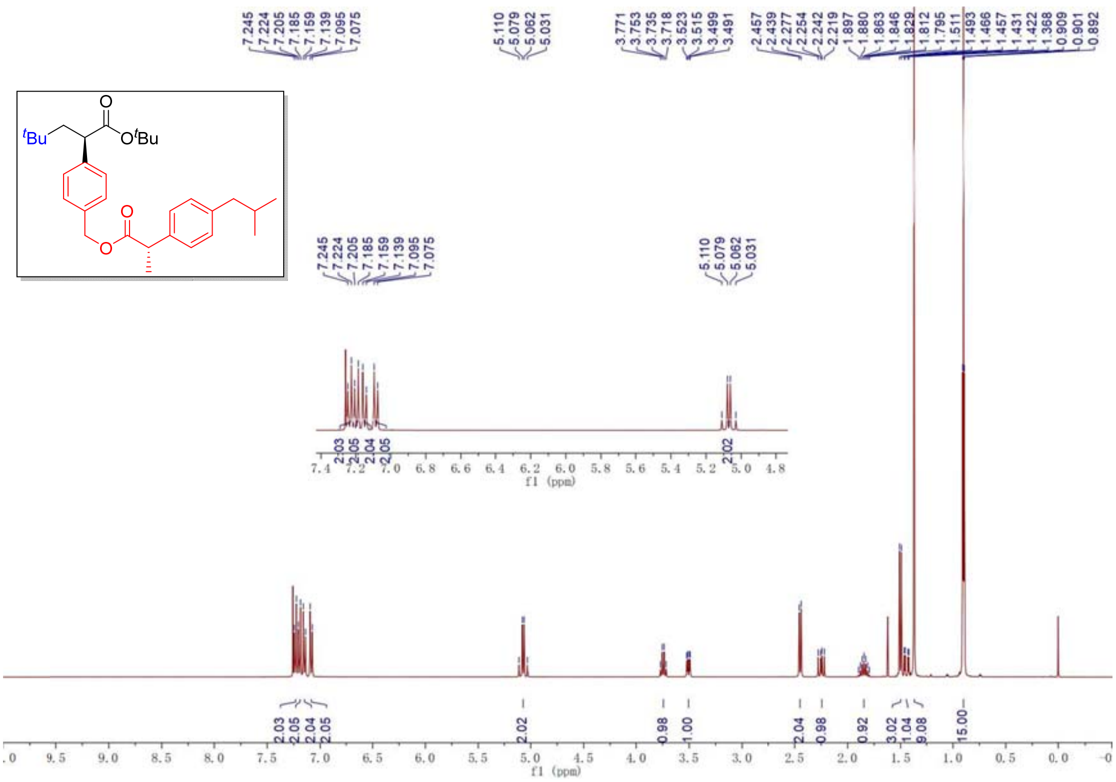

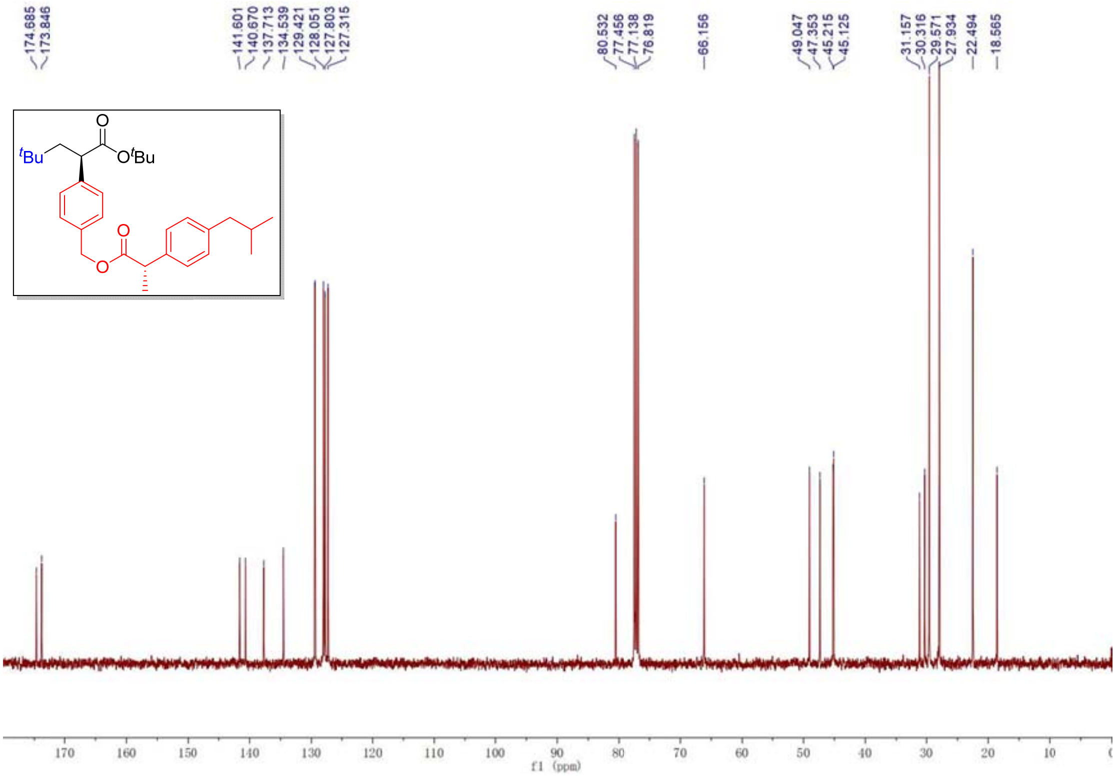

Supplementary Figure 29. ^{1}**H (400 MHz) and** ^{13}**C {**^{1}**H} (101 MHz) NMR spectra of 4ac in** $CDCl_3$

(*R*)-(*E*)-3,7-dimethylocta-2,6-dien-1-yl 4-(1-(tert-butoxy)-4,4-dimethyl-1-oxopentan-2-yl)benzoate (4ad)

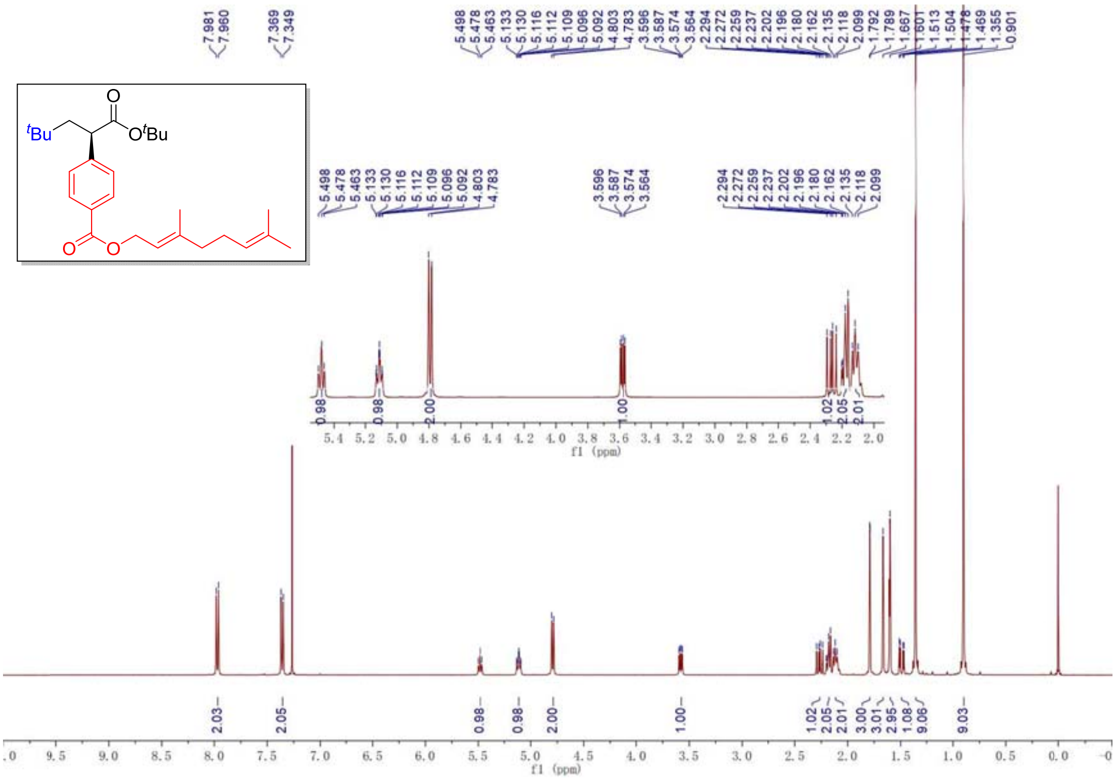

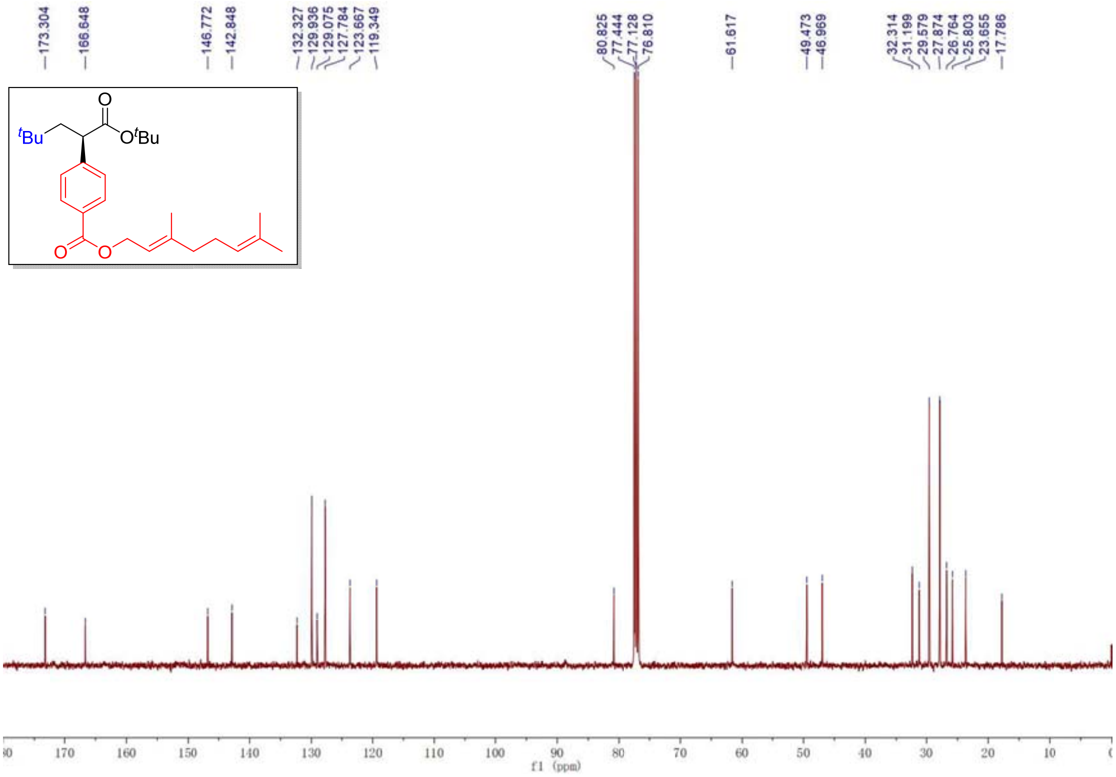

Supplementary Figure 30. ^{1}H (400 MHz) and ^{13}C {^{1}H} (101 MHz) NMR spectra of 4ad in $CDCl_3$

(*R*)-*tert*-butyl 2-(4-acetylphenyl)-4,4-dimethylhexanoate (5a)

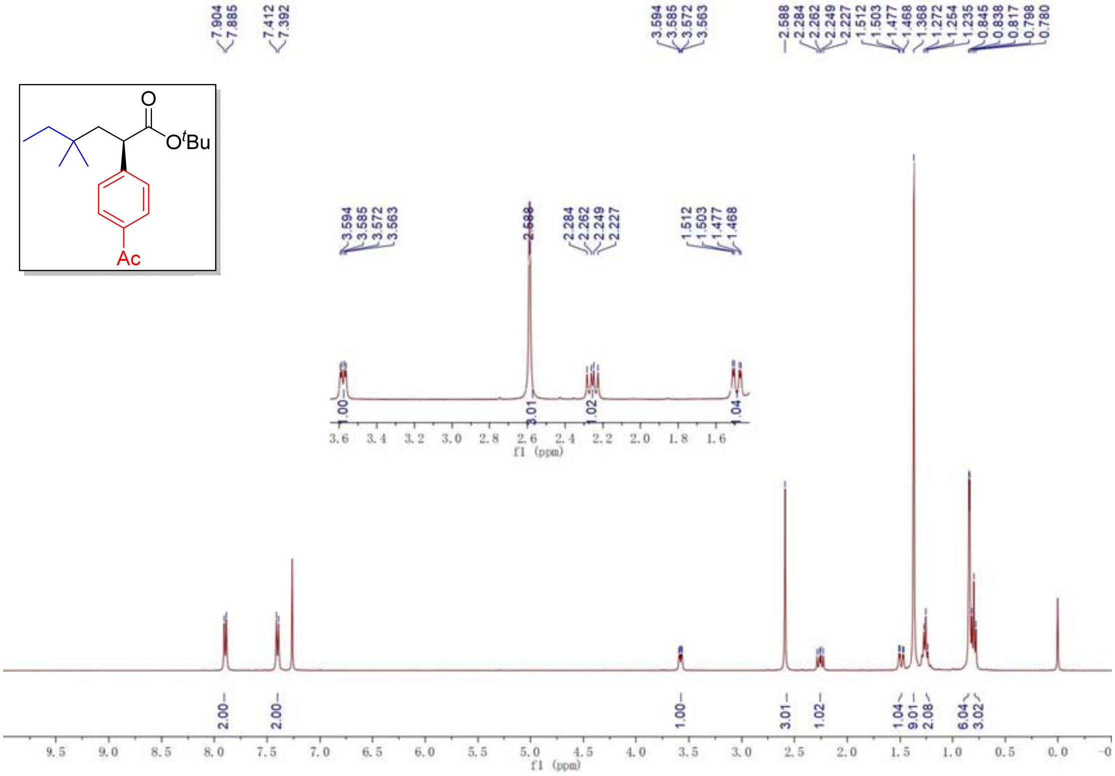

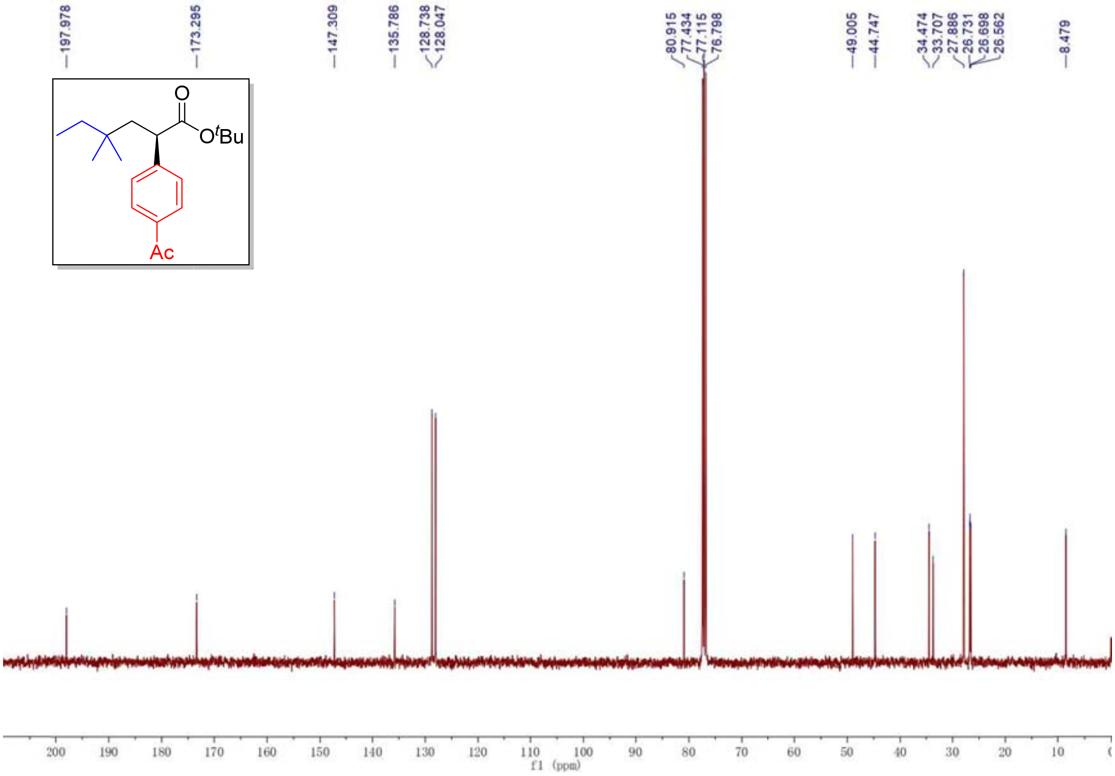

Supplementary Figure 31. ^{1}**H (400 MHz) and** ^{13}**C {**^{1}**H} (101 MHz) NMR spectra of 5a in** $CDCl_3$

(*R*)-*tert*-butyl 2-(4-acetylphenyl)-6-bromo-4,4-dimethylhexanoate (5b)

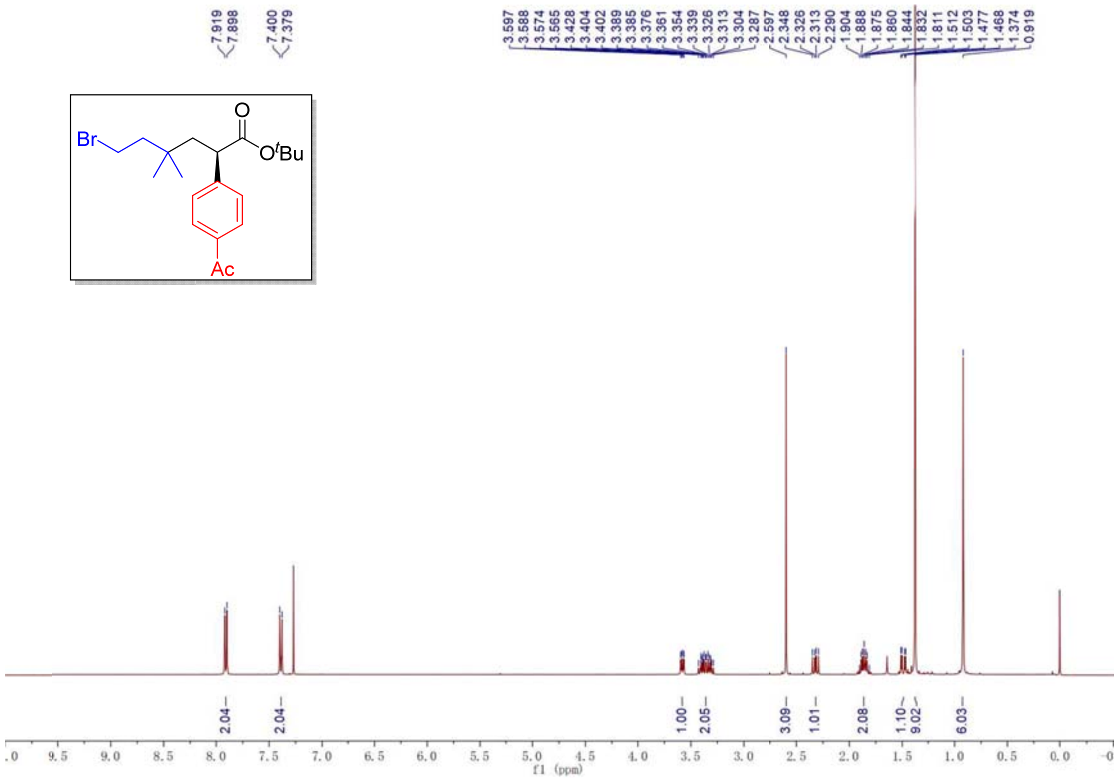

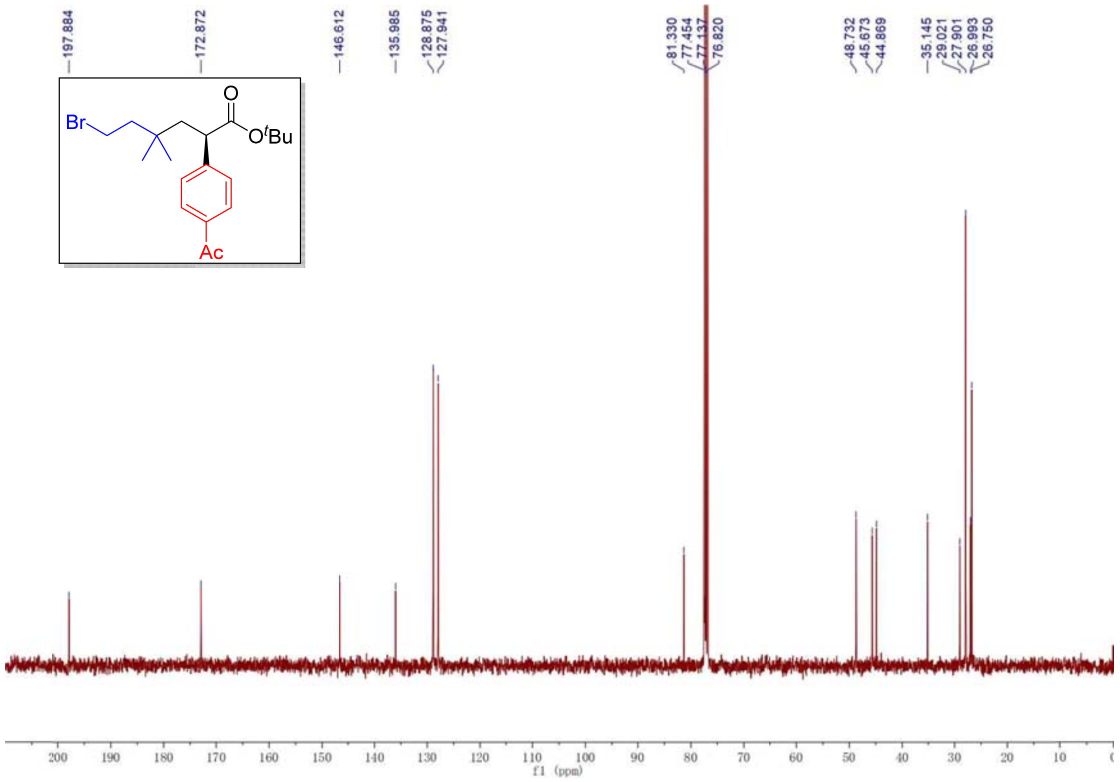

Supplementary Figure 32. ^{1}**H (400 MHz) and** ^{13}**C {**^{1}**H} (101 MHz) NMR spectra of 5b in** $CDCl_3$

(*R*)-*tert*-butyl 2-(4-acetylphenyl)-4,4-dimethyl-6-phenylhexanoate (5c)

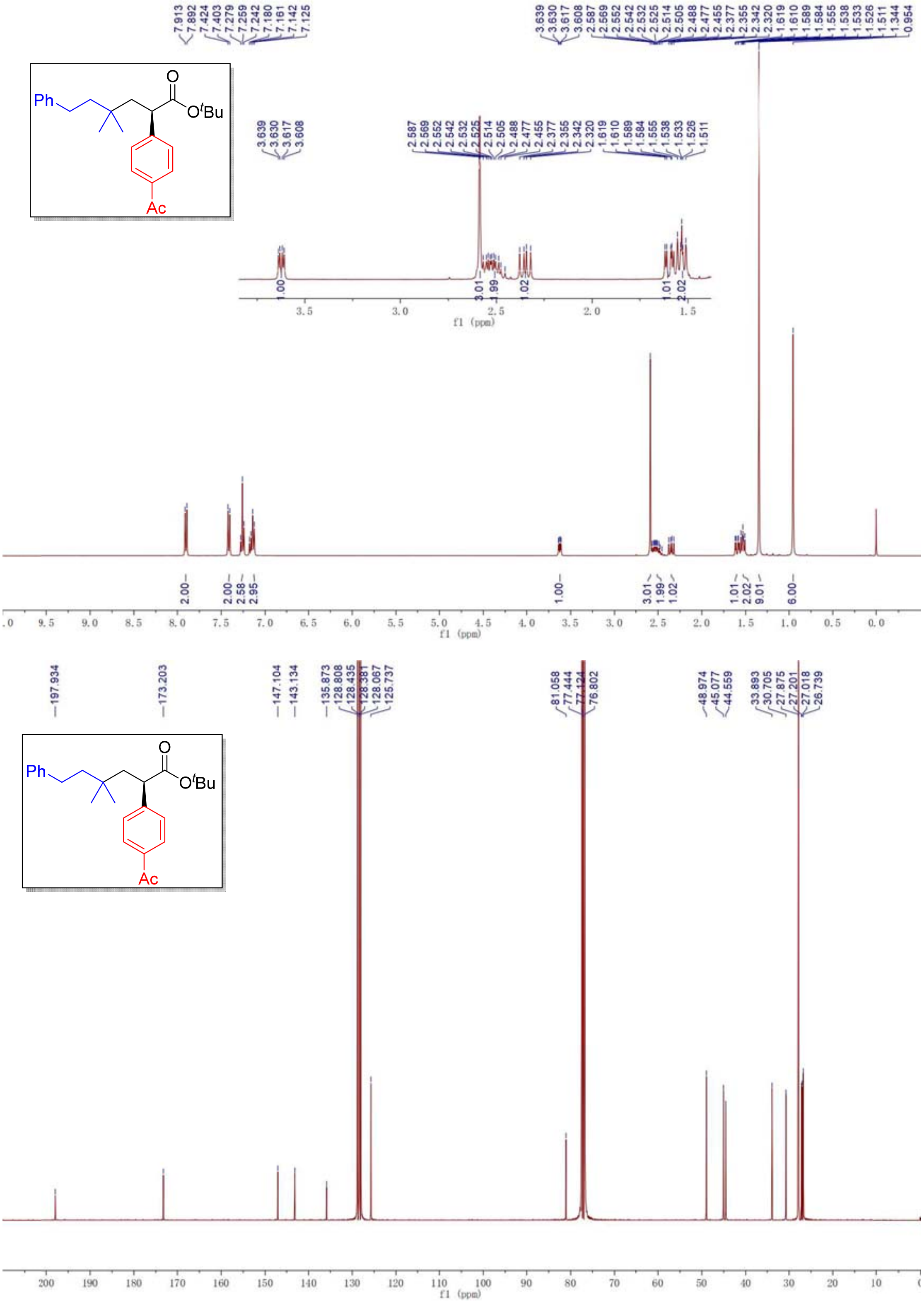

Supplementary Figure 33. ^{1}**H (400 MHz) and** ^{13}**C {**^{1}**H} (101 MHz) NMR spectra of 5c in** $CDCl_3$

(*R*)-5-(4-acetylphenyl)-6-(*tert*-butoxy)-3,3-dimethyl-6-oxohexyl benzoate (5d)

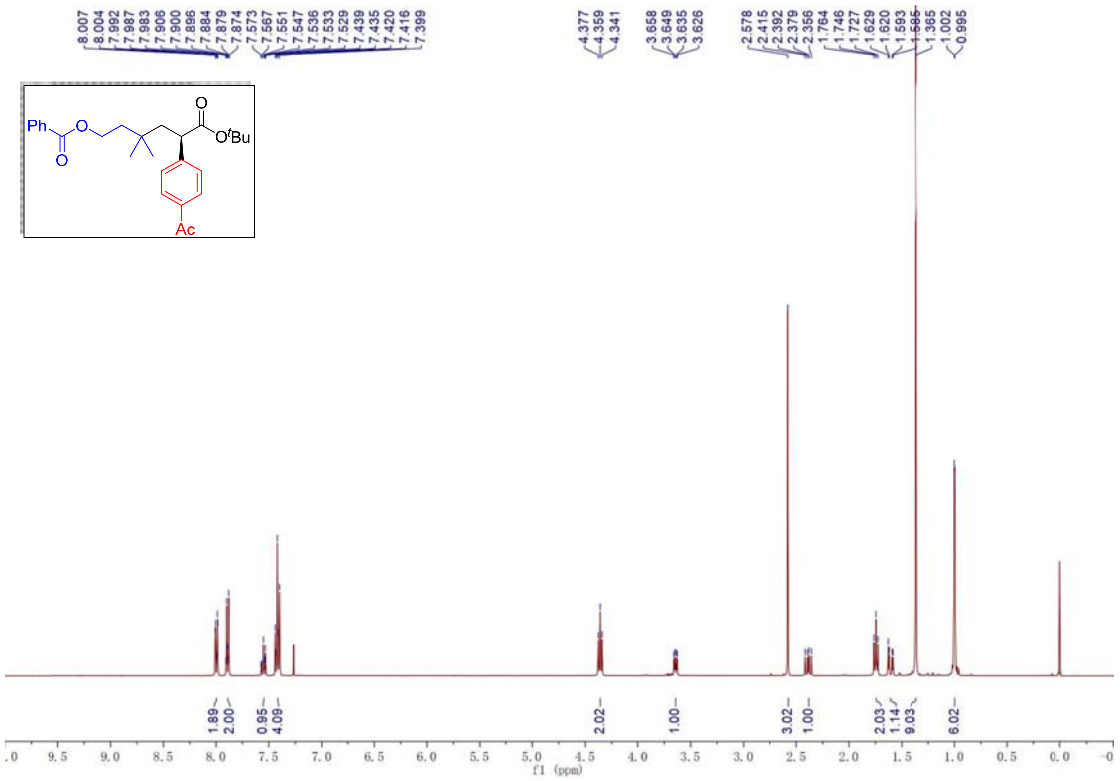

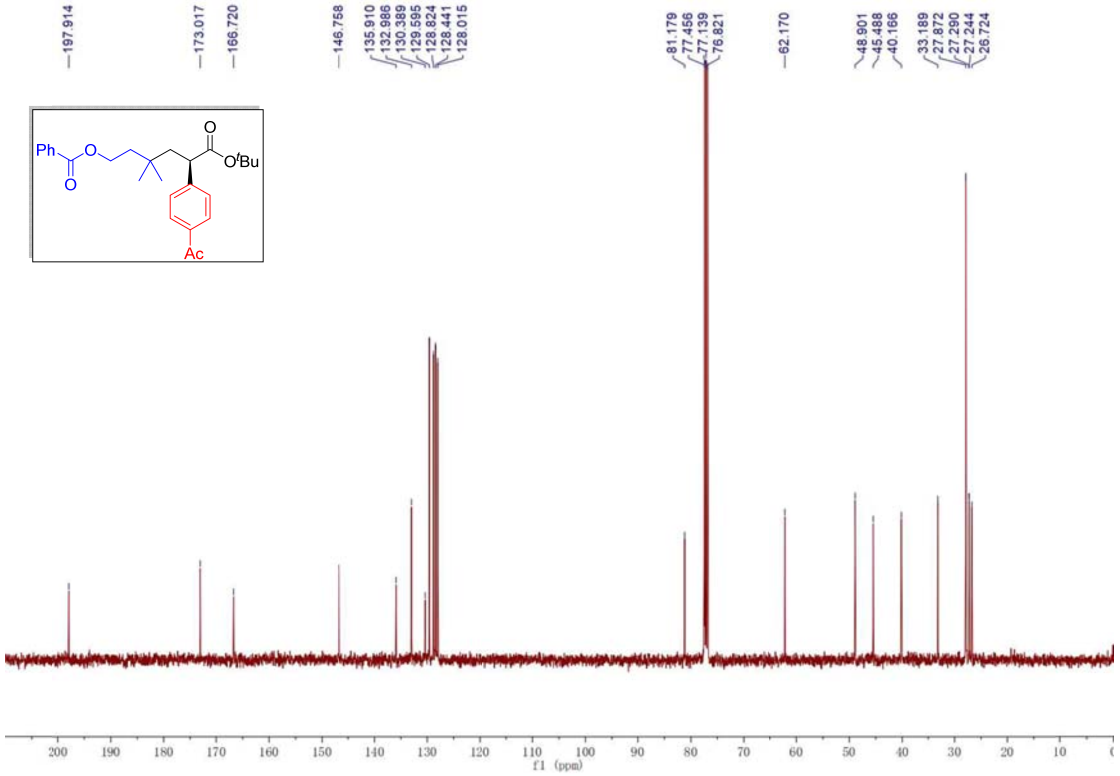

Supplementary Figure 34. ^{1}H (400 MHz) and ^{13}C {^{1}H} (101 MHz) NMR spectra of 5d in $CDCl_3$

(*R*)-5-(4-acetylphenyl)-6-(*tert*-butoxy)-3,3-dimethyl-6-oxohexyl 4-cyanobenzoate (5e)

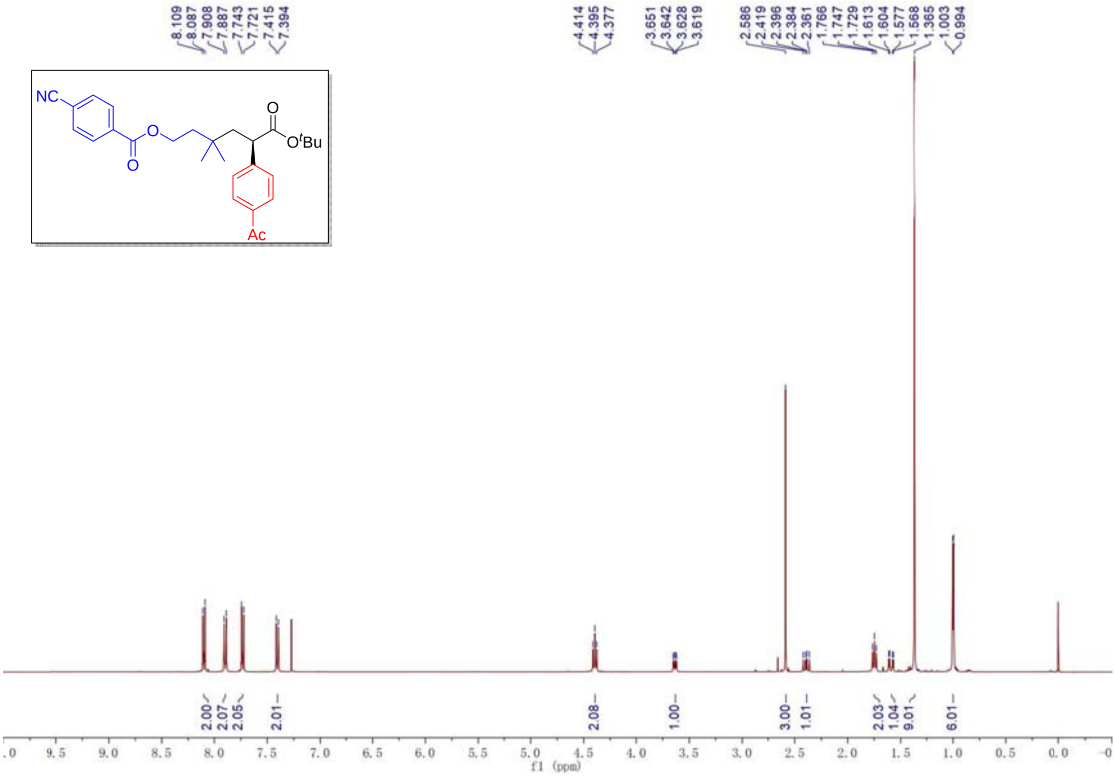

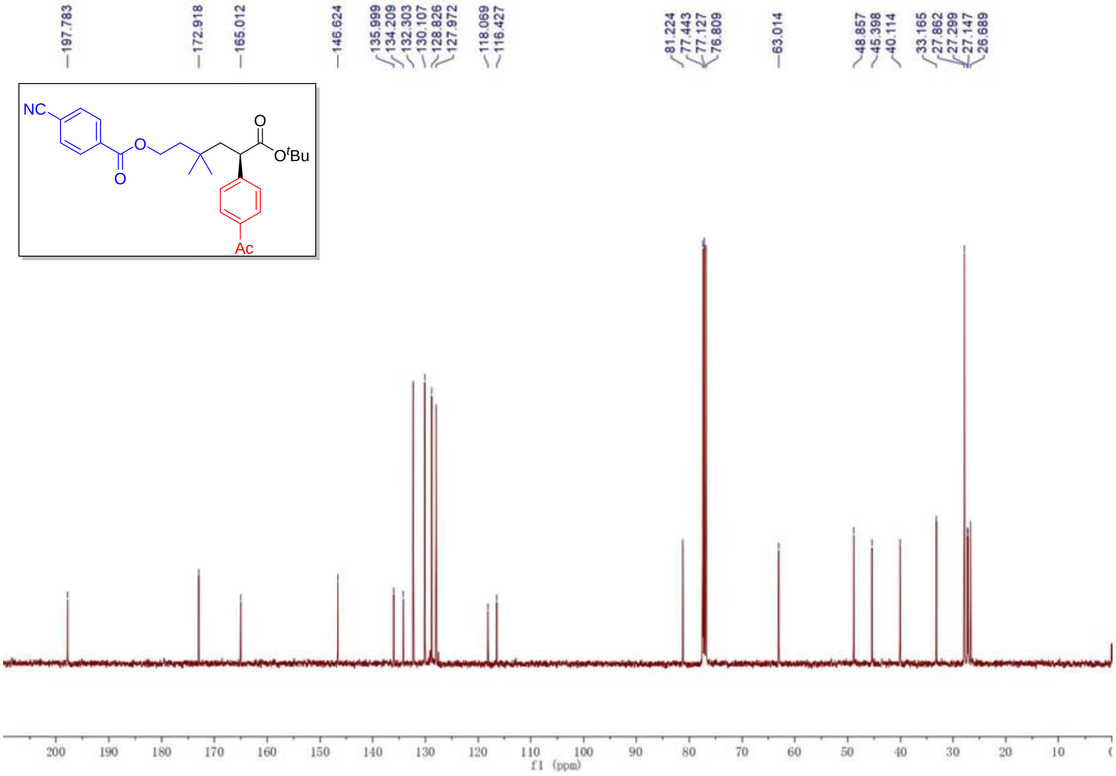

Supplementary Figure 35. ^{1}H (400 MHz) and ^{13}C {^{1}H} (101 MHz) NMR spectra of 5e in $CDCl_3$

(*R*)-5-(4-acetylphenyl)-6-(*tert*-butoxy)-3,3-dimethyl-6-oxohexyl isonicotinate (5f)

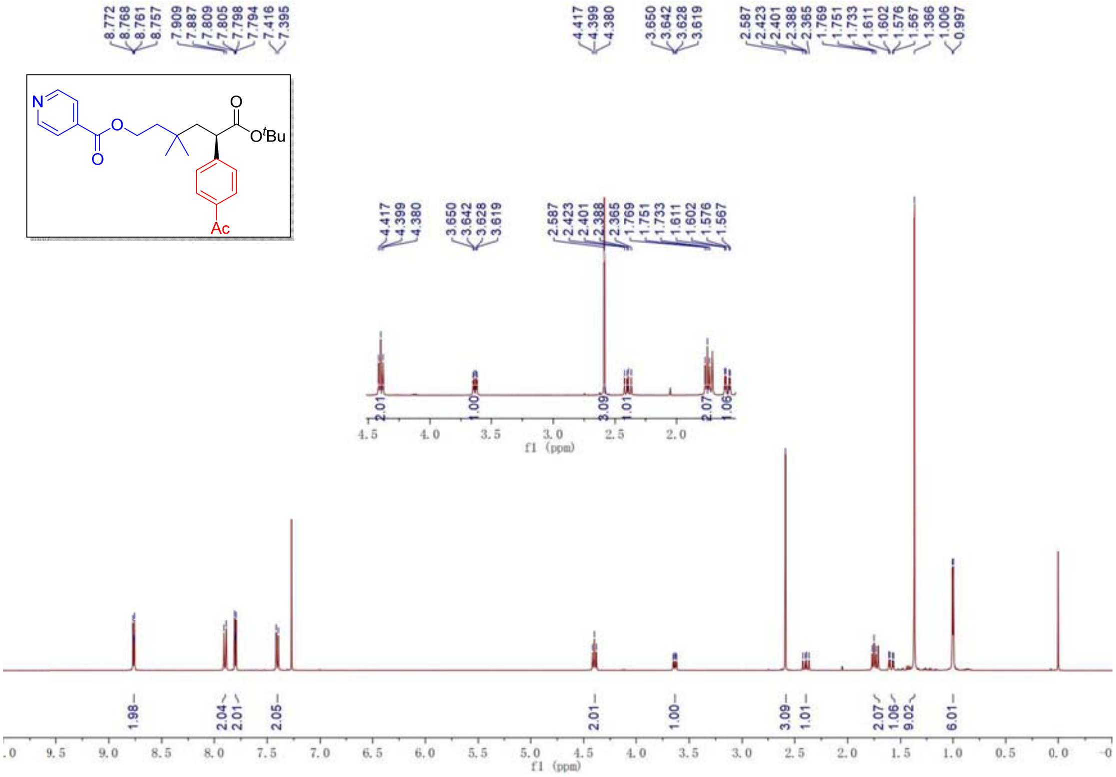

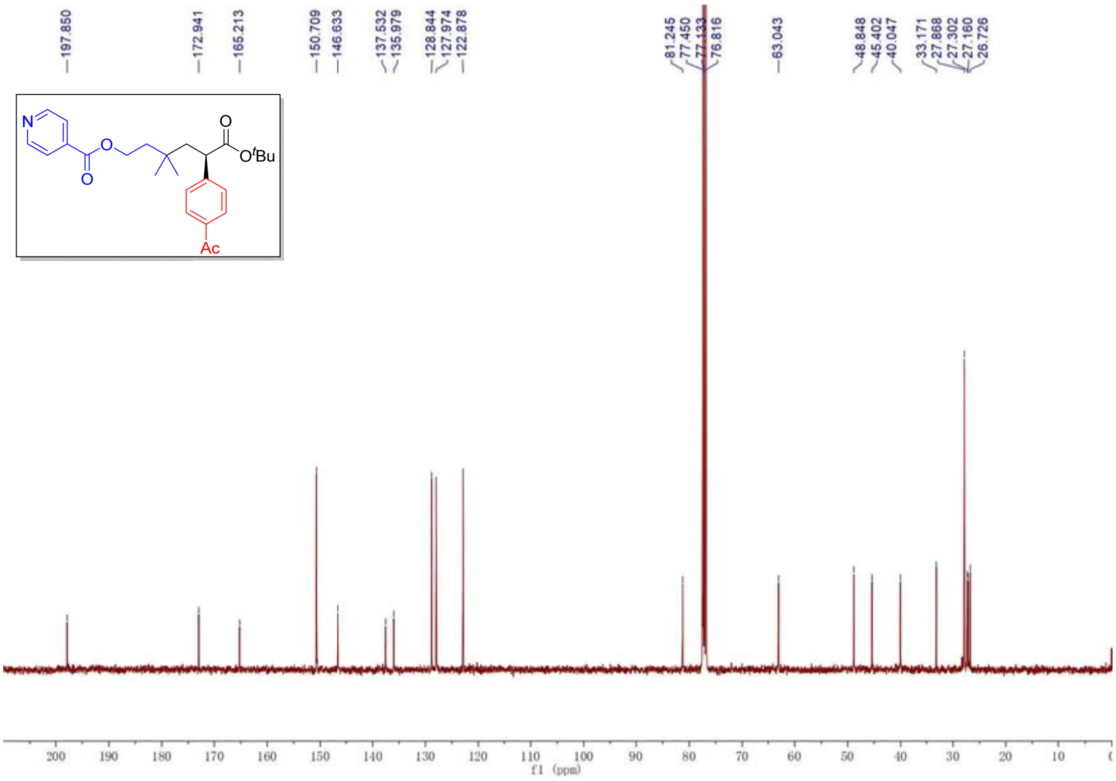

Supplementary Figure 36. ^{1}H (400 MHz) and ^{13}C {^{1}H} (101 MHz) NMR spectra of 5f in $CDCl_3$

(*R*)-5-(4-acetylphenyl)-6-(*tert*-butoxy)-3,3-dimethyl-6-oxohexyl thiophene-2-carboxylate (5g)

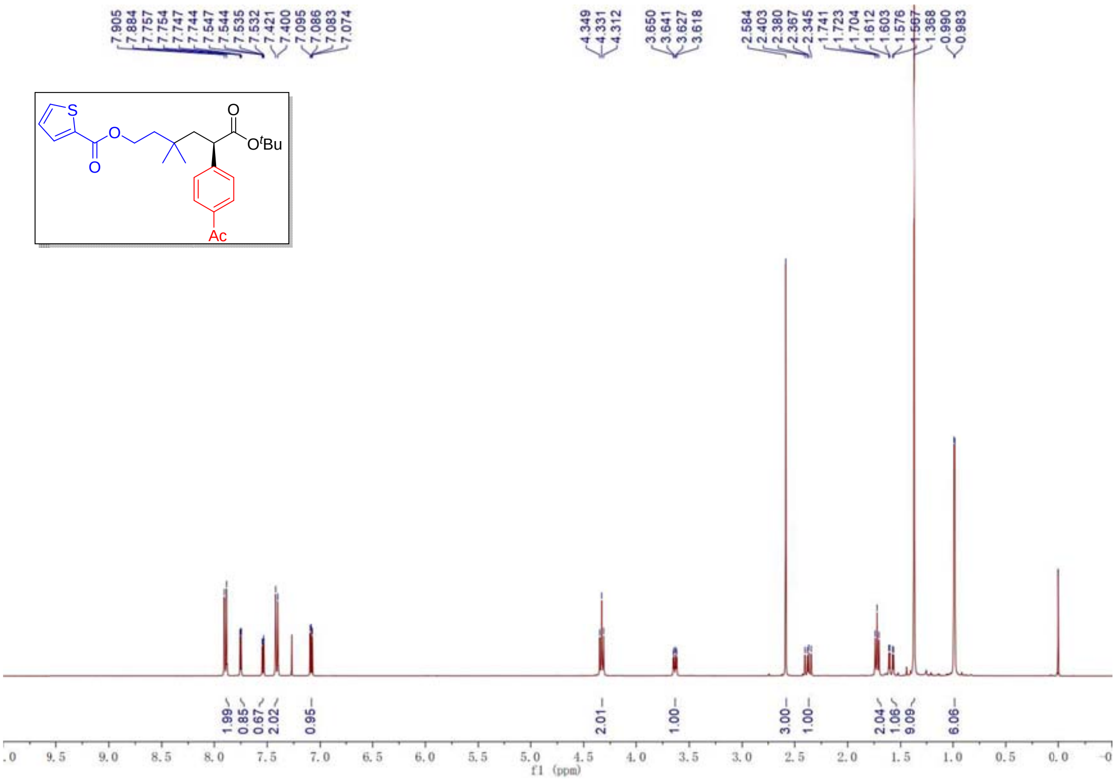

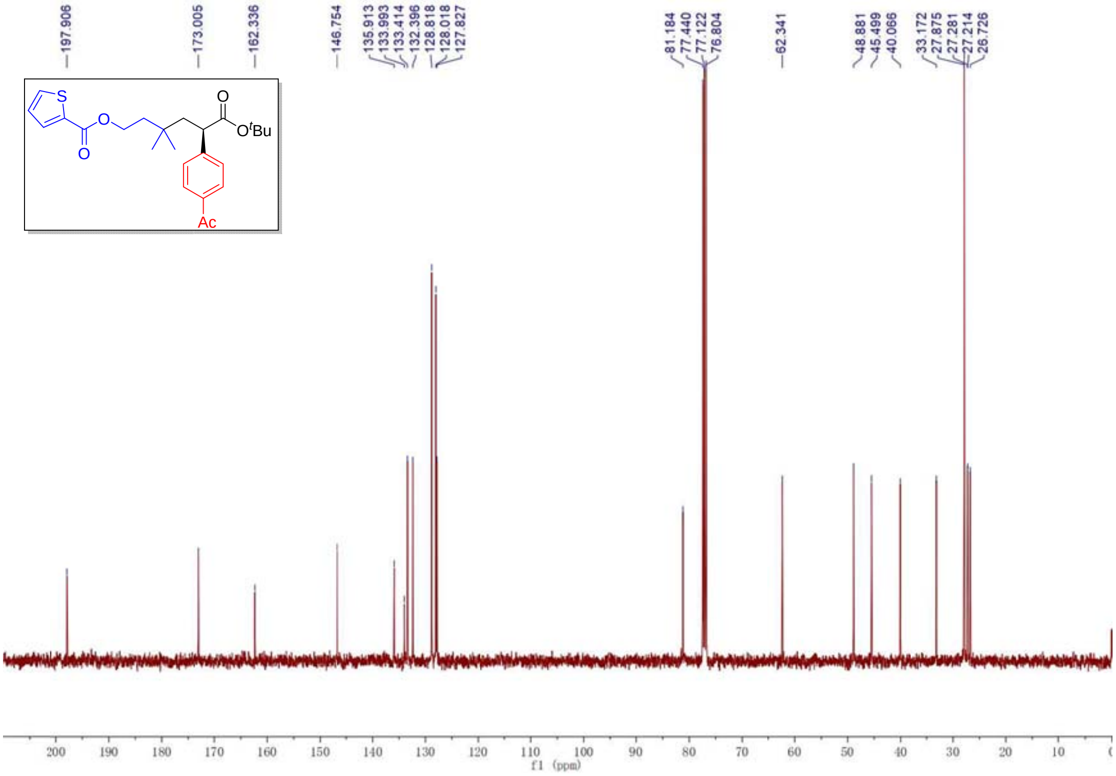

Supplementary Figure 37. ^{1}H (400 MHz) and ^{13}C {^{1}H} (101 MHz) NMR spectra of 5g in $CDCl_3$

(*R*)-5-(4-acetylphenyl)-6-(*tert*-butoxy)-3,3-dimethyl-6-oxohexyl furan-2-carboxylate (5h)

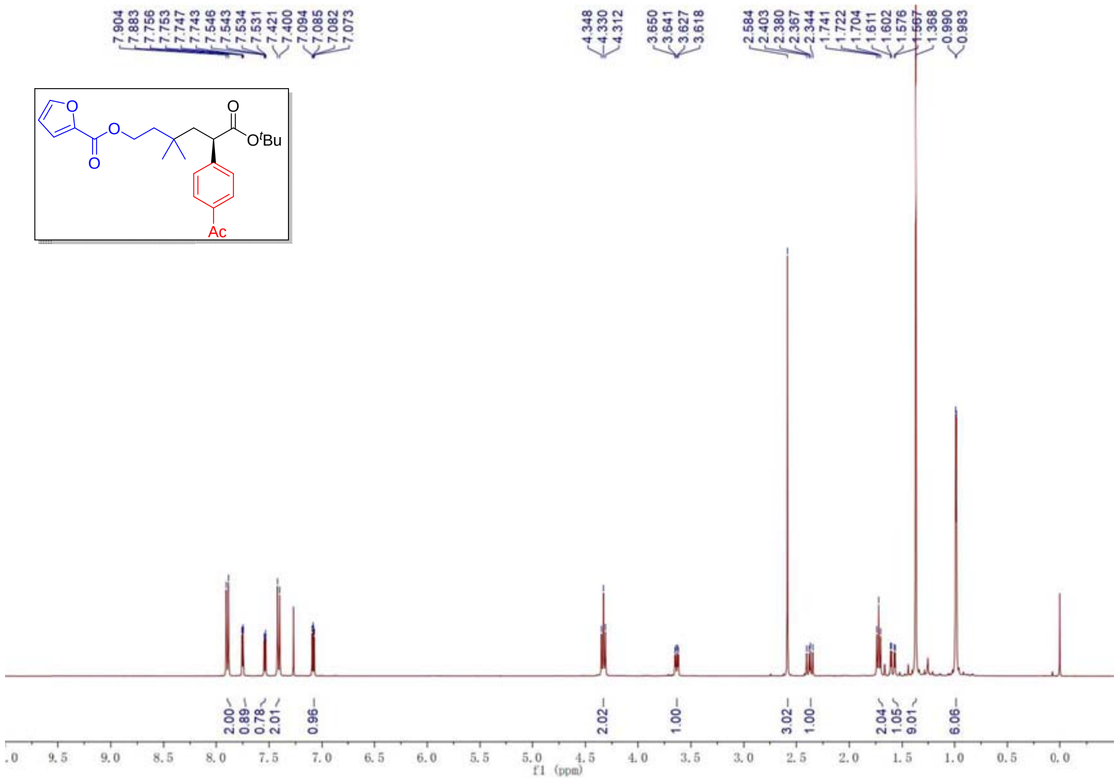

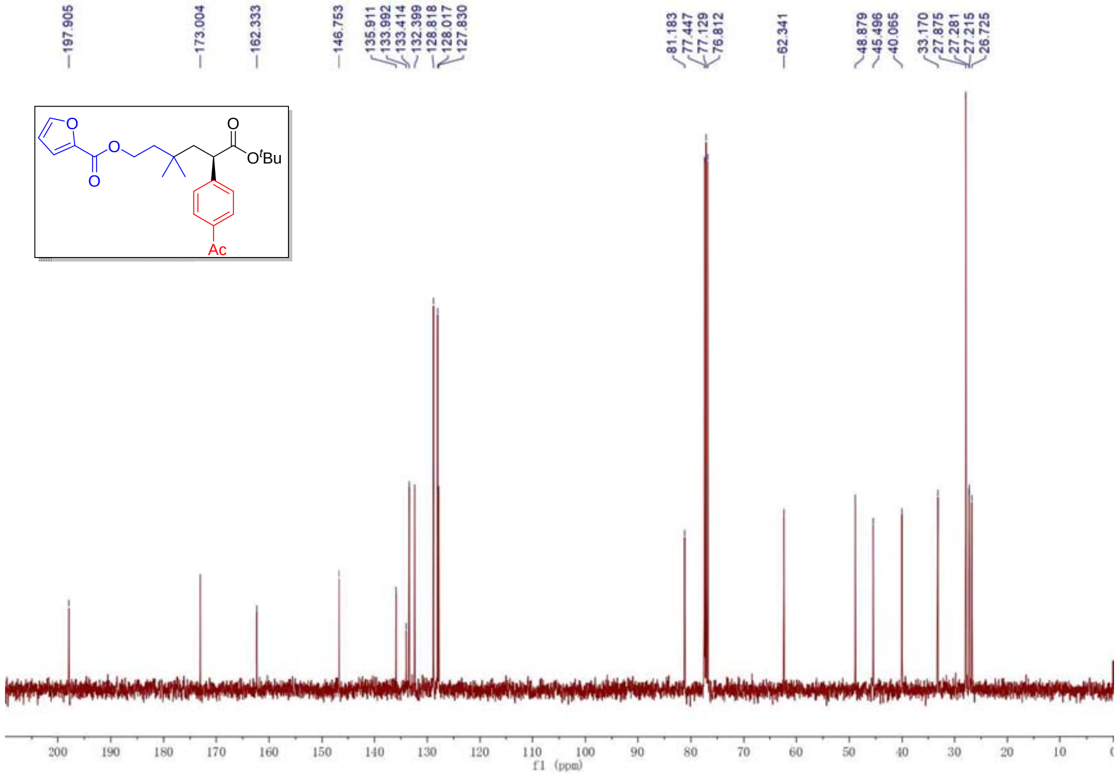

Supplementary Figure 38. ^{1}H (400 MHz) and ^{13}C {^{1}H} (101 MHz) NMR spectra of 5h in $CDCl_3$

(*R*)-*tert*-butyl 2-(4-acetylphenyl)-6-(2-(1,3-dioxoisoindolin-2-yl)acetoxy)-4,4-dimethylhexanoate (5i)

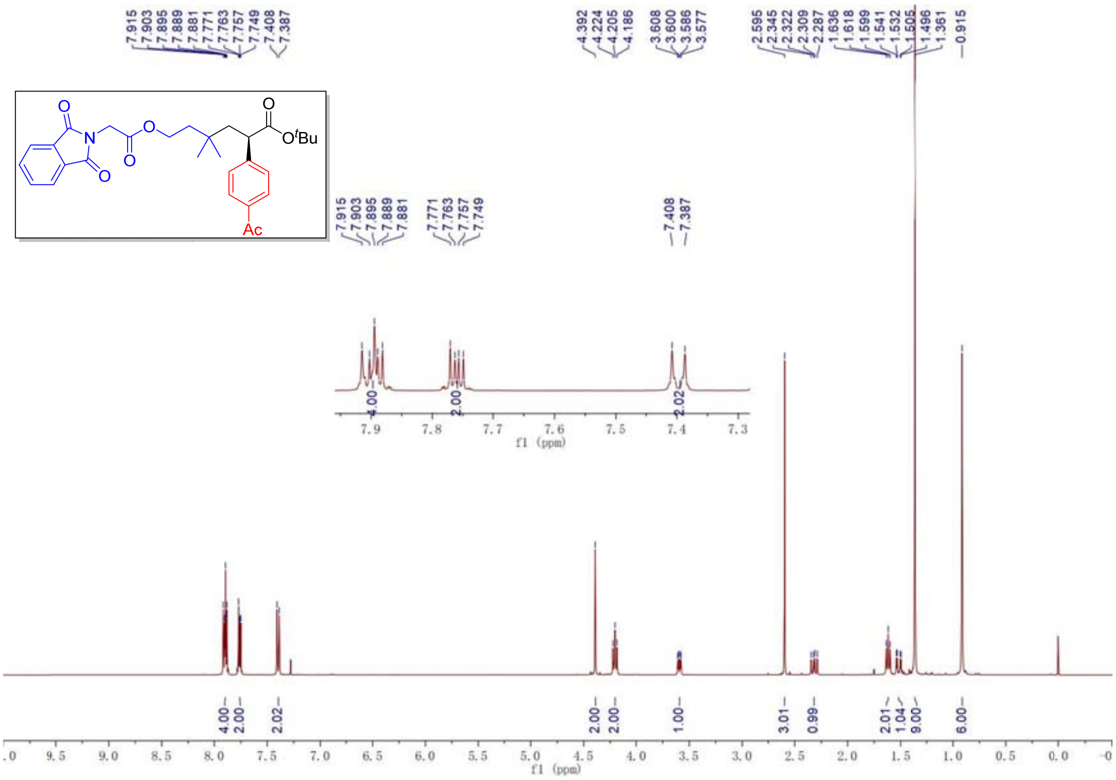

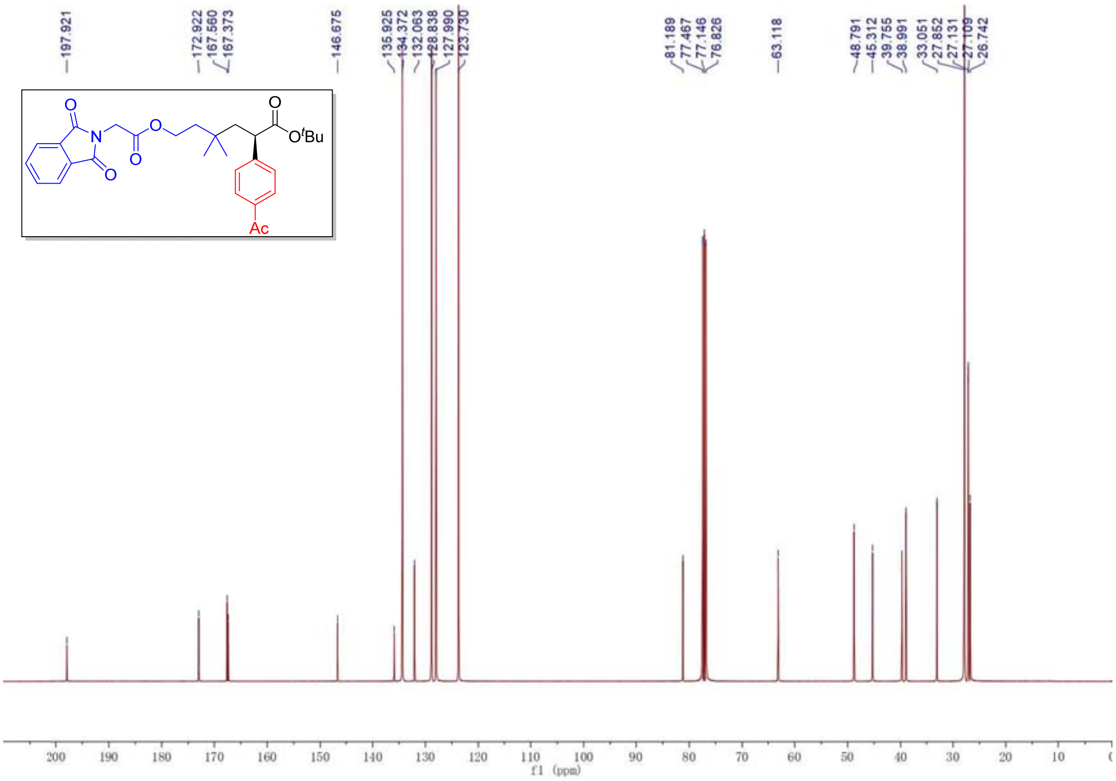

Supplementary Figure 39. ^{1}**H (400 MHz) and** ^{13}**C {**^{1}**H} (101 MHz) NMR spectra of 5i in** $CDCl_3$

(*R*)-*tert*-butyl 2-(4-acetylphenyl)-4,4-dimethyl-6-((4-oxo-4-phenylbutanoyl)oxy)hexanoate (5j)

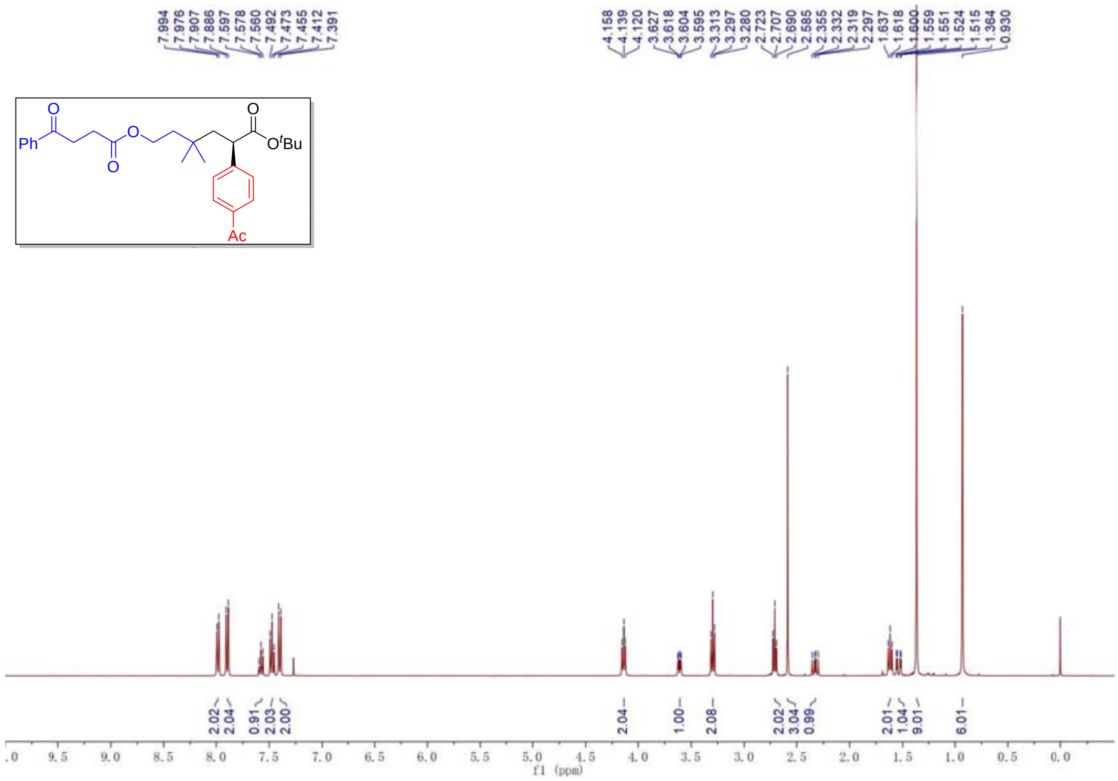

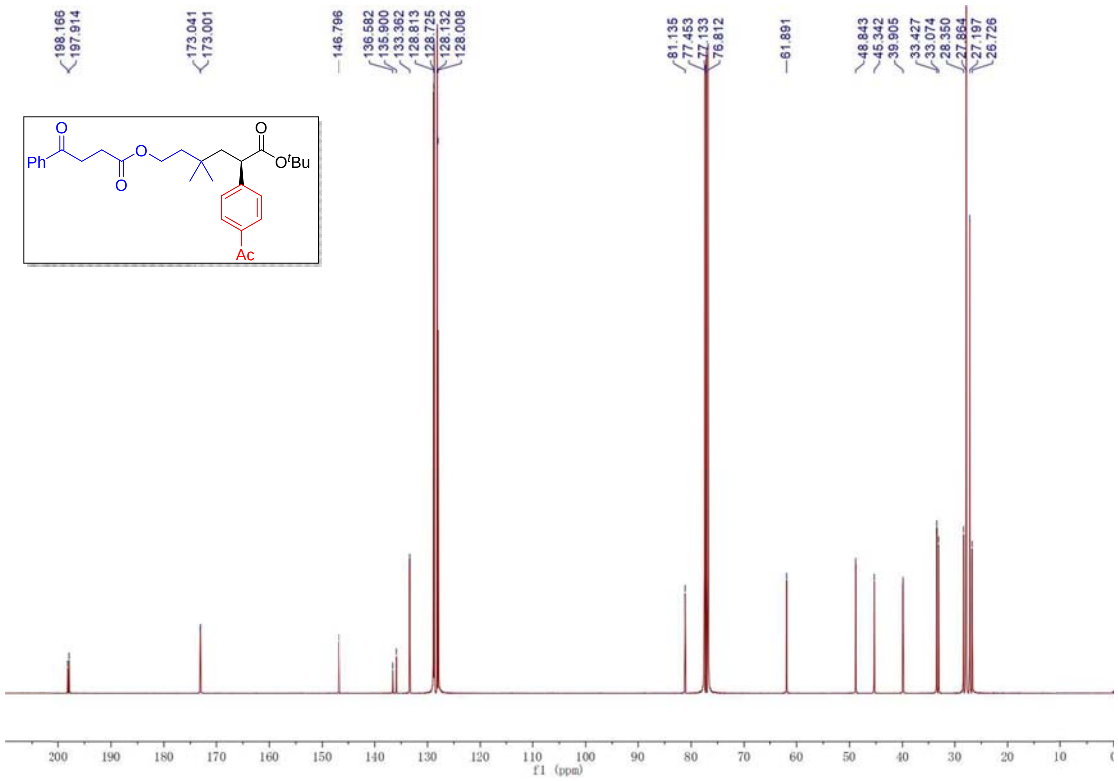

Supplementary Figure 40. ^{1}**H (400 MHz) and** ^{13}**C {**^{1}**H} (101 MHz) NMR spectra of 5j in CDCl**$_3$

(*R*)-*tert*-butyl 2-(4-acetylphenyl)-6-((3,3-diphenylpropanoyl)oxy)-4,4-dimethylhexanoate (5k)

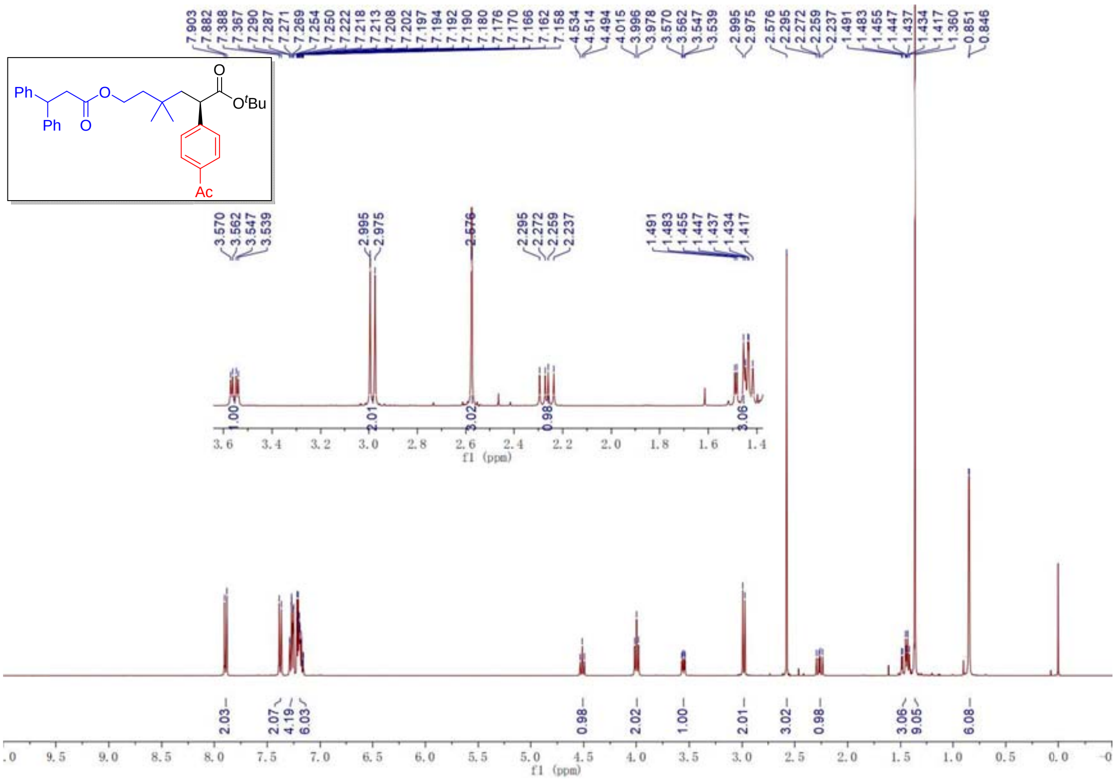

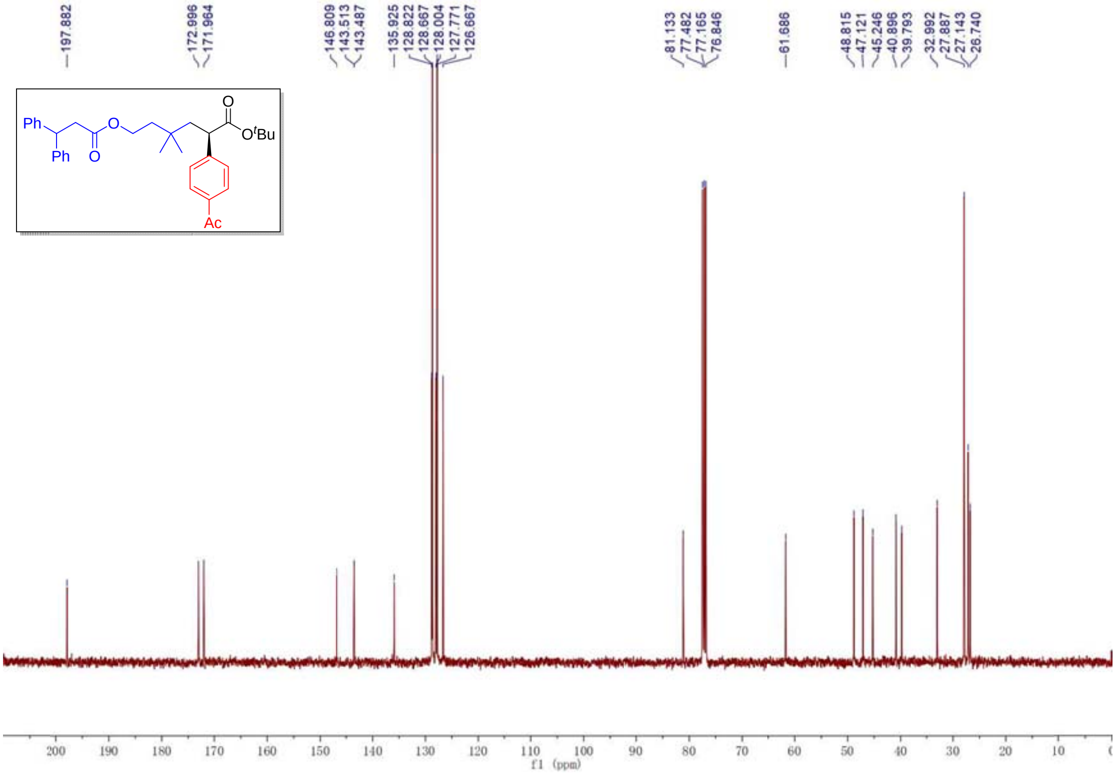

Supplementary Figure 41. ^{1}H (400 MHz) and ^{13}C {^{1}H} (101 MHz) NMR spectra of 5k in $CDCl_3$

(*R*)-*tert*-butyl 2-(4-acetylphenyl)-4-ethyl-4-methylhexanoate (5l)

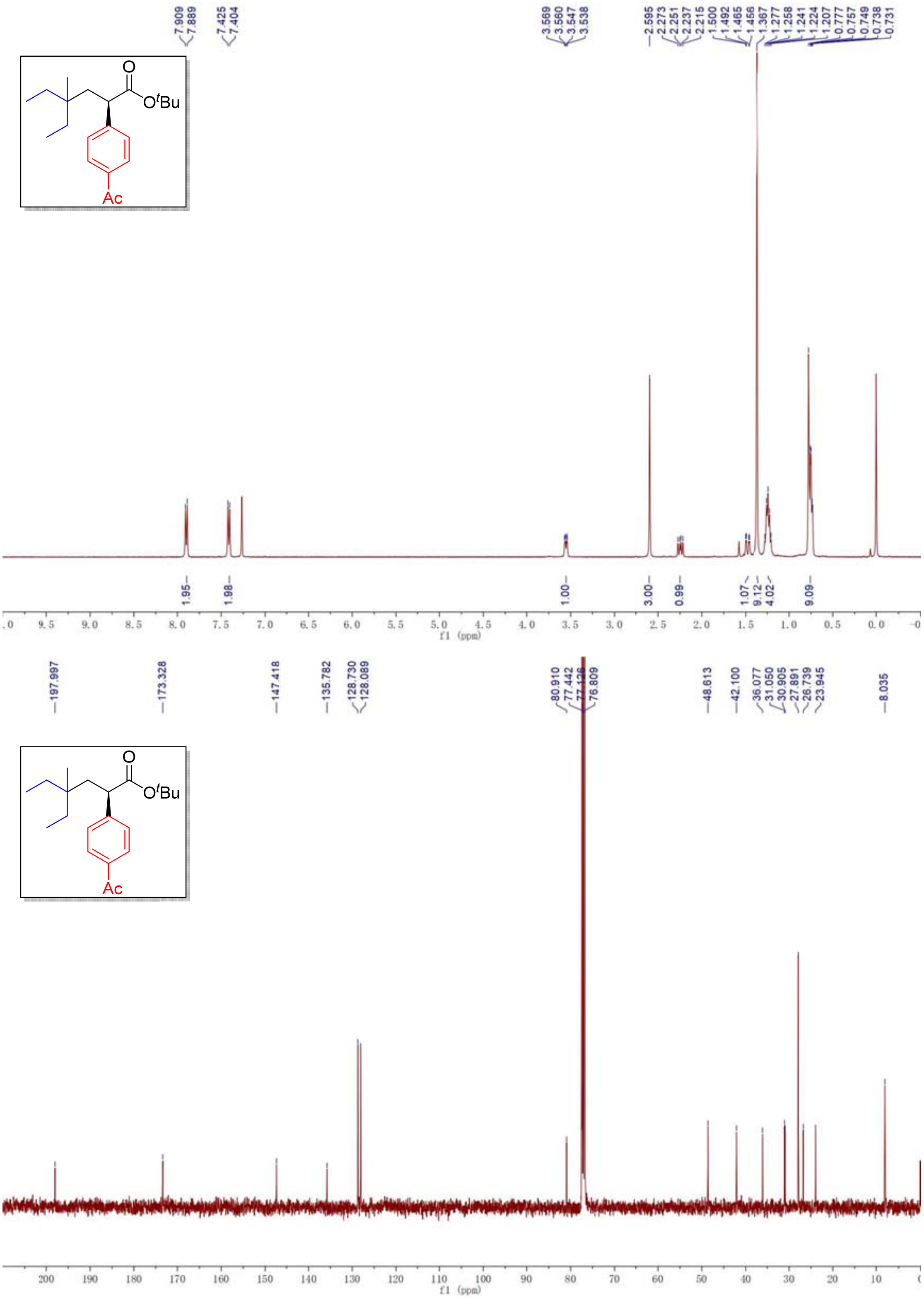

Supplementary Figure 42. ^{1}**H (400 MHz) and** ^{13}**C {**^{1}**H} (101 MHz) NMR spectra of 5l in** $CDCl_3$

(***R***)**-*****tert*****-butyl 2-(4-acetylphenyl)octanoate (6a)**

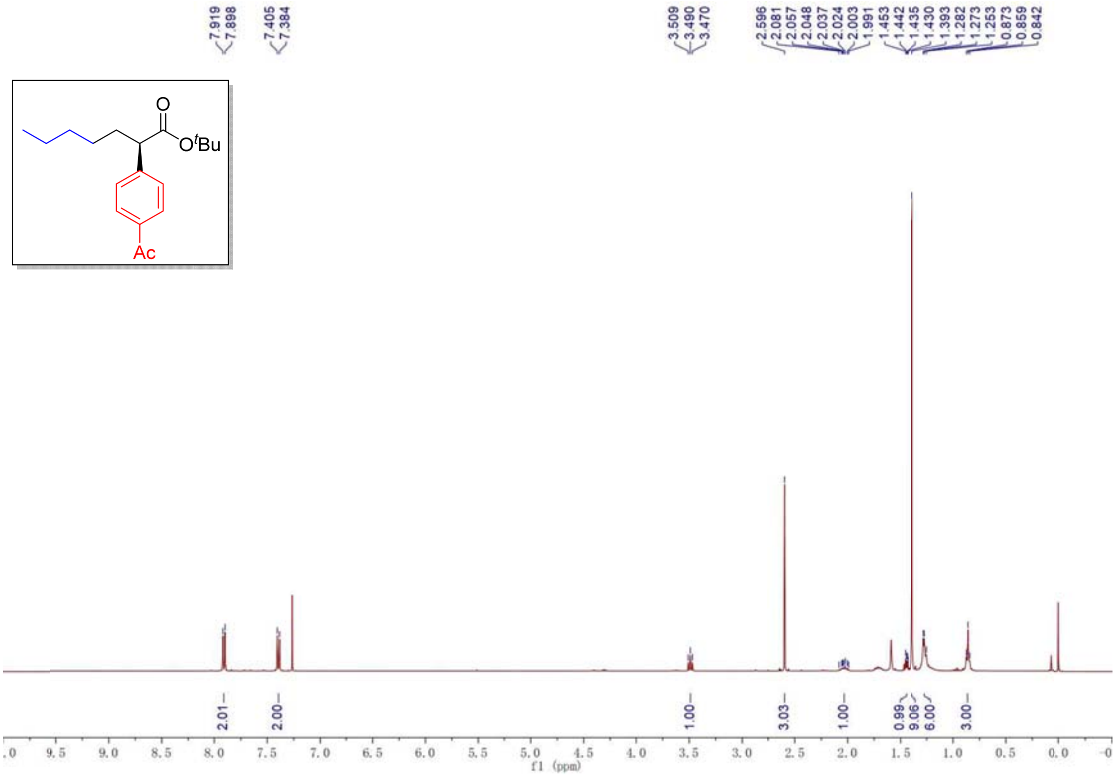

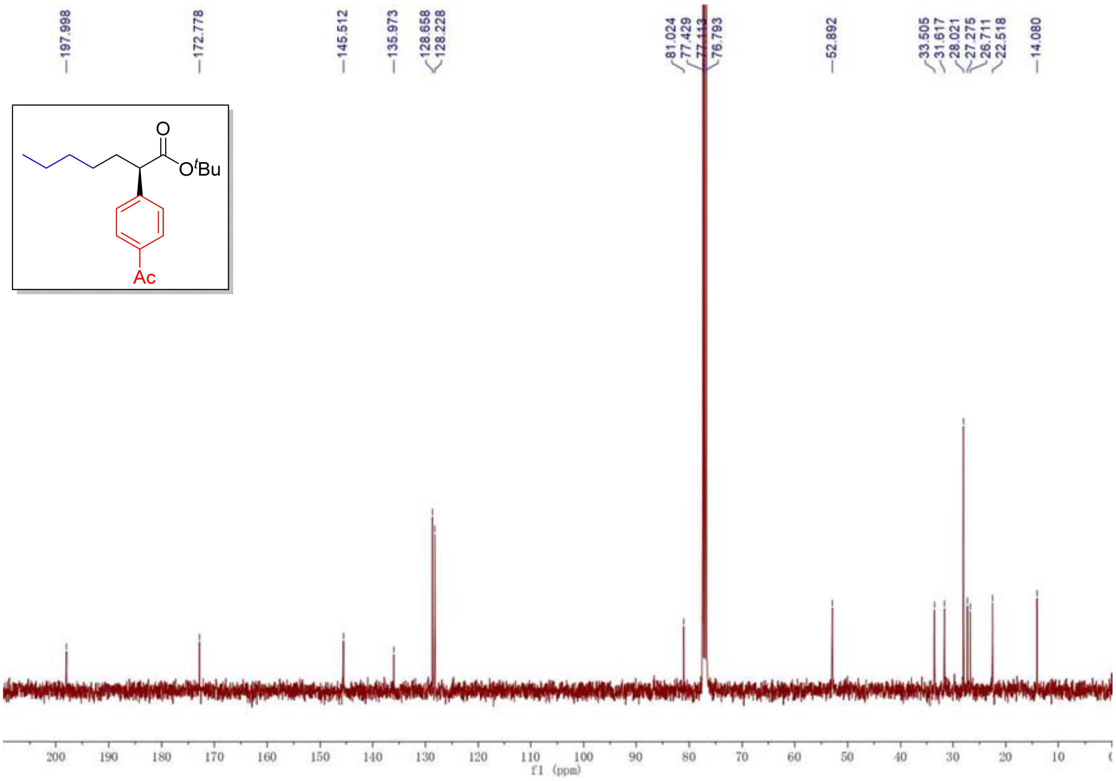

Supplementary Figure 43. ^{1}**H (400 MHz) and** ^{13}**C {**^{1}**H} (101 MHz) NMR spectra of 6a in** $CDCl_3$

(*R*)-*tert*-butyl 2-(4-acetylphenyl)-4-methylpentanoate (6b)

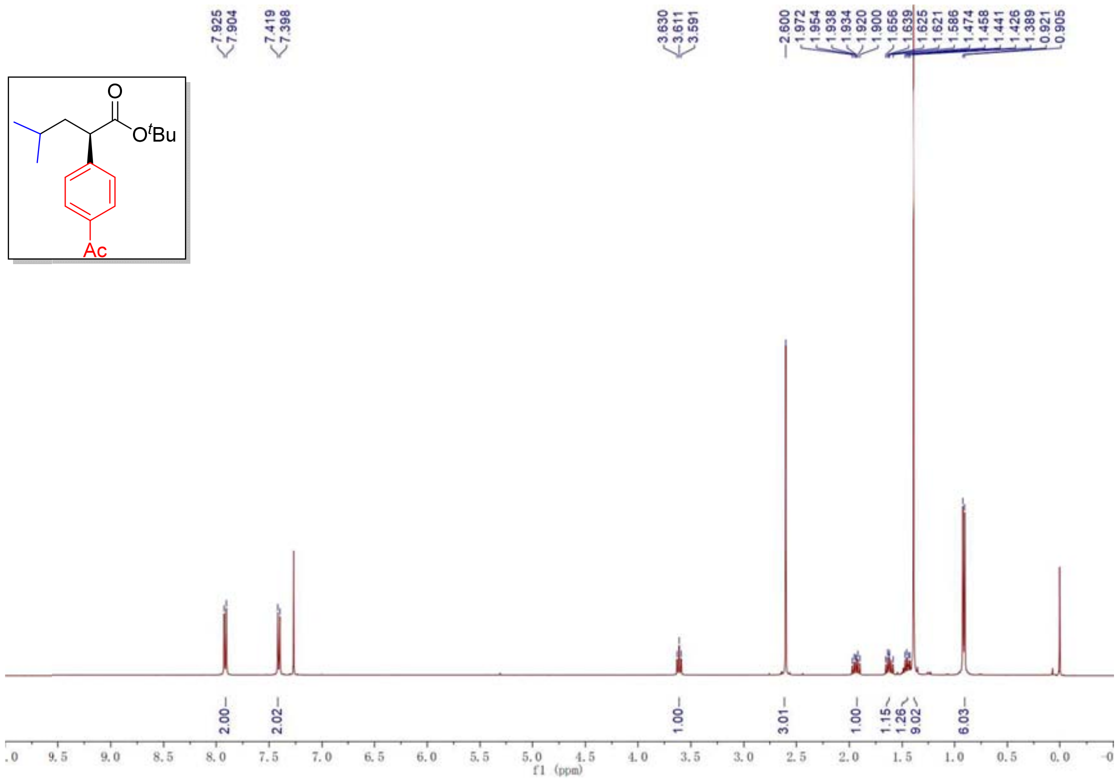

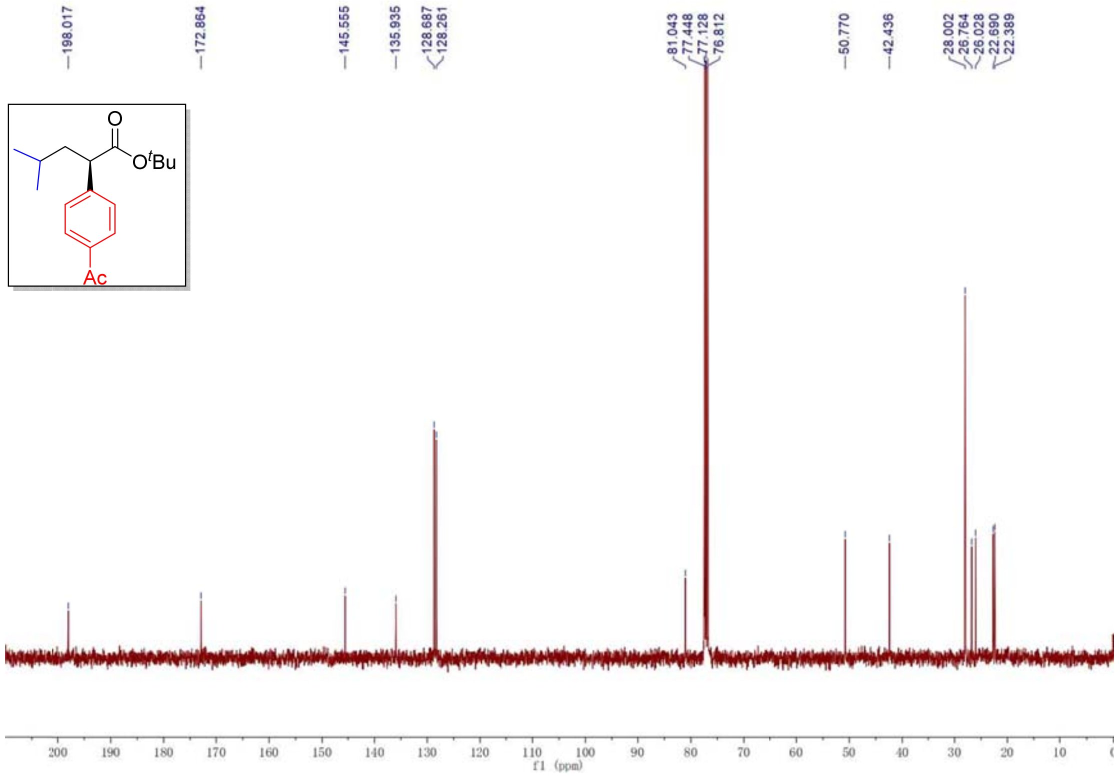

Supplementary Figure 44. ^{1}**H (400 MHz) and** ^{13}**C {**^{1}**H} (101 MHz) NMR spectra of 6b in** $CDCl_3$

(*R*)-*tert*-butyl 2-(4-acetylphenyl)-3-cyclopentylpropanoate (6c)

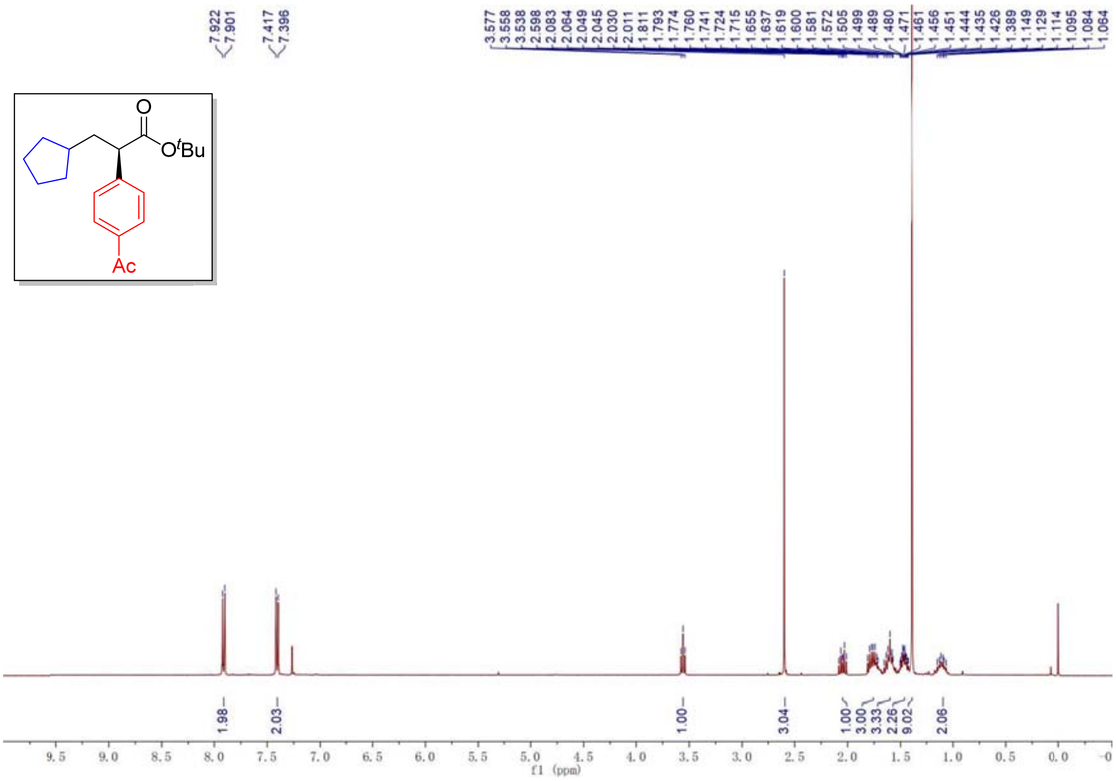

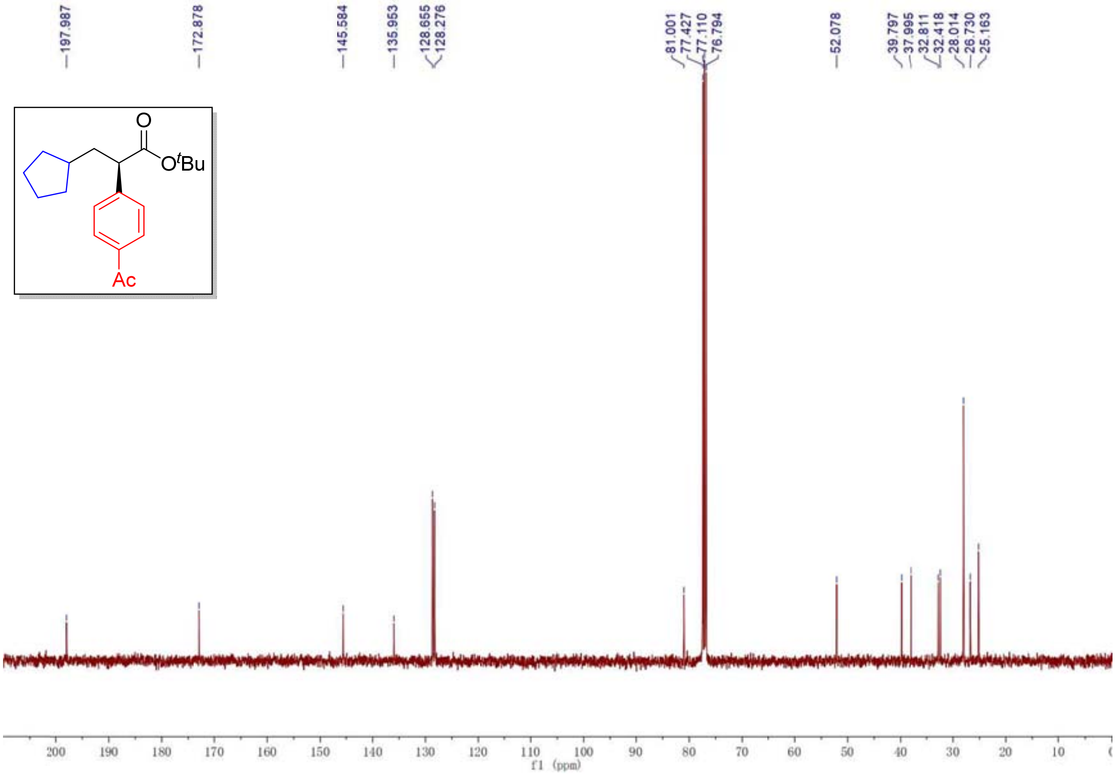

Supplementary Figure 45. **^{1}H (400 MHz) and ^{13}C {^{1}H} (101 MHz) NMR spectra of 6c in $CDCl_3$**

(*R*)-*tert*-butyl 2-(4-acetylphenyl)-3-cyclohexylpropanoate (6d)

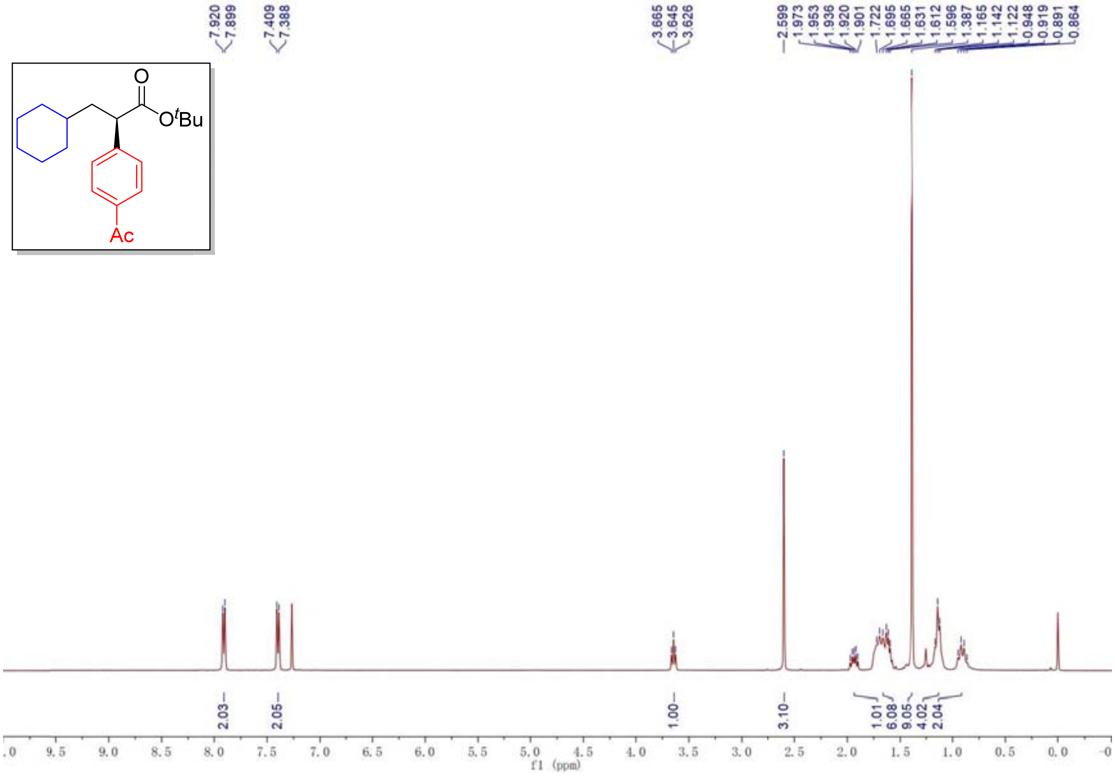

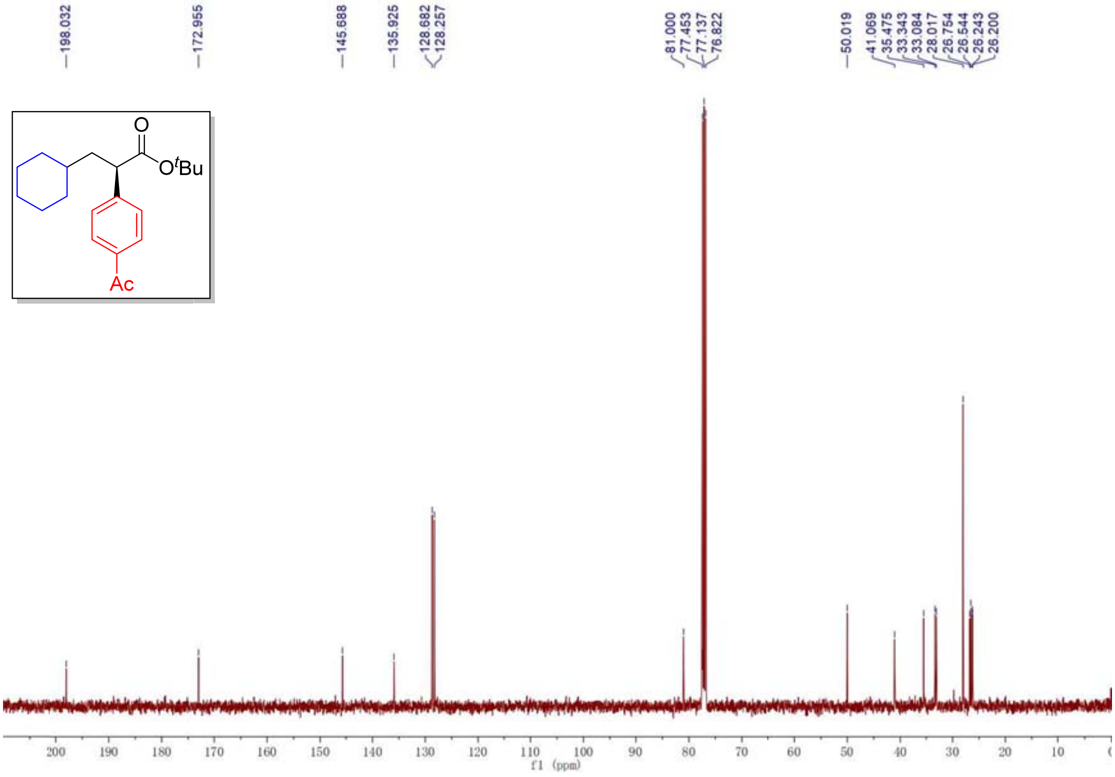

Supplementary Figure 46. ^{1}**H (400 MHz) and** ^{13}**C {**^{1}**H} (101 MHz) NMR spectra of 6d in** $CDCl_3$

(*R*)-*tert*-butyl 2-(4-acetylphenyl)-3-cycloheptylpropanoate (6e)

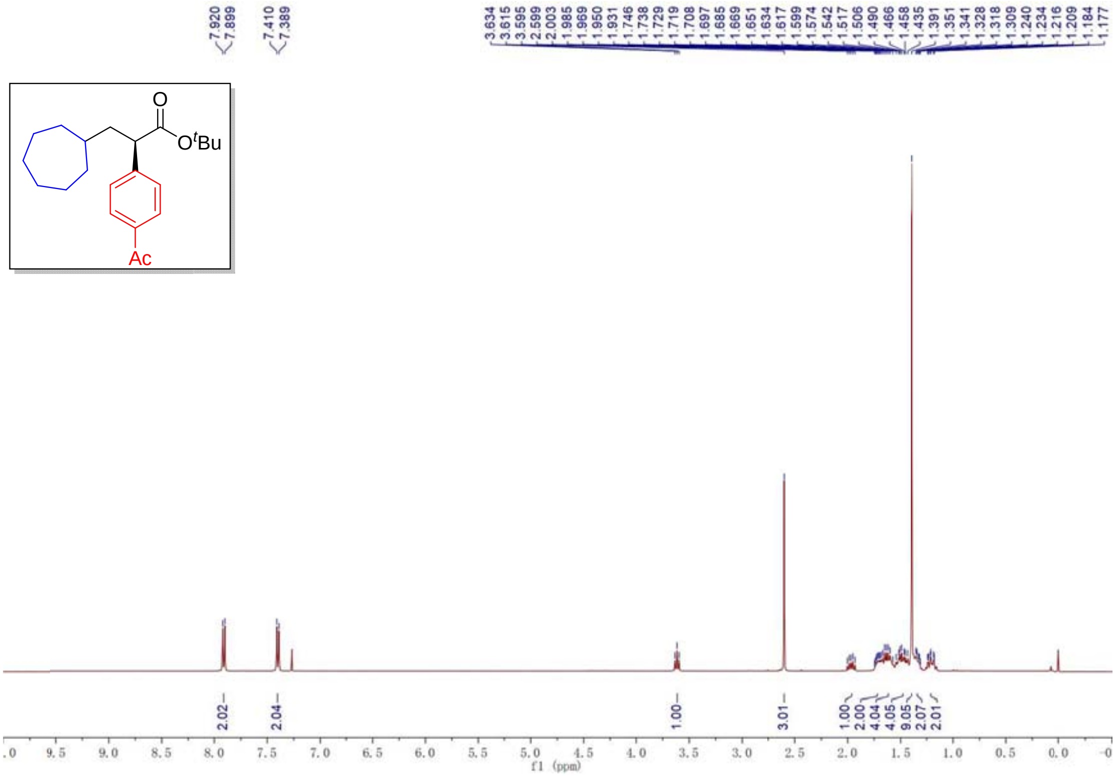

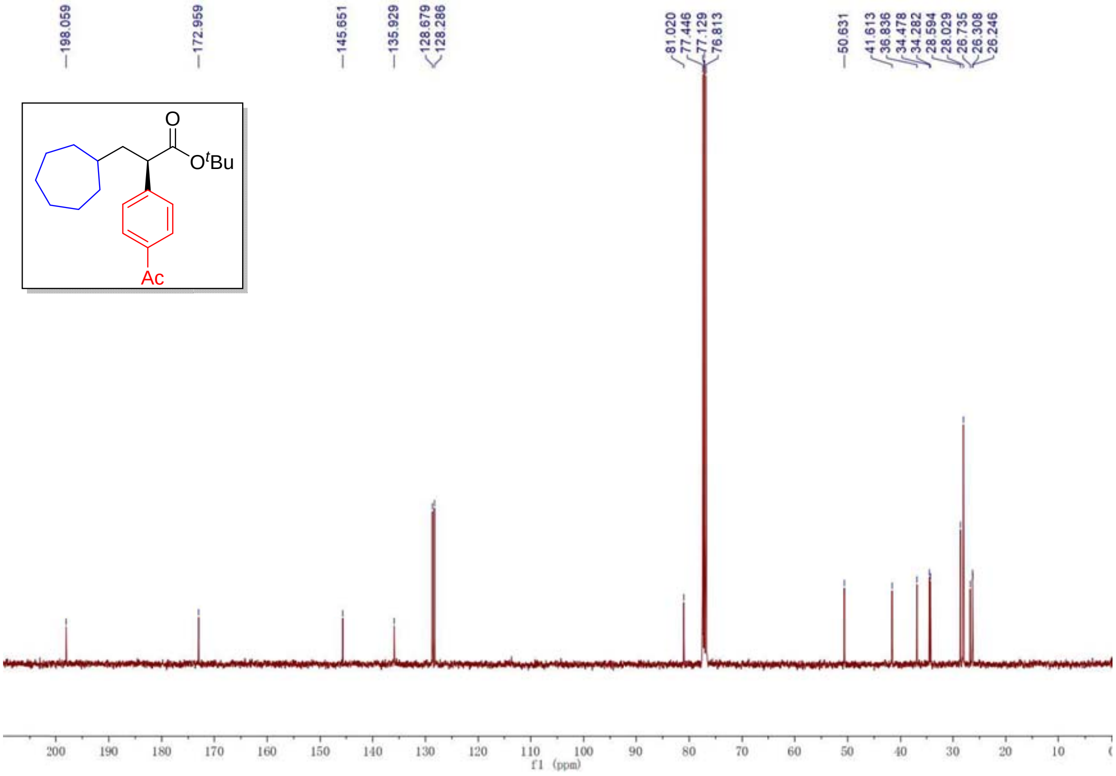

Supplementary Figure 47. ^{1}H (400 MHz) and ^{13}C {^{1}H} (101 MHz) NMR spectra of 6e in $CDCl_3$

(*R*)-*tert*-butyl 2-(4-acetylphenyl)-3-cyclooctylpropanoate (6f)

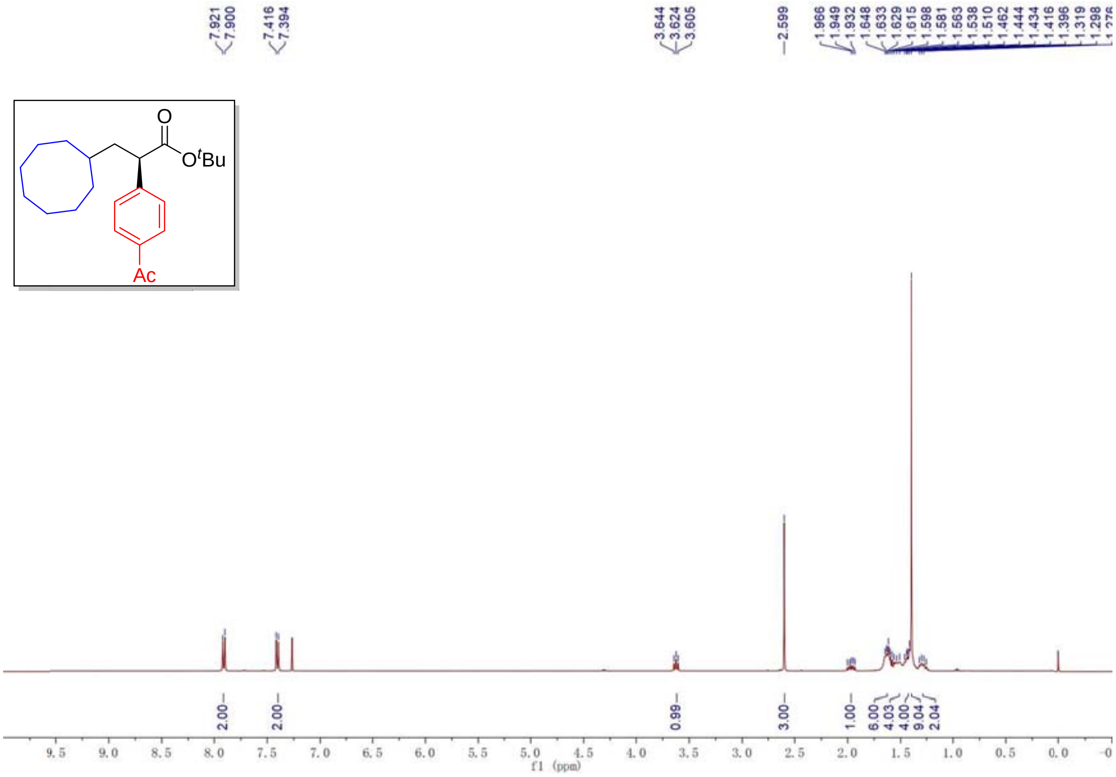

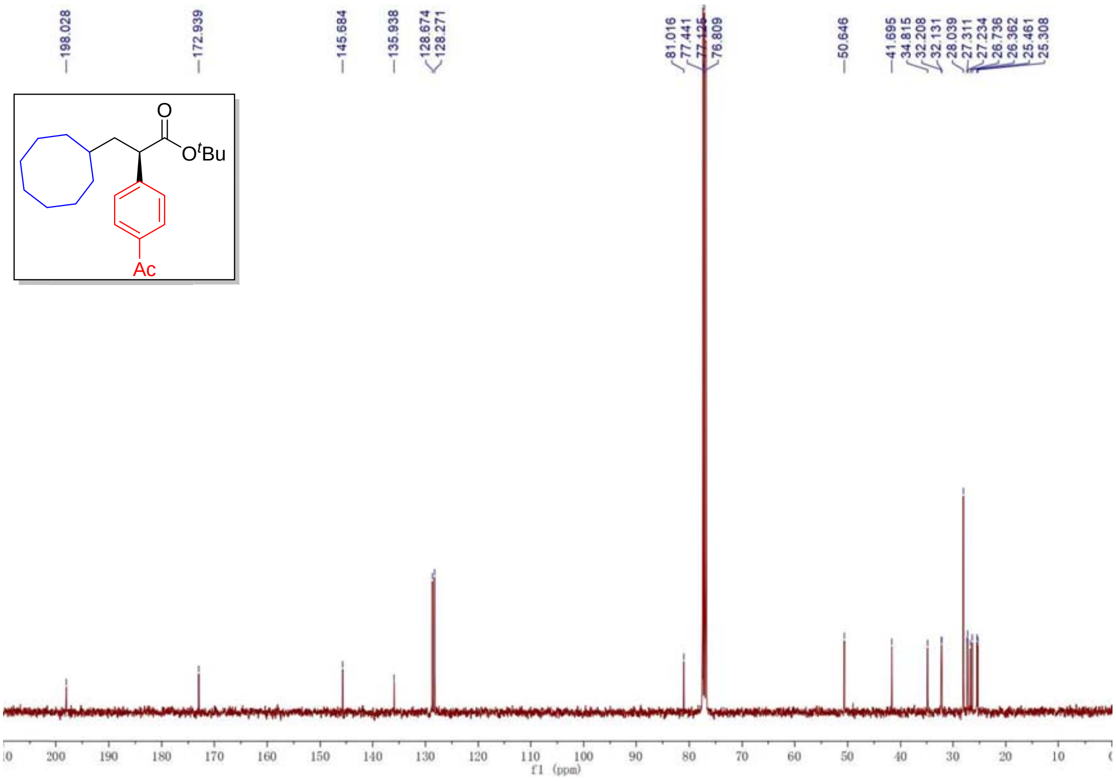

Supplementary Figure 48. ^{1}H (400 MHz) and ^{13}C {^{1}H} (101 MHz) NMR spectra of 6f in $CDCl_3$

(*R*)-2-(4-acetylphenyl)-4,4-dimethyl-N-phenylpentanamide (6g)

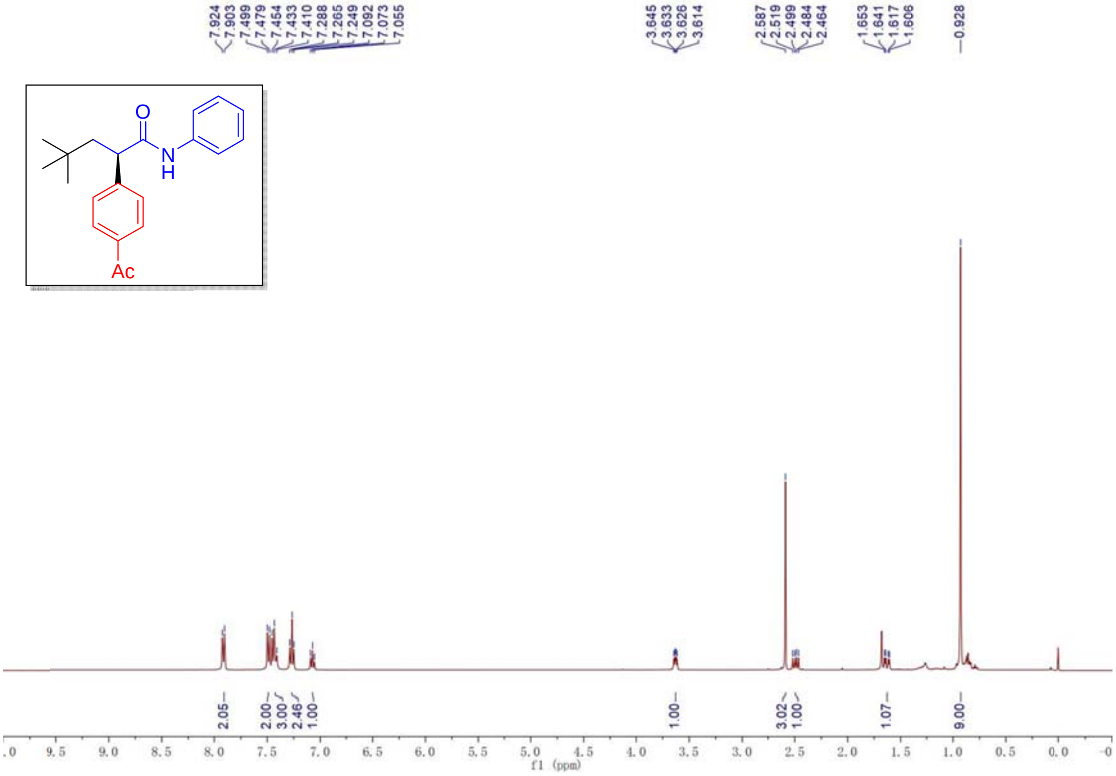

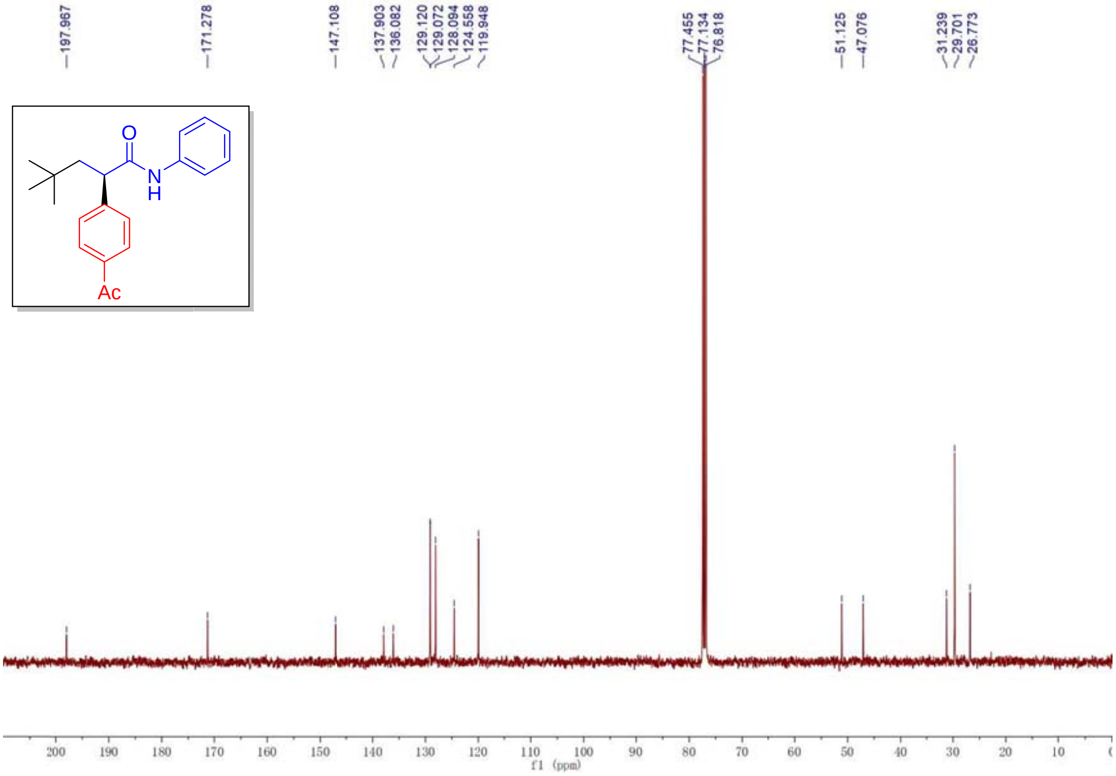

Supplementary Figure 49. **^{1}H (400 MHz) and ^{13}C {^{1}H} (101 MHz) NMR spectra of 6g in $CDCl_3$**

(*R*)-1-(4-acetylphenyl)-3,3-dimethylbutyl benzoate (6h)

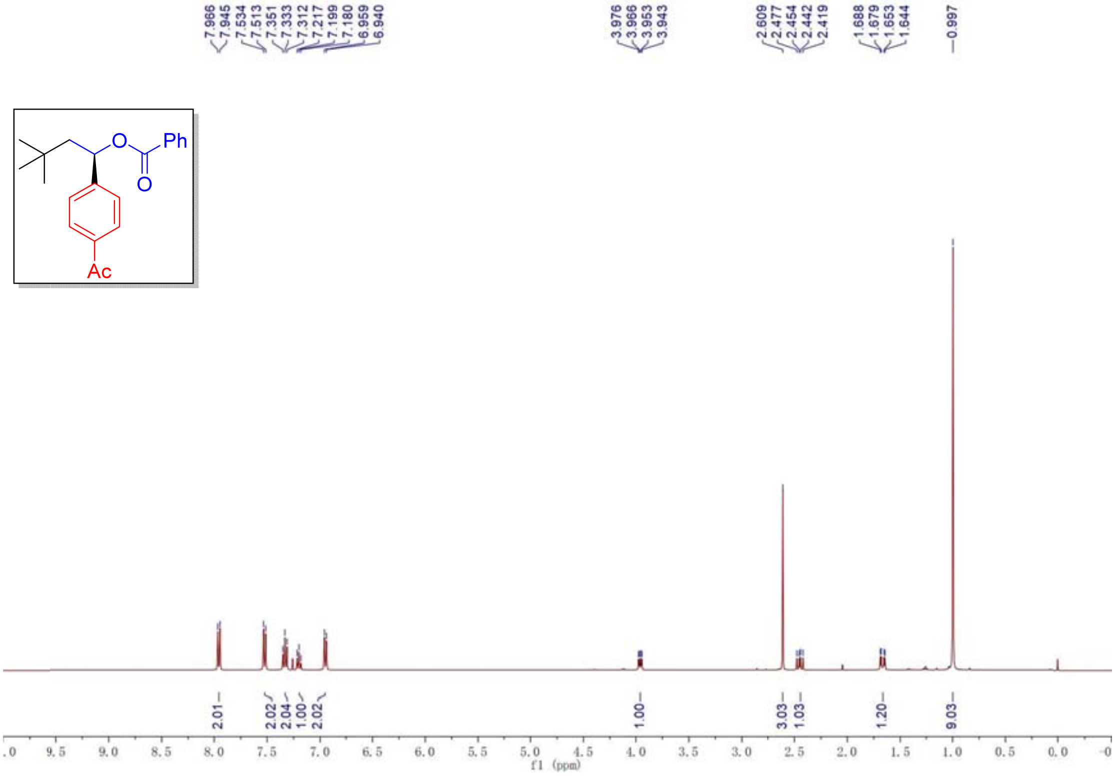

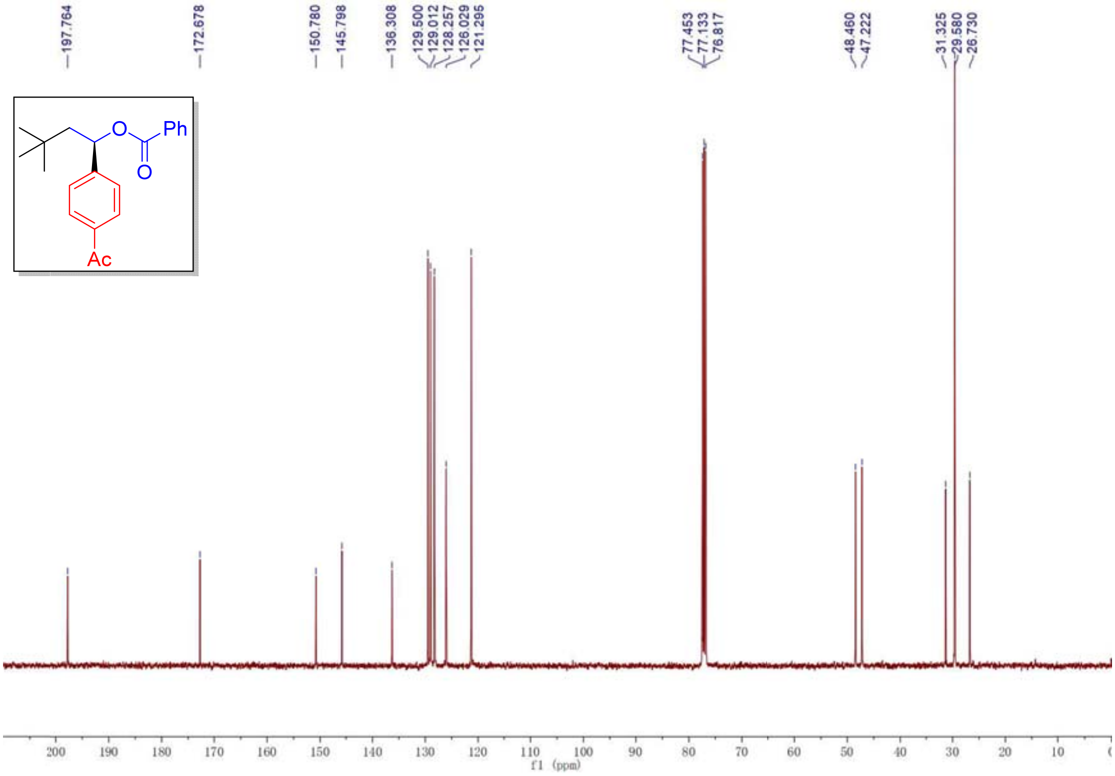

Supplementary Figure 50. ^{1}**H (400 MHz) and** ^{13}**C {**^{1}**H} (101 MHz) NMR spectra of 6h in CDCl**$_3$

Supplementary methods

All reactions were carried out under an atmosphere of dry argon. Anhydrous *N,N*-dimethylacetamide (DMA) was purchased from J&K and used without further purification. Unless otherwise stated, reagents were commercially available and used as purchased. Chemicals were obtained from Sigma-Aldrich, Acros, Energy Chemical, TCI China or Alfa Aesar. The progress of the reactions was monitored by thin-layer chromatography (TLC). Flash chromatography was performed with Qingdao Haiyang flash silica gel (200–300 mesh). The NMR spectra were obtained using a Brüker 400 MHz Fourier-transform NMR spectrometer. Chemical shifts were reported in units of parts per million (ppm) downfield from tetramethylsilane (TMS), and all coupling constants were reported in hertz. Specific rotations were measured with a Rudolph-Autopol III Polarimeter at the indicated temperature (25 ℃) with a sodium lamp (D line, 589 nm). The infrared spectra were obtained with KBr plates by using an IS10 FT-IR Spectrometer (ThermoFisher Corporation). High resolution mass spectrometry (HRMS) data were obtained on a Waters LC-TOF mass spectrometer (Xevo G2-XS QTof) using electrospray ionization (ESI) in positive or negative mode. Melting points were measured using a WRS-1C Melt-Temp apparatus and were uncorrected. Enantiomeric excesses (ee) were determined on an Agilent 1260 HPLC system using a chiral column (Daicel Chiralcel IC-3, Daicel Chiralcel IA, Daicel Chiralpak AD-H) and isopropanol-hexanes as the eluent.

Systematic Study of Reaction Conditions (Table S1)

The screen of nickel salts

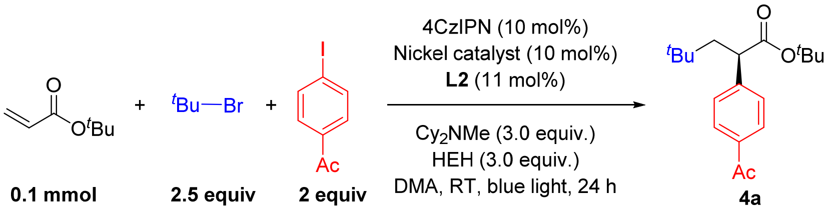

Entry	Nickel catalyst	DMA (mL)	GC yield (%)	ee (%)
1	Ni(COD)$_2$	1.0	20	86
2	$NiBr_2$	1.0	23	86
3	NiI_2	1.0	23	85
4	$NiBr_2$·DME	1.0	16	86
5	$NiCl_2$·DME	1.0	20	69
6	NiF_2	1.0	Trace	--
7	$NiCl_2[(PPh)_3]_2$	1.0	Trace	--

The screen of solvents

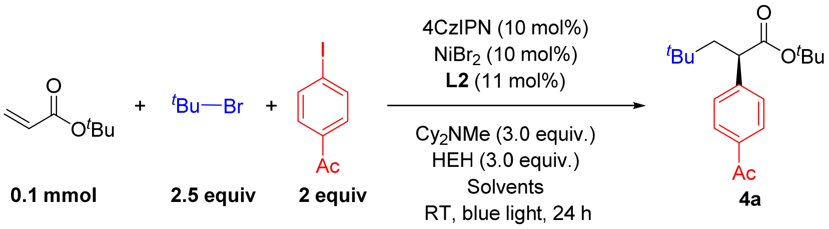

Entry	Solvent (1.0 mL)	GC yield (%)	ee (%)
1	DMA	25	86
2	DMF	5	--
3	DMSO	0	--
4	THF	Trace	--
5	NMP	10	82
6	CH_3CN	0	--
7	Toluene	0	--
8	acetone	17	75

The screen of reaction concentration

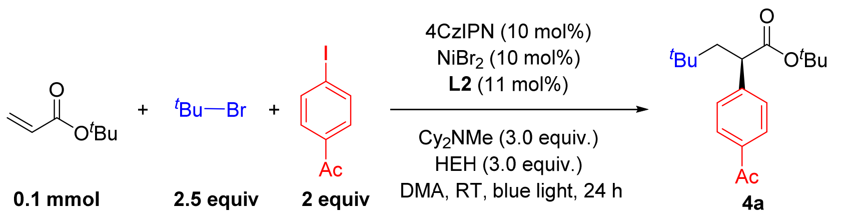

Entry	Solvent	Concentration (mL)	GC yield (%)	ee (%)
1	DMA	0.2	17	--
2	DMA	0.6	22	--
3	DMA	1.0	26	--
4	DMA	1.5	28	--
5	DMA	2.0	32	--
6	DMA	2.5	35	86
7	DMA	3.0	38	86
8	DMA	4.0	37	86

The screen of stoichiometry

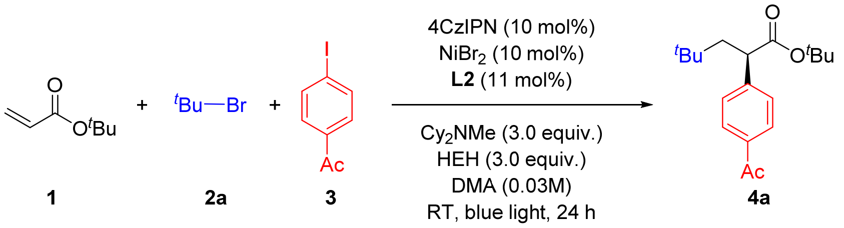

Entry	Ratio (**1**:**2a**:**3**)	GC yield (%)	ee (%)
1	1:2:1	17	--
2	1:2.5:1	22	--
3	1:3:1	24	--
4	1:4:1	29	--
5	1:5:1	30	--
6	1:3:2	44	86
7	1:4:2	54	86
8	1:5:2	40	--

The screen of Chiral ligand

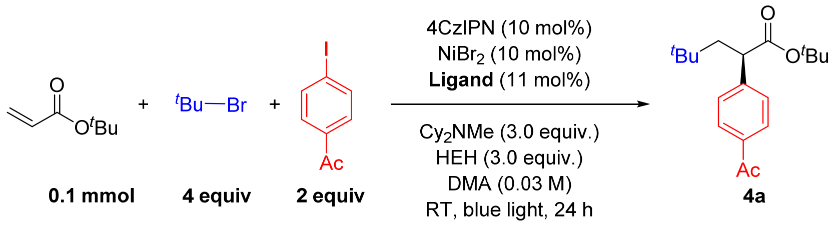

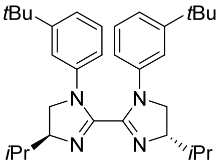

L6
ee: 92%

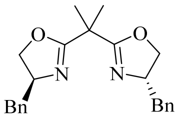

L7
ee: 8%

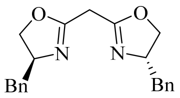

L8
Trace

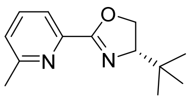

L9
ee: 18%

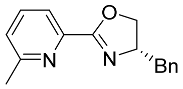

L10
ee: 20%

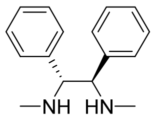

L11
Trace

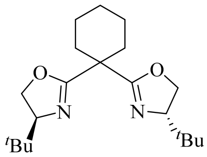

L12
Trace

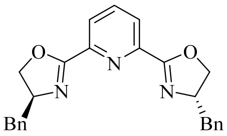

L13
Trace

The screen of Photocatalyst

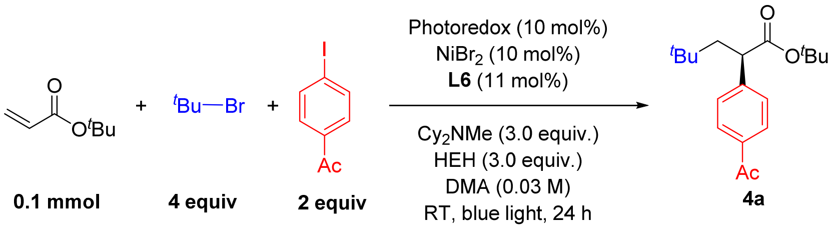

Entry	Photoredox (10% mol)	GC yield (%)	ee (%)
1	$Ir(ppy)_2(dtbbpy)PF_6$	55	90
2	4CzIPN	71	92
3	$(Ir[p\text{-}Fppy]_2(bpy))PF_6$	42	89
4	$Ru(phen)_3Cl_2$	60	90
5	Perylene	Trace	--

The screen of 3° -alkyl halides and aryl halides

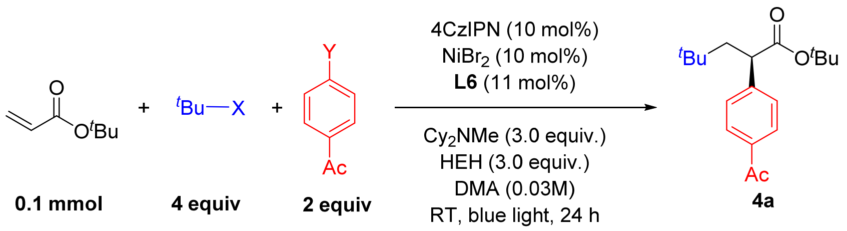

Entry	X	Y	GC yield (%)	ee (%)
1	Br	I	71	91
2	Br	Br	82	92
3	Br	Cl	Trace	--
4	I	I	14	--
5	I	Br	9	--
6	I	Cl	Trace	--
7	Cl	I	Trace	--
8	Cl	Br	Trace	--
9	Cl	Cl	Trace	--

The screen of acrylic esters

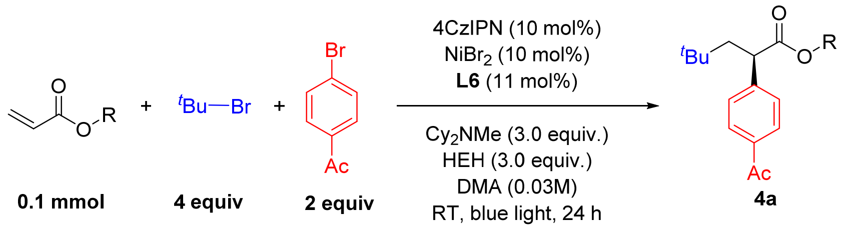

Entry	R	Isolated yield(%)	ee(%)
1	Me	25	74
2	Et	28	65
3	*t*Bu	79	92
4	Bn	31	55
5	Ph	35	47
6	Cy	49	59

The screen of the amount of photocatalyst

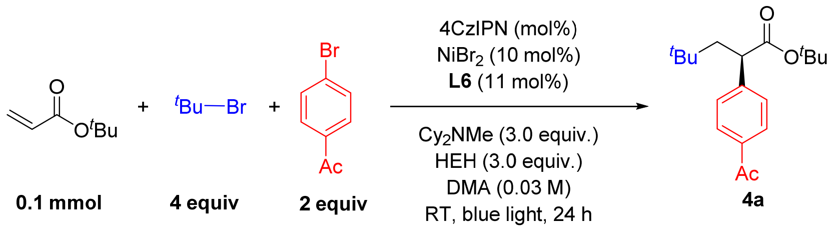

Entry	4CzIPN (mol%)	GC yield (%)	ee (%)
1	10	82	92
2	7.5	63	92
3	5	54	92
4	2.5	21	--

The screen of reaction time

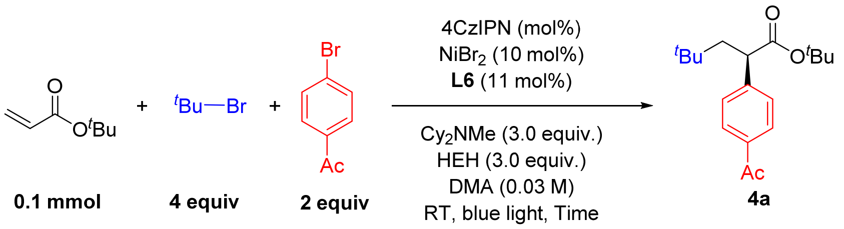

Entry	4CzIPN (mol%)	Reaction time (h)	GC yield (%)	ee (%)
1	10	12	43	--
2	10	24	82	92
3	10	36	83	92
4	10	48	79	92
5	5	12	21	--
6	5	24	54	92
7	5	36	56	92
8	5	48	54	92

The screen of base

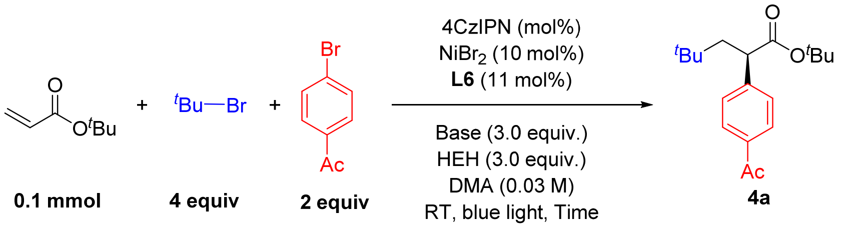

Entry	Base(3.0 equiv.)	GC yield (%)	ee (%)
1	Cy_2NMe	82	92
2	Et_3N	58	91
3	TMEDA	16	72
4	N,N'-Dimethyl-1,2-ethanediamine	Trace	--
5	Triethanolamine	Trace	--
6	N,N-Dimethylethylenediamine	50	91
7	Na_2CO_3	60	91
8	Cs_2CO_3	Trace	--

Additional studies using metallic reductants

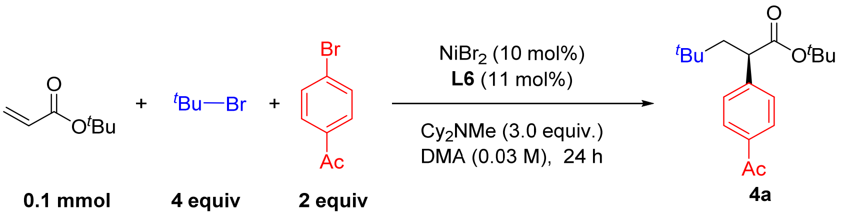

Entry	Reductant (3.0 eq.)	Temp (℃)	GC yield (%)	ee (%)
1	Zn	rt	Trace	--
2	Mn	rt	Trace	--
3	Zn	80	22	90
4	Mn	80	20	90
5	TDAE	rt	0	--

The group of Prof. Cristina Nevado has tested TDAE as reductant according to the reported literature[1]

Optimization of 2° -alkyl halides

The screen of 2° -alkyl halides and aryl halides

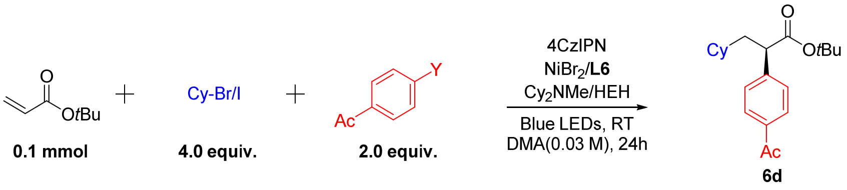

Entry	2° -alkyl halides	Y	GC yield (%)	ee (%)
1	Cy- Br	Br	0	--
2	Cy- I	Br	Trace	--
3	Cy-Br	I	0	--
4	Cy-I	I	53	83

The screen of Chiral ligand

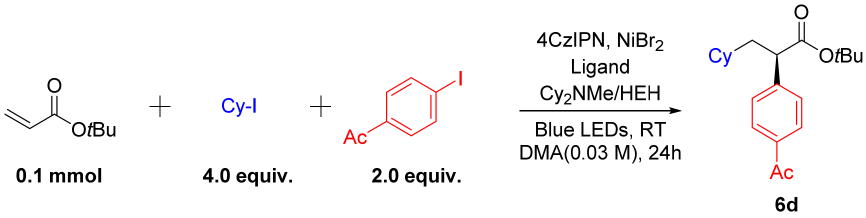

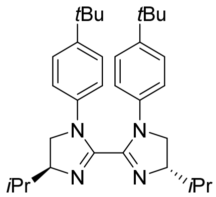

L4
GC yield: 20%
ee: 77%

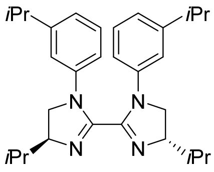

L5
GC yield: 23%
ee: 77%

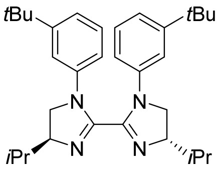

L6
GC yield: 53%
ee: 83%

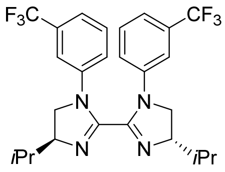

L14
GC yield: 50%
ee: 82%

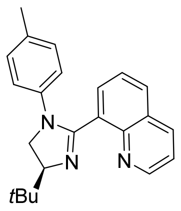

L15
GC yield: 5%
ee: --

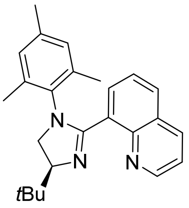

L16
GC yield: 4%
ee: --

The screen of solvents

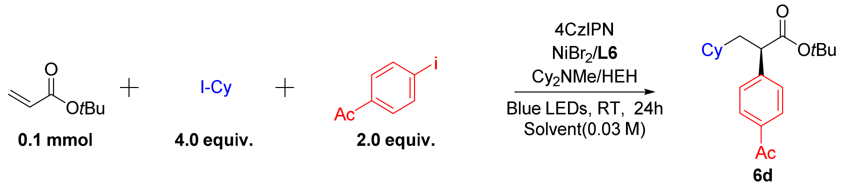

Entry	Solvents (3.0 mL)	GC yield (%)	ee (%)
1	DMA	54	83
2	DME	9	80
3	DMF	10	80
4	THF	28	90
5	EA	35	84
6	acetone	31	90
7	2-MeTHF	0	--
8	THF/DMA=2:1	48	85
9	THF/DMA=1:2	58	82
10	acetone /DMA=2:1	63	88
11	acetone /DMA=1:2	50	86

The screen of Nickel catalyst

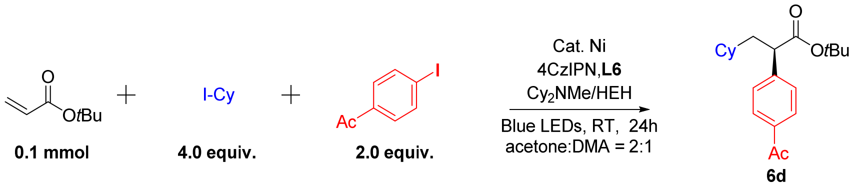

Entry	Nickel catalyst	GC yield (%)	ee (%)
1	$NiBr_2$	63	88
2	$(PCy_3)_2NiCl_2$	54	90
3	$NiCl_2$·glyme	68	90
4	$NiCl_2$·glyme [a]	30	91
5	$NiCl_2$·glyme [b]	21	92
6	$NiBr_2$·DME	Trace	--
7	$NiBr_2$·diglyme	32	89

[a] 3.0 mL acetone as solvent. [b] 3.0 mL THF as solvent.

Synthesis of the Ligand L6, 4CzIPN and Halide substrates

a. Synthesis of Ligand L6

Ligands **L6** were prepared according to the reported procedures[2-3]

b. Synthesis of organic photoredox catalyst 4CzIPN

Organic photoredox catalyst 4CzIPN was prepared according to the reported procedure[4]

c. Synthesis of Halide substrates

Compound **3ac** and **3ad** were prepared according to the reported procedure[5]. Compound **2c**, **2i**, **2j**, **2k** and **2l** were prepared according to general literature procedures[6]. Compound **2d**, **2e**, **2f**, **2g** and **2h** were prepared

according to general literature procedures[7]

General Procedures for nickel/photoredox catalysis enantioselective reductive domino alkyl arylation of acrylates

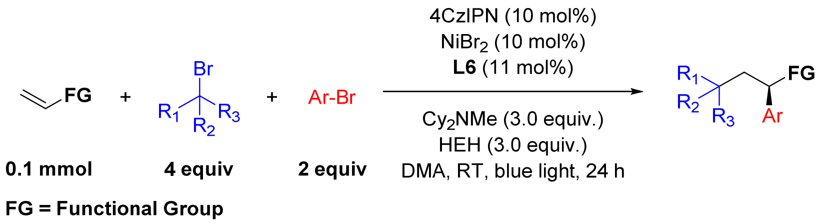

General Procedure 1: To an oven-dried vial equipped with a stir bar was added $NiBr_2$ (2.2 mg, 0.01 mmol), **L6** (5.4 mg, 0.011 mmol) and DMA (3.0 mL) under an argon atmosphere inside a glove box at RT. After 30 minutes, the light green mixture was added olefin (0.1 mmol, 1.0 equiv.), 3°-alkyl bromide (0.4 mmol, 4.0 equiv.), aryl bromide (0.2 mmol, 2.0 equiv.), Cy_2NMe (58.5 mg, 64 μL, 0.3 mmol, 3.0 equiv.), HEH (75.9 mg, 0.3 mmol, 3.0 equiv.) and 4CzIPN (8.0 mg, 0.01 mmol). Once HEH and 4CzIPN were added, the solution turned from light green to yellow. The vial was sealed with a cap and removed from the glove box. The reaction mixture was stirred at RT under blue light (Figure 2). After 24 h, the mixture color was changed to light green again. EtOAc (10 mL) was added to the resulting solution, which was then washed with brine (5 mL x 5). The organic layer was dried over anhydrous Na_2SO_4, filtered and concentrated to give the crude product. The crude residue was purified by flash column chromatography to afford the corresponding product.

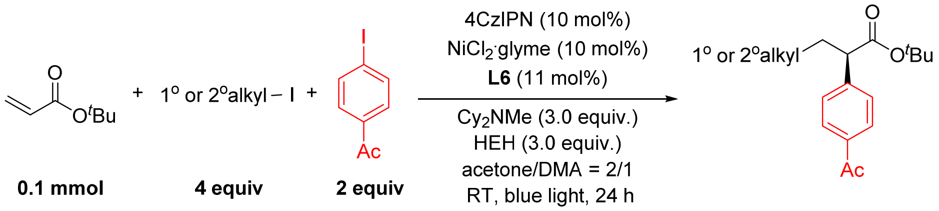

General Procedure 2: To an oven-dried vial equipped with a stir bar was added $NiCl_2$·glyme (2.2 mg, 0.01 mmol), **L6** (5.4 mg, 0.011 mmol) and acetone/DMA (2.0/1.0, v/v, 3.0 mL) under an argon atmosphere inside a glove box at RT. After 30 minutes, the light green mixture was added *tert*-Butyl acrylate (0.1 mmol, 1.0 equiv.), 1° or 2°-alkyl iodides (0.4 mmol, 4.0 equiv.), 1-(4-iodophenyl)ethan-1-one (0.2 mmol, 2.0 equiv.), Cy_2NMe (58.5 mg, 64 μL, 0.3 mmol, 3.0 equiv.), HEH (75.9 mg, 0.3 mmol, 3.0 equiv.) and 4CzIPN (8.0 mg, 0.01 mmol). Once HEH and 4CzIPN were added, the solution turned from light green to yellow. The vial was sealed with a cap and removed from the glove box. The reaction mixture was stirred at RT under blue light (Figure 2). After 24 h, the mixture color was changed to light green again. EtOAc (10 mL) was added to the resulting solution, which was then washed with brine (5 mL x 5). The organic layer was dried over anhydrous Na_2SO_4, filtered and concentrated to give the crude product. The crude residue was purified by flash column chromatography to afford the corresponding product.

Mechanism studay

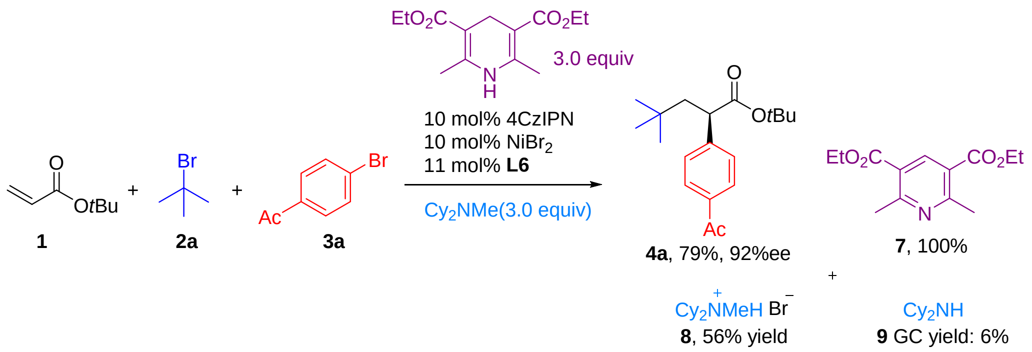

General Procedure: To an oven-dried vial equipped with a stir bar was added $NiBr_2$ (2.2 mg, 0.01 mmol), **L6** (5.4 mg, 0.011 mmol) and DMA (3.0 mL) under an argon atmosphere inside a glove box at RT. After 30 minutes, the light green mixture was added olefin 1 (0.1 mmol, 1.0 equiv.), *tert*-butyl bromide **2a** (0.4 mmol, 4.0 equiv.), aryl bromide **3a** (0.2 mmol, 2.0 equiv.), Cy_2NMe (58.5 mg, 64 μL, 0.3 mmol, 3.0 equiv.), HEH (75.9 mg, 0.3 mmol, 3.0 equiv.) and 4CzIPN (8.0 mg, 0.01 mmol). The vial was sealed with a cap and removed from the glove box. The reaction mixture was stirred at RT under blue light. After 24 h, EtOAc (5 mL) and hexanes (10 mL) were added to the resulting solution in the refrigerator overnight, compound **8** as a white precipitate was obtained by filtration (56% yield). Compound **9** (6% yield) was determined by GC using tetradecane as an internal standard. The filtrate was then washed with brine (5 mL x 5). The organic layer was dried over anhydrous Na_2SO_4, filtered and concentrated to give the crude product. The crude residue was purified by flash column chromatography to afford **4a** (79% yield, 92% ee) and pyridine **7** (100% yield relative to HEH).

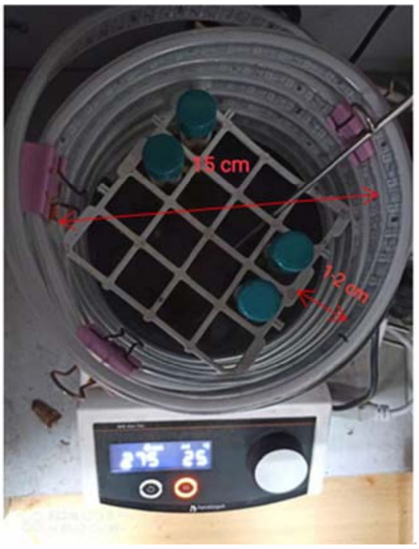

Figure 1

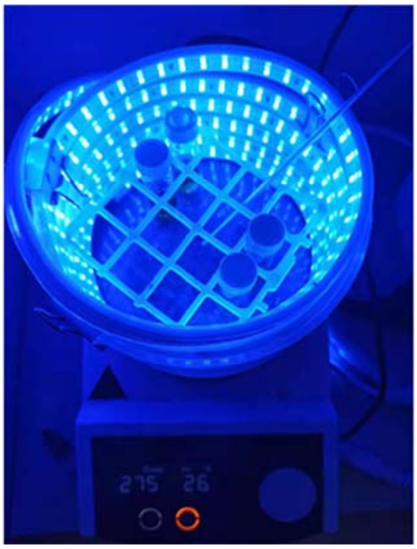

Figure 2

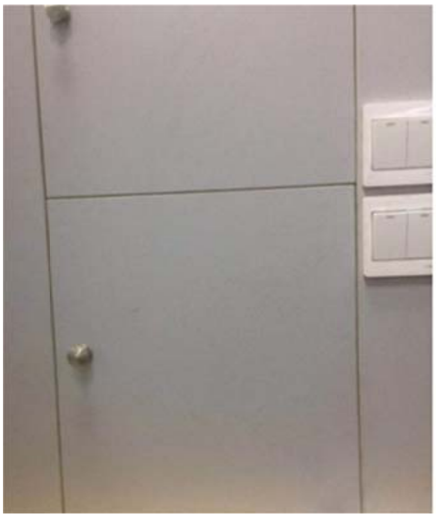

Figure 3

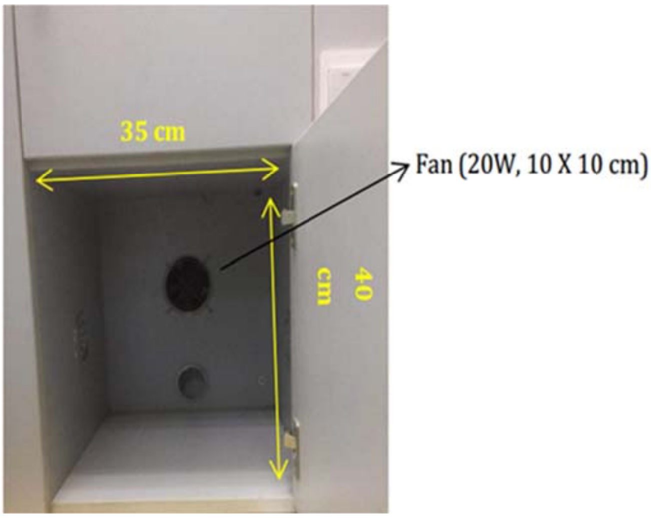

Figure 4

Note: The reaction was performed in a box with a fan (20W, 10 x 10 cm) to offset the heat generated from the LED belt (Figure 3 and 4). Even though, the reaction temperature would be maintained, during the course of reaction, at 29 - 33 ℃, which is slightly higher than room temperature. The blue LED belt (ADL, 450 nm, 12V) was commercially product.

Unsuccessful olefins in this reaction

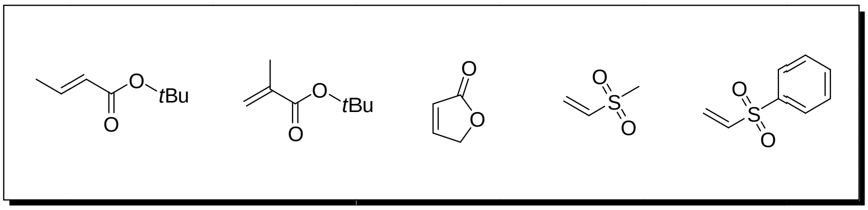

Characterization Data for Products

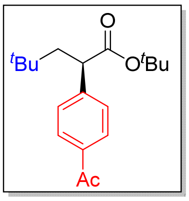

(*R*)-*tert*-Butyl 2-(4-acetylphenyl)-4,4-dimethylpentanoate (4a)

The reaction was performed following the General Procedure 1 with $NiBr_2$ (2.2 mg, 0.01 mmol) and **L6** (5.4 mg, 0.011 mmol), *tert*-butyl acrylate (15.0 μL, 0.1 mmol, 1.0 equiv.), 1-(4-bromophenyl)ethanone (39.8 mg, 0.2 mmol, 2.0 equiv.), ^{t}BuBr (54.8 mg, 44.9 μL, 0.4 mmol, 4.0 equiv.), Cy_2NMe (58.5 mg, 64 μL, 0.3 mmol), HEH (75.9 mg, 0.3 mmol) and 4CzIPN (8.0 mg, 0.01 mmol) in DMA (3.0 mL). The crude product was purified by flash chromatography on silica gel (eluted with petroleum ether:EtOAc = 20:1) to give the product (24.0 mg, 79% yield, 92% ee) as a white solid. Melting point: 74.8 – 76.6 ^{o}C; R_f = 0.5 (petroleum ether:EtOAc = 20:1). The spectroscopic data for this product match the literature data[8]. The ee was determined by HPLC with a Daicel Chiralcel IA column (^{i}PrOH/hexanes = 0.5/99.5, 1.0 mL/min, 254 nm, major t_r = 9.092 min (*S*), minor t_r = 10.709 min (*R*)). FTIR (neat, cm^{-1}) ν: 3428, 2978, 2960, 2867, 1721, 1687, 1365, 1146, 959, 833, 800, 596. The optical rotation of the product generated in the presence of **L6** was measured as $[\alpha]_D^{25}$ = –31.73 (*c* = 0.68, $CHCl_3$), *lit.* $[\alpha]_D^{25}$ = –25.3562 (*c* = 0.2733, $CHCl_3$, *R* enantiomer, 90% ee). Based on the literature value[8], we assign our product as the *R* enantiomer.

4a. Racemic product

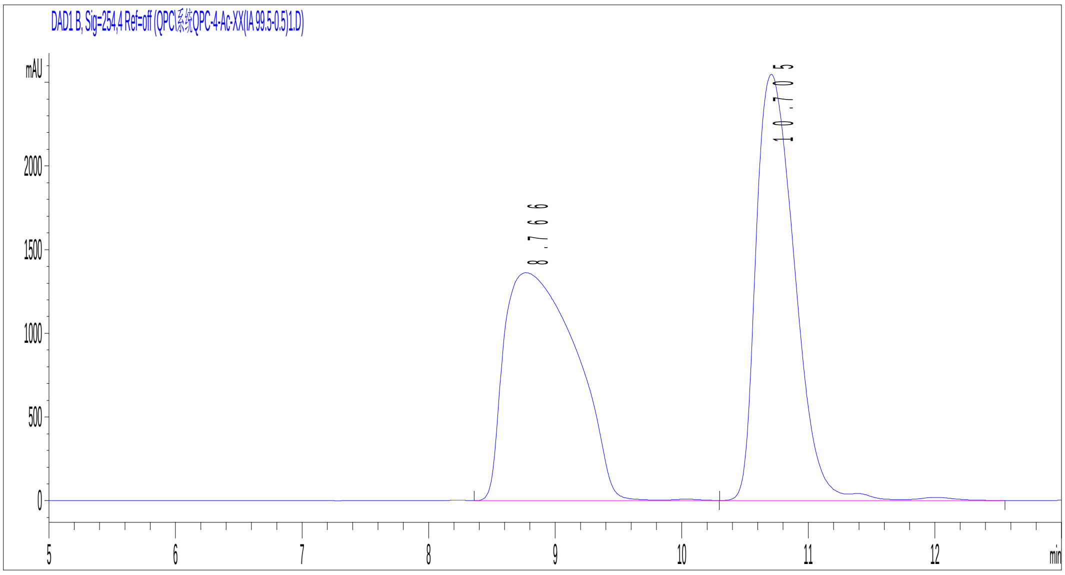

Peak	Ret Time	Type	Width	Area	Height	Area
#	[min]		[min]	mAU*s	[mAU]	%
1	8.766	BV R	0.7088	54136.2	1361.6	49.994
2	10.705	BV R	0.3406	54148.9	2546.7	50.006
Totals				108285.1	3908.3	

4a. Enantioenriched product, 92% ee

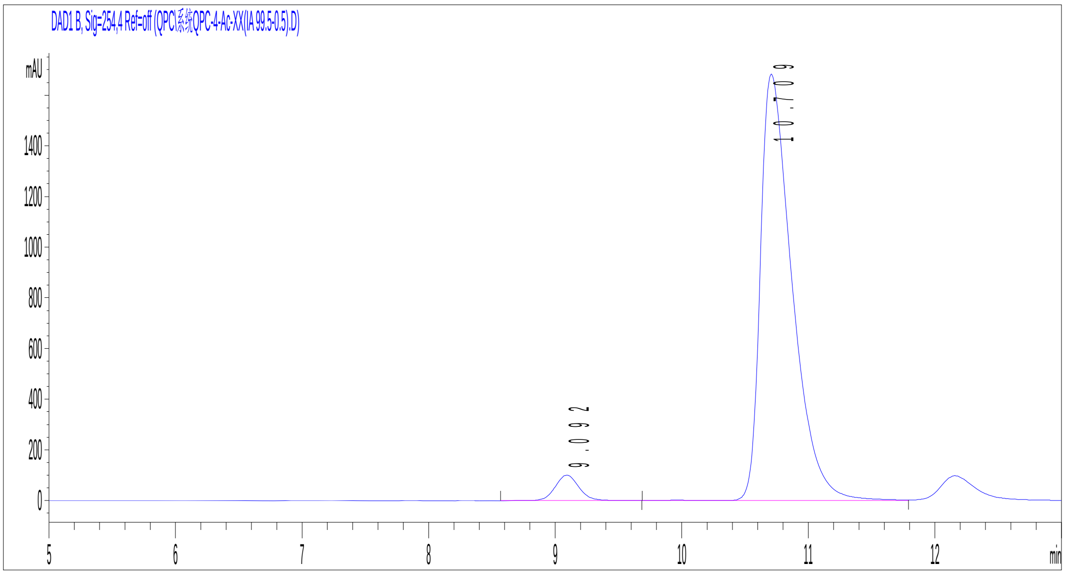

Peak	Ret Time	Type	Width	Area	Height	Area
#	[min]		[min]	mAU*s	[mAU]	%
1	9.092	BB	0.2021	1307.3	100.3	4.272
2	10.709	VV R	0.2628	29290.6	1683.1	95.728
Totals				30597.9	1783.4	

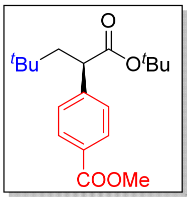

(*R*)-methyl 4-(1-(*tert*-butoxy)-4,4-dimethyl-1-oxopentan-2-yl)benzoate (4b)

The reaction was performed following the General Procedure 1 with $NiBr_2$ (2.2 mg, 0.01 mmol) and **L6** (5.4 mg, 0.011 mmol), *tert*-butyl acrylate (15.0 μL, 0.1 mmol, 1.0 equiv.), methyl 4-bromobenzoate (43.0 mg, 0.2 mmol, 2.0 equiv.), ^{t}BuBr (54.8 mg, 44.9 μL, 0.4 mmol, 4.0 equiv.), Cy_2NMe (58.5 mg, 64 μL, 0.3 mmol), HEH (75.9 mg, 0.3 mmol) and 4CzIPN (8.0 mg, 0.01 mmol) in DMA (3.0 mL). The crude product was purified by flash chromatography on silica gel (eluted with petroleum ether:EtOAc = 50:1) to give the product (19.2 mg, 60% yield, 91% ee) as a white solid. Melting point: 63.9 – 65.7 °C; R_f = 0.50 (petroleum ether:EtOAc = 20:1). $[\alpha]_D^{25}$ = –21.12 (*c* = 1.04, $CHCl_3$), *lit.* $[\alpha]_D^{25}$ = –28.3204 (*c* = 0.2567, $CHCl_3$, 97% ee). The spectroscopic data for this product match the literature data[8]. The ee was determined by HPLC with a Daicel Chiralcel IC-3 column (^{i}PrOH/hexanes = 0.5/99.5, 1.0 mL/min, 254 nm, major t_r = 10.437 min (*S*), minor t_r = 11.031 min (*R*)). FTIR (neat, cm^{-1}) ν: 2996, 2952, 2864, 1723, 1609, 1366, 1279, 1144, 1020, 844, 756, 703.

4b. Racemic product

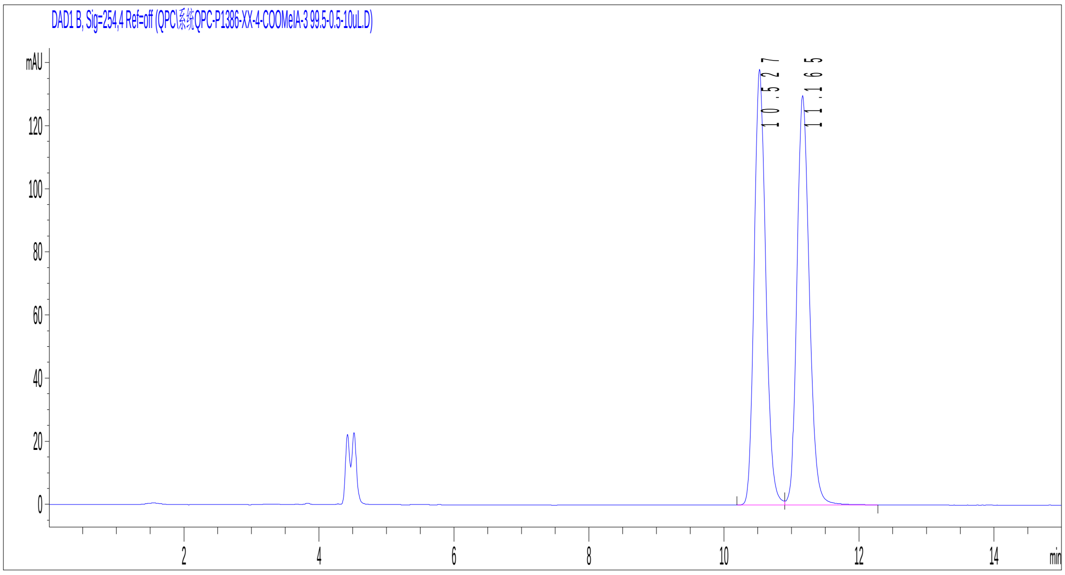

Peak	Ret Time	Type	Width	Area	Height	Area
#	[min]		[min]	mAU*s	[mAU]	%
1	10.527	BV	0.1853	1647.2	138	49.769
2	11.165	VB	0.1996	1662.5	129.7	50.231
Totals				3309.7	267.7	

4b. Enantioenriched, 91% ee

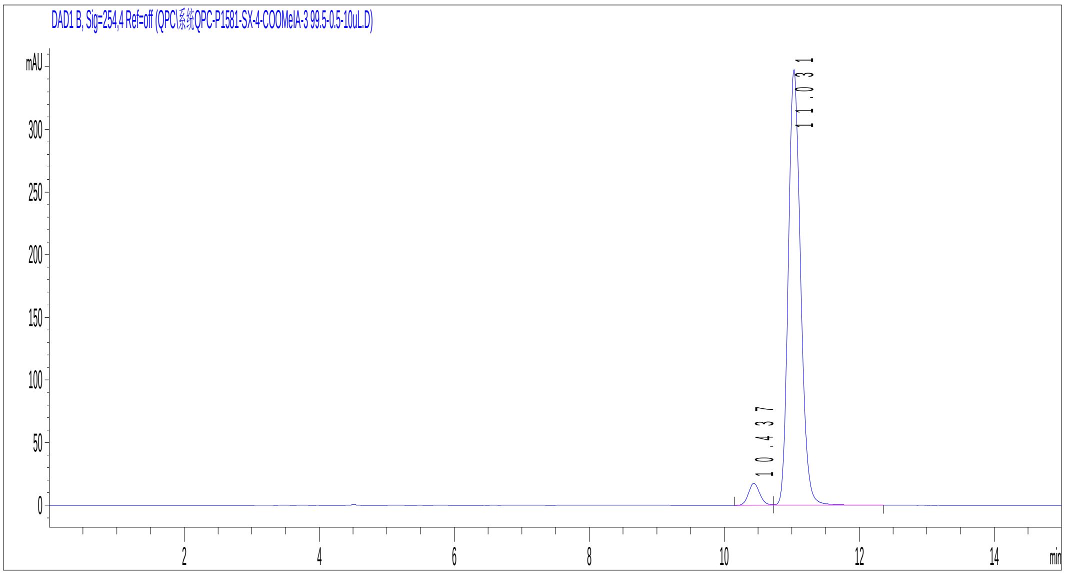

Peak	Ret Time	Type	Width	Area	Height	Area
#	[min]		[min]	mAU*s	[mAU]	%
1	10.437	BV	0.1795	204	17.6	4.422
2	11.031	VB	0.196	4409.8	347.7	95.578
Totals				4613.8	365.3	

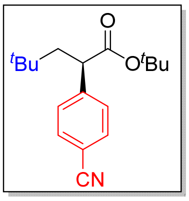

(*R*)-*tert*-butyl 2-(4-cyanophenyl)-4,4-dimethylpentanoate (4c)

The reaction was performed following the General Procedure 1 with $NiBr_2$ (2.2 mg, 0.01 mmol) and **L6** (5.4 mg, 0.011 mmol), *tert*-butyl acrylate (15.0 μL, 0.1 mmol, 1.0 equiv.), 4-bromobenzonitrile (36.4 mg, 0.2 mmol, 2.0 equiv.), ^{t}BuBr (54.8 mg, 44.9 μL, 0.4 mmol, 4.0 equiv.), Cy_2NMe (58.5 mg, 64 μL, 0.3 mmol), HEH (75.9 mg, 0.3 mmol) and 4CzIPN (8.0 mg, 0.01 mmol) in DMA (3.0 mL). The crude product was purified by flash chromatography on silica gel (eluted with petroleum ether:EtOAc = 50:1) to give the product (12.9 mg, 45% yield, 92% ee) as a white solid. Melting point: 64.9 – 66.6 °C; R_f = 0.48 (petroleum ether:EtOAc = 20:1). $[\alpha]_D^{25}$ = –23.56 (c = 0.457, $CHCl_3$), *lit.* $[\alpha]_D^{25}$ = –22.3676 (c = 0.3733, $CHCl_3$, 93% ee). The spectroscopic data for this product match the literature data[8]. The ee was determined by HPLC with a Daicel Chiralcel IA column (^{i}PrOH/hexanes = 0.2/99.8, 1.0 mL/min, 250 nm, major t_r = 11.322 min (*S*), minor t_r = 13.871 min (*R*)). FTIR (neat, cm^{-1}) ν: 2957, 2918, 2868, 2850, 2229, 1726, 1476, 1367, 1259, 1144, 1020, 837, 800.

4c. Racemic product

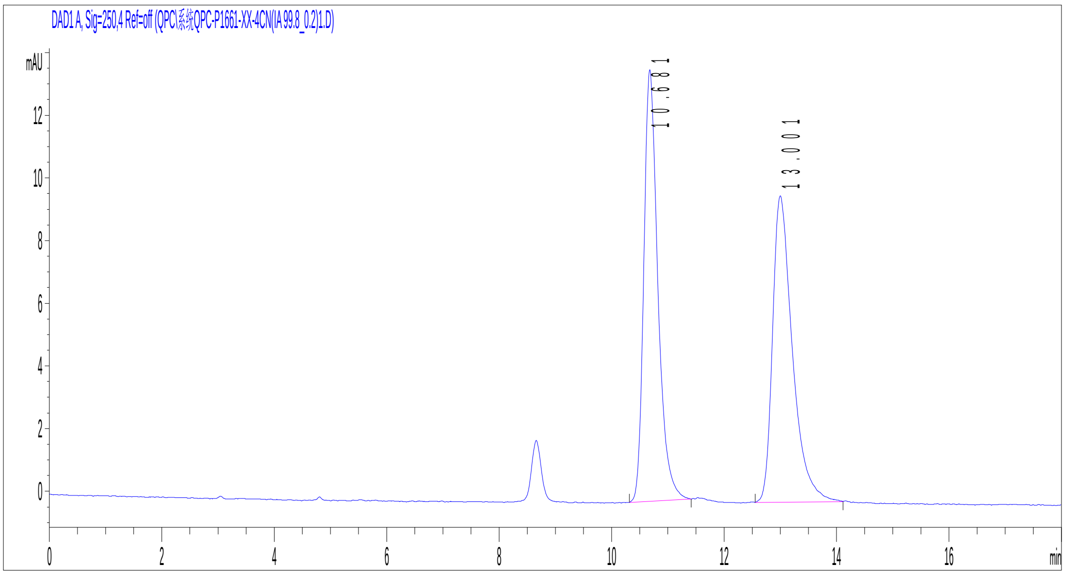

Peak	Ret Time	Type	Width	Area	Height	Area
#	[min]		[min]	mAU*s	[mAU]	%
1	10.681	BB	0.263	239.5	13.8	49.994
2	13.001	BB	0.3641	239.6	9.8	50.006
Totals				479.1	23.6	

4c. Enantioenriched, 92% ee

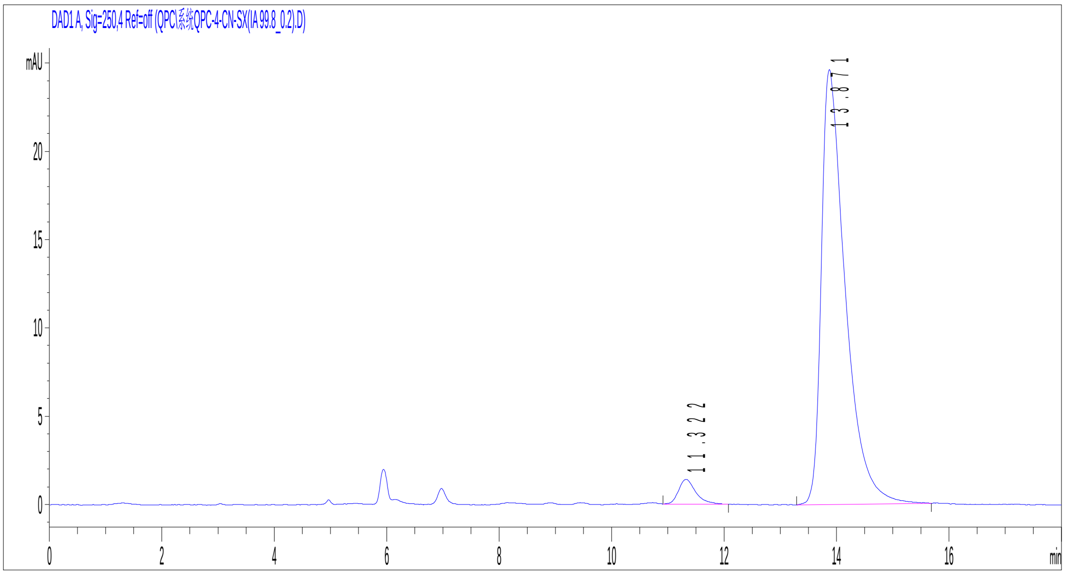

Peak	Ret Time	Type	Width	Area	Height	Area
#	[min]		[min]	mAU*s	[mAU]	%
1	11.322	BB	0.2959	29.8	1.4	3.926
2	13.871	BB	0.4355	729.4	24.6	96.074
Totals				759.2	26.0	

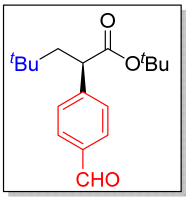

(*R*)-*tert*-butyl 2-(4-formylphenyl)-4,4-dimethylpentanoate (4d)

The reaction was performed following the General Procedure 1 with $NiBr_2$ (2.2 mg, 0.01 mmol) and **L6** (5.4 mg, 0.011 mmol), *tert*-butyl acrylate (15.0 μL, 0.1 mmol, 1.0 equiv.), 4-bromobenzaldehyde (37.0 mg, 0.2 mmol, 2.0 equiv.), ^{t}BuBr (54.8 mg, 44.9 μL, 0.4 mmol, 4.0 equiv.), Cy_2NMe (58.5 mg, 64 μL, 0.3 mmol), HEH (75.9 mg, 0.3 mmol) and 4CzIPN (8.0 mg, 0.01 mmol) in DMA (3.0 mL). The crude product was purified by flash chromatography on silica gel (eluted with petroleum ether:EtOAc = 50:1) to give the product (16.3 mg, 56% yield, 92% ee) as a white solid. Melting point: 81.3 – 82.2 ^{o}C; R_f = 0.44 (petroleum ether:EtOAc = 20:1). $[\alpha]_D^{25}$ = –44.63 (c = 0.357, $CHCl_3$), *lit.* $[\alpha]_D^{25}$ = –23.2669 (c = 0.3033, $CHCl_3$, 93% ee). The spectroscopic data for this product match the literature data[8]. The ee was determined by HPLC with a Daicel Chiralcel IA column (^{i}PrOH/hexanes = 3/97, 1.0 mL/min, 254 nm, major t_r = 4.524 min (*S*), minor t_r = 4.764 min (*R*)). FTIR (neat, cm^{-1}) ν: 3437, 2957, 2918, 2849, 1729, 1705, 1606, 1367, 1142, 872, 843, 799, 766.

4d. Racemic product

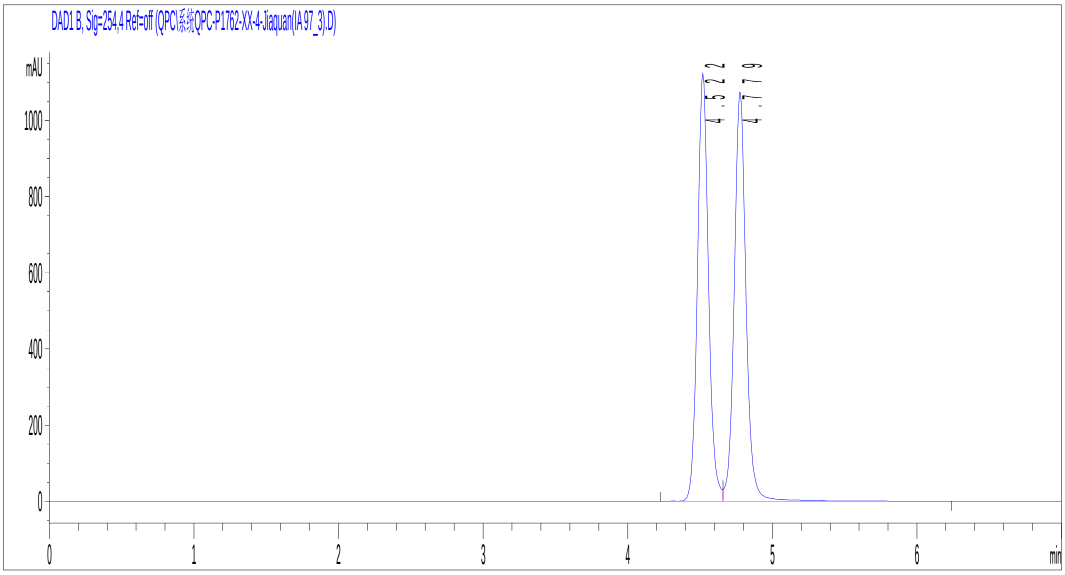

Peak	Ret Time	Type	Width	Area	Height	Area
#	[min]		[min]	mAU*s	[mAU]	%
1	4.522	VV R	0.0797	5939.4	1123.3	49.200
2	4.779	VB	0.0864	6132.6	1078.3	50.800
Totals				12072	2201.6	

4d. Enantioenriched product, 92% ee

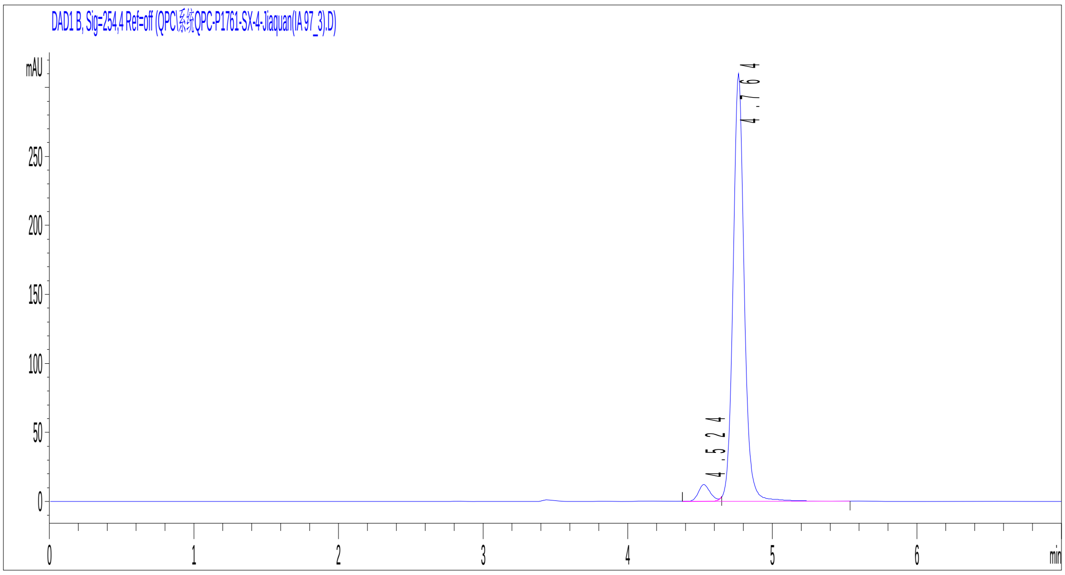

Peak	Ret Time	Type	Width	Area	Height	Area
#	[min]		[min]	mAU*s	[mAU]	%
1	4.524	BV E	0.0877	68.2	12.1	3.931
2	4.764	VB R	0.0807	1665.9	309.9	96.069
Totals				1734.1	322	

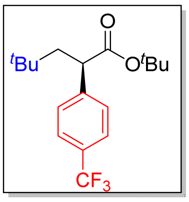

(*R*)-*tert*-Buty 4,4-dimethyl-2-(4-(trifluoromethyl)phenyl)pentanoate (4e)

The reaction was performed following the General Procedure 1 with $NiBr_2$ (2.2 mg, 0.01 mmol) and **L6** (5.4 mg, 0.011 mmol), *tert*-butyl acrylate (15.0 μL, 0.1 mmol, 1.0 equiv.), 4-Bromobenzotrifluoride (45.0 mg, 28.0 μL, 0.2 mmol, 2.0 equiv.), ^{t}BuBr (54.8 mg, 44.9 μL, 0.4 mmol, 4.0 equiv.), Cy_2NMe (58.5 mg, 64 μL, 0.3 mmol), HEH (75.9 mg, 0.3 mmol) and 4CzIPN (8.0 mg, 0.01 mmol) in DMA (3.0 mL). The crude product was purified by flash chromatography on silica gel (eluted with petroleum ether:EtOAc = 50:1) to give the product (25.1 mg, 76% yield, 93% ee) as a white solid. Melting point: 61.9 – 63.7 °C; R_f = 0.54 (petroleum ether:EtOAc = 20:1). $[\alpha]_D^{25}$ = –18.41 (c = 0.353, $CHCl_3$), *lit.* $[\alpha]_D^{25}$ = –19.2946 (c = 0.5333, $CHCl_3$, 92% ee). The spectroscopic data for this product match the literature data[8]. The ee was determined by HPLC with a Daicel Chiralcel AD-H column (^{i}PrOH/hexanes = 0.2/99.8, 1.0 mL/min, 230 nm, major t_r = 4.452 min (*S*), minor t_r = 4.679 min (*R*)). FTIR (neat, cm^{-1}) ν: 2956, 2929, 2853, 1719, 1367, 1325, 1260, 1151, 1109, 1069, 1020.

4e. Racemic product

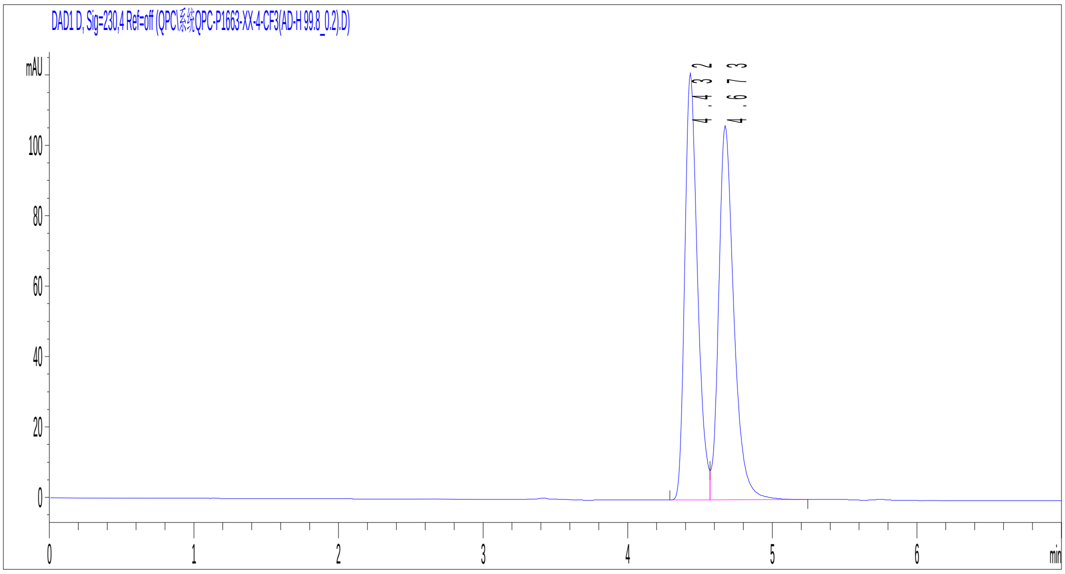

Peak	Ret Time	Type	Width	Area	Height	Area
#	[min]		[min]	mAU*s	[mAU]	%
1	4.432	BV	0.0935	742.5	121.2	48.841
2	4.673	VB	0.1114	777.8	106.3	51.159
Totals				1520.3	227.5	

4e. Enantioenriched product, 93% ee

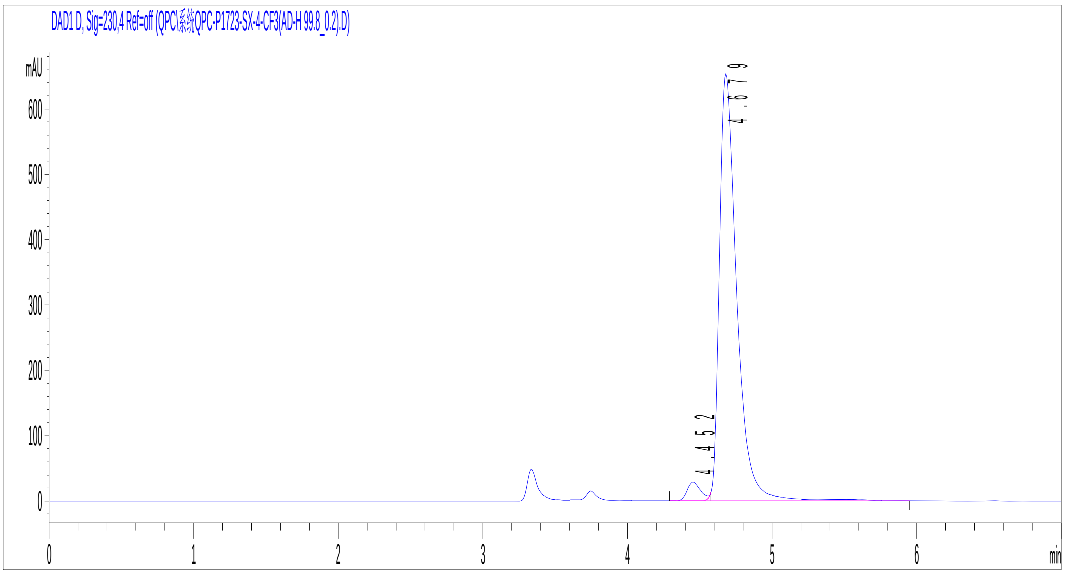

Peak	Ret Time	Type	Width	Area	Height	Area
#	[min]		[min]	mAU*s	[mAU]	%
1	4.452	BV E	0.0975	186.4	28.8	3.293
2	4.679	VV R	0.1251	5474.3	653.9	96.707
Totals				5660.7	682.7	

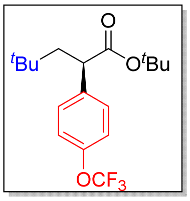

(*R*)-*tert*-butyl 4,4-dimethyl-2-(4-(trifluoromethoxy)phenyl)pentanoate (4f)

The reaction was performed following the General Procedure 1 with $NiBr_2$ (2.2 mg, 0.01 mmol) and **L6** (5.4 mg, 0.011 mmol), *tert*-butyl acrylate (15.0 μL, 0.1 mmol, 1.0 equiv.), 1-bromo-4-(trifluoromethoxy)benzene (48.2 mg, 29.7 μL, 0.2 mmol, 2.0 equiv.), ^{t}BuBr (54.8 mg, 44.9 μL, 0.4 mmol, 4.0 equiv.), Cy_2NMe (58.5 mg, 64 μL, 0.3 mmol), HEH (75.9 mg, 0.3 mmol) and 4CzIPN (8.0 mg, 0.01 mmol) in DMA (3.0 mL). The crude product was purified by flash chromatography on silica gel (eluted with petroleum ether:EtOAc = 80:1) to give the product (29.1 mg, 84% yield, 91% ee) as a white solid. Melting point: 41.2 – 42.4 °C; R_f = 0.51 (petroleum ether:EtOAc = 50:1). $[\alpha]_D^{25}$ = –21.55 (*c* = 0.413, $CHCl_3$), ^{1}H NMR (400 MHz, $CDCl_3$) δ: 7.32 (d, *J* = 8.6 Hz, 2H), 7.14 (d, *J* = 7.9 Hz, 2H), 3.54 (dd, *J* = 9.4, 3.4 Hz, 1H), 2.25 (dd, *J* = 14.0, 9.4 Hz, 1H), 1.38 (s, 9H), 0.91 (s, 9H). ^{13}C{^{1}H} NMR (101 MHz, $CDCl_3$) δ: 173.63, 148.08, 140.39, 129.06, 121.14 (q, *J* =69.69 Hz), 121.04, 80.81, 48.71, 47.32, 31.17, 29.54, 27.90. ^{19}F NMR (375 MHz, $CDCl_3$) δ: -57.78 (s). The ee was determined by HPLC with a Daicel Chiralcel AD-H column (^{i}PrOH/hexanes = 0.2/99.8, 1.0 mL/min, 250 nm, major t_r = 3.824 min (*S*), minor t_r = 4.02 min (*R*)). FTIR (neat, cm^{-1}) ν: 3436, 2960, 2934, 1730, 1508, 1368, 1261, 1221, 1143, 1020, 842, 804, 767. HRMS: calcd for $C_{18}H_{26}F_3O_3$ $[M+H]^+$ 347.1834, found 347.1836.

4f. Racemic product

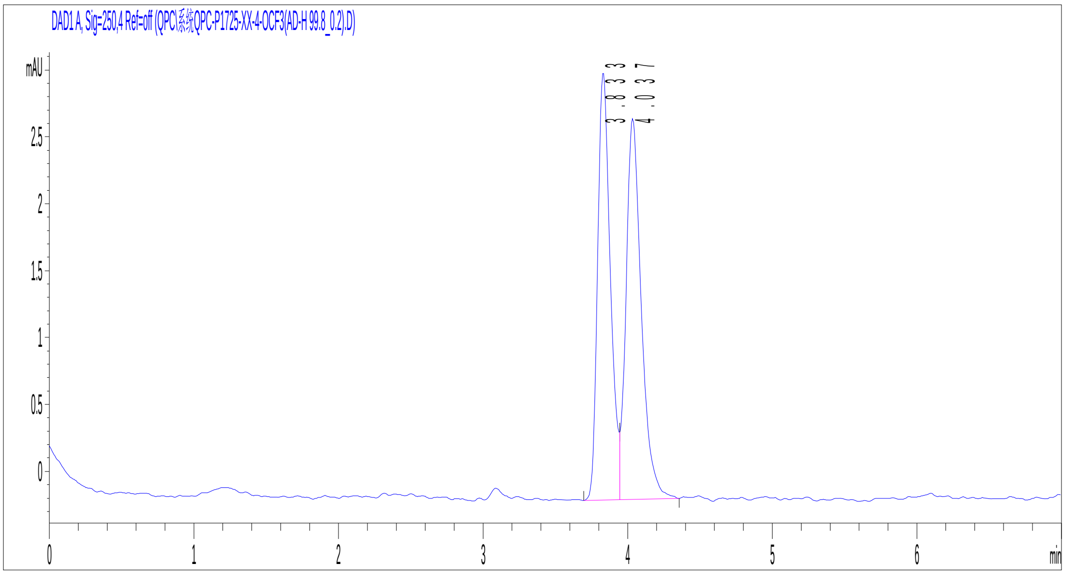

Peak	Ret Time	Type	Width	Area	Height	Area
#	[min]		[min]	mAU*s	[mAU]	%
1	3.833	BV	0.0928	19.4	3.2	48.039
2	4.037	VB	0.1102	21	2.8	51.961
Totals				40.4	6	

4f. Enantioenriched product, 91% ee

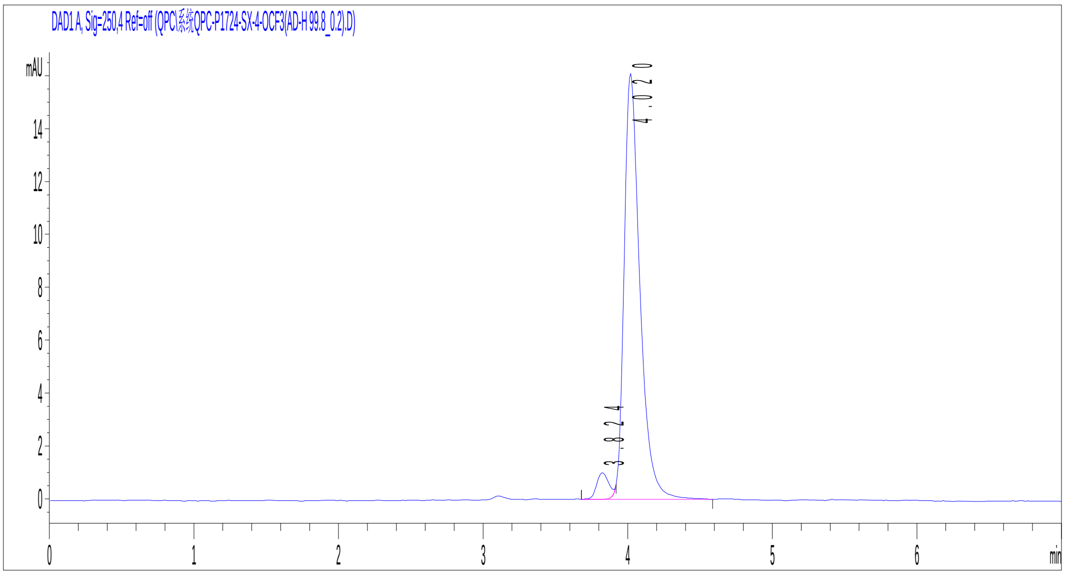

Peak	Ret Time	Type	Width	Area	Height	Area
#	[min]		[min]	mAU*s	[mAU]	%
1	3.824	BV E	0.0888	5.7	1	4.471
2	4.02	VB R	0.1149	122.8	16.1	95.529
Totals				128.5	17.1	

(*R*)-*tert*-butyl 2-(4-fluorophenyl)-4,4-dimethylpentanoate (4g)

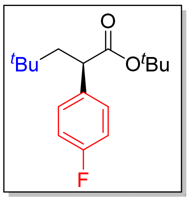

The reaction was performed following the General Procedure 1 with $NiBr_2$ (2.2 mg, 0.01 mmol) and **L6** (5.4 mg, 0.011 mmol), *tert*-butyl acrylate (15.0 μL, 0.1 mmol, 1.0 equiv.), 1-bromo-4-fluorobenzene (35 mg, 22.0 μL, 0.2 mmol, 2.0 equiv.), ^{t}BuBr (54.8 mg, 44.9 μL, 0.4 mmol, 4.0 equiv.), Cy_2NMe (58.5 mg, 64 μL, 0.3 mmol), HEH (75.9 mg, 0.3 mmol) and 4CzIPN (8.0 mg, 0.01 mmol) in DMA (3.0 mL). The crude product was purified by flash chromatography on silica gel (eluted with petroleum ether:EtOAc = 80:1) to give the product (23.8 mg, 85% yield, 90% ee) as a white solid. Melting point: 55.5 – 60.2 °C; R_f = 0.32 (petroleum ether:EtOAc = 80:1). $[\alpha]_D^{25}$ = –18.82 (*c* = 0.473, $CHCl_3$), ^{1}H NMR (400 MHz, $CDCl_3$) δ: 7.29 – 7.23 (m, 2H), 7.00 – 6.94 (m, 2H), 3.51 (dd, *J* = 9.2, 3.7 Hz, 1H), 2.23 (dd, *J* = 14.0, 9.2 Hz, 1H), 1.47 (dd, *J* = 14.0, 3.7 Hz, 1H), 1.37 (s, 9H), 0.90 (s, 9H). $^{13}C\{^1H\}$ NMR (101 MHz, $CDCl_3$) δ: 173.88, 162.55 (d, *J* = 245.8Hz), 137.4 (d, *J* = 3.3 Hz), 129.22 (d, *J* = 7.8 Hz), 115.33 (d, *J* = 21.5 Hz), 80.58, 48.58, 47.27, 31.12, 29.58, 27.90. ^{19}F NMR (375 MHz, $CDCl_3$) δ: -116.29 (s). The ee was determined by HPLC with a Daicel Chiralcel AD-H column (^{i}PrOH/hexanes = 0.2/99.8, 1.0 mL/min, 254 nm, major t_r = 4.244 min (*S*), minor t_r = 4.543 min (*R*)). FTIR (neat, cm^{-1}) ν: 3007, 2953, 2867, 1723, 1600, 1508, 1367, 1217, 1142, 1017, 872, 840, 767. HRMS: calcd for $C_{17}H_{26}FO_2$ $[M+H]^+$ 281.1917, found 281.1914.

4g. Racemic product

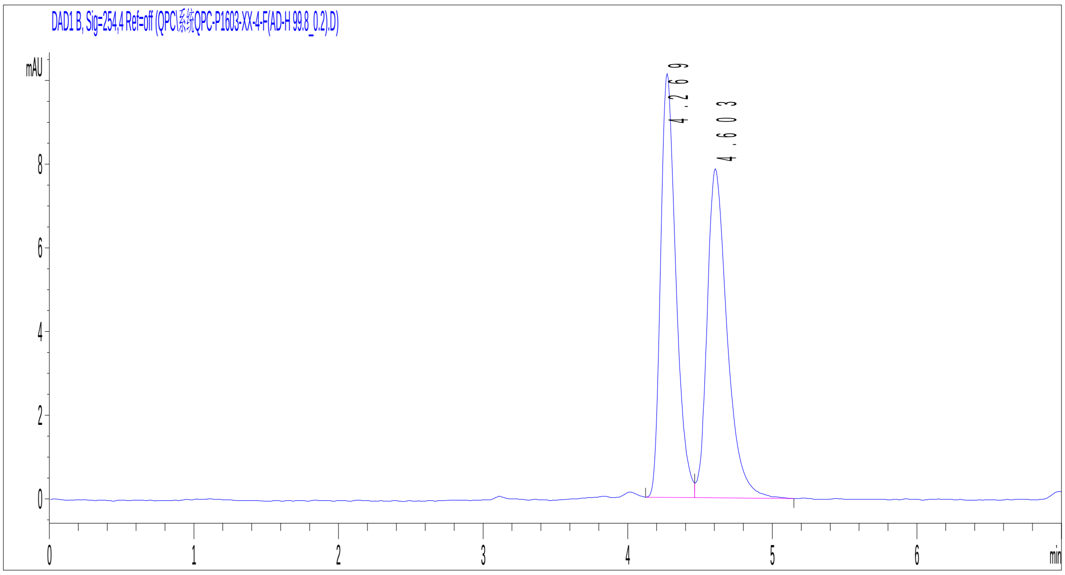

Peak	Ret Time	Type	Width	Area	Height	Area
#	[min]		[min]	mAU*s	[mAU]	%
1	4.269	BV	0.112	74.7	10.1	49.548
2	4.603	VB	0.1469	76	7.9	50.452
Totals				150.7	18.0	

4g. Enantioenriched product, 90% ee

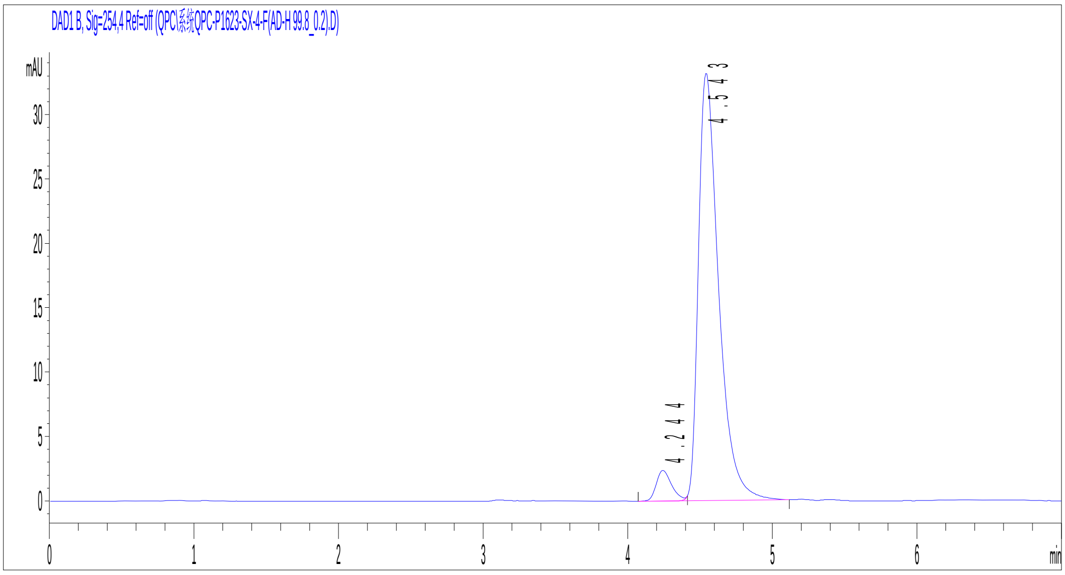

Peak	Ret Time	Type	Width	Area	Height	Area
#	[min]		[min]	mAU*s	[mAU]	%
1	4.244	BV E	0.1119	17.5	2.4	5.148
2	4.543	VB R	0.1474	322.6	33.2	94.852
Totals				340.1	35.6	

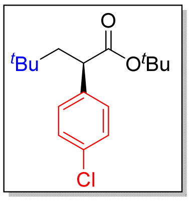

(*R*)-*tert*-butyl 2-(4-chlorophenyl)-4,4-dimethylpentanoate (4h)

The reaction was performed following the General Procedure 1 with $NiBr_2$ (2.2 mg, 0.01 mmol) and **L6** (5.4 mg, 0.011 mmol), *tert*-butyl acrylate (15.0 μL, 0.1 mmol, 1.0 equiv.), 1-bromo-4-chlorobenzene (38.3 mg, 0.2 mmol, 2.0 equiv.), ^{t}BuBr (54.8 mg, 44.9 μL, 0.4 mmol, 4.0 equiv.), Cy_2NMe (58.5 mg, 64 μL, 0.3 mmol), HEH (75.9 mg, 0.3 mmol) and 4CzIPN (8.0 mg, 0.01 mmol) in DMA (3.0 mL). The crude product was purified by flash chromatography on silica gel (eluted with petroleum ether:EtOAc = 80:1) to give the product (26.7 mg, 90% yield, 90% ee) as a white solid. Melting point: 84.1 – 84.9 °C; R_f = 0.32 (petroleum ether:EtOAc = 80:1). $[\alpha]_D^{25}$ = –20.55 (c = 0.613, $CHCl_3$), ^{1}H NMR (400 MHz, $CDCl_3$) δ: 7.29 – 7.21 (m, 4H), 3.50 (dd, J = 9.2, 3.7 Hz, 1H), 2.23 (dd, J = 14.0, 9.2 Hz, 1H), 1.46 (dd, J = 14.0, 3.7 Hz, 1H), 1.37 (s, 9H), 0.90 (s, 9H). ^{13}C{^{1}H} NMR (101 MHz, $CDCl_3$) δ: 173.62, 140.20, 132.60, 129.13, 128.68, 80.71, 48.77, 47.17, 31.15, 29.57, 27.90. The ee was determined by HPLC with a Daicel Chiralcel IA column (^{i}PrOH/hexanes = 0.2/99.8, 0.8 mL/min, 230 nm, major t_r = 5.274 min (*S*), minor t_r = 5.654 min (*R*)). FTIR (neat, cm^{-1}) ν: 3001, 2962, 2932, 2867, 1717, 1366, 1259, 1142, 1017, 842, 831, 799, 770. HRMS: calcd for $C_{17}H_{25}ClNaO_2$ $[M+Na]^+$ 319.1441, found 319.1436.

4h. Racemic product

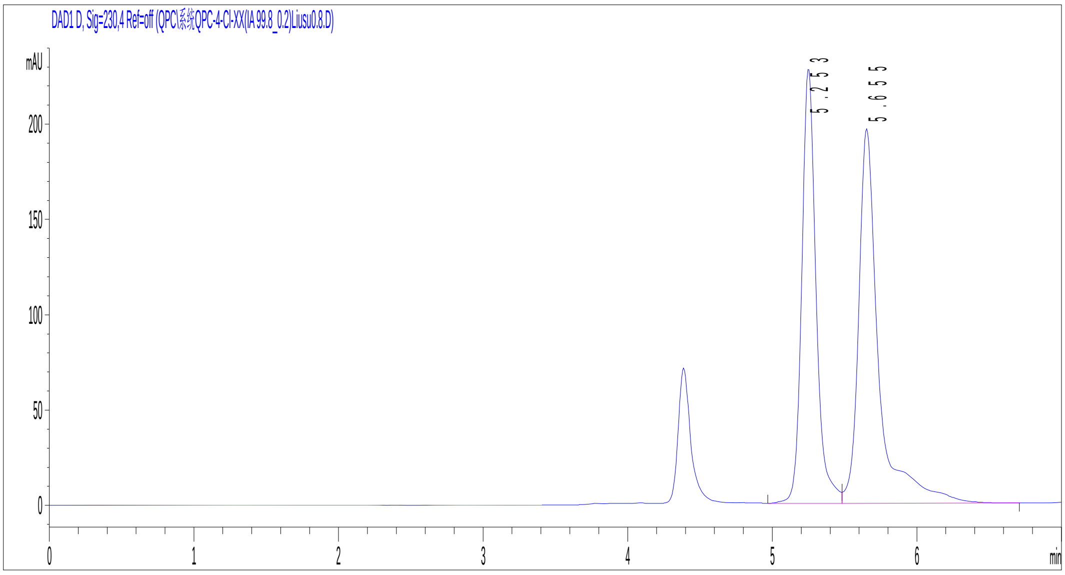

Peak	Ret Time	Type	Width	Area	Height	Area
#	[min]		[min]	mAU*s	[mAU]	%
1	5.253	BV	0.1041	1566.7	228.2	45.266
2	5.655	VB	0.1404	1894.4	196.6	54.734
Totals				3461.1	424.8	

4h. Enantioenriched product, 90% ee

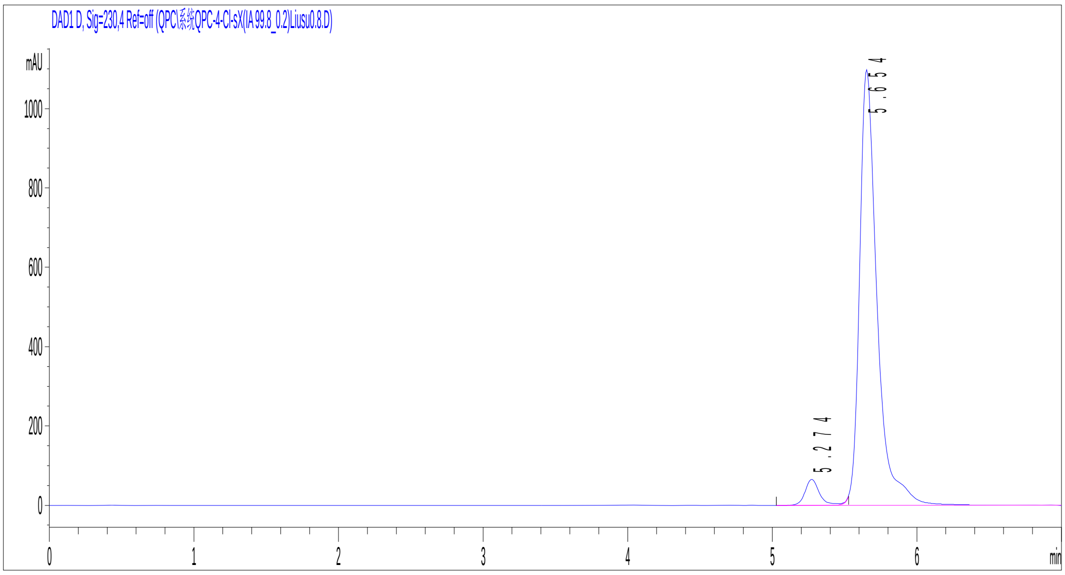

Peak	Ret Time	Type	Width	Area	Height	Area
#	[min]		[min]	mAU*s	[mAU]	%
1	5.274	BV E	0.1043	450.4	65.4	4.654
2	5.654	VB R	0.124	9225.6	1097.8	95.346
Totals				9676	1163.2	

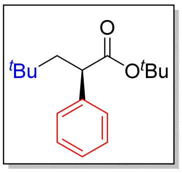

(*R*)-tert-butyl 4,4-dimethyl-2-phenylpentanoate (4i)

The reaction was performed following the General Procedure 1 with $NiBr_2$ (2.2 mg, 0.01 mmol) and **L6** (5.4 mg, 0.011 mmol), *tert*-butyl acrylate (15.0 μL, 0.1 mmol, 1.0 equiv.), Bromobenzene (31.4 mg, 21.1 μL, 0.2 mmol, 2.0 equiv.), ^{t}BuBr (54.8 mg, 44.9 μL, 0.4 mmol, 4.0 equiv.), Cy_2NMe (58.5 mg, 64 μL, 0.3 mmol), HEH (75.9 mg, 0.3 mmol) and 4CzIPN (8.0 mg, 0.01 mmol) in DMA (3.0 mL). The crude product was purified by flash chromatography on silica gel (eluted with petroleum ether:EtOAc = 80:1) to give the product (22.0 mg, 84% yield, 87% ee) as a white solid. Melting point: 34.0 – 40.1 °C; R_f = 0.4 (petroleum ether:EtOAc = 50:1), $[\alpha]_D^{25}$ = –25.26(c = 0.26, $CHCl_3$). ^{1}H NMR (400 MHz, $CDCl_3$) δ: 7.32 – 7.26 (m, 4H), 7.25 – 7.19 (m, 1H), 3.53 (dd, J = 9.4, 3.4 Hz, 1H), 2.27 (dd, J = 14.0, 9.4 Hz, 1H), 1.49 (dd, J = 14.0, 3.4 Hz, 1H), 1.37 (s, 9H), 0.91 (s, 9H). ^{13}C{^{1}H} NMR (101 MHz, $CDCl_3$) δ: 174.02, 141.77, 128.55, 127.74, 126.76, 80.42, 49.32, 47.27, 31.15, 29.58, 27.93. The ee was determined by HPLC with a Daicel Chiralcel AD-H column (^{i}PrOH/hexanes = 0.2/99.8, 1.0 mL/min, 230 nm, major t_r = 4.254 min (*S*), minor t_r =4.53 min (*R*)). FTIR (neat, cm^{-1}) ν: 2957, 2928, 2867, 1730, 1366, 1260, 1214, 1142, 1018, 803, 749, 697. HRMS: calcd for $C_{17}H_{27}O_2$ $[M+H]^+$ 263.2011, found 263.2001.

4i. Racemic product

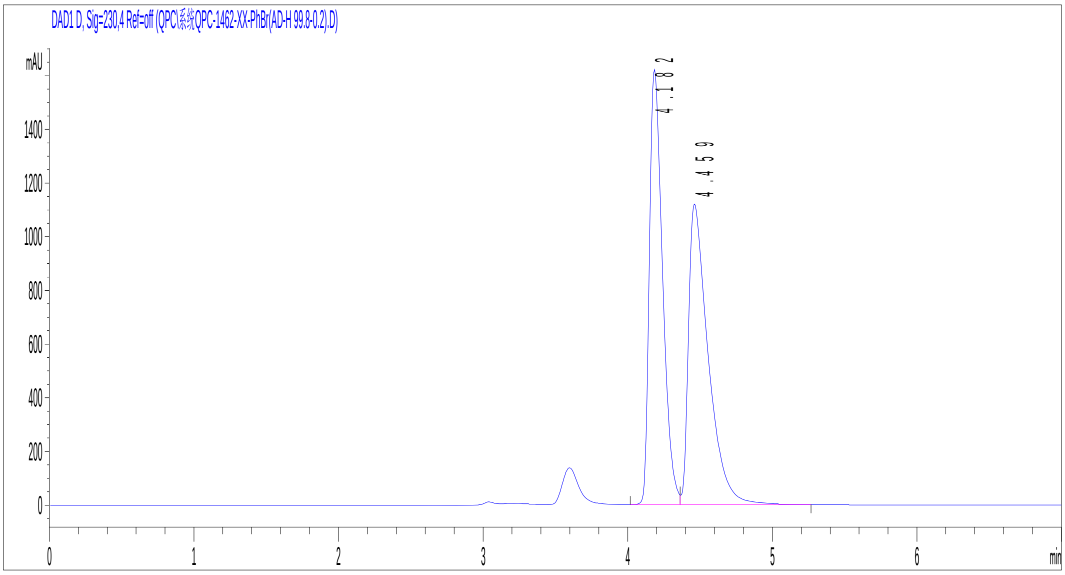

Peak	Ret Time	Type	Width	Area	Height	Area
#	[min]		[min]	mAU*s	[mAU]	%
1	4.182	BV	0.0983	10330.3	1621.4	49.358
2	4.459	VB	0.1404	10599.1	1120.3	50.642
Totals				20929.4	2741.7	

4i. Enantioenriched product, 87% ee

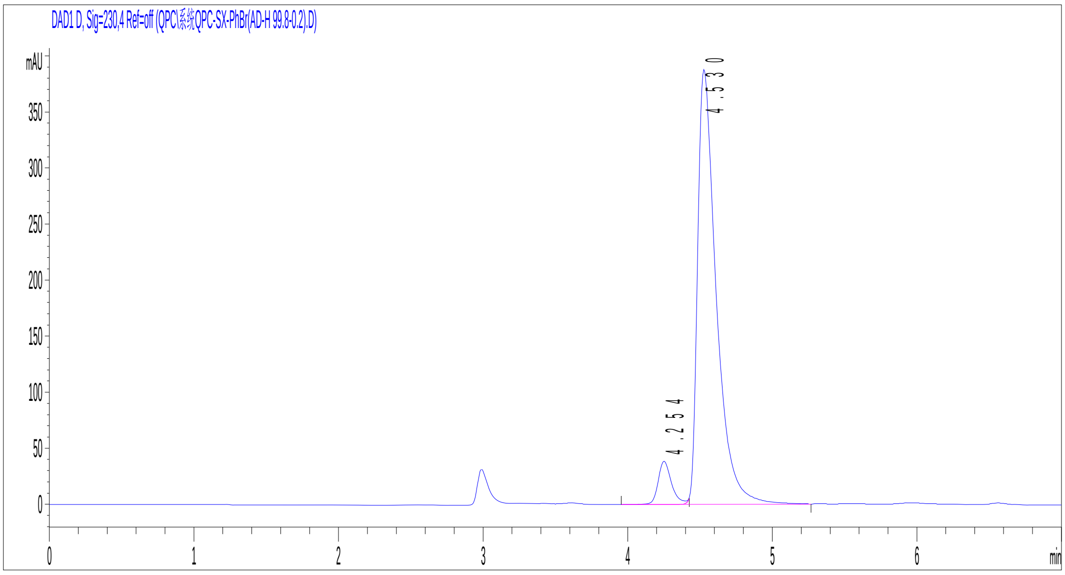

Peak	Ret Time	Type	Width	Area	Height	Area
#	[min]		[min]	mAU*s	[mAU]	%
1	4.254	BV E	0.0967	246.5	38.5	6.639
2	4.53	VB R	0.1323	3466.5	387.8	93.361
Totals				3713	426.3	

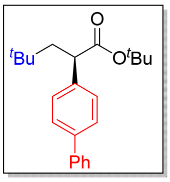

(*R*)-*tert*-butyl 2-([1,1'-biphenyl]-4-yl)-4,4-dimethylpentanoate (4j)

The reaction was performed following the General Procedure 1 with $NiBr_2$ (2.2 mg, 0.01 mmol) and **L6** (5.4 mg, 0.011 mmol), *tert*-butyl acrylate (15.0 μL, 0.1 mmol, 1.0 equiv.), 4-bromo-1,1'-biphenyl (46.6 mg, 0.2 mmol, 2.0 equiv.), ^{t}BuBr (54.8 mg, 44.9 μL, 0.4 mmol, 4.0 equiv.), Cy_2NMe (58.5 mg, 64 μL, 0.3 mmol), HEH (75.9 mg, 0.3 mmol) and 4CzIPN (8.0 mg, 0.01 mmol) in DMA (3.0 mL). The crude product was purified by flash chromatography on silica gel (eluted with petroleum ether:EtOAc = 80:1) to give the product (28.1 mg, 83% yield, 86% ee) as a white solid. Melting point: 76.7 – 78.3 ^{o}C; R_f = 0.46 (petroleum ether:EtOAc = 50:1). $[\alpha]_D^{25}$ = –13.93 (*c* = 0.493, $CHCl_3$). *lit.* $[\alpha]_D^{25}$ = –13.9110 (*c* = 0.6333, $CHCl_3$, 85% ee). The spectroscopic data for this product match the literature data[8]. The ee was determined by HPLC with a Daicel Chiralcel AD-H column (^{i}PrOH/hexanes = 0.2/99.8, 1.0 mL/min, 254 nm, major t_r = 6.824 min (*S*), minor t_r = 8.133 min (*R*)). FTIR (neat, cm^{-1}) ν: 2955, 2931, 1728, 1486, 1477, 1366, 1259, 1162, 1142, 878, 756, 697.

4j. Racemic product

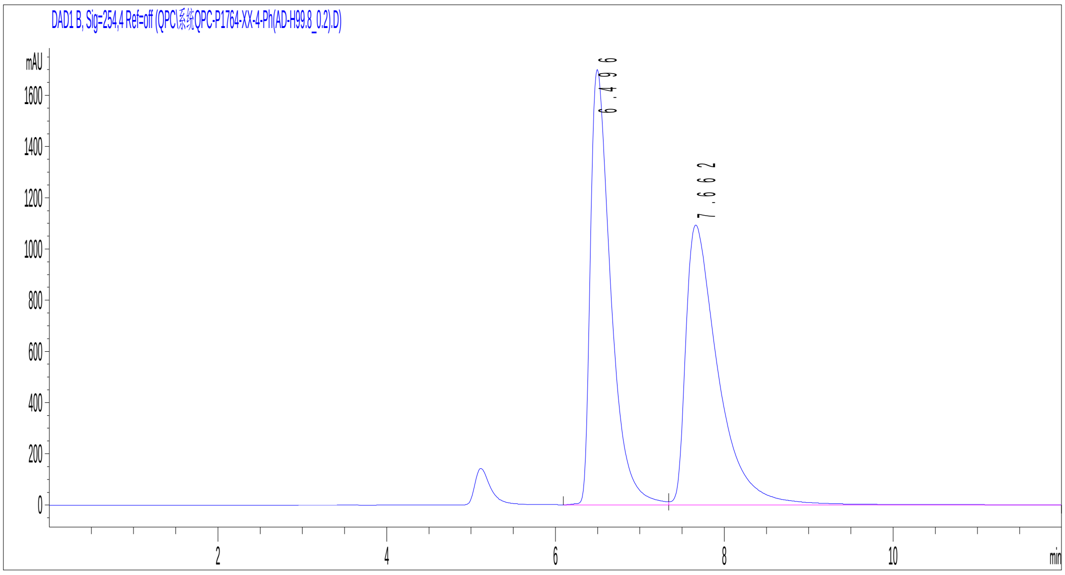

Peak	Ret Time	Type	Width	Area	Height	Area
#	[min]		[min]	mAU*s	[mAU]	%
1	6.496	VV	0.2552	28733	1700.1	49.442
2	7.662	VBA	0.3976	29382	1093.1	50.558
Totals				58115	2793.2	

4j. enantioenriched, 85% ee

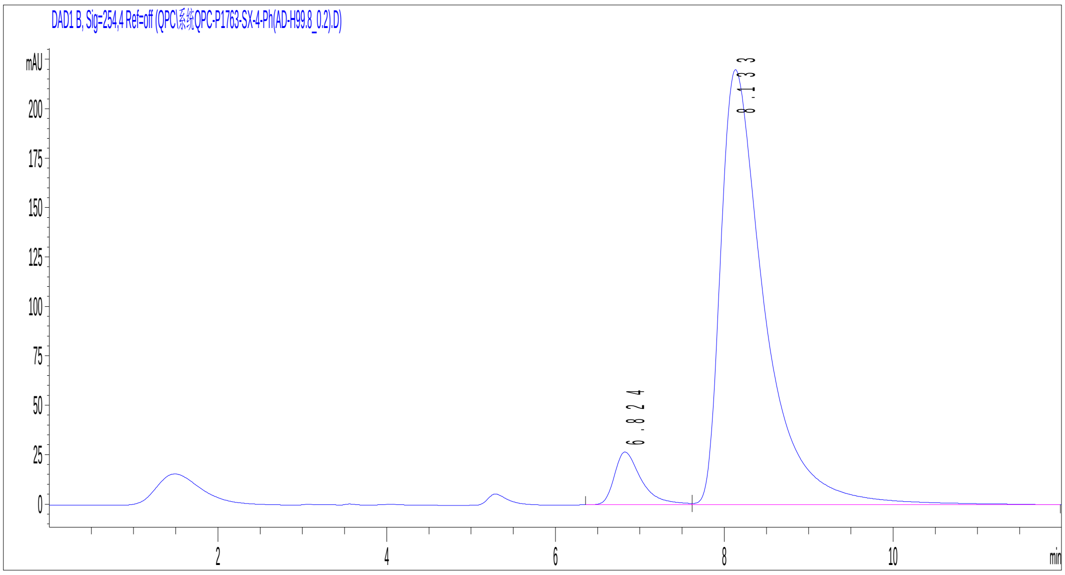

Peak	Ret Time	Type	Width	Area	Height	Area
#	[min]		[min]	mAU*s	[mAU]	%
1	6.824	BV	0.3358	594	26.7	6.929
2	8.133	VBA	0.5391	7978.4	220	93.071
Totals				8572.4	246.7	

(*R*)-*tert*-butyl 2-(4-(tert-butyl)phenyl)-4,4-dimethylpentanoate (4k)

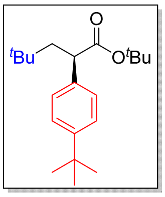

The reaction was performed following the General Procedure 1 with $NiBr_2$ (2.2 mg, 0.01 mmol) and **L6** (5.4 mg, 0.011 mmol), *tert*-butyl acrylate (15.0 μL, 0.1 mmol, 1.0 equiv.), 1-bromo-4-(*tert*-butyl)benzene (42.6 mg, 34.7 μL, 0.2 mmol, 2.0 equiv.), ^{t}BuBr (54.8 mg, 44.9 μL, 0.4 mmol, 4.0 equiv.), Cy_2NMe (58.5 mg, 64 μL, 0.3 mmol), HEH (75.9 mg, 0.3 mmol) and 4CzIPN (8.0 mg, 0.01 mmol) in DMA (3.0 mL). The crude product was purified by flash chromatography on silica gel (eluted with petroleum ether:EtOAc = 80:1) to give the product (22.9 mg, 72% yield, 86% ee) as a white solid. Melting point: 43.3 – 44.2 °C; R_f = 0.31 (petroleum ether:EtOAc = 50:1). $[\alpha]_D^{25}$ = –16.09 (*c* = 0.486, $CHCl_3$). ^{1}H NMR (400 MHz, $CDCl_3$) δ: 7.30 (d, *J* = 8.4 Hz, 2H), 7.21 (d, *J* = 8.5 Hz, 2H), 3.51 (dd, *J* = 9.9, 2.9 Hz, 1H), 2.27 (dd, *J* = 13.9, 9.9 Hz, 1H), 1.46 (dd, *J* = 14.0, 2.9 Hz, 1H), 1.39 (s, 9H), 1.30 (s, 9H), 0.91 (s, 9H). $^{13}C\{^1H\}$ NMR (101 MHz, $CDCl_3$) δ: 174.13, 149.35, 138.55, 127.10, 125.32, 80.23, 48.63, 47.40, 34.35, 31.34, 31.02, 29.45, 27.85 . The ee was determined by HPLC with a Daicel Chiralcel IA column (^{i}PrOH/hexanes = 0.1/100, 1.0 mL/min, 230 nm, major t_r = 6.373 min (*S*), minor t_r = 6.775 min (*R*)). FTIR (neat, cm^{-1}) ν: 3003, 2960, 2868, 1730, 1477, 1366, 1260, 1143, 1019, 845, 803, 571. HRMS: calcd for $C_{21}H_{35}O_2$ $[M+H]^+$ 319.2637, found 319.2637.

4k. Racemic product

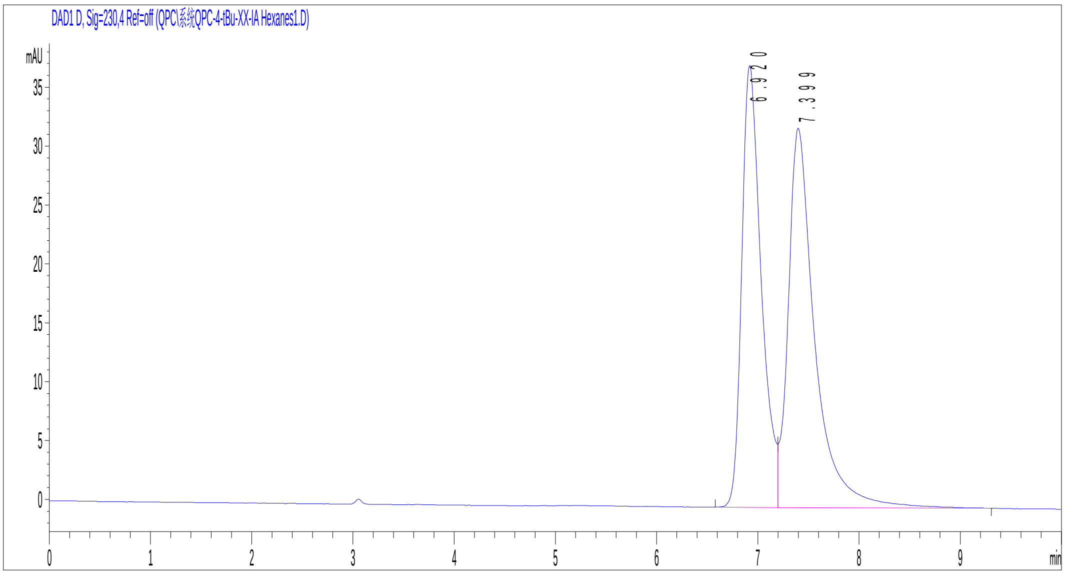

Peak	Ret Time	Type	Width	Area	Height	Area
#	[min]		[min]	mAU*s	[mAU]	%
1	6.92	BV	0.2024	502.9	37.5	45.972
2	7.399	VB	0.2679	591	32.2	54.028
Totals				1093.9	69.7	

4k. Enantioenriched, 86% ee

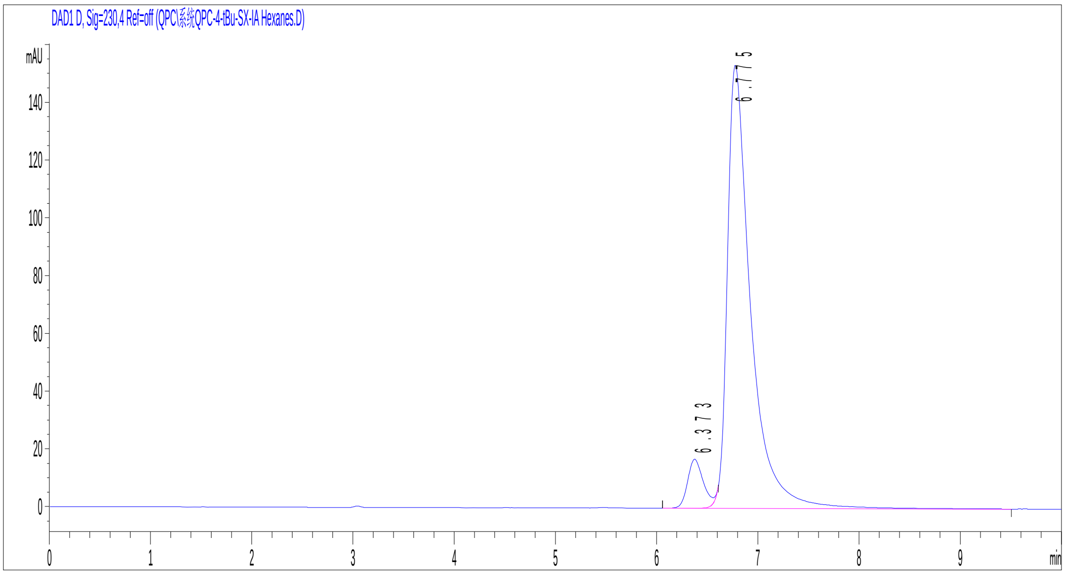

Peak	Ret Time	Type	Width	Area	Height	Area
#	[min]		[min]	mAU*s	[mAU]	%
1	6.373	BV E	0.1645	184	17	6.944
2	6.775	VB R	0.2356	2466.5	153.4	93.056
Totals				2650.5	170.4	

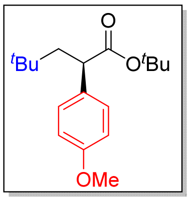

(*R*)-*tert*-butyl 2-(4-methoxyphenyl)-4,4-dimethylpentanoate (4l)

The reaction was performed following the General Procedure 1 with $NiBr_2$ (2.2 mg, 0.01 mmol) and **L6** (5.4 mg, 0.011 mmol), *tert*-butyl acrylate (15.0 μL, 0.1 mmol, 1.0 equiv.), 1-bromo-4-methoxybenzene (37.4 mg, 0.2 mmol, 2.0 equiv.), ^{t}BuBr (54.8 mg, 44.9 μL, 0.4 mmol, 4.0 equiv.), Cy_2NMe (58.5 mg, 64 μL, 0.3 mmol), HEH (75.9 mg, 0.3 mmol) and 4CzIPN (8.0 mg, 0.01 mmol) in DMA (3.0 mL). The crude product was purified by flash chromatography on silica gel (eluted with petroleum ether:EtOAc = 50:1) to give the product (20.8 mg, 71% yield, 85% ee) as a white solid. Melting point: 53.6 – 62.8 °C; R_f = 0.42 (petroleum ether:EtOAc = 20:1). $[\alpha]_D^{25}$ = –21.54 (c = 0.621, $CHCl_3$), ^{1}H NMR (400 MHz, $CDCl_3$) δ: 7.22 (d, J = 8.7 Hz, 2H), 6.83 (d, J = 8.7 Hz, 2H), 3.79 (s, 3H), 3.47 (dd, J = 9.3, 3.5 Hz, 1H), 2.23 (dd, J = 13.9, 9.3 Hz, 1H), 1.47 (dd, J = 14.0, 3.6 Hz, 1H), 1.37 (s, 9H), 0.90 (s, 9H). $^{13}C\{^1H\}$ NMR (101 MHz, $CDCl_3$) δ: 174.20, 158.30, 133.76, 128.60, 113.76, 80.21, 55.19, 48.31, 47.19, 30.98, 29.49, 27.83. The ee was determined by HPLC with a Daicel Chiralcel IA column (^{i}PrOH/hexanes = 0.5/99.5, 1.0 mL/min, 250 nm, major t_r = 4.634 min (S), minor t_r = 4.954 min (R)). FTIR (neat, cm^{-1}) ν: 2925, 2857, 1689, 1569, 1346, 1248, 1129, 1024, 858, 836, 752, 691. HRMS: calcd for $C_{18}H_{29}O_3$ $[M+H]^+$ 293.2117, found 293.2109.

4l. Racemic product

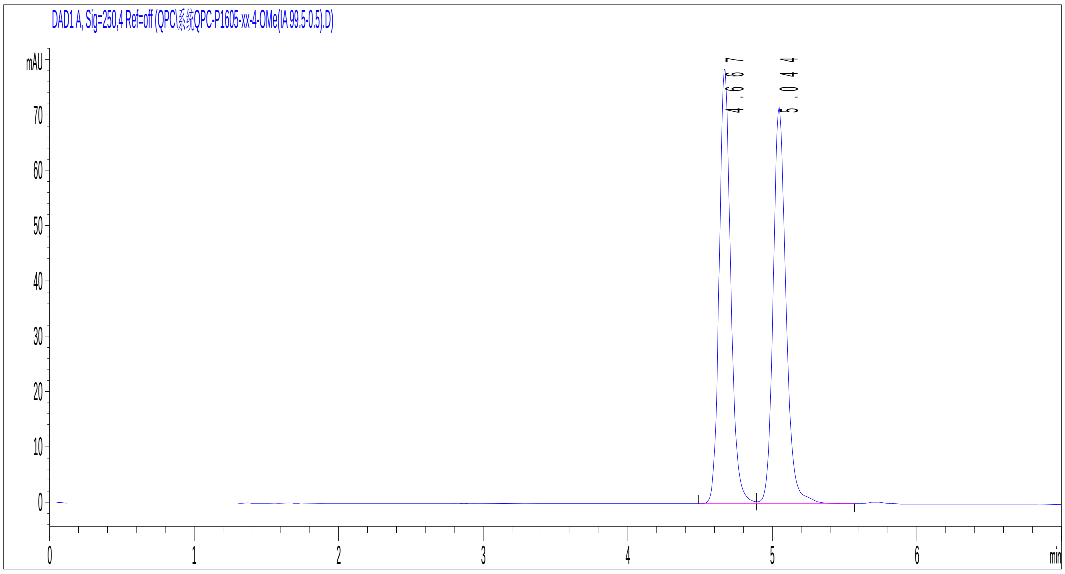

Peak	Ret Time	Type	Width	Area	Height	Area
#	[min]		[min]	mAU*s	[mAU]	%
1	4.66	BV	0.0847	437.2	78.8	49.583
2	5.044	VB	0.0943	444.5	71.7	50.417
Totals				881.7	150.5	

4l. Enantioenriched product, 85% ee

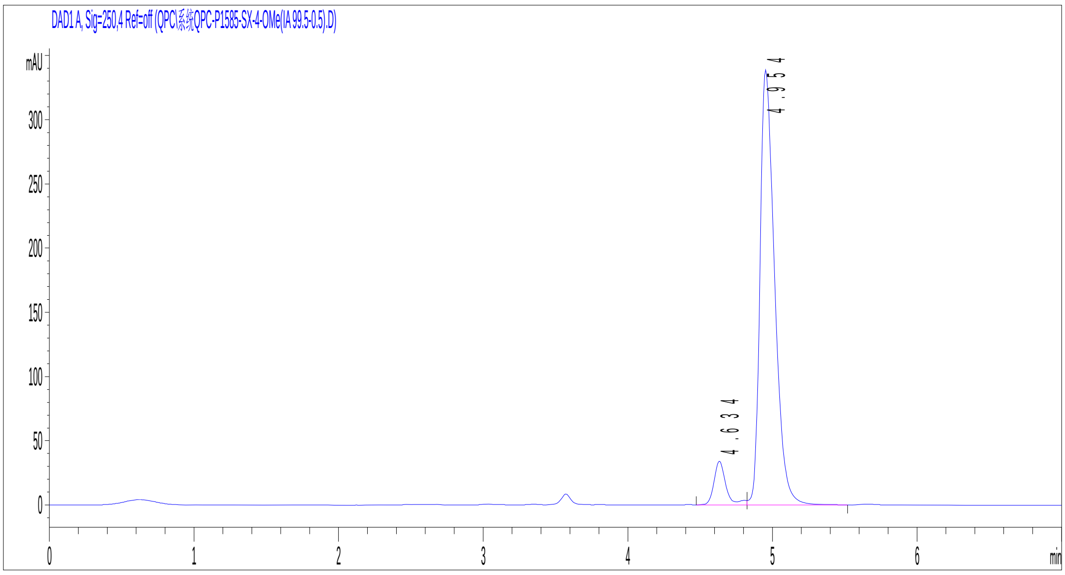

Peak	Ret Time	Type	Width	Area	Height	Area
#	[min]		[min]	mAU*s	[mAU]	%
1	4.634	BV R	0.0807	194.6	34	7.598
2	4.954	VB	0.1075	2366.8	338.7	92.402
Totals				2561.4	372.7	

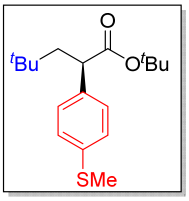

(*R*)-*tert*-butyl 4,4-dimethyl-2-(4-(methylthio)phenyl)pentanoate (4m)

The reaction was performed following the General Procedure 1 with $NiBr_2$ (2.2 mg, 0.01 mmol) and **L6** (5.4 mg, 0.011 mmol), *tert*-butyl acrylate (15.0 μL, 0.1 mmol, 1.0 equiv.), (4-bromophenyl)(methyl)sulfane (40.6 mg, 0.2 mmol, 2.0 equiv.), ^{t}BuBr (54.8 mg, 44.9 μL, 0.4 mmol, 4.0 equiv.), Cy_2NMe (58.5 mg, 64 μL, 0.3 mmol), HEH (75.9 mg, 0.3 mmol) and 4CzIPN (8.0 mg, 0.01 mmol) in DMA (3.0 mL). The crude product was purified by flash chromatography on silica gel (eluted with petroleum ether:EtOAc = 50:1) to give the product (25.0 mg, 81% yield, 86% ee) as a white solid. Melting point: 70.2 – 70.8 °C; R_f = 0.51 (petroleum ether:EtOAc = 20:1). $[\alpha]_D^{25}$ = –26.38 (c = 0.733, $CHCl_3$). *lit.* $[\alpha]_D^{25}$ = –20.5765 (c = 0.2600, $CHCl_3$, 83% ee). The spectroscopic data for this product match the literature data[8]. The ee was determined by HPLC with a Daicel Chiralcel IA column (^{i}PrOH/hexanes = 0.5/99.5, 1.0 mL/min, 250 nm, major t_r = 4.333 min (*S*), minor t_r = 4.552 min (*R*)). FTIR (neat, cm^{-1}) ν: 3007, 2960, 2860, 1726, 1365, 1257, 1143, 1017, 958, 842, 826, 770.

4m. Racemic product

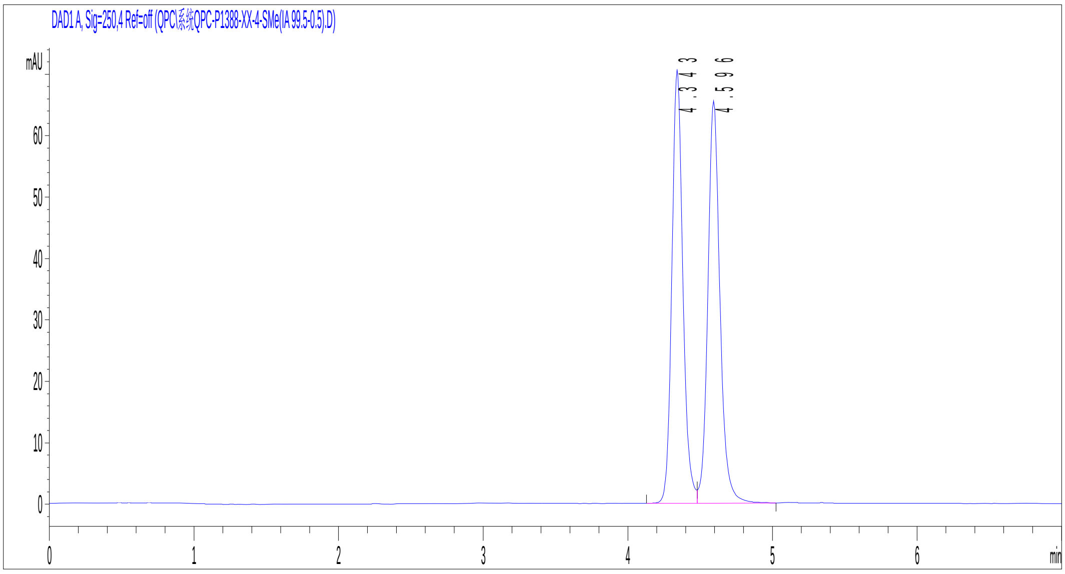

Peak	Ret Time	Type	Width	Area	Height	Area
#	[min]		[min]	mAU*s	[mAU]	%
1	4.343	BV	0.0795	372.2	70.6	49.396
2	4.596	VB	0.0899	381.3	65.5	50.604
Totals				753.5	136.1	

4m. Enantioenriched product, 86% ee

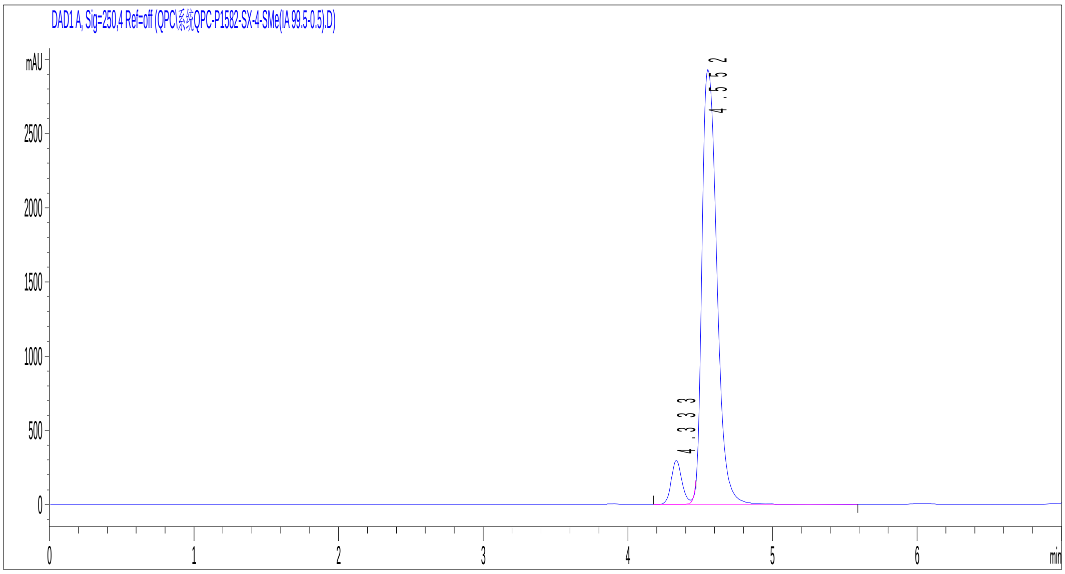

Peak	Ret Time	Type	Width	Area	Height	Area
#	[min]		[min]	mAU*s	[mAU]	%
1	4.333	BV E	0.0782	1541.4	298.5	6.794
2	4.552	VV R	0.1142	21144.8	2930	93.206
Totals				22686.2	3228.5	

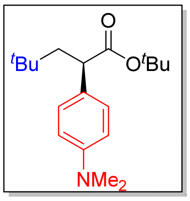

(*R*)-*tert*-butyl 2-(4-(dimethylamino)phenyl)-4,4-dimethylpentanoate (4n)

The reaction was performed following the General Procedure 1 with $NiBr_2$ (2.2 mg, 0.01 mmol) and **L6** (5.4 mg, 0.011 mmol), *tert*-butyl acrylate (15.0 μL, 0.1 mmol, 1.0 equiv.), 4-bromo-*N,N*-dimethylaniline (40.0 mg, 0.2 mmol, 2.0 equiv.), ^{t}BuBr (54.8 mg, 44.9 μL, 0.4 mmol, 4.0 equiv.), Cy_2NMe (58.5 mg, 64 μL, 0.3 mmol), HEH (75.9 mg, 0.3 mmol) and 4CzIPN (8.0 mg, 0.01 mmol) in DMA (3.0 mL). The crude product was purified by flash chromatography on silica gel (eluted with petroleum ether:EtOAc = 80:1) to give the product (18.0 mg, 59% yield, 83% ee) as a white solid. Melting point: 68.5 – 70.0 ^{o}C; R_f = 0.31 (petroleum ether:EtOAc = 50:1). $[\alpha]_D^{25}$ = –50.06 (*c* = 0.483, $CHCl_3$). ^{1}H NMR (400 MHz, $CDCl_3$) δ: 7.16 (d, *J* = 8.8 Hz, 2H), 6.67 (d, *J* = 8.8 Hz, 2H), 3.43 (dd, *J* = 9.5, 3.3 Hz, 1H), 2.92 (s, 6H), 2.22 (dd, *J* = 13.9, 9.5 Hz, 1H), 1.45 (dd, *J* = 13.9, 3.4 Hz, 1H), 1.37 (s, 9H), 0.90 (s, 9H). $^{13}C\{^1H\}$ NMR (101 MHz, $CDCl_3$) δ: 174.46, 149.38, 129.62, 128.17, 112.65, 79.94, 48.09, 47.26, 40.65, 30.94, 29.49, 27.85. The ee was determined by HPLC with a Daicel Chiralcel IC-3 column (^{i}PrOH/hexanes = 0.5/99.5, 1.0 mL/min, 254 nm, major t_r = 6.233 min (*S*), minor t_r = 6.642 min (*R*)). FTIR (neat, cm^{-1}) ν: 3008, 2959, 2806, 1715, 1615, 1521, 1366, 1142, 1068, 1019, 813. HRMS: calcd for $C_{19}H_{32}NO_2$ $[M+H]^+$ 306.2433, found 306.2431.

4n. Racemic product

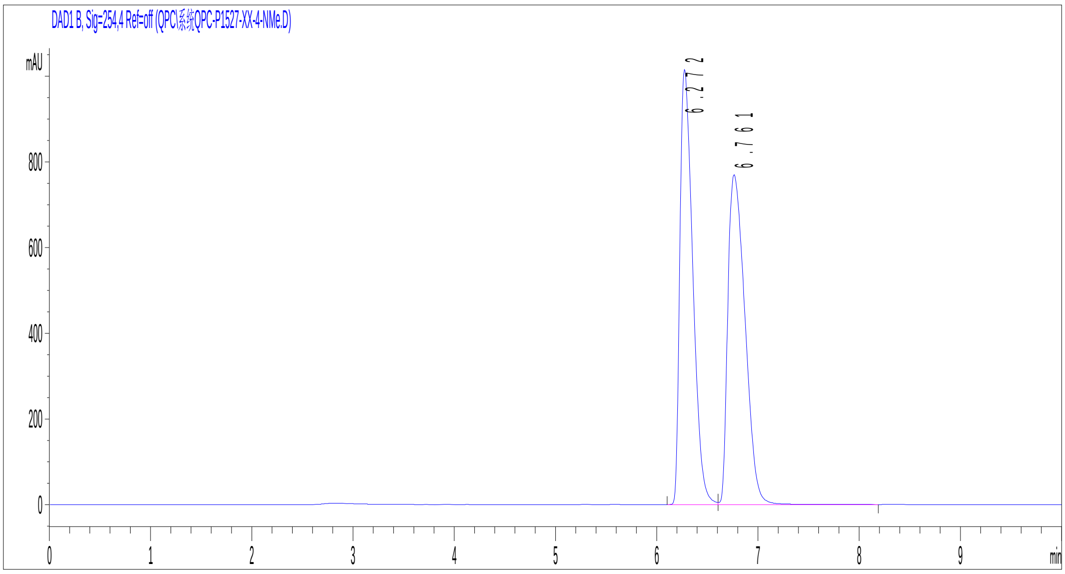

Peak	Ret Time	Type	Width	Area	Height	Area
#	[min]		[min]	mAU*s	[mAU]	%
1	6.272	BV	0.1395	9536.9	1074.6	49.677
2	6.761	VB	0.1937	9660.9	818.5	50.323
Totals				19197.8	1893.1	

4n. Enantioenriched product, 83% ee

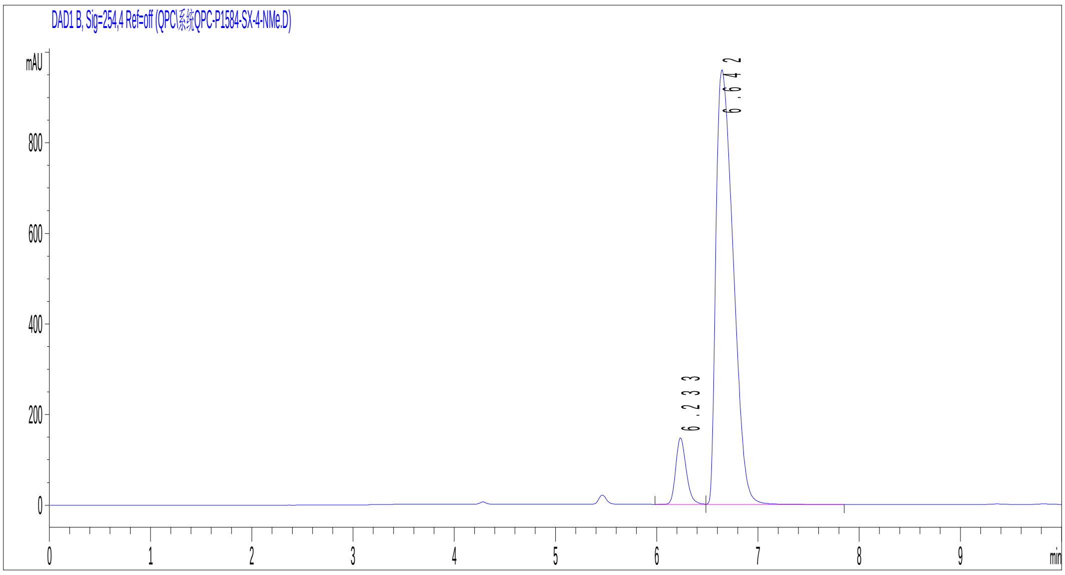

Peak	Ret Time	Type	Width	Area	Height	Area
#	[min]		[min]	mAU*s	[mAU]	%
1	6.233	BV	0.1103	1061	146.9	8.516
2	6.642	VB	0.1926	11397.3	959.5	91.484
Totals				12458.3	1106.4	

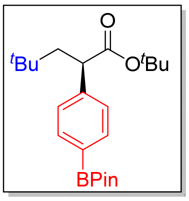

(*R*)-*tert*-butyl 4,4-dimethyl-2-(4-(4,4,5,5-tetramethyl-1,3,2-dioxaborolan-2-yl)phenyl)pentanoate (4o)

The reaction was performed following the General Procedure 1 with $NiBr_2$ (2.2 mg, 0.01 mmol) and **L6** (5.4 mg, 0.011 mmol), *tert*-butyl acrylate (15.0 μL, 0.1 mmol, 1.0 equiv.), 2-(4-bromophenyl)-4,4,5,5-tetramethyl-1,3,2-dioxaborolane (56.6 mg, 0.2 mmol, 2.0 equiv.), ^{t}BuBr (54.8 mg, 44.9 μL, 0.4 mmol, 4.0 equiv.), Cy_2NMe (58.5 mg, 64 μL, 0.3 mmol), HEH (75.9 mg, 0.3 mmol) and 4CzIPN (8.0 mg, 0.01 mmol) in DMA (3.0 mL). The crude product was purified by flash chromatography on silica gel (eluted with petroleum ether:EtOAc = 50:1) to give the product (27.6 mg, 71% yield, 86% ee) as a white solid. Melting point: 115.2 - 117.4 °C; R_f = 0.38 (petroleum ther:EtOAc = 20:1). $[\alpha]_D^{25}$ = –19.71 (*c* = 0.837, $CHCl_3$). ^{1}H NMR (400 MHz, $CDCl_3$) δ: 7.74 (d, *J* = 8.1 Hz, 2H), 7.31 (d, *J* = 8.1 Hz, 2H), 3.54 (dd, *J* = 9.2, 3.5 Hz, 1H), 2.26 (dd, *J* = 14.0, 9.3 Hz, 1H), 1.48 (dd, *J* = 14.0, 3.6 Hz, 1H), 1.35 (s, 9H), 1.34 (s, 12H), 0.90 (s, 9H). $^{13}C\{^1H\}$ NMR (101 MHz, $CDCl_3$) δ: 173.72, 144.96, 135.08, 127.21, 83.83, 80.51, 49.56, 47.09, 31.17, 29.59, 27.90, 24.96. The ee was determined by HPLC with a Daicel Chiralcel AD-H column (^{i}PrOH/hexanes = 0.5/99.5, 1.0 mL/min, 230 nm, major t_r = 6.233 min (*S*), minor t_r = 6.642 min (*R*)). FTIR (neat, cm^{-1}) ν: 2976, 2958, 2868, 1730, 1610, 1362, 1143, 1091, 963, 860, 844, 658. HRMS: calcd for $C_{23}H_{38}BO_4$ $[M+H]^+$ 388.2899, found 388.2906.

4o. Racemic product

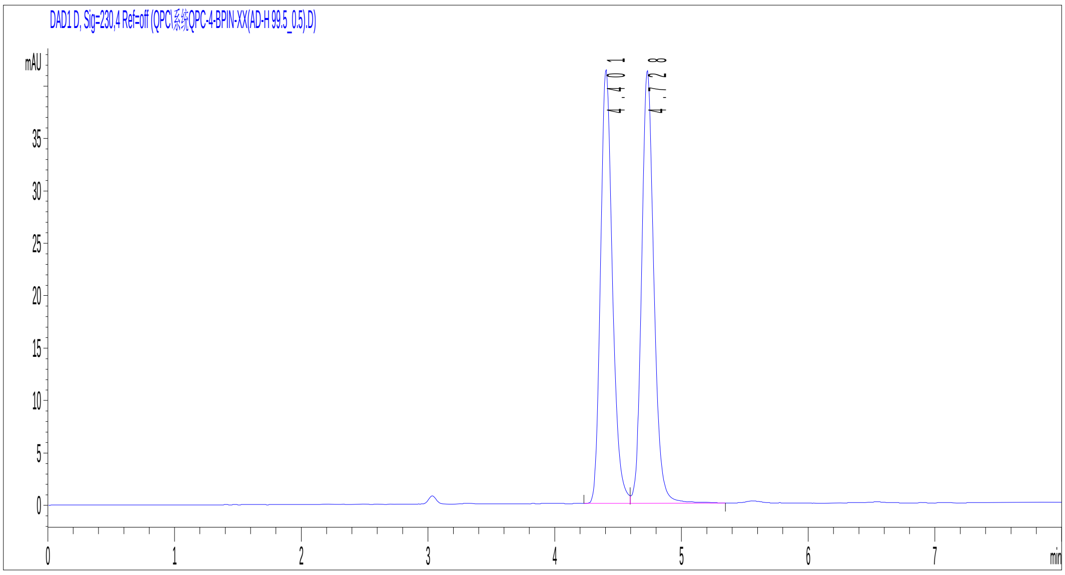

Peak	Ret Time	Type	Width	Area	Height	Area
#	[min]		[min]	mAU*s	[mAU]	%
1	4.401	BV	0.1001	270.2	41.4	49.323
2	4.728	VB	0.1024	277.6	41.3	50.677
Totals				547.8	82.7	

4o. Enantioenriched product, 86% ee

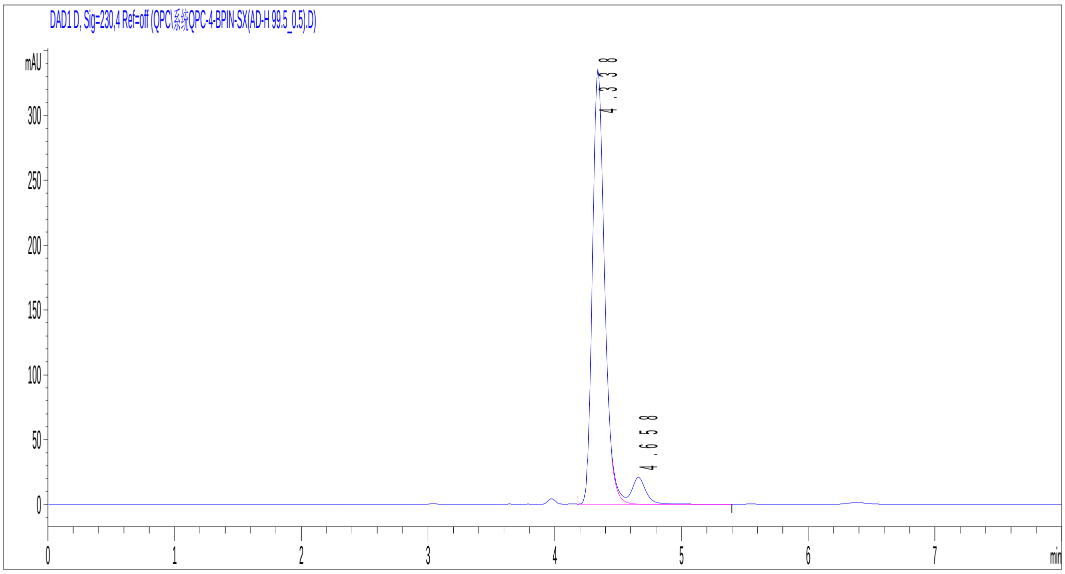

Peak	Ret Time	Type	Width	Area	Height	Area
#	[min]		[min]	mAU*s	[mAU]	%
1	4.338	BV R	0.1039	2236.6	334.9	92.938
2	4.658	VB E	0.1194	170	20.8	7.062
Totals				2406.6	355.7	

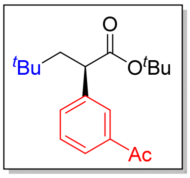

(*R*)-*tert*-butyl2-(3-acetylphenyl)-4,4-dimethylpentanoate (4p)

The reaction was performed following the General Procedure 1 with $NiBr_2$ (2.2 mg, 0.01 mmol) and **L6** (5.4 mg, 0.011 mmol), *tert*-butyl acrylate (15.0 μL, 0.1 mmol, 1.0 equiv.), 1-(3-bromophenyl)ethanone (39.8 mg, 26.5 μL, 0.2 mmol, 2.0 equiv.), ^{t}BuBr (54.8 mg, 44.9 μL, 0.4 mmol, 4.0 equiv.), Cy_2NMe (58.5 mg, 64 μL, 0.3 mmol), HEH (75.9 mg, 0.3 mmol) and 4CzIPN (8.0 mg, 0.01 mmol) in DMA (3.0 mL). The crude product was purified by flash chromatography on silica gel (eluted with petroleum ether:EtOAc = 50:1) to give the product (18.3 mg, 60% yield, 88% ee) as a white solid. Melting point: 57.0 – 60.5 °C; R_f = 0.48 (petroleum ether:EtOAc = 20:1). $[\alpha]_D^{25}$ = –18.09 (*c* = 0.853, $CHCl_3$). *lit.* $[\alpha]_D^{25}$ = –19.8776 (*c* = 0.1967, $CHCl_3$, 92% ee). The spectroscopic data for this product match the literature data[8]. The ee was determined by HPLC with a Daicel Chiralcel IC-3 column (^{i}PrOH/hexanes = 2/98, 1.0 mL/min, 230 nm, major t_r = 12.251 min (*S*), minor t_r = 12.829 min (*R*)). FTIR (neat, cm^{-1}) ν: 3002, 2960, 2868, 1730, 1688, 1367, 1269, 1144, 844, 801, 761, 693.

4p. Racemic product

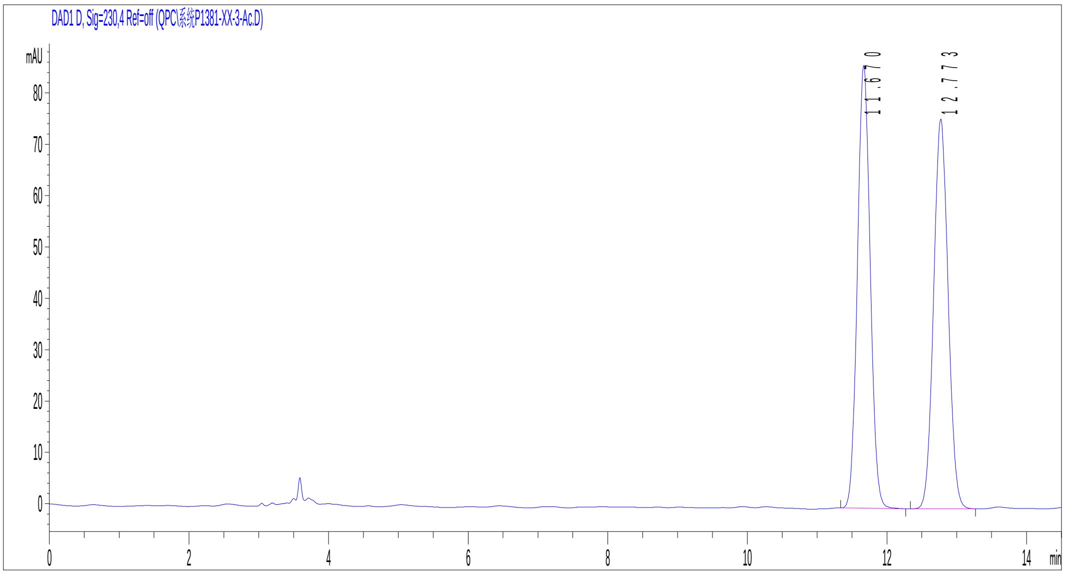

Peak	Ret Time	Type	Width	Area	Height	Area
#	[min]		[min]	mAU*s	[mAU]	%
1	11.67	BB	0.1987	1099	86.2	49.989
2	12.773	BB	0.2249	1099.5	75.9	50.011
Totals				2198.5	162.1	

4p. Enantioenriched product, 88% ee

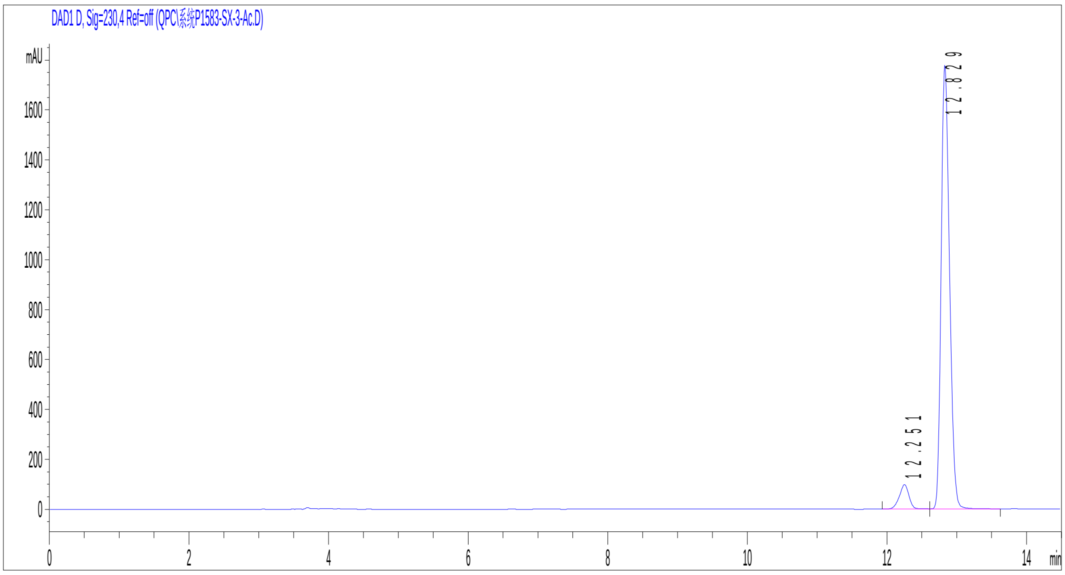

Peak	Ret Time	Type	Width	Area	Height	Area
#	[min]		[min]	mAU*s	[mAU]	%
1	12.251	BV	0.1452	954.7	98.4	6.074
2	12.829	VB	0.1289	14762.9	1777.8	93.926
Totals				15717.6	1876.2	

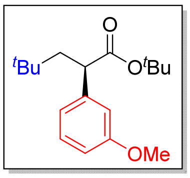

(*R*)-*tert*-butyl 2-(3-methoxyphenyl)-4,4-dimethylpentanoate (4q)

The reaction was performed following the General Procedure 1 with $NiBr_2$ (2.2 mg, 0.01 mmol) and **L6** (5.4 mg, 0.011 mmol), *tert*-butyl acrylate (15.0 μL, 0.1 mmol, 1.0 equiv.), 1-bromo-3-methoxybenzene (37.4 mg, 25.1 μL，0.2 mmol, 2.0 equiv.), ^{t}BuBr (54.8 mg, 44.9 μL, 0.4 mmol, 4.0 equiv.), Cy_2NMe (58.5 mg, 64 μL, 0.3 mmol), HEH (75.9 mg, 0.3 mmol) and 4CzIPN (8.0 mg, 0.01 mmol) in DMA (3.0 mL). The crude product was purified by flash chromatography on silica gel (eluted with petroleum ether:EtOAc = 50:1) to give the product (22.5 mg, 77% yield, 88% ee) as a white solid. Melting point: 47.4 – 48.6 °C; R_f = 0.43 (petroleum ether:EtOAc = 20:1). $[\alpha]_D^{25}$ = –19.62 (*c* = 0.530, $CHCl_3$). ^{1}H NMR (400 MHz, $CDCl_3$) δ: 7.20 (t, *J* = 7.9 Hz, 1H), 6.89 (d, *J* = 7.7 Hz, 1H), 6.87 – 6.84 (m, 1H), 6.76 (m, 1H), 3.80 (s, 3H), 3.50 (dd, *J* = 9.5, 3.3 Hz, 1H), 2.26 (dd, *J* = 14.0, 9.5 Hz, 1H), 1.48 (dd, *J* = 14.0, 3.3 Hz, 1H), 1.38 (s, 9H), 0.91 (s, 9H). $^{13}C\{^1H\}$ NMR (101 MHz, $CDCl_3$) δ: 173.85, 159.67, 143.31, 129.48, 120.18, 113.31, 112.17, 80.46, 55.26, 49.34, 47.30, 31.14, 29.56, 27.94. The ee was determined by HPLC with a Daicel Chiralcel IC-3 column (^{i}PrOH/hexanes = 0.5/99.5, 1.0 mL/min, 254 nm, major t_r = 4.476 min (*S*), minor t_r = 4.745 min (*R*)). FTIR (neat, cm^{-1}) ν: 2955, 2867, 1729, 1599, 1366, 1261, 1140, 1044, 872, 846, 761, 694. HRMS: calcd for $C_{18}H_{29}O_3$ $[M+H]^+$ 293.2117, found 293.2120.

4q. Racemic product

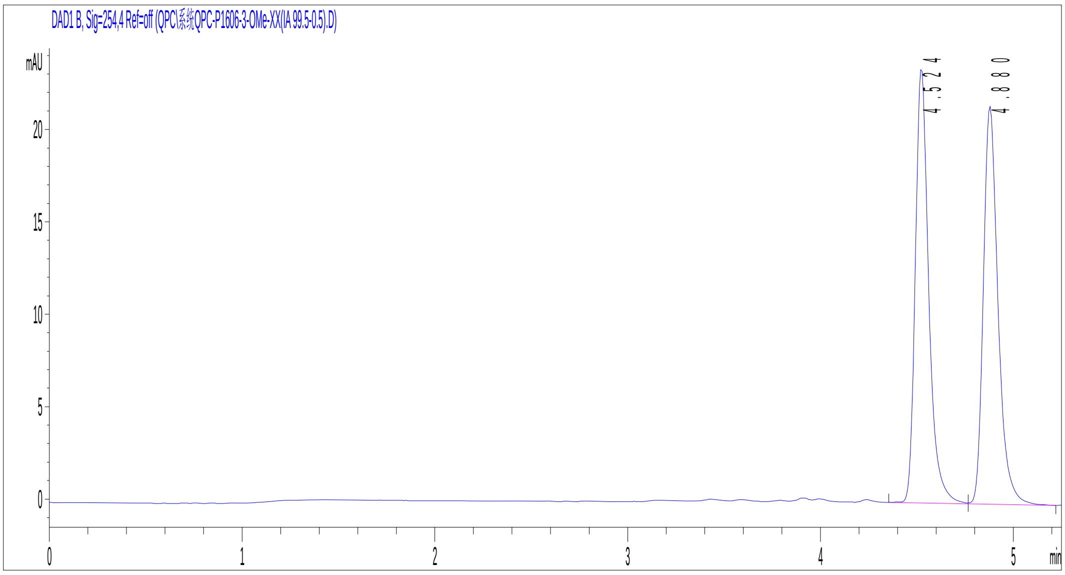

Peak	Ret Time	Type	Width	Area	Height	Area
#	[min]		[min]	mAU*s	[mAU]	%
1	4.524	BV	0.0723	113.8	23.6	50.002
2	4.88	VB	0.0817	113.8	21.5	49.998
Totals				227.6	45.1	

4q. Enantioenriched product, 88% ee

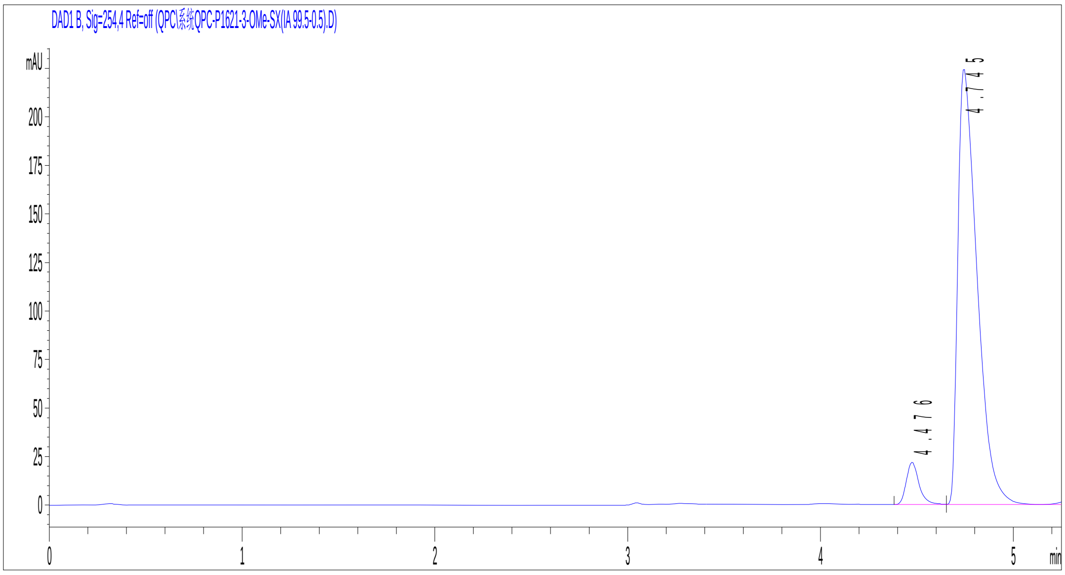

Peak	Ret Time	Type	Width	Area	Height	Area
#	[min]		[min]	mAU*s	[mAU]	%
1	4.476	BV	0.0713	99.7	21.8	5.965
2	4.745	VV R	0.1088	1571	224.6	94.035
Totals				1670.7	246.4	

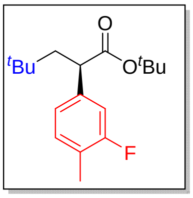

(*R*)-*tert*-butyl 2-(3-fluoro-4-methylphenyl)-4,4-dimethylpentanoate (4r)

The reaction was performed following the General Procedure 1 with $NiBr_2$ (2.2 mg, 0.01 mmol) and **L6** (5.4 mg, 0.011 mmol), *tert*-butyl acrylate (15.0 μL, 0.1 mmol, 1.0 equiv.), 4-bromo-2-fluoro-1-methylbenzene (37.8 mg, 25.3 μL， 0.2 mmol, 2.0 equiv.), ^{t}BuBr (54.8 mg, 44.9 μL, 0.4 mmol, 4.0 equiv.), Cy_2NMe (58.5 mg, 64 μL, 0.3 mmol), HEH (75.9 mg, 0.3 mmol) and 4CzIPN (8.0 mg, 0.01 mmol) in DMA (3.0 mL). The crude product was purified by flash chromatography on silica gel (eluted with petroleum ether:EtOAc = 50:1) to give the product (22.1 mg, 75% yield, 89% ee) as a white solid. Melting point: 71.9 – 72.8 ^{o}C; R_f= 0.56 (petroleum ether:EtOAc = 20:1). $[\alpha]_D^{25}$ = –23.27 (*c* = 0.583, $CHCl_3$). ^{1}H NMR (400 MHz, $CDCl_3$) δ: 7.09 (t, *J* = 7.9 Hz, 1H), 6.97 (m, 1H), 6.95 (s, 1H), 3.48 (dd, *J* = 9.3, 3.5 Hz, 1H), 2.26 – 2.18 (m, 4H), 1.46 (dd, *J* = 14.0, 3.5 Hz, 1H), 1.38 (s, 9H), 0.90 (s, 9H). ^{13}C{^{1}H} NMR (101 MHz, $CDCl_3$) δ: 173.68, 161.55 (d, *J* = 245.0 Hz), 141.30 (d, *J* = 7.4 Hz), 131.35 (d, *J* = 5.5 Hz), 123.29 – 122.95 (m), 114.37, 114.15, 80.62, 48.79, 47.22, 31.13, 29.57, 27.92, 14.32 (d, *J* = 3.5 Hz). ^{19}F NMR (375 MHz, $CDCl_3$) δ: -117.36 (s). The ee was determined by HPLC with a Daicel Chiralcel IA column (^{i}PrOH/hexanes = 0.2/99.8, 1.0 mL/min, 230 nm, major t_r = 3.966 min (*S*), minor t_r = 4.213 min (*R*)). FTIR (neat, cm^{-1}) ν: 3000, 2959, 2931, 1720, 1508, 1366, 1259, 1140, 1116, 1018, 847, 800, 764. HRMS: calcd for $C_{18}H_{28}FO_2$ $[M+H]^+$ 295.2073, found 295.2072.

4r. Racemic product

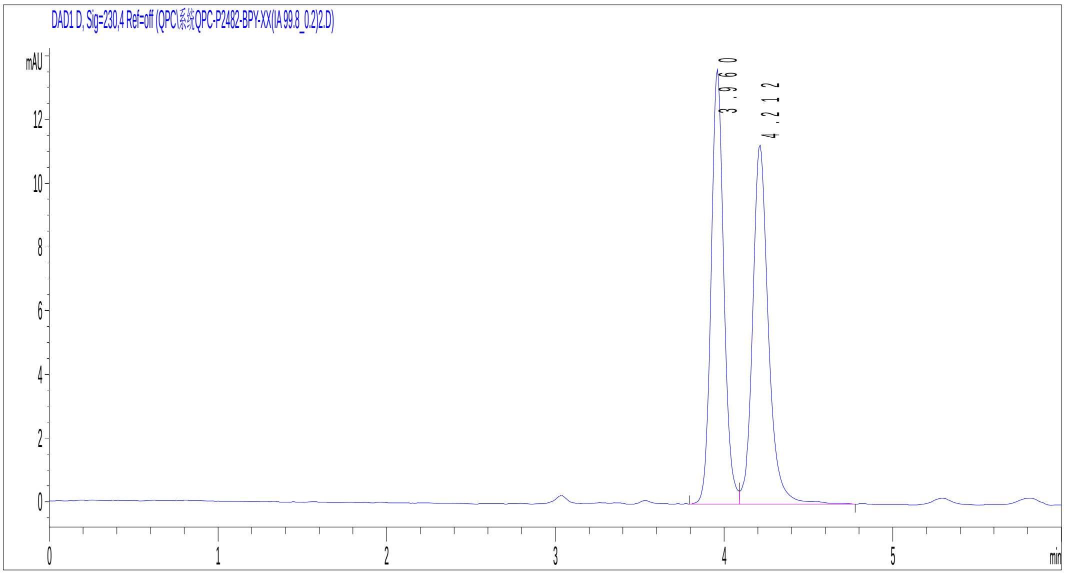

Peak	Ret Time	Type	Width	Area	Height	Area
#	[min]		[min]	mAU*s	[mAU]	%
1	3.96	BV	0.0791	71.4	13.6	49.301
2	4.212	VB	0.098	73.5	11.3	50.699
Totals				144.9	24.9	

4r. Enantioenriched product, 89% ee

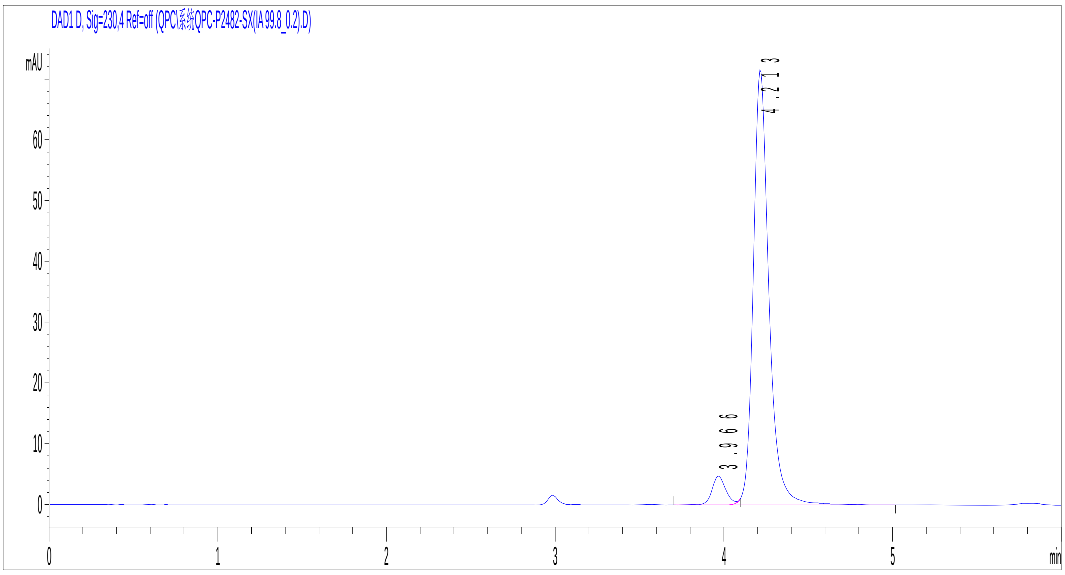

Peak	Ret Time	Type	Width	Area	Height	Area
#	[min]		[min]	mAU*s	[mAU]	%
1	3.966	BV E	0.0857	26.7	4.7	5.453
2	4.213	VB R	0.0973	462.9	71.7	94.547
Totals				489.6	76.4	

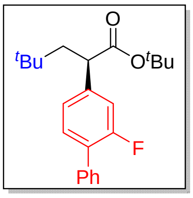

(*R*)-*tert*-butyl 2-(2-fluoro-[1,1'-biphenyl]-4-yl)-4,4-dimethylpentanoate (4s)

The reaction was performed following the General Procedure 1 with $NiBr_2$ (2.2 mg, 0.01 mmol) and **L6** (5.4 mg, 0.011 mmol), *tert*-butyl acrylate (15.0 μL, 0.1 mmol, 1.0 equiv.), 4-bromo-2-fluoro-1,1'-biphenyl (50.2 mg, 0.2 mmol, 2.0 equiv.), ^{t}BuBr (54.8 mg, 44.9 μL, 0.4 mmol, 4.0 equiv.), Cy_2NMe (58.5 mg, 64 μL, 0.3 mmol), HEH (75.9 mg, 0.3 mmol) and 4CzIPN (8.0 mg, 0.01 mmol) in DMA (3.0 mL). The crude product was purified by flash chromatography on silica gel (eluted with petroleum ether:EtOAc = 50:1) to give the product (29.6 mg, 83% yield, 89% ee) as a white solid. Melting point: 103.1 – 104.6 °C; R_f = 0.43 (petroleum ether:EtOAc = 20:1). $[\alpha]_D^{25}$ = –16.73 (c = 0.5, $CHCl_3$), *lit.* $[\alpha]_D^{25}$ = –10.4060 (c = 0.3200, $CHCl_3$, 90% ee). The spectroscopic data for this product match the literature data[8]. The ee was determined by HPLC with a Daicel Chiralcel IA column (^{i}PrOH/hexanes = 0.2/99.8, 1.0 mL/min, 254 nm, major t_r = 4.826 min (*S*), minor t_r = 5.244 min (*R*)). FTIR (neat, cm^{-1}) ν: 3436, 2956, 2918, 2849, 1716, 1402, 1366, 1139, 1018, 872, 819, 761, 693.

4s. Racemic product

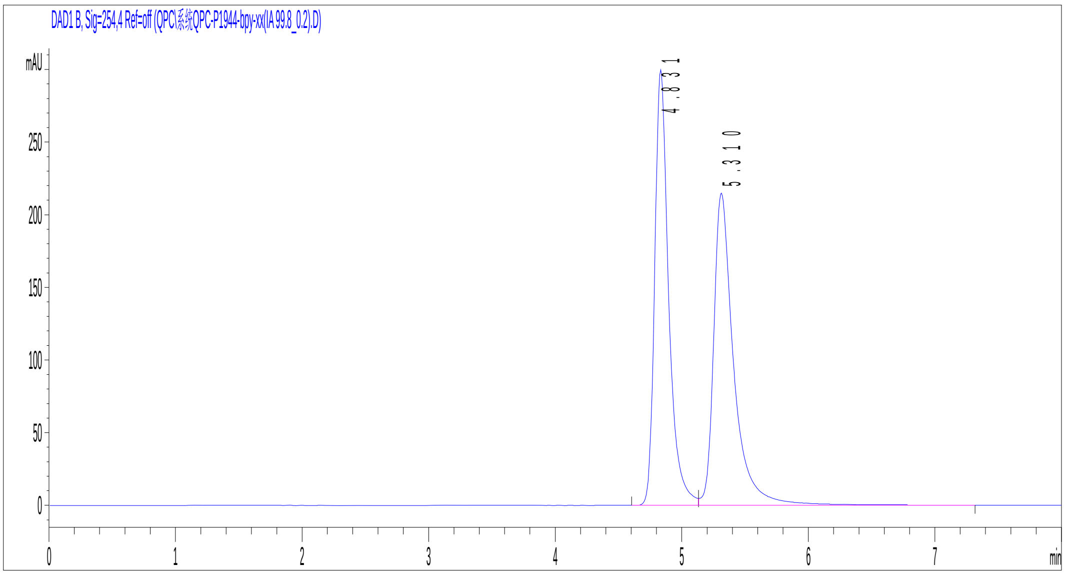

Peak	Ret Time	Type	Width	Area	Height	Area
#	[min]		[min]	mAU*s	[mAU]	%
1	4.831	BV	0.1154	2297.5	299.7	49.291
2	5.31	VB	0.1622	2363.6	215	50.709
Totals				4661.1	514.7	

4s. Enantioenriched product, 89% ee

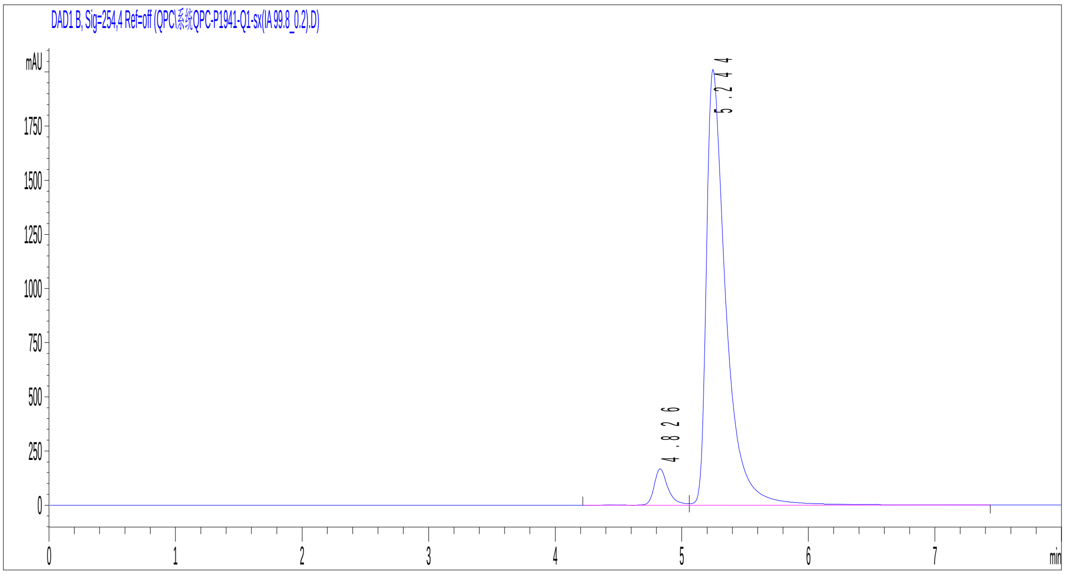

Peak	Ret Time	Type	Width	Area	Height	Area
#	[min]		[min]	mAU*s	[mAU]	%
1	4.826	VV R	0.1137	1271.2	167.6	5.553
2	5.244	VB	0.1575	21622.8	2010.7	94.447
Totals				22894	2178.3	

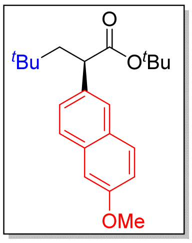

(*R*)-*tert*-butyl 2-(6-methoxynaphthalen-2-yl)-4,4-dimethylpentanoate (4t)

The reaction was performed following the General Procedure 1 with $NiBr_2$ (2.2 mg, 0.01 mmol) and **L6** (5.4 mg, 0.011 mmol), *tert*-butyl acrylate (15.0 μL, 0.1 mmol, 1.0 equiv.), 2-bromo-6-methoxynaphthalene (47.4 mg, 0.2 mmol, 2.0 equiv.), ^{t}BuBr (54.8 mg, 44.9 μL, 0.4 mmol, 4.0 equiv.), Cy_2NMe (58.5 mg, 64 μL, 0.3 mmol), HEH (75.9 mg, 0.3 mmol) and 4CzIPN (8.0 mg, 0.01 mmol) in DMA (3.0 mL). The crude product was purified by flash chromatography on silica gel (eluted with petroleum ether:EtOAc = 50:1) to give the product (28.8 mg, 84% yield, 88% ee) as a white solid. Melting point: 104.0 – 104.9 °C; R_f = 0.47 (petroleum ether:EtOAc = 20:1). $[\alpha]_D^{25}$ = –21.95 (*c* = 0.577, $CHCl_3$). ^{1}H NMR (400 MHz, $CDCl_3$) δ: 7.72 – 7.65 (m, 3H), 7.42 (dd, *J* = 8.4, 1.9 Hz, 1H), 7.16 – 7.07 (m, 2H), 3.91 (s, 3H), 3.66 (dd, *J* = 9.2, 3.5 Hz, 1H), 2.34 (dd, *J* = 14.0, 9.2 Hz, 1H), 1.58 (dd, *J* = 14.0, 3.6 Hz, 1H), 1.37 (s, 9H), 0.93 (s, 9H). ^{13}C{^{1}H} NMR (101 MHz, $CDCl_3$) δ: 174.12, 157.57, 136.95, 133.56, 129.37, 129.06, 127.03, 126.72, 126.11, 118.87, 105.63, 80.46, 55.38, 49.26, 47.30, 31.21, 29.64, 27.96. The ee was determined by HPLC with a Daicel Chiralcel IA column (^{i}PrOH/hexanes = 0.2/99.8, 1.0 mL/min, 254 nm, major t_r = 7.575 min (*S*), minor t_r = 7.978 min (*R*)). FTIR (neat, cm^{-1}) ν: 2949, 2928, 2865, 1725, 1606, 1506, 1364, 1140, 1033, 853, 845, 801. HRMS: calcd for $C_{22}H_{31}O_3$ $[M+H]^+$ 343.2273, found 343.2274.

4t. Racemic product

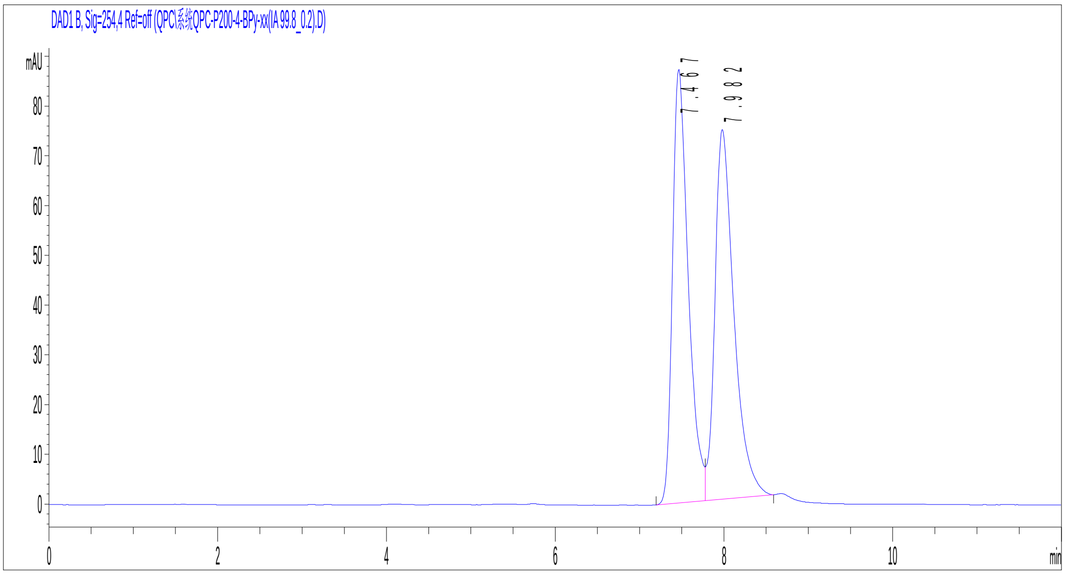

Peak	Ret Time	Type	Width	Area	Height	Area
#	[min]		[min]	mAU*s	[mAU]	%
1	7.467	BV	0.1976	1132.2	87.1	49.215
2	7.982	VB	0.2354	1168.3	74.3	50.785
Totals				2300.5	161.4	

4t. Enantioenriched product, 88% ee

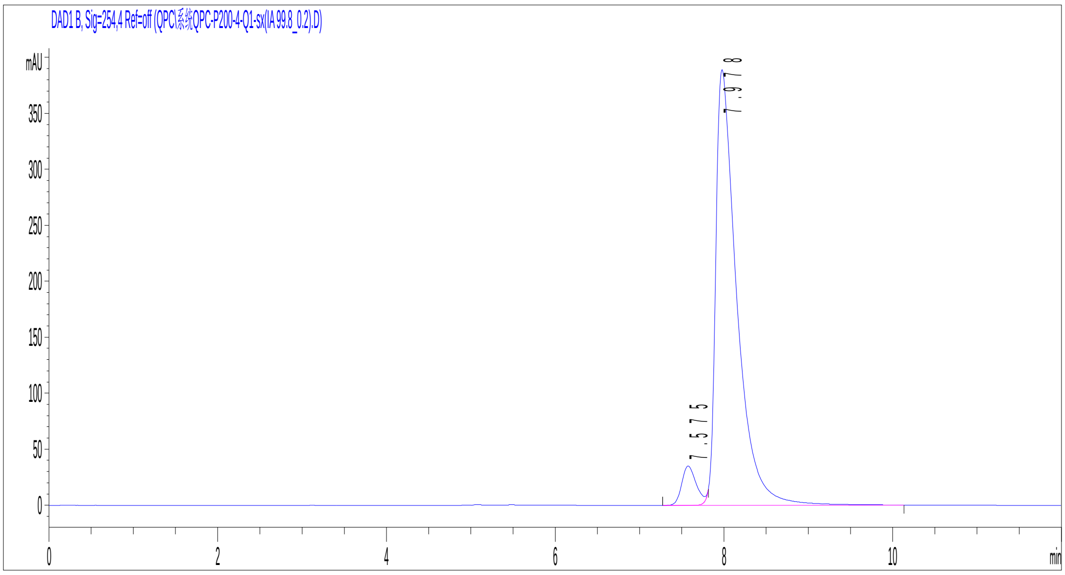

Peak	Ret Time	Type	Width	Area	Height	Area
#	[min]		[min]	mAU*s	[mAU]	%
1	7.575	BV E	0.1808	418.2	35.2	5.840
2	7.978	VB R	0.2542	6743.2	389.1	94.160
Totals				7161.4	424.3	

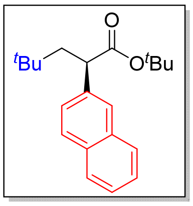

(*R*)-*tert*-butyl 4,4-dimethyl-2-(naphthalen-2-yl)pentanoate (4u)

The reaction was performed following the General Procedure 1 with $NiBr_2$ (2.2 mg, 0.01 mmol) and **L6** (5.4 mg, 0.011 mmol), *tert*-butyl acrylate (15.0 μL, 0.1 mmol, 1.0 equiv.), 2-bromonaphthalene (41.4 mg, 0.2 mmol, 2.0 equiv.), ^{t}BuBr (54.8 mg, 44.9 μL, 0.4 mmol, 4.0 equiv.), Cy_2NMe (58.5 mg, 64 μL, 0.3 mmol), HEH (75.9 mg, 0.3 mmol) and 4CzIPN (8.0 mg, 0.01 mmol) in DMA (3.0 mL). The crude product was purified by flash chromatography on silica gel (eluted with petroleum ether:EtOAc = 50:1) to give the product (25.0 mg, 80% yield, 90% ee) as a white solid. Melting point: 90.1 – 91.9 °C; R_f = 0.56 (petroleum ether:EtOAc = 20:1). $[\alpha]_D^{25}$ = –45.22 (*c* = 0.443, $CHCl_3$). ^{1}H NMR (400 MHz, $CDCl_3$) δ: 7.83 – 7.80 (m, 1H), 7.78 (d, *J* = 8.1 Hz, 2H), 7.74 (s, 1H), 7.49 – 7.40 (m, 3H), 3.70 (dd, *J* = 9.2, 3.5 Hz, 1H), 2.36 (dd, *J* = 14.0, 9.3 Hz, 1H), 1.59 (dd, *J* = 14.0, 3.5 Hz, 1H), 1.37 (s, 9H), 0.94 (s, 9H). $^{13}C\{^1H\}$ NMR (101 MHz, $CDCl_3$) δ: 173.97, 139.23, 133.57, 132.49, 128.20, 127.89, 127.68, 126.26, 126.21, 126.06, 125.66, 80.58, 49.46, 47.30, 31.25, 29.64, 27.95. The ee was determined by HPLC with a Daicel Chiralcel IA column (^{i}PrOH/hexanes = 0.2/99.8, 1.0 mL/min, 250 nm, major t_r = 5.204 min (*S*), minor t_r = 5.441 min (*R*)). FTIR (neat, cm^{-1}) ν: 2949, 2863, 2850, 1724, 1364 1259, 1140, 1018, 844, 802, 763, 749. HRMS: calcd for $C_{21}H_{28}NaO_2$ $[M+Na]^+$ 335.1987, found 335.1982.

4u. Racemic product

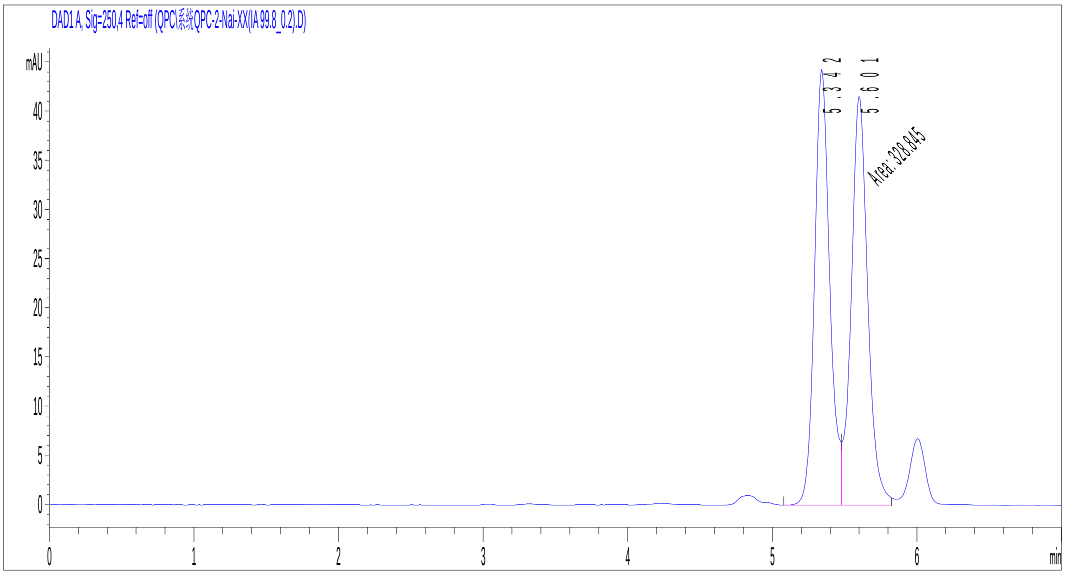

Peak	Ret Time	Type	Width	Area	Height	Area
#	[min]		[min]	mAU*s	[mAU]	%
1	5.342	BV	0.1112	323.6	44.3	49.595
2	5.601	MF	0.1317	328.8	41.6	50.405
Totals				652.4	85.9	

4u. Enantioenriched product , 90% ee

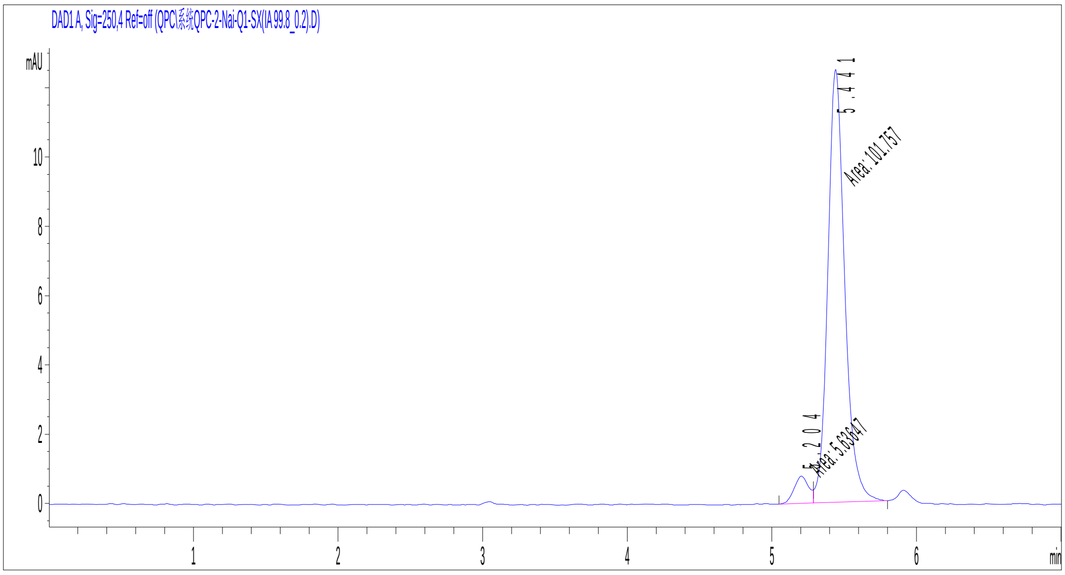

Peak	Ret Time	Type	Width	Area	Height	Area
#	[min]		[min]	mAU*s	[mAU]	%
1	5.204	MF	0.1191	5.6	7.9E-1	5.248
2	5.441	FM	0.1355	101.8	12.5	94.752
Totals				107.4	13.29	

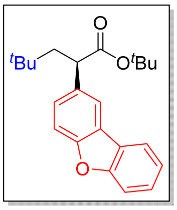

(*R*)-*tert*-butyl 2-(dibenzo[b,d]furan-2-yl)-4,4-dimethylpentanoate (4v)

The reaction was performed following the General Procedure 1 with $NiBr_2$ (2.2 mg, 0.01 mmol) and **L6** (5.4 mg, 0.011 mmol), *tert*-butyl acrylate (15.0 μL, 0.1 mmol, 1.0 equiv.), 2-bromodibenzo[b,d]furan (49.4 mg, 0.2 mmol, 2.0 equiv.), ^{t}BuBr (54.8 mg, 44.9 μL, 0.4 mmol, 4.0 equiv.), Cy_2NMe (58.5 mg, 64 μL, 0.3 mmol), HEH (75.9 mg, 0.3 mmol) and 4CzIPN (8.0 mg, 0.01 mmol) in DMA (3.0 mL). The crude product was purified by flash chromatography on silica gel (eluted with petroleum ether:EtOAc = 50:1) to give the product (30.3 mg, 86% yield, 85% ee) as a white solid. Melting point: 106.8 – 109.1 °C; R_f = 0.64 (petroleum ether:EtOAc = 20:1). $[\alpha]_D^{25}$ = –21.20 (c = 0.703, $CHCl_3$). ^{1}H NMR (400 MHz, $CDCl_3$) δ: 7.98 – 7.93 (m, 1H), 7.90 (d, J = 1.8 Hz, 1H), 7.57 – 7.53 (m, 1H), 7.49 (d, J = 8.5 Hz, 1H), 7.47 – 7.39 (m, 2H), 7.34 (td, J = 7.6, 1.0 Hz, 1H), 3.70 (dd, J = 9.2, 3.6 Hz, 1H), 2.37 (dd, J = 13.9, 9.3 Hz, 1H), 1.60 (dd, J = 14.0, 3.6 Hz, 1H), 1.37 (s, 9H), 0.94 (s, 9H). ^{13}C{^{1}H} NMR (101 MHz, $CDCl_3$) δ: 174.28, 156.60, 155.28, 136.40, 127.20, 127.08, 124.42, 124.31, 122.74, 120.81, 119.68, 111.75, 111.55, 80.56, 49.19, 47.70, 31.24, 29.66, 27.95. The ee was determined by HPLC with a Daicel Chiralcel IC-3 column (^{i}PrOH/hexanes = 0.2/99.8, 1.0 mL/min, 254 nm, major t_r = 6.129min (*S*), minor t_r = 7.522 min (*R*)). FTIR (neat, cm^{-1}) ν: 2948, 2865, 1725, 1477, 1448, 1365, 1200, 1142, 1120, 1022, 844, 802, 746. HRMS: calcd for $C_{23}H_{29}O_3$ $[M+H]^+$ 353.2117, found 353.2121.

4v. Racemic product

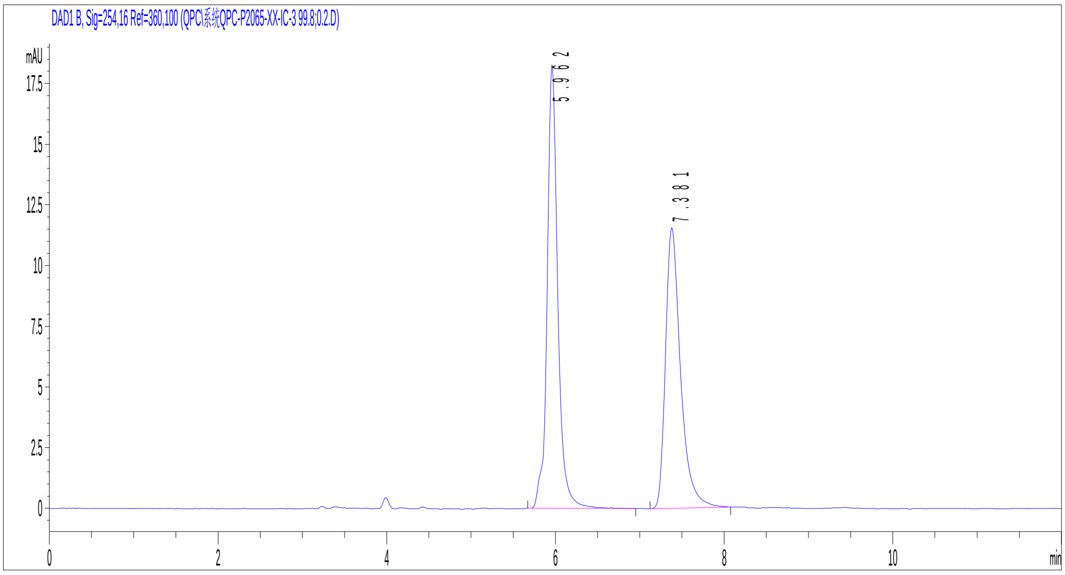

Peak	Ret Time	Type	Width	Area	Height	Area
#	[min]		[min]	mAU*s	[mAU]	%
1	5.962	BB	0.1322	159.7	18.2	52.602
2	7.381	BB	0.1873	143.9	11.6	47.398
Totals				303.6	29.8	

4v. Enantioenriched product, 85% ee

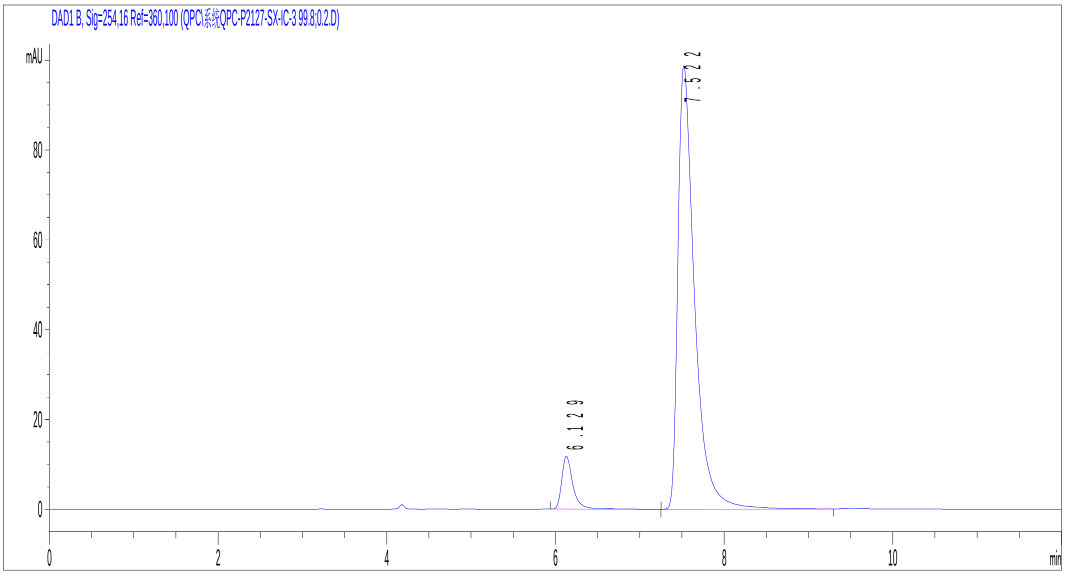

Peak	Ret Time	Type	Width	Area	Height	Area
#	[min]		[min]	mAU*s	[mAU]	%
1	6.129	BB	0.1401	109.1	11.8	7.478
2	7.522	BB	0.2055	1350.3	98.8	92.522
Totals				1459.4	110.6	

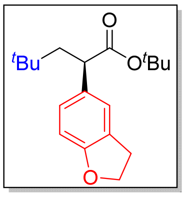

(*R*)-*tert*-butyl 2-(2,3-dihydrobenzofuran-5-yl)-4,4-dimethylpentanoate (4w)

The reaction was performed following the General Procedure 1 with $NiBr_2$ (2.2 mg, 0.01 mmol) and **L6** (5.4 mg, 0.011 mmol), *tert*-butyl acrylate (15.0 μL, 0.1 mmol, 1.0 equiv.), 5-bromo-2,3-dihydrobenzofuran (39.8 mg, 0.2 mmol, 2.0 equiv.), ^{t}BuBr (54.8 mg, 44.9 μL, 0.4 mmol, 4.0 equiv.), Cy_2NMe (58.5 mg, 64 μL, 0.3 mmol), HEH (75.9 mg, 0.3 mmol) and 4CzIPN (8.0 mg, 0.01 mmol) in DMA (3.0 mL). The crude product was purified by flash chromatography on silica gel (eluted with petroleum ether:EtOAc = 50:1) to give the product (25.1 mg, 75% yield, 86% ee) as a white solid. Melting point: 99.7 – 102.2 °C; R_f = 0.39 (petroleum ether:EtOAc = 20:1). $[\alpha]_D^{25}$ = –13.81 (*c* = 0.333, $CHCl_3$). ^{1}H NMR (400 MHz, $CDCl_3$) δ: 7.14 (s, 1H), 7.02 (d, *J* = 8.1 Hz, 1H), 6.69 (d, *J* = 8.2 Hz, 1H), 4.54 (t, *J* = 8.7 Hz, 2H), 3.45 (dd, *J* = 9.4, 3.4 Hz, 1H), 3.18 (t, *J* = 8.7 Hz, 2H), 2.23 (dd, *J* = 13.9, 9.4 Hz, 1H), 1.45 (dd, *J* = 13.9, 3.4 Hz, 1H), 1.38 (s, 9H), 0.90 (s, 9H). ^{13}C{^{1}H} NMR (101 MHz, $CDCl_3$) δ: 174.46, 158.97, 133.85, 127.40, 127.20, 124.13, 109.06, 80.26, 71.33, 48.62, 47.59, 31.11, 29.85, 29.60, 27.97. The ee was determined by HPLC with a Daicel Chiralcel IA column (^{i}PrOH/hexanes = 2/98, 1.0 mL/min, 230 nm, major t_r = 13.038 min (*S*), minor t_r = 13.975 min (*R*)). FTIR (neat, cm^{-1}) ν: 3435, 2955, 2867, 1726, 1491, 1366, 1244, 1144, 984, 945, 847, 815. HRMS: calcd for $C_{19}H_{29}O_3$ $[M+H]^+$ 305.2117, found 305.2110.

4w. Racemic product

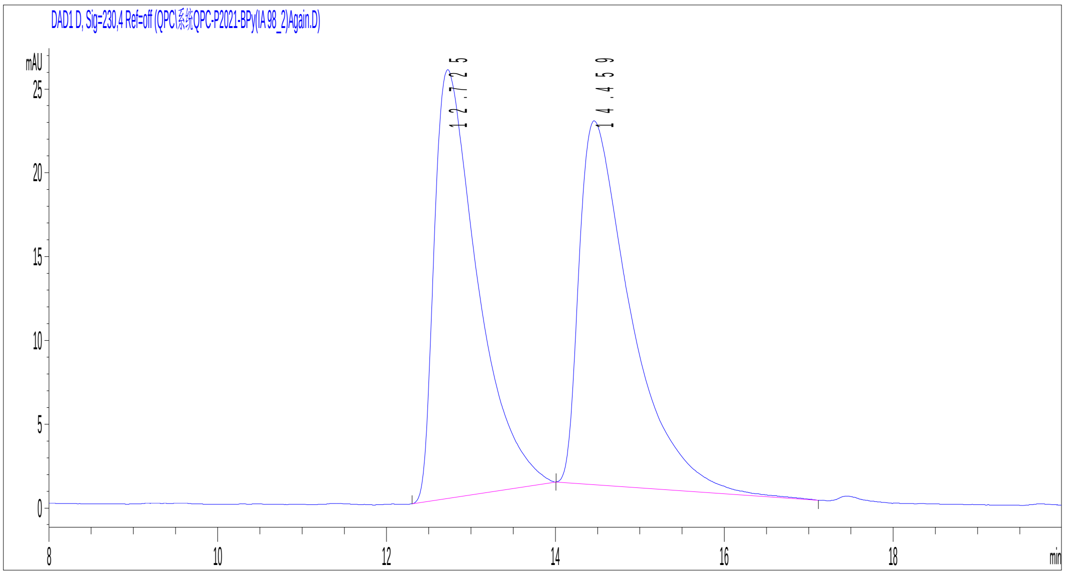

Peak	Ret Time	Type	Width	Area	Height	Area
#	[min]		[min]	mAU*s	[mAU]	%
1	12.725	BB	0.5287	913.4	25.6	49.720
2	14.459	BB	0.6185	923.6	21.7	50.280
Totals				1837	47.3	

4w. Enantioenriched product, 86% ee

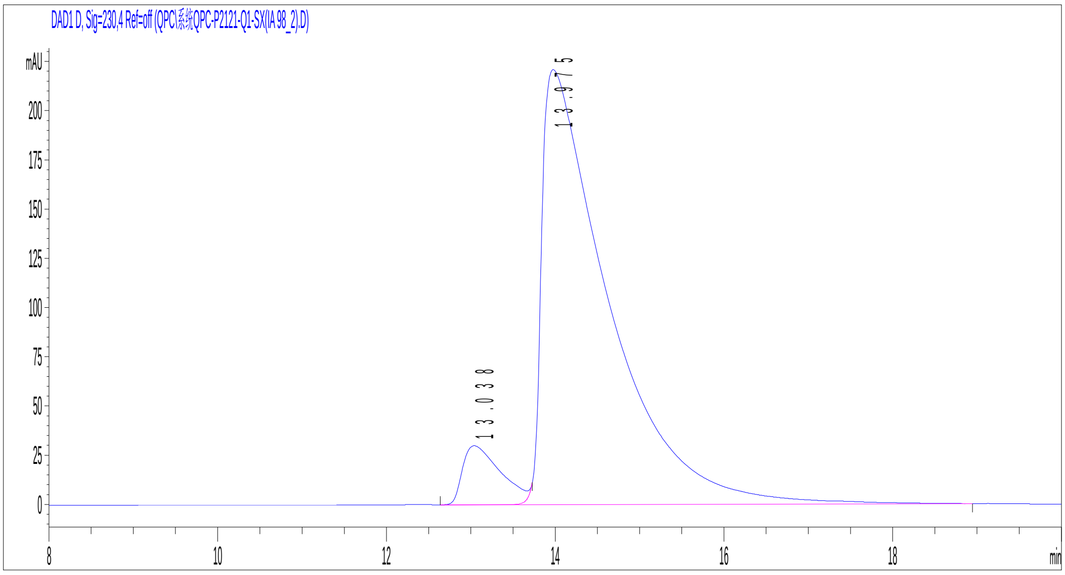

Peak	Ret Time	Type	Width	Area	Height	Area
#	[min]		[min]	mAU*s	[mAU]	%
1	13.038	BV E	0.465	941.7	29.9	7.201
2	13.975	VB R	0.7691	12136	220.8	92.799
Totals				13077.7	250.7	

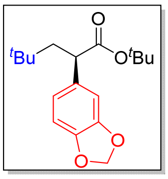

(*R*)-*tert*-butyl 2-(benzo[d][1,3]dioxol-5-yl)-4,4-dimethylpentanoate (4x)

The reaction was performed following the General Procedure 1 with $NiBr_2$ (2.2 mg, 0.01 mmol) and **L6** (5.4 mg, 0.011 mmol), *tert*-butyl acrylate (15.0 μL, 0.1 mmol, 1.0 equiv.), 5-bromobenzo[d][1,3]dioxole (40.2 mg, 24.1 μL, 0.2 mmol, 2.0 equiv.), ^{t}BuBr (54.8 mg, 44.9 μL, 0.4 mmol, 4.0 equiv.), Cy_2NMe (58.5 mg, 64 μL, 0.3 mmol), HEH (75.9 mg, 0.3 mmol) and 4CzIPN (8.0 mg, 0.01 mmol) in DMA (3.0 mL). The crude product was purified by flash chromatography on silica gel (eluted with petroleum ether:EtOAc = 50:1) to give the product (23.0 mg, 75% yield, 89% ee) as a white solid. Melting point: 67.6 – 69.2 °C; R_f = 0.79 (petroleum ether:EtOAc = 20:1). $[\alpha]_D^{25}$ = –13.39 (*c* = 0.483, $CHCl_3$). ^{1}H NMR (400 MHz, $CDCl_3$) δ: 6.82 (s, 1H), 6.73 (m, 2H), 5.93 (s, 2H), 3.44 (dd, *J* = 9.2, 3.2 Hz, 1H), 2.20 (dd, *J* = 13.9, 9.3 Hz, 1H), 1.45 (dd, *J* = 13.9, 3.3 Hz, 1H), 1.38 (s, 9H), 0.90 (s, 9H). ^{13}C{^{1}H} NMR (101 MHz, $CDCl_3$) δ: 174.05, 147.68, 146.36, 135.58, 120.92, 108.23, 108.17, 101.01, 80.47, 48.91, 47.38, 31.11, 29.58, 27.95. The ee was determined by HPLC with a Daicel Chiralcel IC-3 column (^{i}PrOH/hexanes = 0.5/99.5, 1.0 mL/min, 250 nm, major t_r = 4.868 min (*S*), minor t_r = 5.02 min (*R*)). FTIR (neat, cm^{-1}) ν: 3043, 2954, 2869, 1720, 1487, 1366, 1145, 1039, 933, 871, 805, 766. HRMS: calcd for $C_{18}H_{27}O_4$ $[M+H]^+$ 307.1909, found 307.1910.

4x. Racemic product

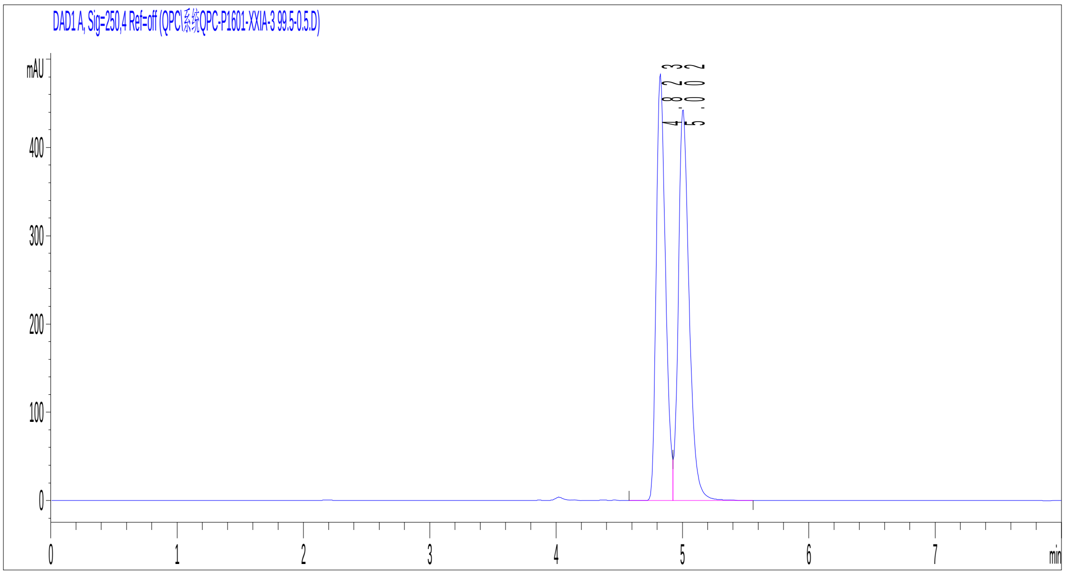

Peak	Ret Time	Type	Width	Area	Height	Area
#	[min]		[min]	mAU*s	[mAU]	%
1	4.823	BV	0.0794	2466	484	48.490
2	5.002	VB	0.0909	2619.6	443.7	51.510
Totals				5085.6	927.7	

4x. Enantioenriched product, 89% ee

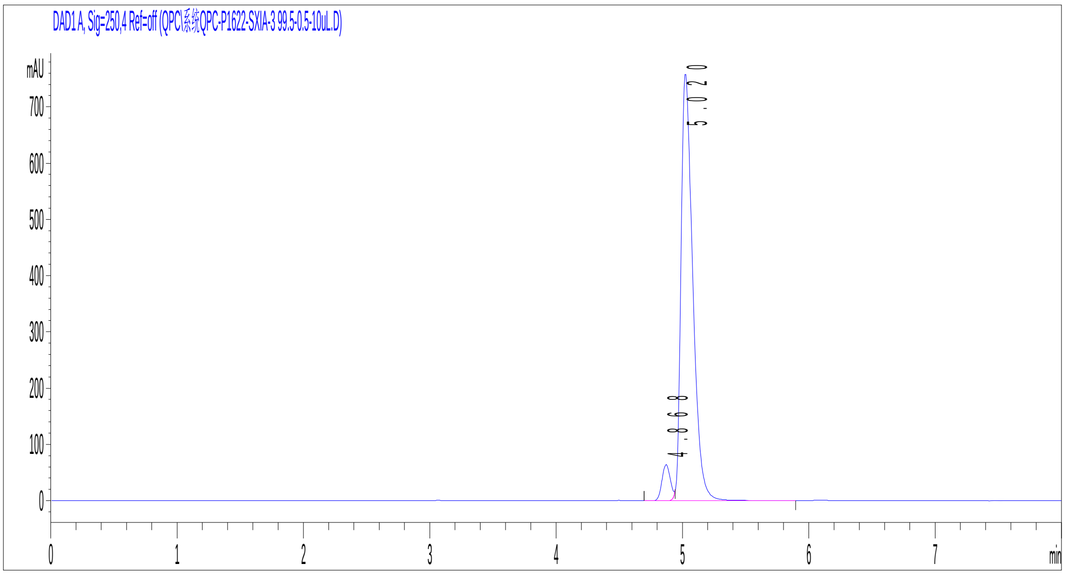

Peak	Ret Time	Type	Width	Area	Height	Area
#	[min]		[min]	mAU*s	[mAU]	%
1	4.868	BV E	0.0732	294.1	64.4	5.660
2	5.02	VB R	0.0992	4902.1	760.6	94.340
Totals				5196.2	825	

(R)-*tert*-butyl 2-(benzofuran-5-yl)-4,4-dimethylpentanoate (4y)

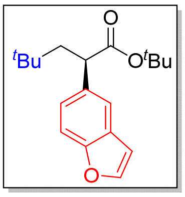

The reaction was performed following the General Procedure 1 with $NiBr_2$ (2.2 mg, 0.01 mmol) and **L6** (5.4 mg, 0.011 mmol), *tert*-butyl acrylate (15.0 μL, 0.1 mmol, 1.0 equiv.), 5-bromobenzofuran (39.4 mg, 0.2 mmol, 2.0 equiv.), ^{t}BuBr (54.8 mg, 44.9 μL, 0.4 mmol, 4.0 equiv.), Cy_2NMe (58.5 mg, 64 μL, 0.3 mmol), HEH (75.9 mg, 0.3 mmol) and 4CzIPN (8.0 mg, 0.01 mmol) in DMA (3.0 mL). The crude product was purified by flash chromatography on silica gel (eluted with petroleum ether:EtOAc = 50:1) to give the product (25.1 mg, 83% yield, 89% ee) as a white solid. Melting point: 88.7 – 88.9 °C; R_f = 0.39 (petroleum ether:EtOAc = 20:1). $[\alpha]_D^{25}$ = –16.74 (c = 0.667, $CHCl_3$). ^{1}H NMR (400 MHz, $CDCl_3$) δ: 7.60 (d, J = 2.2 Hz, 1H), 7.53 (d, J = 1.8 Hz, 1H), 7.42 (d, J = 8.5 Hz, 1H), 7.27 – 7.23 (m, 1H), 6.75 – 6.71 (m, 1H), 3.62 (dd, J = 9.4, 3.4 Hz, 1H), 2.32 (dd, J = 14.0, 9.4 Hz, 1H), 1.53 (dd, J = 14.0, 3.5 Hz, 1H), 1.37 (s, 9H), 0.92 (s, 9H). $^{13}C\{^1H\}$ NMR (101 MHz, $CDCl_3$) δ: 174.36, 154.05, 145.31, 136.40, 127.61, 124.24, 120.07, 111.28, 106.72, 80.40, 49.10, 47.76, 31.19, 29.61, 27.95. The ee was determined by HPLC with a Daicel Chiralcel IA column (^{i}PrOH/hexanes = 0.2/99.8, 1.0 mL/min, 254 nm, major t_r = 4.817 min (S), minor t_r = 4.998 min (R)). FTIR (neat, cm^{-1}) ν: 3420, 2950, 2866, 1718, 1464, 1326, 1260, 1144, 1026, 885, 843, 827, 764. HRMS: calcd for $C_{19}H_{27}O_3$ $[M+H]^+$ 303.1960, found 303.1966.

4y. Racemic product

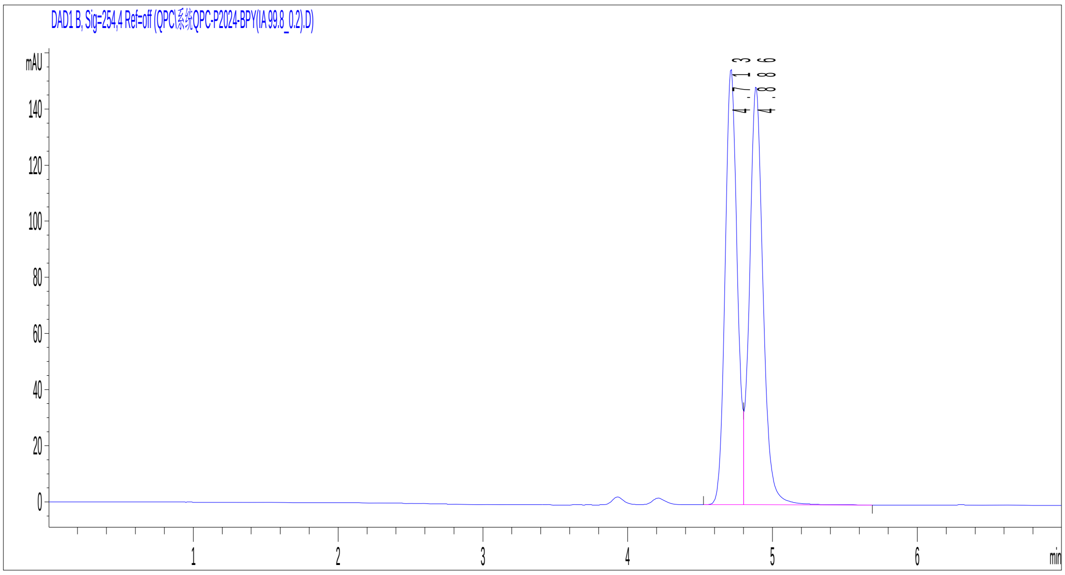

Peak	Ret Time	Type	Width	Area	Height	Area
#	[min]		[min]	mAU*s	[mAU]	%
1	4.713	BV	0.0877	901.9	155.4	48.162
2	4.886	VB	0.0999	970.7	149.2	51.838
Totals				1872.6	304.6	

4y. Enantioenriched product, 89% ee

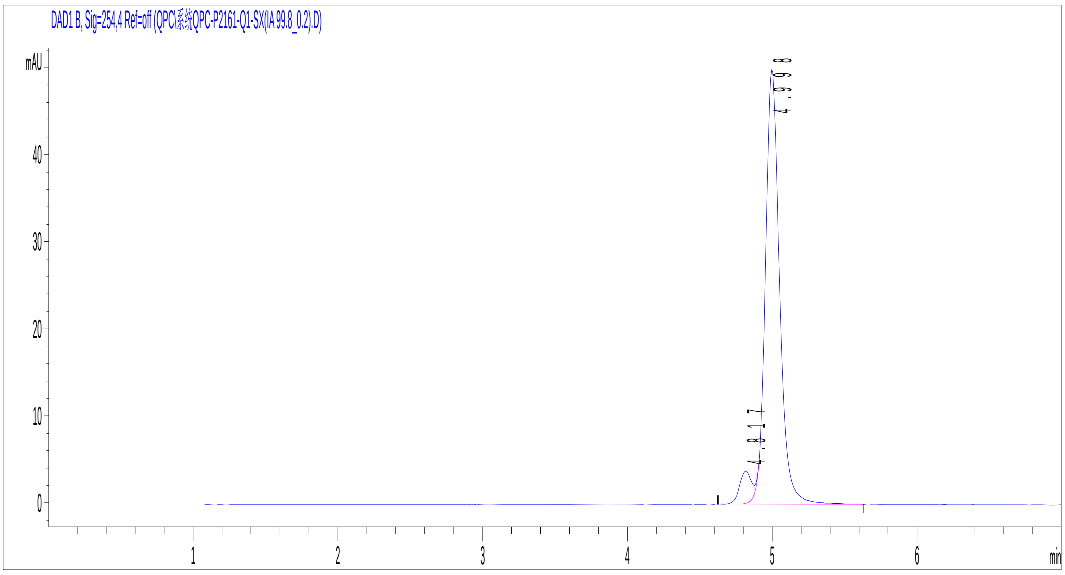

Peak	Ret Time	Type	Width	Area	Height	Area
#	[min]		[min]	mAU*s	[mAU]	%
1	4.817	BV E	0.0857	20.9	3.7	5.747
2	4.998	VB R	0.104	342.5	49.9	94.253
Totals				363.4	53.6	

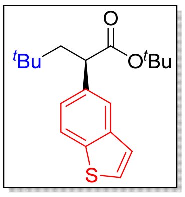

(*R*)-*tert*-butyl 2-(benzo[b]thiophen-5-yl)-4,4-dimethylpentanoate (4z)

The reaction was performed following the General Procedure 1 with $NiBr_2$ (2.2 mg, 0.01 mmol) and **L6** (5.4 mg, 0.011 mmol), *tert*-butyl acrylate (15.0 μL, 0.1 mmol, 1.0 equiv.), 5-bromobenzo[b]thiophene (42.6 mg, 0.2 mmol, 2.0 equiv.), ^{t}BuBr (54.8 mg, 44.9 μL, 0.4 mmol, 4.0 equiv.), Cy_2NMe (58.5 mg, 64 μL, 0.3 mmol), HEH (75.9 mg, 0.3 mmol) and 4CzIPN (8.0 mg, 0.01 mmol) in DMA (3.0 mL). The crude product was purified by flash chromatography on silica gel (eluted with petroleum ether:EtOAc = 50:1) to give the product (26.1 mg, 82% yield, 88% ee) as a white solid. Melting point: 133.6 – 136.0 °C; R_f = 0.42 (petroleum ether:EtOAc = 20:1). $[\alpha]_D^{25}$ = –22.37 (*c* = 0.517, $CHCl_3$). ^{1}H NMR (400 MHz, $CDCl_3$) δ: 7.79 (d, *J* = 8.4 Hz, 1H), 7.75 (s, 1H), 7.42 (d, *J* = 5.4 Hz, 1H), 7.35 – 7.27 (m, 2H), 3.65 (dd, *J* = 9.3, 3.4 Hz, 1H), 2.34 (dd, *J* = 14.5, 9.3 Hz, 1H), 1.55 (dd, *J* = 14.2, 3.1 Hz, 1H), 1.37 (d, *J* = 0.8 Hz, 9H), 0.93 (d, *J* = 0.8 Hz, 9H). ^{13}C{^{1}H} NMR (101 MHz, $CDCl_3$) δ: 174.14, 139.98, 138.24, 138.05, 126.71, 124.44, 123.96, 122.55, 122.51, 80.50, 49.20, 47.66, 31.21, 29.62, 27.95. The ee was determined by HPLC with a Daicel Chiralcel IA column (^{i}PrOH/hexanes = 0.2/99.8, 1.0 mL/min, 254 nm, major t_r = 5.475 min (*S*), minor t_r = 5.944 min (*R*)). FTIR (neat, cm^{-1}) ν: 3104, 3080, 2948, 2864, 1719, 1420, 1364, 1139, 1054, 843, 829, 765, 704. HRMS: calcd for $C_{19}H_{26}NaO_2S$ $[M+Na]^+$ 341.1551, found 341.1552.

4z. Racemic product

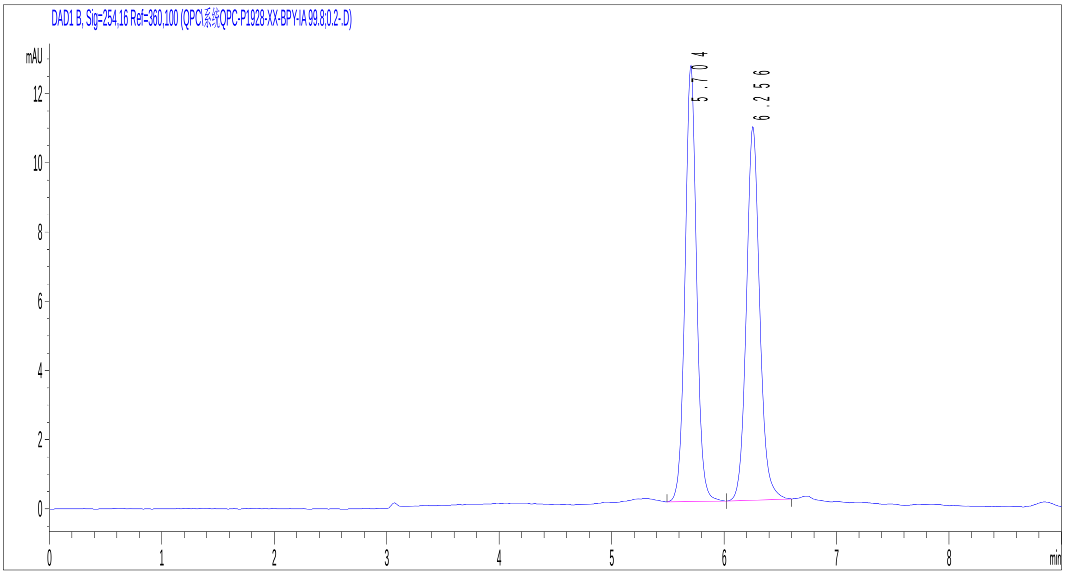

Peak	Ret Time	Type	Width	Area	Height	Area
#	[min]		[min]	mAU*s	[mAU]	%
1	5.704	BB	0.1089	89.6	12.6	50.114
2	6.256	BB	0.1263	89.2	10.8	49.886
Totals				178.8	23.4	

4z. Enantioenriched product, 88% ee

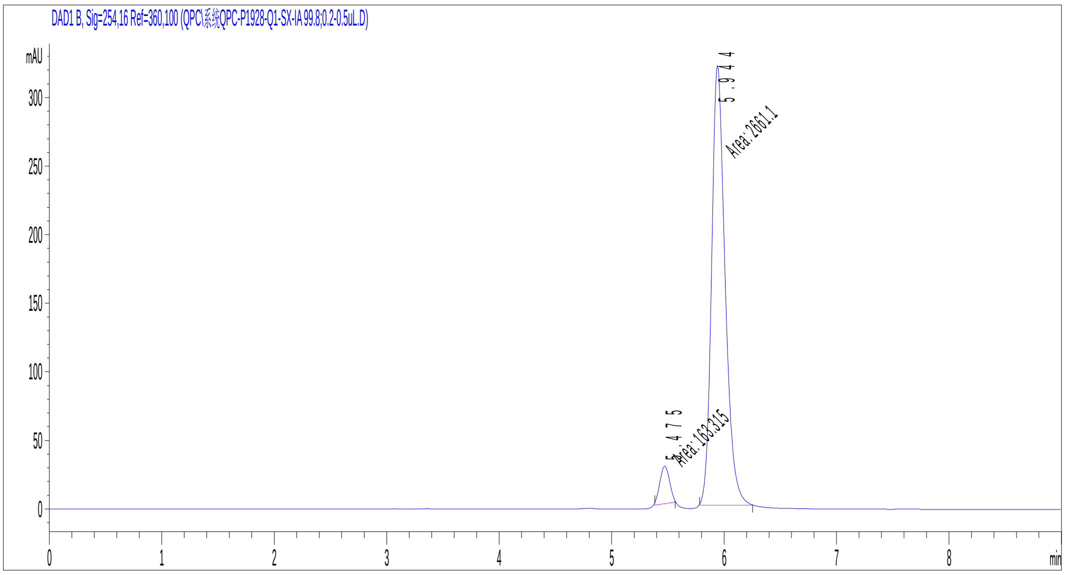

Peak	Ret Time	Type	Width	Area	Height	Area
#	[min]		[min]	mAU*s	[mAU]	%
1	5.475	MM	0.0991	163.3	27.5	5.782
2	5.944	MM	0.1383	2661.1	320.8	94.218
Totals				2824.4	348.3	

(*R*)-*tert*-butyl 4,4-dimethyl-2-(1-methyl-1*H*-indol-5-yl)pentanoate (4aa)

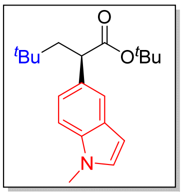

The reaction was performed following the General Procedure 1 with $NiBr_2$ (2.2 mg, 0.01 mmol) and **L6** (5.4 mg, 0.011 mmol), *tert*-butyl acrylate (15.0 μL, 0.1 mmol, 1.0 equiv.), 5-bromo-1-methyl-1*H*-indole (42.0 mg, 0.2 mmol, 2.0 equiv.), ^{t}BuBr (54.8 mg, 44.9 μL, 0.4 mmol, 4.0 equiv.), Cy_2NMe (58.5 mg, 64 μL, 0.3 mmol), HEH (75.9 mg, 0.3 mmol) and 4CzIPN (8.0 mg, 0.01 mmol) in DMA (3.0 mL). The crude product was purified by flash chromatography on silica gel (eluted with petroleum ether:EtOAc = 50:1) to give the product (23.7 mg, 75% yield, 83% ee) as a white solid. Melting point: 74.9 – 79.5 °C; R_f = 0.44 (petroleum ether:EtOAc = 20:1). $[\alpha]_D^{25}$ = –12.26 (*c* = 0.257, $CHCl_3$). ^{1}H NMR (400 MHz, $CDCl_3$) δ: 7.54 (s, 1H), 7.24 (d, *J* = 8.5 Hz, 1H), 7.19 (dd, *J* = 8.5, 1.7 Hz, 1H), 7.01 (d, *J* = 3.1 Hz, 1H), 6.44 – 6.42 (m, 1H), 3.75 (s, 3H), 3.62 (dd, *J* = 9.5, 3.3 Hz, 1H), 2.33 (dd, *J* = 14.0, 9.4 Hz, 1H), 1.54 (dd, *J* = 14.0, 3.3 Hz, 1H), 1.36 (s, 9H), 0.92 (s, 9H). ^{13}C{^{1}H} NMR (101 MHz, $CDCl_3$) δ: 174.78, 135.86, 132.86, 129.11, 128.64, 121.63, 119.76, 109.22, 100.91, 80.06, 49.22, 47.94, 32.95, 31.18, 29.67, 28.01. The ee was determined by HPLC with a Daicel Chiralcel IC-3 column (^{i}PrOH/hexanes = 3/97, 1.0 mL/min, 254 nm, major t_r = 4.347 min (*S*), minor t_r = 4.552 min (*R*)). FTIR (neat, cm^{-1}) ν: 3096, 2949, 2866, 1721, 1490, 1364, 1245, 1145, 1079, 843, 805, 758, 719. HRMS: calcd for $C_{20}H_{30}NO_2$ $[M+H]^+$ 316.2277, found 316.2284.

4aa. Racemic product

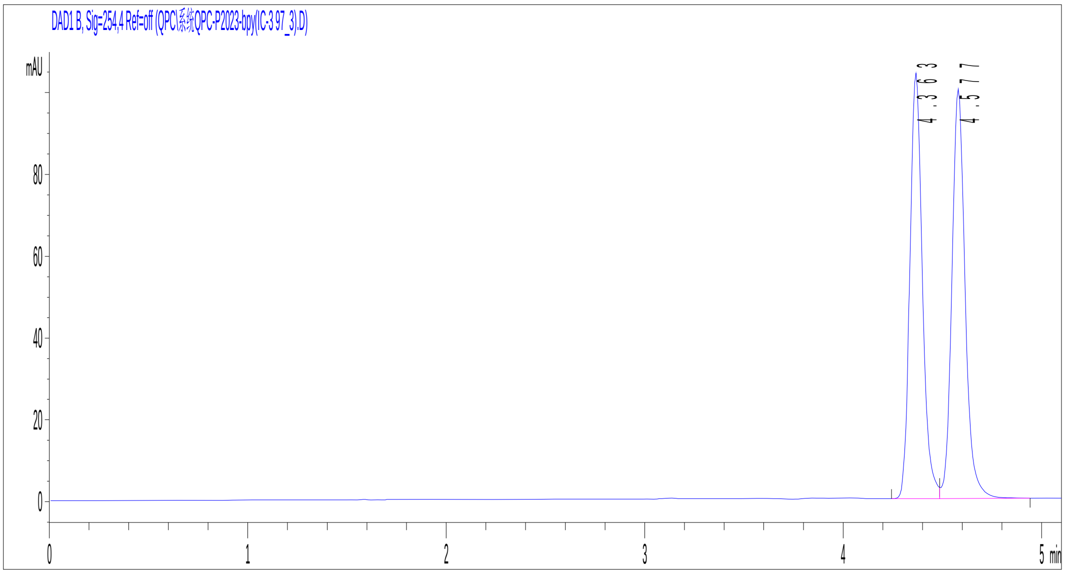

Peak	Ret Time	Type	Width	Area	Height	Area
#	[min]		[min]	mAU*s	[mAU]	%
1	4.363	BV	0.067	455	104	49.463
2	4.577	VB	0.0722	464.8	100	50.537
Totals				919.8	204	

4aa. Enantioenriched product, 83% ee

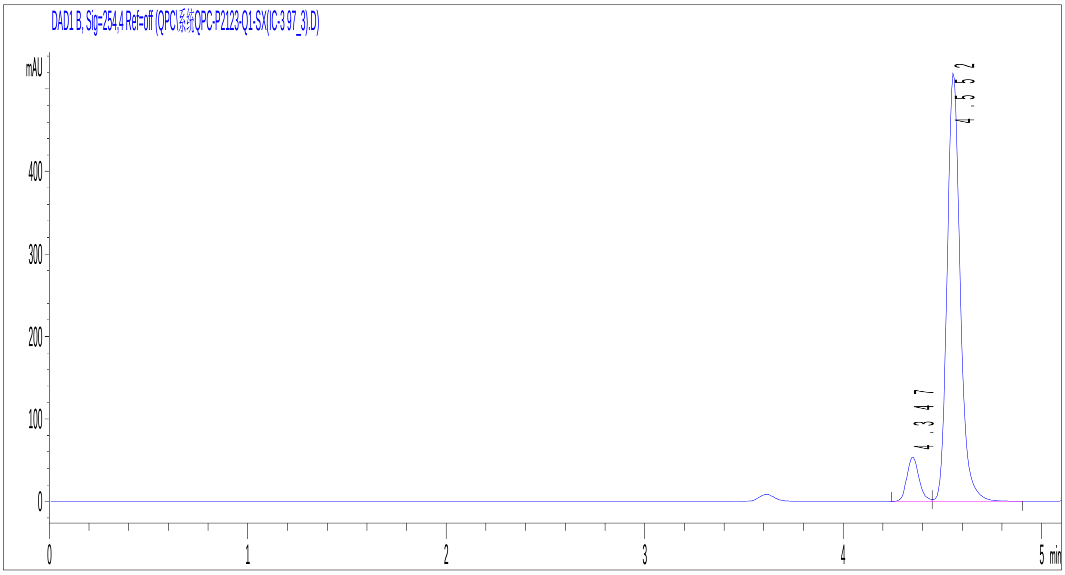

Peak	Ret Time	Type	Width	Area	Height	Area
#	[min]		[min]	mAU*s	[mAU]	%
1	4.347	BV	0.0652	226.8	53.8	8.707
2	4.552	VB	0.0714	2378.2	519.6	91.293
Totals				2605	573.4	

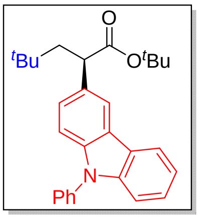

(*R*)-*tert*-butyl 4,4-dimethyl-2-(9-phenyl-9*H*-carbazol-3-yl)pentanoate (4ab)

The reaction was performed following the General Procedure 1 with $NiBr_2$ (2.2 mg, 0.01 mmol) and **L6** (5.4 mg, 0.011 mmol), *tert*-butyl acrylate (15.0 μL, 0.1 mmol, 1.0 equiv.), 3-bromo-9-phenyl-9H-carbazole (64.4 mg, 0.2 mmol, 2.0 equiv.), ^{t}BuBr (54.8 mg, 44.9 μL, 0.4 mmol, 4.0 equiv.), Cy_2NMe (58.5 mg, 64 μL, 0.3 mmol), HEH (75.9 mg, 0.3 mmol) and 4CzIPN (8.0 mg, 0.01 mmol) in DMA (3.0 mL). The crude product was purified by flash chromatography on silica gel (eluted with petroleum ether:EtOAc = 50:1) to give the product (36.8 mg, 86% yield, 86% ee) as a white solid. Melting point: 141.9 – 144.5 °C; R_f = 0.61 (petroleum ether:EtOAc = 20:1). $[\alpha]_D^{25}$ = –15.59 (*c* = 0.357, $CHCl_3$). ^{1}H NMR (400 MHz, $CDCl_3$) δ: 8.14 (d, *J* = 7.7 Hz, 1H), 8.07 (s, 1H), 7.64 – 7.51 (m, 4H), 7.49 – 7.42 (m, 1H), 7.41 – 7.38 (m, 2H), 7.37 – 7.31 (m, 2H), 7.30 – 7.26 (m, 1H), 3.73 (dd, *J* = 9.3, 3.5 Hz, 1H), 2.39 (dd, *J* = 13.9, 9.3 Hz, 1H), 1.63 (dd, *J* = 14.0, 3.5 Hz, 1H), 1.38 (s, 9H), 0.95 (s, 9H). ^{13}C{^{1}H} NMR (101 MHz, $CDCl_3$) δ: 174.60, 141.18, 139.95, 137.82, 133.51, 129.94, 127.45, 127.10, 125.99, 125.93, 123.52, 123.38， 120.43, 119.94, 119.31, 109.86, 109.78, 80.34, 49.25, 47.83, 31.25, 29.70, 27.99. The ee was determined by HPLC with a Daicel Chiralcel AD-H column (^{i}PrOH/hexanes = 1.0/99.0, 1.0 mL/min, 254 nm, major t_r = 5.454 min (*S*), minor t_r = 6.217 min (*R*)). FTIR (neat, cm^{-1}) ν: 2956, 2923, 2851, 1727, 1502, 1365, 1233, 1142, 1026, 803, 744, 730, 698. HRMS: calcd for $C_{29}H_{34}NO_2$ $[M+H]^+$ 428.2590, found 428.2587.

4ab. Racemic product

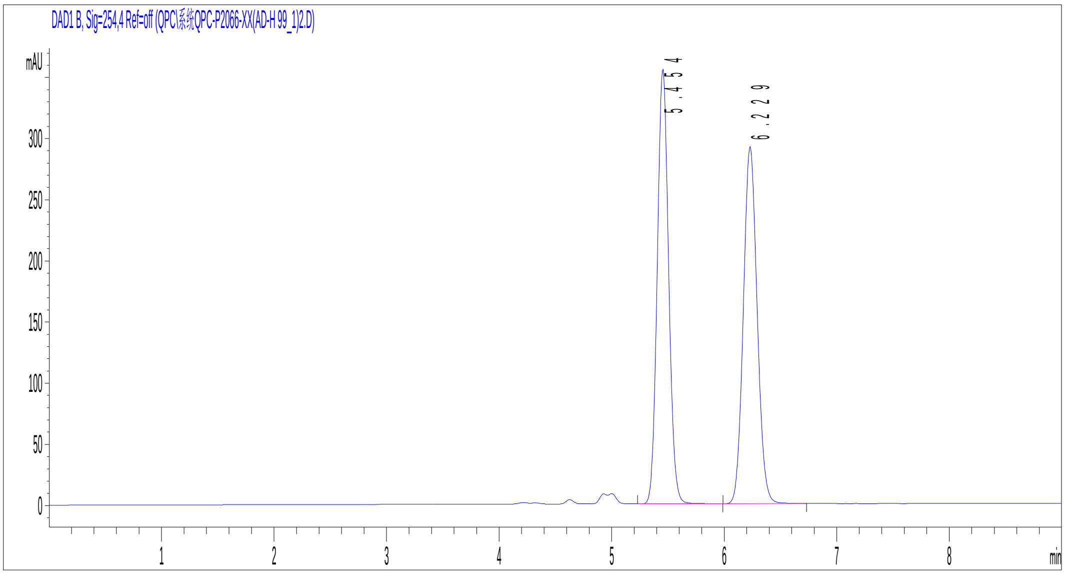

Peak	Ret Time	Type	Width	Area	Height	Area
#	[min]		[min]	mAU*s	[mAU]	%
1	5.454	BB	0.1097	2490.6	355.5	50.017
2	6.229	BB	0.1335	2488.9	291.9	49.983
Totals				4979.5	647.4	

4ab. Enantioenriched product, 86% ee

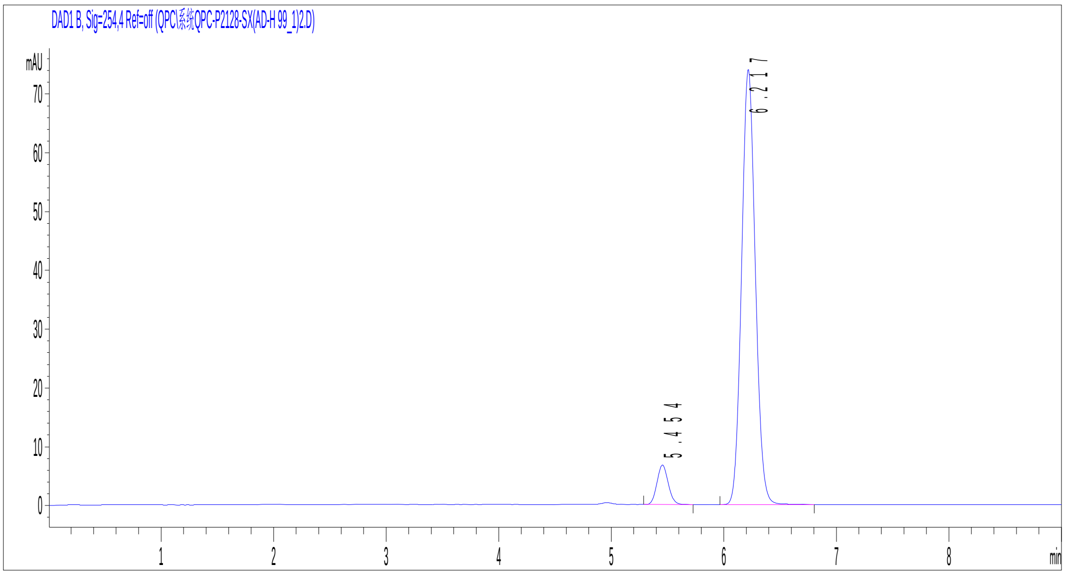

Peak	Ret Time	Type	Width	Area	Height	Area
#	[min]		[min]	mAU*s	[mAU]	%
1	5.454	BB	0.1072	46.7	6.7	6.913
2	6.217	BB	0.1311	629	74.1	93.087
Totals				675.7	80.8	

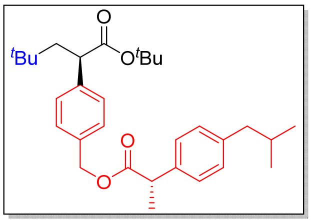

(*R*)-*tert*-butyl 2-(4-((((*S*)-2-(4-isobutylphenyl)propanoyl)oxy)methyl) phenyl)-4,4-dimethylpentanoate (4ac)

The reaction was performed following the General Procedure 1 with $NiBr_2$ (2.2 mg, 0.01 mmol) and **L6** (5.4 mg, 0.011 mmol), *tert*-butyl acrylate (15.0 μL, 0.1 mmol, 1.0 equiv.), 4-bromobenzyl (*S*)-2-(4-isobutylphenyl)propanoate (78.3 mg, 0.2 mmol, 2.0 equiv.), ^{t}BuBr (54.8 mg, 44.9 μL, 0.4 mmol, 4.0 equiv.), Cy_2NMe (58.5 mg, 64 μL, 0.3 mmol), HEH (75.9 mg, 0.3 mmol) and 4CzIPN (8.0 mg, 0.01 mmol) in DMA (3.0 mL). The crude product was purified by flash chromatography on silica gel (eluted with petroleum ether:EtOAc = 50:1) to give the product (39.4 mg, 82% yield, 87% de) as a colorless oil. R_f = 0.61 (petroleum ether:EtOAc = 20:1). $[\alpha]_D^{25}$ = –34.01 (*c* = 0.763, $CHCl_3$). ^{1}H NMR (400 MHz, $CDCl_3$) δ: 7.23 (d, *J* = 8.2 Hz, 2H), 7.20 (d, *J* = 8.1 Hz, 2H), 7.15 (d, *J* = 8.2 Hz, 2H), 7.08 (d, *J* = 8.1 Hz, 2H), 5.12 – 5.02 (m, 2H), 3.74 (q, *J* = 7.2 Hz, 1H), 3.51 (dd, *J* = 9.5, 3.2 Hz, 1H), 2.45 (d, *J* = 7.2 Hz, 2H), 2.25 (dd, *J* = 14.0, 9.5 Hz, 1H), 1.85 (m, 1H), 1.50 (d, *J* = 7.2 Hz, 3H), 1.44 (dd, *J* = 14.0, 3.3 Hz, 1H), 1.37 (s, 9H), 0.91 (s, 3H), 0.90 (s, 9H), 0.89 (s, 3H). ^{13}C{^{1}H} NMR (101 MHz, $CDCl_3$) δ: 174.69, 173.85, 141.60, 140.67, 137.71, 134.54, 129.42, 128.05, 127.80, 127.32, 80.53, 66.16, 49.05, 47.35, 45.22, 45.13, 31.16, 30.32, 29.57, 27.93, 22.49, 18.57. The ee was determined by HPLC with a Daicel Chiralcel AD-H column (^{i}PrOH/hexanes = 0.5/99.5, 1.0 mL/min, 230 nm, major t_r = 9.723 min (*S*), minor t_r = 10.683 min (*R*)). FTIR (neat, cm^{-1}) ν: 3000, 2953, 2868, 1723, 1512, 1455, 1366, 1145, 1092, 845, 777, 522. HRMS: calcd for $C_{31}H_{45}O_4$ $[M+H]^+$ 481.3318, found 481.3326.

4ac. Racemic product

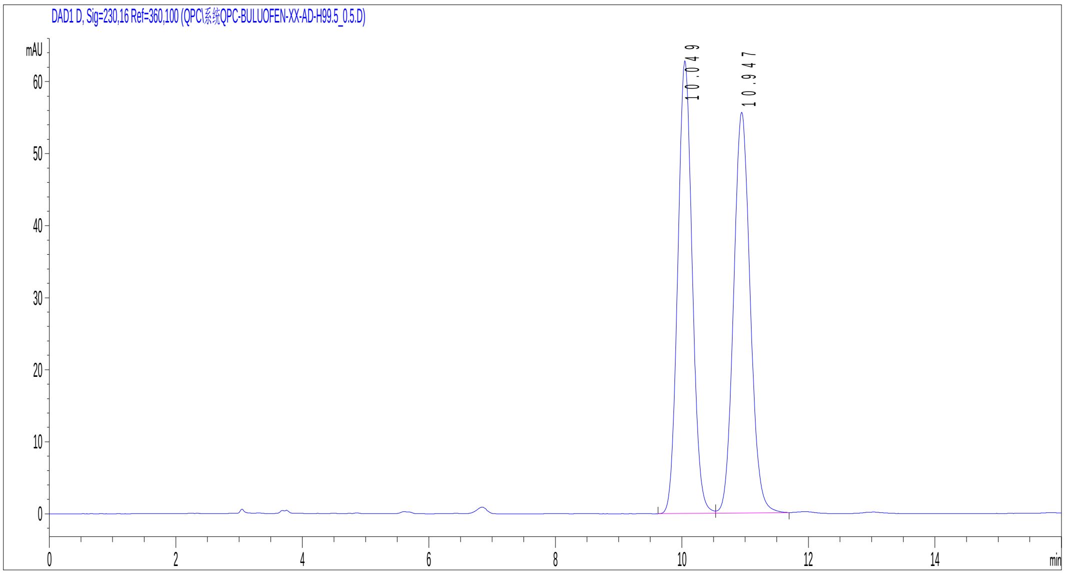

Peak	Ret Time	Type	Width	Area	Height	Area
#	[min]		[min]	mAU*s	[mAU]	%
1	10.049	BV	0.2474	998.4	62.8	49.575
2	10.947	VB	0.2829	1015.5	55.6	50.425
Totals				2013.9	118.4	

4ac. Enantioenriched product, 87% ee

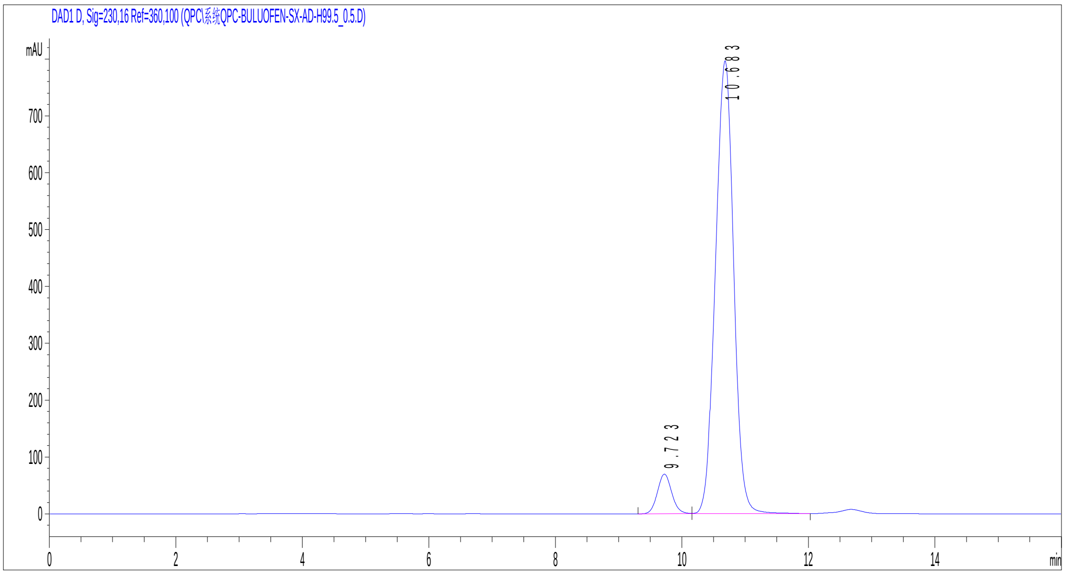

Peak	Ret Time	Type	Width	Area	Height	Area
#	[min]		[min]	mAU*s	[mAU]	%
1	9.723	BV	0.243	1095	69.8	6.436
2	10.683	VB	0.3132	15917.8	796.3	93.564
Totals				17012.8	866.1	

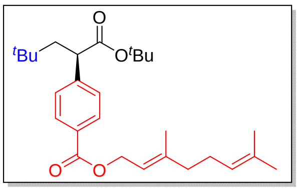

(*R*)-(*E*)-3,7-dimethylocta-2,6-dien-1-yl 4-(1-(tert-butoxy)-4,4-dimethyl-1-oxopentan-2-yl)benzoate (4ad)

The reaction was performed following the General Procedure 1 with $NiBr_2$ (2.2 mg, 0.01 mmol) and **L6** (5.4 mg, 0.011 mmol), *tert*-butyl acrylate (15.0 μL, 0.1 mmol, 1.0 equiv.), (*E*)-3,7-dimethylocta-2,6-dien-1-yl 4-bromobenzoate (67.5 mg, 0.2 mmol, 2.0 equiv.), ^{t}BuBr (54.8 mg, 44.9 μL, 0.4 mmol, 4.0 equiv.), Cy_2NMe (58.5 mg, 64 μL, 0.3 mmol), HEH (75.9 mg, 0.3 mmol) and 4CzIPN (8.0 mg, 0.01 mmol) in DMA (3.0 mL). The crude product was purified by flash chromatography on silica gel (eluted with petroleum ether:EtOAc = 50:1) to give the product (33.9 mg, 79% yield, 91% ee) as a colorless oil. R_f = 0.49 (petroleum ether:EtOAc = 20:1). $[\alpha]_D^{25}$ = –14.94 (*c* = 0.513, $CHCl_3$). ^{1}H NMR (400 MHz, $CDCl_3$) δ: 7.97 (d, *J* = 8.4 Hz, 2H), 7.36 (d, *J* = 8.4 Hz, 2H), 5.48 (t, *J* = 7.2 Hz, 1H), 5.11 (m, 1H), 4.79 (d, *J* = 7.9 Hz, 2H), 3.58 (dd, *J* = 9.0, 3.7 Hz, 1H), 2.27 (dd, *J* = 14.0, 9.1 Hz, 1H), 2.21 – 2.15 (m, 2H), 2.12 (t, *J* = 7.2 Hz, 2H), 1.79 (d, *J* = 1.1 Hz, 3H), 1.67 (s, 3H), 1.60 (s, 3H), 1.49 (dd, *J* = 14.0, 3.7 Hz, 1H), 1.35 (s, 9H), 0.90 (s, 9H). ^{13}C{^{1}H} NMR (101 MHz, $CDCl_3$) δ: 173.30, 166.65, 146.77, 142.85, 132.33, 129.94, 129.08, 127.78, 123.67, 119.35, 80.83, 61.62, 49.47, 46.97, 32.31, 31.20, 29.58, 27.87, 26.76, 25.80, 23.66, 17.79. The ee was determined by HPLC with a Daicel Chiralcel IA column (^{i}PrOH/hexanes = 0.2/99.8, 1.0 mL/min, 210 nm, major t_r = 9.96 min (*S*), minor t_r = 12.26 min (*R*)). FTIR (neat, cm^{-1}) ν: 3435, 2956, 2925, 2856, 1720, 1610, 1367, 1271, 1099, 1020, 844, 804, 756. HRMS: calcd for $C_{28}H_{43}O_4$ $[M+H]^+$ 443.3161, found 443.3152.

4ad. Racemic product

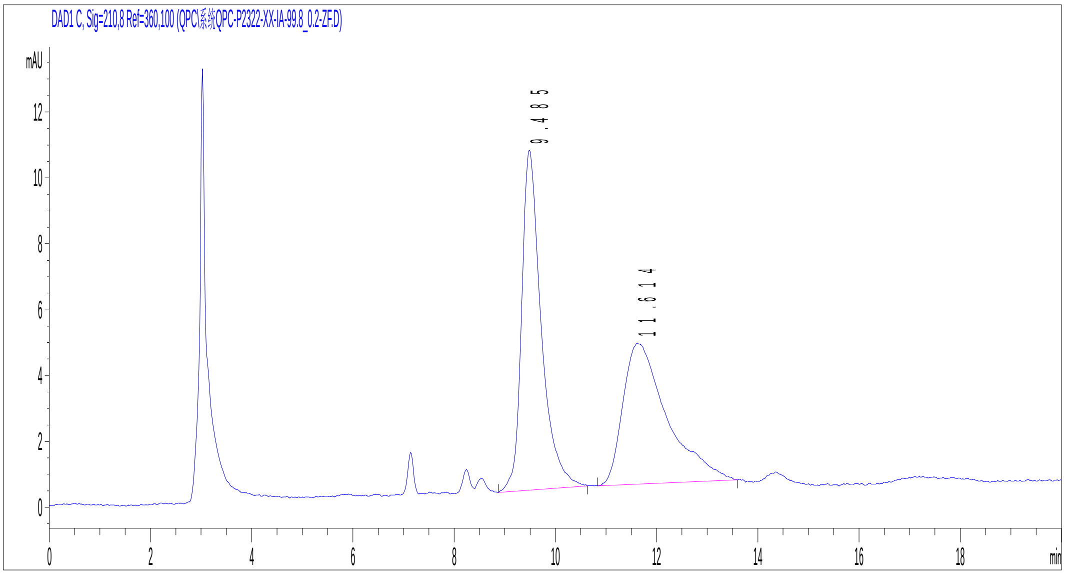

Peak	Ret Time	Type	Width	Area	Height	Area
#	[min]		[min]	mAU*s	[mAU]	%
1	9.485	BB	0.3988	278.4	10.3	51.826
2	11.614	BB	0.7175	258.8	4.3	48.174
Totals				537.2	14.6	

4ad. Enantioenriched product, 91% ee

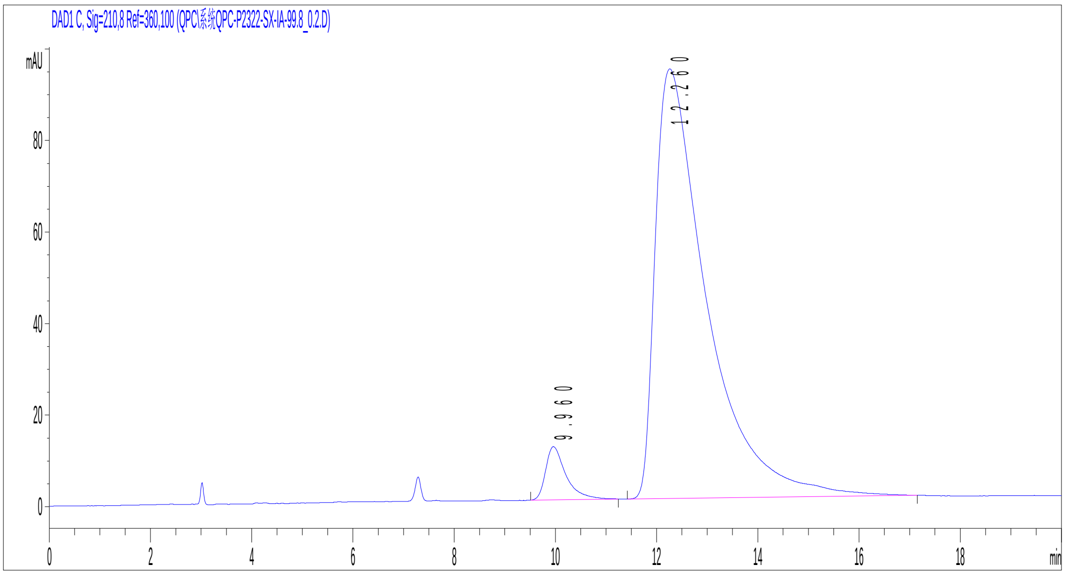

Peak	Ret Time	Type	Width	Area	Height	Area
#	[min]		[min]	mAU*s	[mAU]	%
1	9.96	BB	0.408	325.3	11.6	4.681
2	12.26	BB	0.9952	6624.3	93.9	95.319
Totals				6949.6	105.5	

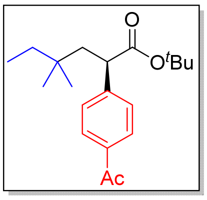

(*R*)-*tert*-butyl 2-(4-acetylphenyl)-4,4-dimethylhexanoate (5a)

The reaction was performed following the General Procedure 1 with $NiBr_2$ (2.2 mg, 0.01 mmol) and **L6** (5.4 mg, 0.011 mmol), *tert*-butyl acrylate (15.0 μL, 0.1 mmol, 1.0 equiv.), 1-(4-bromophenyl)ethan-1-one (39.8 mg, 0.2 mmol, 2.0 equiv.), 2-bromo-2-methylbutane (60.4 mg, 51.1 μL, 0.4 mmol, 4.0 equiv.), Cy_2NMe (58.5 mg, 64 μL, 0.3 mmol), HEH (75.9 mg, 0.3 mmol) and 4CzIPN (8.0 mg, 0.01 mmol) in DMA (3.0 mL). The crude product was purified by flash chromatography on silica gel (eluted with petroleum ether:EtOAc = 50:1) to give the product (23.9 mg, 75% yield, 92% ee) as a white solid. Melting point: 54.0 – 56.1 °C; R_f = 0.48 (petroleum ether:EtOAc = 20:1). $[\alpha]_D^{25}$ = –20.05 (*c* = 0.743, $CHCl_3$). ^{1}H NMR (400 MHz, $CDCl_3$) δ: 7.89 (d, J = 8.0 Hz, 2H), 7.40 (d, *J* = 8.0 Hz, 2H), 3.58 (dd, *J* = 8.8, 3.5 Hz, 1H), 2.59 (s, 3H), 2.26 (dd, *J* = 14.1, 8.9 Hz, 1H), 1.49 (dd, *J* = 14.2, 3.6 Hz, 1H), 1.37 (s, 9H), 1.25 (t, *J* = 7.4 Hz, 2H), 0.84 (d, *J* = 2.8 Hz, 6H), 0.80 (t, *J* = 7.5 Hz, 3H). $^{13}C\{^1H\}$ NMR (101 MHz, $CDCl_3$) δ: 197.98, 173.30, 147.31, 135.79, 128.74, 128.05, 80.92, 49.01, 44.75, 34.47, 33.71, 27.89, 26.73, 26.70, 26.56, 8.48. The ee was determined by HPLC with a Daicel Chiralcel IA column (^{i}PrOH/hexanes = 0.2/99.8, 1.0 mL/min, 250 nm, major t_r = 13.566 min (*S*), minor t_r = 16.274 min (*R*)). FTIR (neat, cm^{-1}) ν: 3436, 2963, 2931, 1729, 1686, 1606, 1267, 1142, 1017, 956, 842, 802, 596. HRMS: calcd for $C_{20}H_{31}O_3$ $[M+H]^+$ 319.2273, found 319.2278.

5a. Racemic product

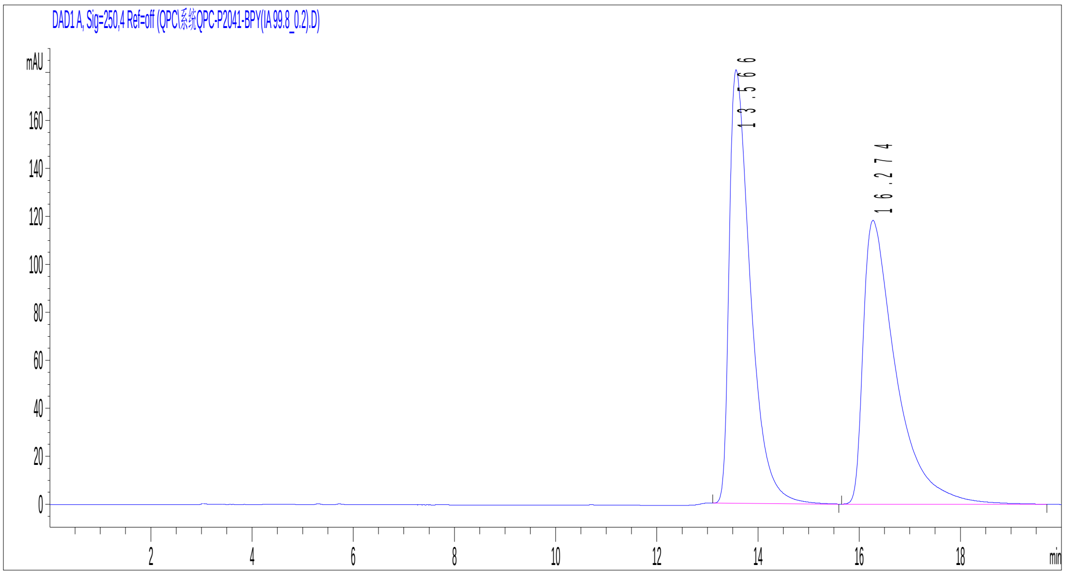

Peak	Ret Time	Type	Width	Area	Height	Area
#	[min]		[min]	mAU*s	[mAU]	%
1	13.566	BB	0.4426	5275.9	180.7	49.822
2	16.274	BB	0.6622	5313.6	118.3	50.178
Totals				10589.5	299.0	

5a. Enantioenriched product, 92% ee

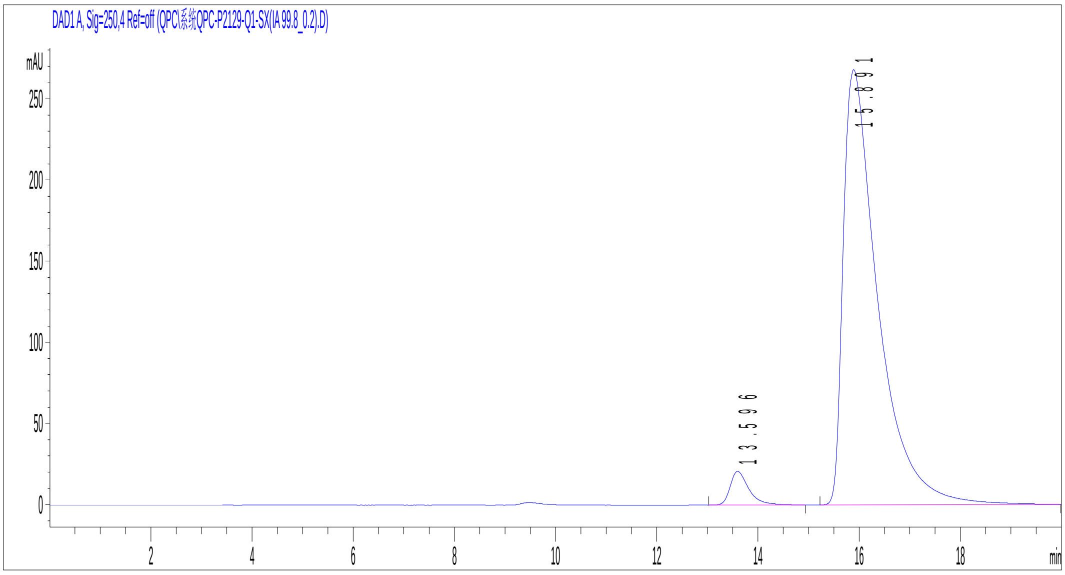

Peak	Ret Time	Type	Width	Area	Height	Area
#	[min]		[min]	mAU*s	[mAU]	%
1	13.596	BB	0.3837	529.9	20.8	3.987
2	15.891	BBA	0.6973	12761.5	268.1	96.013
Totals				13291.4	288.9	

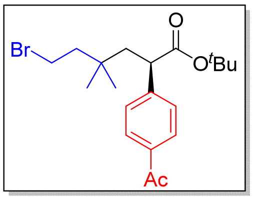

(*R*)-*tert*-butyl 2-(4-acetylphenyl)-6-bromo-4,4-dimethylhexanoate (5b)

The reaction was performed following the General Procedure 1 with $NiBr_2$ (2.2 mg, 0.01 mmol) and **L6** (5.4 mg, 0.011 mmol), tert-butyl acrylate (15.0 μL, 0.1 mmol, 1.0 equiv.), 1-(4-bromophenyl)ethan-1-one (39.8 mg, 0.2 mmol, 2.0 equiv.), 1,3-dibromo-3-methylbutane (92.0 mg, 54.4 μL 0.4 mmol, 4.0 equiv.), Cy_2NMe (58.5 mg, 64 μL, 0.3 mmol), HEH (75.9 mg, 0.3 mmol) and 4CzIPN (8.0 mg, 0.01 mmol) in DMA (3.0 mL). The crude product was purified by flash chromatography on silica gel (eluted with petroleum ether:EtOAc = 50:1) to give the product (27.8 mg, 70% yield, 85% ee) as a colorless oil. R_f = 0.56 (petroleum ether:EtOAc = 20:1). $[\alpha]_D^{25}$ = –2.06 (*c* = 0.21, $CHCl_3$). ^{1}H NMR (400 MHz, $CDCl_3$) δ: 7.91 (d, *J* = 8.4 Hz, 2H), 7.39 (d, *J* = 8.3 Hz, 2H), 3.58 (dd, *J* = 9.0, 3.5 Hz, 1H), 3.45 – 3.27 (m, 2H), 2.60 (s, 3H), 2.32 (dd, *J* = 14.2, 9.1 Hz, 1H), 1.95 – 1.78 (m, 2H), 1.49 (dd, *J* = 14.2, 3.5 Hz, 1H), 1.37 (s, 9H), 0.92 (s, 6H). $^{13}C\{^1H\}$ NMR (101 MHz, $CDCl_3$) δ: 197.88, 172.87, 146.61, 135.99, 128.88, 127.94, 81.33, 48.73, 45.67, 44.87, 35.15, 29.02, 27.90, 26.99, 26.75. The ee was determined by HPLC with a Daicel Chiralcel IA column (^{i}PrOH/hexanes = 3/97, 1.0 mL/min, 254 nm, major t_r = 6.033 min (*S*), minor t_r = 6.395 min (*R*)). FTIR (neat, cm^{-1}) ν: 2986, 2983, 2871, 1727, 1685, 1606, 1367, 1268, 1144, 1017, 957, 842, 596. HRMS: calcd for $C_{20}H_{30}BrO_3$ $[M+H]^+$ 397.1378, found 397.1373.

5b. Racemic product

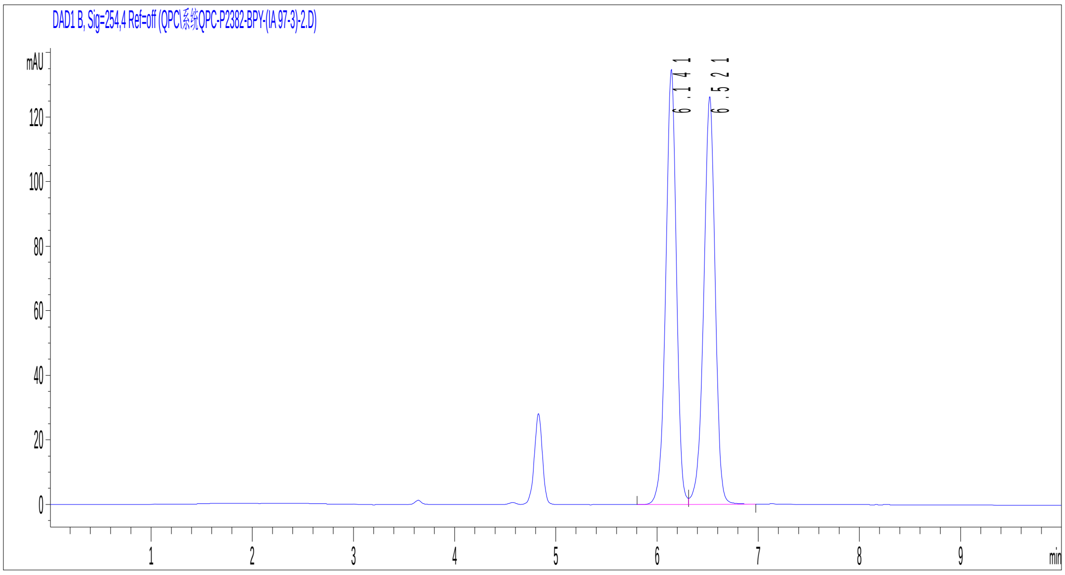

Peak	Ret Time	Type	Width	Area	Height	Area
#	[min]		[min]	mAU*s	[mAU]	%
1	6.141	BV	0.1133	987.5	135	49.856
2	6.521	VB	0.1196	993.2	126.5	50.144
Totals				1980.7	261.5	

5b. Enantioenriched product, 85% ee

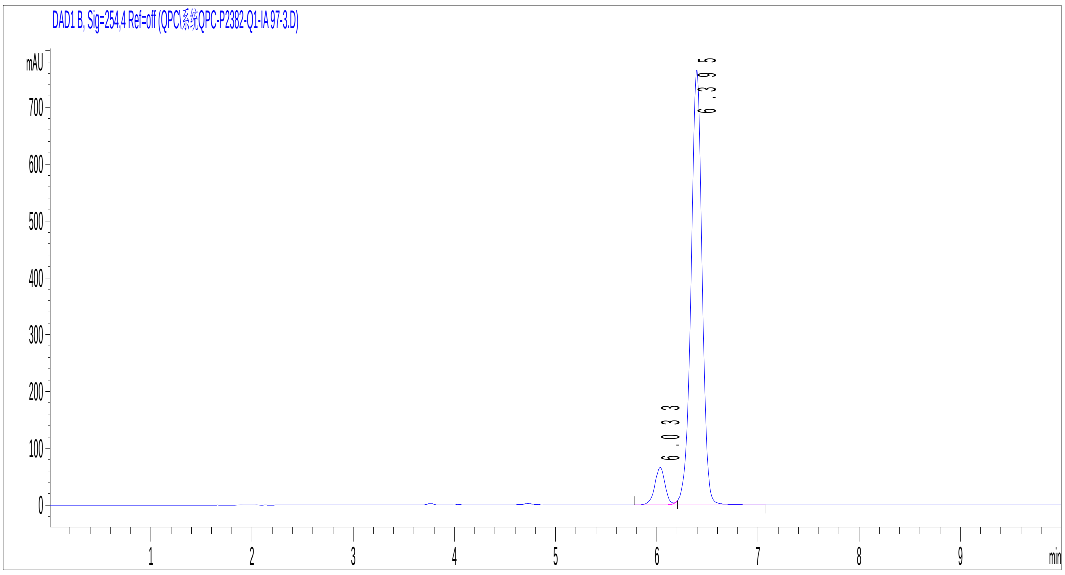

Peak	Ret Time	Type	Width	Area	Height	Area
#	[min]		[min]	mAU*s	[mAU]	%
1	6.033	BV E	0.1082	465.7	66.1	7.393
2	6.395	VB R	0.1168	5833	766.4	92.607
Totals				6298.7	832.5	

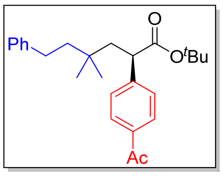

(*R*)-*tert*-butyl 2-(4-acetylphenyl)-4,4-dimethyl-6-phenylhexanoate (5c)

The reaction was performed following the General Procedure 1 with $NiBr_2$ (2.2 mg, 0.01 mmol) and **L6** (5.4 mg, 0.011 mmol), tert-butyl acrylate (15.0 μL, 0.1 mmol, 1.0 equiv.), 1-(4-bromophenyl)ethan-1-one (39.8 mg, 0.2 mmol, 2.0 equiv.), (3-bromo-3-methylbutyl)benzene (90.9 mg, 0.4 mmol, 4.0 equiv.), Cy_2NMe (58.5 mg, 64 μL, 0.3 mmol), HEH (75.9 mg, 0.3 mmol) and 4CzIPN (8.0 mg, 0.01 mmol) in DMA (3.0 mL). The crude product was purified by flash chromatography on silica gel (eluted with petroleum ether:EtOAc = 20:1) to give the product (28.0 mg, 71% yield, 85% ee) as a white solid. Melting point: 75.9 – 77.1 °C; R_f = 0.62 (petroleum ether:EtOAc = 10:1). $[\alpha]_D^{25}$ = –44.40 (c = 0.470, $CHCl_3$). ^1H NMR (400 MHz, $CDCl_3$) δ: 7.90 (d, J = 8.3 Hz, 2H), 7.41 (d, J = 8.3 Hz, 2H), 7.29 – 7.22 (m, 2H), 7.20 – 7.05 (m, 3H), 3.62 (dd, J = 8.8, 3.6 Hz, 1H), 2.59 (s, 3H), 2.58 – 2.44 (m, 2H), 2.35 (dd, J = 14.1, 8.8 Hz, 1H), 1.60 (dd, J = 11.2, 2.9 Hz, 1H), 1.57 – 1.49 (m, 2H), 1.34 (s, 9H), 0.95 (s, 6H). $^{13}C\{^1H\}$ NMR (101 MHz, $CDCl_3$) δ: 197.93, 173.20, 147.10, 143.13, 135.87, 128.81, 128.44, 128.38, 128.07, 125.74, 81.06, 48.97, 45.08, 44.56, 33.89, 30.71, 27.88, 27.20, 27.02, 26.74. The ee was determined by HPLC with a Daicel Chiralcel AD-H column (^{i}PrOH/hexanes = 3/97, 1.0 mL/min, 254 nm, major t_r = 6.167 min (S), minor t_r = 6.425 min (R)). FTIR (neat, cm^{-1}) ν: 3026, 3002, 2959, 2930, 1728, 1668, 1367, 1267, 1017, 957, 802, 699. HRMS: calcd for $C_{26}H_{35}O_3$ $[M+H]^+$ 395.2586, found 395.2581.

5c. Racemic product

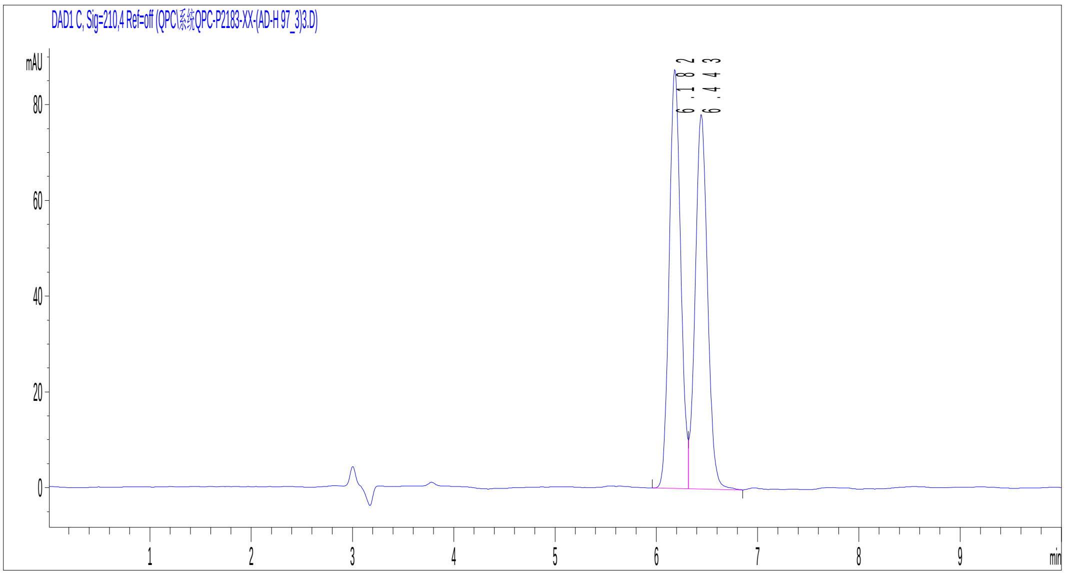

Peak	Ret Time	Type	Width	Area	Height	Area
#	[min]		[min]	mAU*s	[mAU]	%
1	6.182	BV	0.1218	688.8	87.5	50.999
2	6.443	VB	0.1306	661.8	78.3	49.001
Totals				1350.6	165.8	

5c. Enantioenriched product, 86% ee

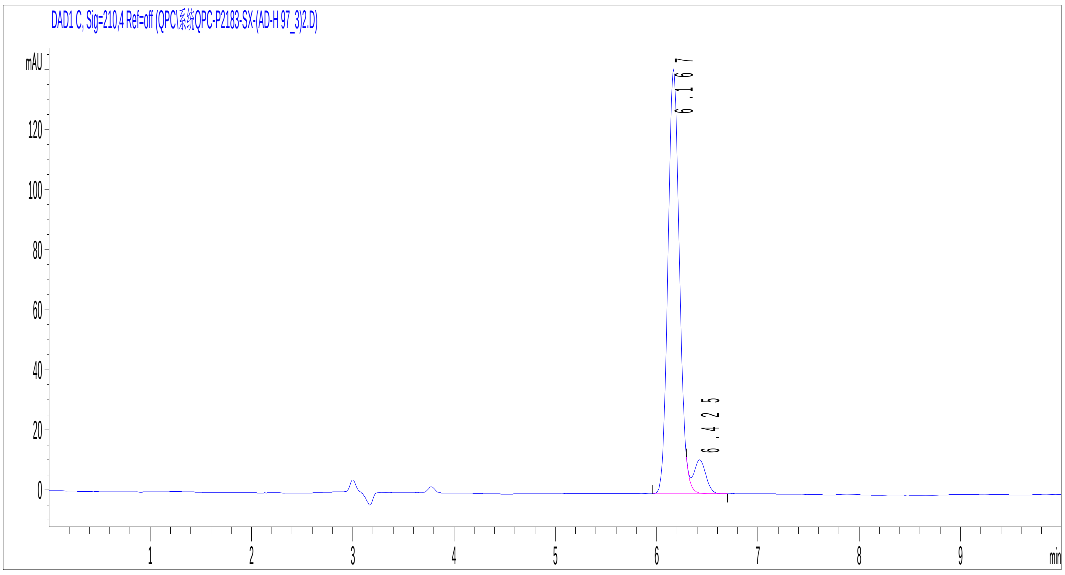

Peak	Ret Time	Type	Width	Area	Height	Area
#	[min]		[min]	mAU*s	[mAU]	%
1	6.167	BV R	0.1224	1118.6	141.2	92.778
2	6.425	VB E	0.1221	87.1	11	7.222
Totals				1205.7	152.2	

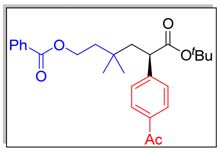

(*R*)-5-(4-acetylphenyl)-6-(*tert*-butoxy)-3,3-dimethyl-6-oxohexyl benzoate (5d)

The reaction was performed following the General Procedure 1 with $NiBr_2$ (2.2 mg, 0.01 mmol) and **L6** (5.4 mg, 0.011 mmol), *tert*-butyl acrylate (15.0 μL, 0.1 mmol, 1.0 equiv.), 1-(4-bromophenyl)ethan-1-one (39.8 mg, 0.2 mmol, 2.0 equiv.), 3-bromo-3-methylbutyl benzoate (108.5 mg, 0.4 mmol, 4.0 equiv.), Cy_2NMe (58.5 mg, 64 μL, 0.3 mmol), HEH (75.9 mg, 0.3 mmol) and 4CzIPN (8.0 mg, 0.01 mmol) in DMA (3.0 mL). The crude product was purified by flash chromatography on silica gel (eluted with petroleum ether:EtOAc = 10:1) to give the product (34.6 mg, 79% yield, 83% ee) as a white solid. Melting point: 60.0 – 61.6 °C; R_f = 0.42 (petroleum ether:EtOAc = 5:1). $[\alpha]_D^{25}$ = –23.41 (*c* = 0.517, $CHCl_3$). ^{1}H NMR (400 MHz, $CDCl_3$) δ: 8.00 (m, 2H), 7.92 – 7.86 (m, 2H), 7.58 – 7.52 (m, 1H), 7.45 – 7.38 (m, 4H), 4.36 (t, *J* = 7.3 Hz, 2H), 3.64 (dd, *J* = 9.1, 3.5 Hz, 1H), 2.58 (s, 3H), 2.39 (dd, *J* = 14.2, 9.1 Hz, 1H), 1.75 (t, *J* = 7.3 Hz, 2H), 1.61 (dd, *J* = 14.3, 3.6 Hz, 1H), 1.37 (s, 9H), 1.00 (d, *J* = 2.8 Hz, 6H). ^{13}C{^{1}H} NMR (101 MHz, $CDCl_3$) δ: 197.91, 173.02, 166.72, 146.76, 135.91, 132.99, 130.39, 129.60, 128.82, 128.44, 128.02, 81.18, 62.17, 48.90, 45.49, 40.17, 33.19, 27.87, 27.29, 27.24, 26.72. The ee was determined by HPLC with a Daicel Chiralcel IC-3 column (^{i}PrOH/hexanes = 10/90, 1.0 mL/min, 250 nm, major t_r = 16.157 min (*S*), minor t_r = 17.55 min (*R*)). FTIR (neat, cm^{-1}) ν: 2962, 2926, 2871, 1720, 1684, 1605, 1367, 1271, 1142, 1026, 810, 712, 597. HRMS: calcd for $C_{27}H_{35}O_5$ $[M+H]^+$ 439.2484, found 439.2479.

5d. Racemic product

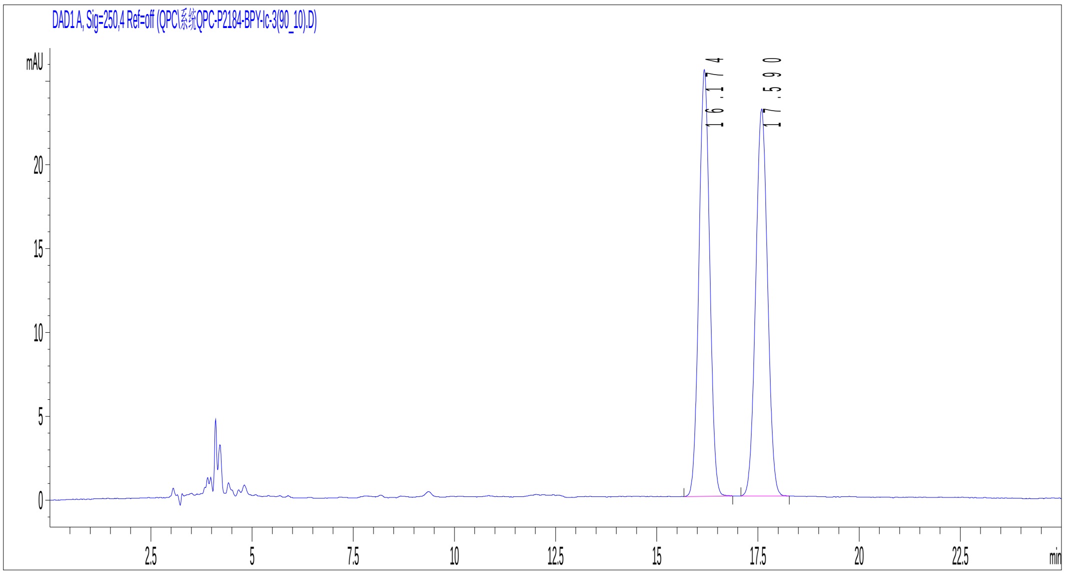

Peak	Ret Time	Type	Width	Area	Height	Area
#	[min]		[min]	mAU*s	[mAU]	%
1	16.174	BB	0.2857	470.7	25.5	50.008
2	17.59	BB	0.3198	470.6	23.1	49.992
Totals				941.3	48.6	

5d. Enantioenriched product, 83% ee

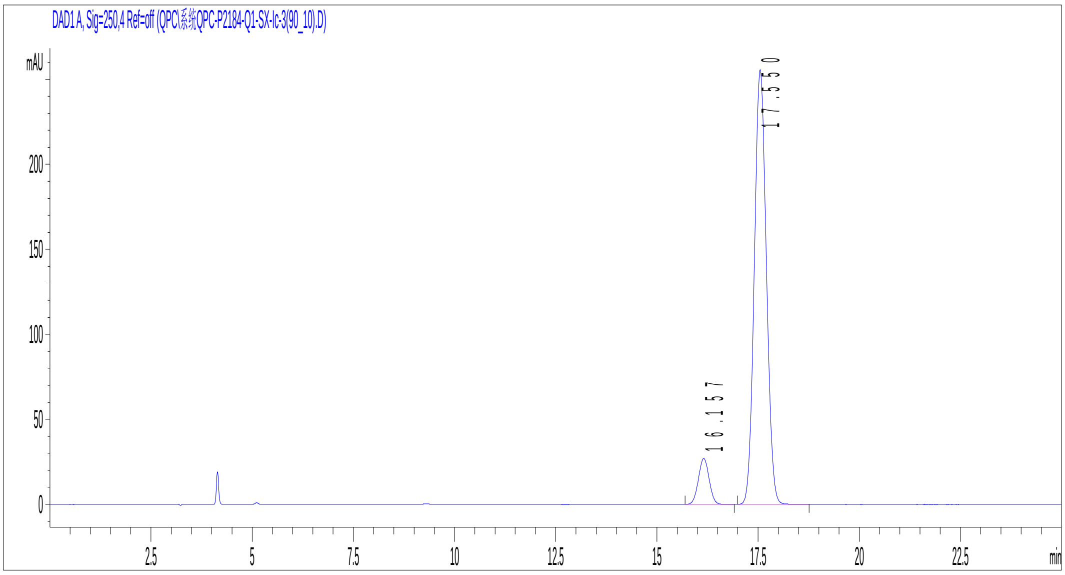

Peak	Ret Time	Type	Width	Area	Height	Area
#	[min]		[min]	mAU*s	[mAU]	%
1	16.157	BB	0.2889	497.8	27	8.622
2	17.55	BB	0.3228	5276.2	255.6	91.378
Totals				5774	282.6	

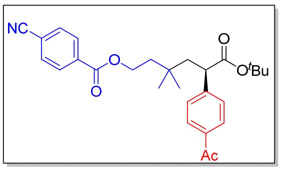

(*R*)-5-(4-acetylphenyl)-6-(*tert*-butoxy)-3,3-dimethyl-6-oxohexyl 4-cyanobenzoate (5e)

The reaction was performed following the General Procedure 1 with $NiBr_2$ (2.2 mg, 0.01 mmol) and **L6** (5.4 mg, 0.011 mmol), *tert*-butyl acrylate (15.0 μL, 0.1 mmol, 1.0 equiv.), 1-(4-bromophenyl)ethan-1-one (39.8 mg, 0.2 mmol, 2.0 equiv.), 3-bromo-3-methylbutyl 4-cyanobenzoate (118.5 mg, 0.4 mmol, 4.0 equiv.), Cy_2NMe (58.5 mg, 64 μL, 0.3 mmol), HEH (75.9 mg, 0.3 mmol) and 4CzIPN (8.0 mg, 0.01 mmol) in DMA (3.0 mL). The crude product was purified by flash chromatography on silica gel (eluted with petroleum ether:EtOAc = 10:1) to give the product (33.4 mg, 72% yield, 83% ee) as a colorless oil. R_f = 0.67 (petroleum ether:EtOAc = 2:1). $[\alpha]_D^{25}$ = –19.98 (*c* = 1.063, $CHCl_3$). ^{1}H NMR (400 MHz, $CDCl_3$) δ: 8.10 (d, *J* = 8.7 Hz, 2H), 7.90 (d, *J* = 8.4 Hz, 2H), 7.73 (d, *J* = 8.8 Hz, 2H), 7.40 (d, *J* = 8.3 Hz, 2H), 4.40 (t, *J* = 7.4 Hz, 2H), 3.63 (dd, *J* = 9.1, 3.5 Hz, 1H), 2.59 (s, 3H), 2.39 (dd, *J* = 14.2, 9.1 Hz, 1H), 1.75 (t, *J* = 7.4 Hz, 2H), 1.59 (dd, *J* = 14.2, 3.6 Hz, 1H), 1.36 (s, 9H), 1.00 (d, *J* = 3.6 Hz, 6H). $^{13}C\{^1H\}$ NMR (101 MHz, $CDCl_3$) δ: 197.78, 172.92, 165.01, 146.62, 135.99, 134.21, 132.30, 130.11, 128.83, 127.97, 118.07, 116.43, 81.22, 63.01, 48.86, 45.40, 40.11, 33.17, 27.86, 27.30, 27.15, 26.69. The ee was determined by HPLC with a Daicel Chiralcel IA column (^{i}PrOH/hexanes = 5/95, 1.0 mL/min, 254 nm, major t_r = 23.6 min (*S*), minor t_r = 25.115 min (*R*)). FTIR (neat, cm^{-1}) ν: 3054, 2963, 2930, 2231, 1724, 1684, 1367, 1273, 1018, 953, 862, 768, 692. HRMS: calcd for $C_{28}H_{34}NO_5$ $[M+H]^+$ 464.2437, found 464.2434.

5e.Racemic product

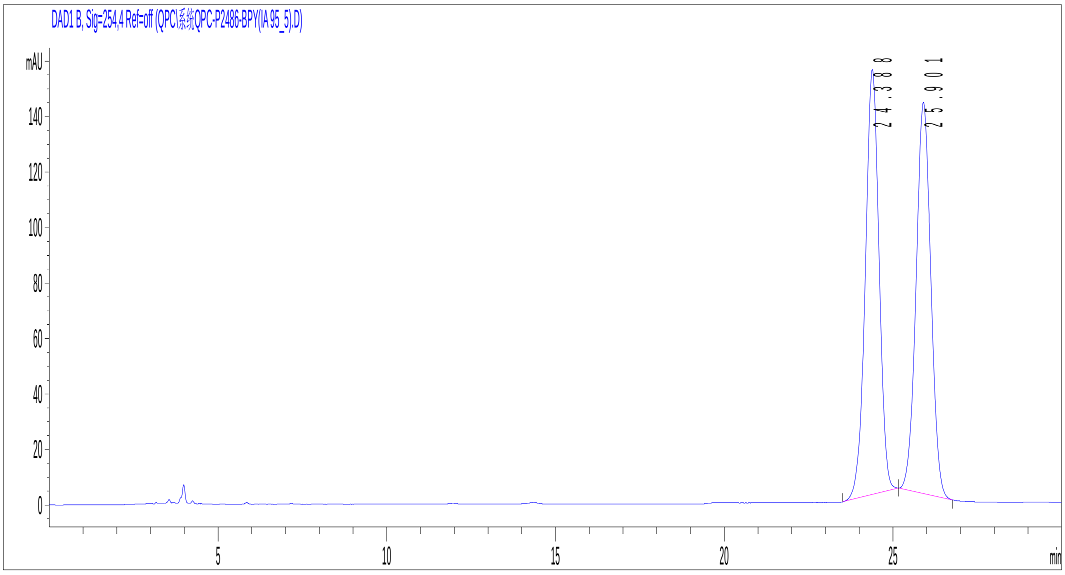

Peak	Ret Time	Type	Width	Area	Height	Area
#	[min]		[min]	mAU*s	[mAU]	%
1	24.388	BB	0.4364	4339.6	153.2	50.270
2	25.901	BB	0.471	4293	141.1	49.730
Totals				8632.6	294.3	

5e. Enantioenriched product, 83% ee

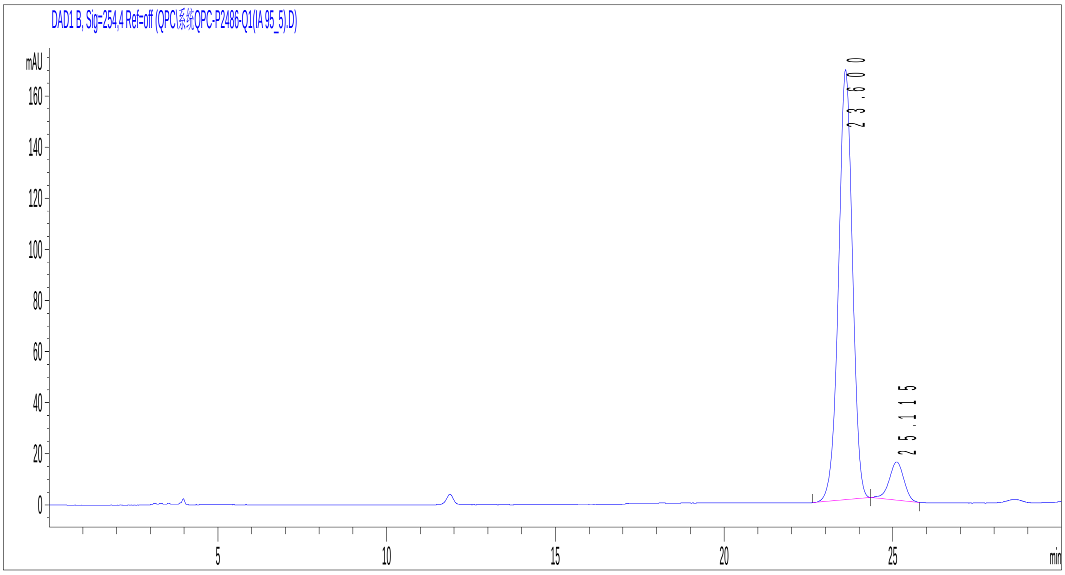

Peak	Ret Time	Type	Width	Area	Height	Area
#	[min]		[min]	mAU*s	[mAU]	%
1	23.6	BB	0.4428	4826.6	168.2	91.517
2	25.115	BB	0.4612	447.4	14.9	8.483
Totals				5274	183.1	

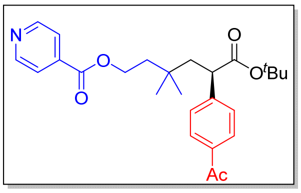

(*R*)-5-(4-acetylphenyl)-6-(*tert*-butoxy)-3,3-dimethyl-6-oxohexyl isonicotinate (5f)

The reaction was performed following the General Procedure 1 with $NiBr_2$ (2.2 mg, 0.01 mmol) and **L6** (5.4 mg, 0.011 mmol), *tert*-butyl acrylate (15.0 μL, 0.1 mmol, 1.0 equiv.), 1-(4-bromophenyl)ethan-1-one (39.8 mg, 0.2 mmol, 2.0 equiv.), 3-bromo-3-methylbutyl isonicotinate (108.9 mg, 0.4 mmol, 4.0 equiv.), Cy_2NMe (58.5 mg, 64 μL, 0.3 mmol), HEH (75.9 mg, 0.3 mmol) and 4CzIPN (8.0 mg, 0.01 mmol) in DMA (3.0 mL). The crude product was purified by flash chromatography on silica gel (eluted with petroleum ether:EtOAc = 5:1) to give the product (26.4 mg, 60% yield, 85% ee) as a colorless oil. R_f = 0.52 (petroleum ether:EtOAc = 2:1). $[\alpha]_D^{25}$ = –20.29 (*c* = 0.46, $CHCl_3$). ^1H NMR (400 MHz, $CDCl_3$) δ: 8.78 – 8.74 (m, 2H), 7.90 (d, *J* = 8.4 Hz, 2H), 7.82 – 7.78 (m, 2H), 7.41 (d, *J* = 8.4 Hz, 2H), 4.40 (t, *J* = 7.4 Hz, 2H), 3.63 (dd, *J* = 9.2, 3.4 Hz, 1H), 2.59 (s, 3H), 2.39 (dd, *J* = 14.2, 9.2 Hz, 1H), 1.75 (t, *J* = 7.4 Hz, 2H), 1.59 (dd, *J* = 14.2, 3.5 Hz, 1H), 1.37 (s, 9H), 1.00 (d, *J* = 3.5 Hz, 6H). $^{13}C\{^1H\}$ NMR (101 MHz, $CDCl_3$) δ: 197.85, 172.94, 165.21, 150.71, 146.63, 137.53, 135.98, 128.84, 127.97, 122.88, 81.25, 63.04, 48.85, 45.40, 40.05, 33.17, 27.87, 27.30, 27.16, 26.73. The ee was determined by HPLC with a Daicel Chiralcel IA column (^{i}PrOH/hexanes = 10/90, 1.0 mL/min, 254 nm, major t_r = 13.09 min (*S*), minor t_r =14.041 min (*R*)). FTIR (neat, cm^{-1}) ν: 3053, 2962, 2926, 2871, 1727, 1685, 1367, 1142, 1018, 801, 758, 707, 677. HRMS: calcd for $C_{26}H_{33}NaNO_5$ $[M+Na]^+$ 462.2256, found 462.2252.

5f. Racemic product

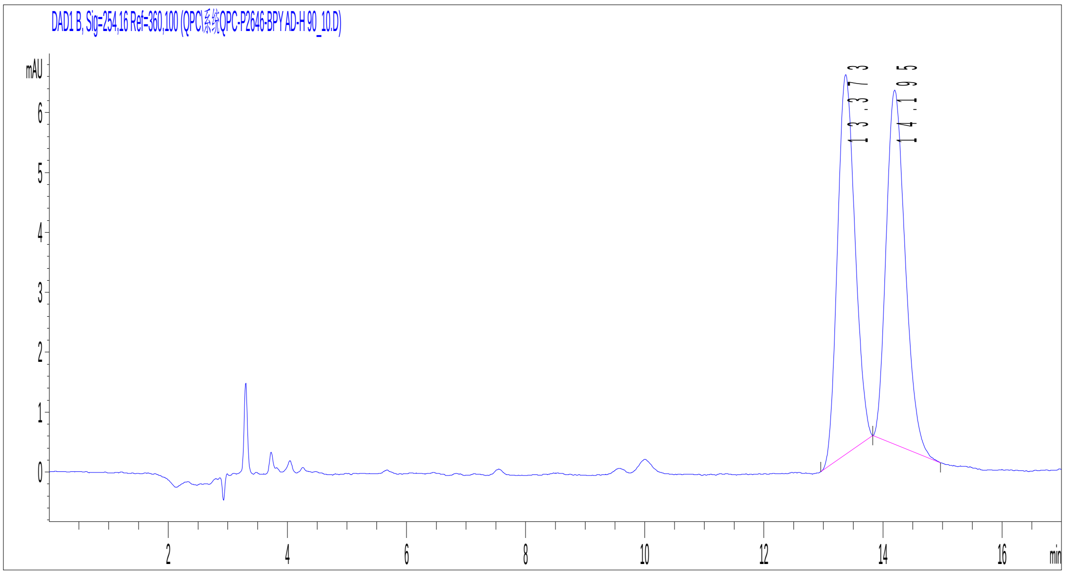

Peak	Ret Time	Type	Width	Area	Height	Area
#	[min]		[min]	mAU*s	[mAU]	%
1	13.373	BB	0.3171	129.9	6.3	49.659
2	14.195	BB	0.3381	131.7	5.9	50.341
Totals				261.6	12.2	

5f. Enantioenriched product, 85% ee

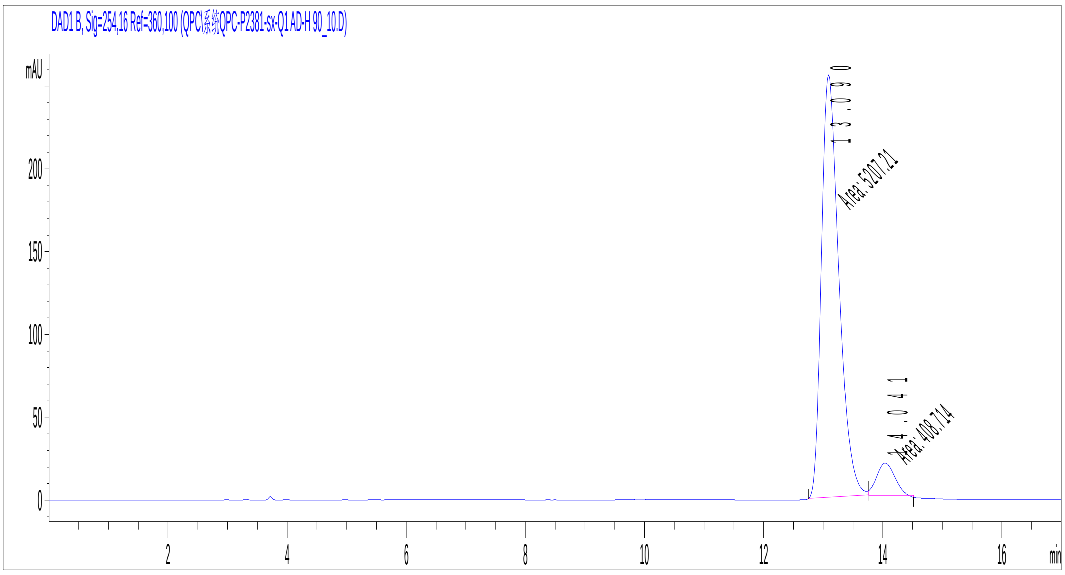

Peak	Ret Time	Type	Width	Area	Height	Area
#	[min]		[min]	mAU*s	[mAU]	%
1	13.09	MM	0.3404	5207.2	255	92.722
2	14.041	MM	0.3499	408.7	19.5	7.278
Totals				5615.9	274.5	

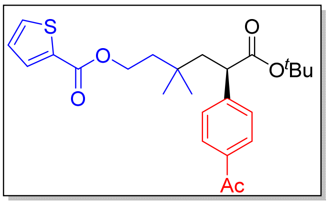

(*R*)-5-(4-acetylphenyl)-6-(*tert*-butoxy)-3,3-dimethyl-6-oxohexyl thiophene-2-carboxylate (5g)

The reaction was performed following the General Procedure 1 with $NiBr_2$ (2.2 mg, 0.01 mmol) and **L6** (5.4 mg, 0.011 mmol), *tert*-butyl acrylate (15.0 μL, 0.1 mmol, 1.0 equiv.), 1-(4-bromophenyl)ethan-1-one (39.8 mg, 0.2 mmol, 2.0 equiv.), 3-bromo-3-methylbutyl thiophene-2-carboxylate (110.9 mg, 0.4 mmol, 4.0 equiv.), Cy_2NMe (58.5 mg, 64 μL, 0.3 mmol), HEH (75.9 mg, 0.3 mmol) and 4CzIPN (8.0 mg, 0.01 mmol) in DMA (3.0 mL). The crude product was purified by flash chromatography on silica gel (eluted with petroleum ether:EtOAc = 10:1) to give the product (32.0 mg, 72% yield, 83% ee) as a white solid. Melting point: 64.4 – 65.2 °C; R_f = 0.59 (petroleum ether:EtOAc = 5:1). $[\alpha]_D^{25}$ = –20.79 (*c* = 0.587, $CHCl_3$). ^{1}H NMR (400 MHz, $CDCl_3$) δ: 7.89 (d, *J* = 8.4 Hz, 2H), 7.75 (dd, *J* = 3.7, 1.3 Hz, 1H), 7.54 (dd, *J* = 5.0, 1.3 Hz, 1H), 7.41 (d, *J* = 8.4 Hz, 2H), 7.08 (dd, *J* = 5.0, 3.8 Hz, 1H), 4.33 (t, *J* = 7.3 Hz, 2H), 3.63 (dd, *J* = 9.1, 3.4 Hz, 1H), 2.58 (s, 3H), 2.37 (dd, *J* = 14.2, 9.1 Hz, 1H), 1.72 (t, *J* = 7.3 Hz, 2H), 1.59 (dd, *J* = 14.2, 3.5 Hz, 1H), 1.37 (s, 9H), 0.99 (d, *J* = 2.8 Hz, 6H). $^{13}C\{^1H\}$ NMR (101 MHz, $CDCl_3$) δ: 197.91, 173.01, 162.34, 146.75, 135.91, 133.99, 133.41, 132.40, 128.82, 128.02, 127.83, 81.18, 62.34, 48.88, 45.50, 40.07, 33.17, 27.88, 27.28, 27.21, 26.73. The ee was determined by HPLC with a Daicel Chiralcel IC-3 column (^{i}PrOH/hexanes = 15/85, 1.0 mL/min, 254 nm, major t_r = 15.451 min (*S*), minor t_r = 17.189 min (*R*)). FTIR (neat, cm^{-1}) ν: 2962, 2926, 2871, 1711, 1684, 1418, 1261, 1142, 1096, 1017, 957, 801, 751. HRMS: calcd for $C_{25}H_{33}O_5S$ $[M+H]^+$ 445.2049, found 445.2041.

5g. Racemic product

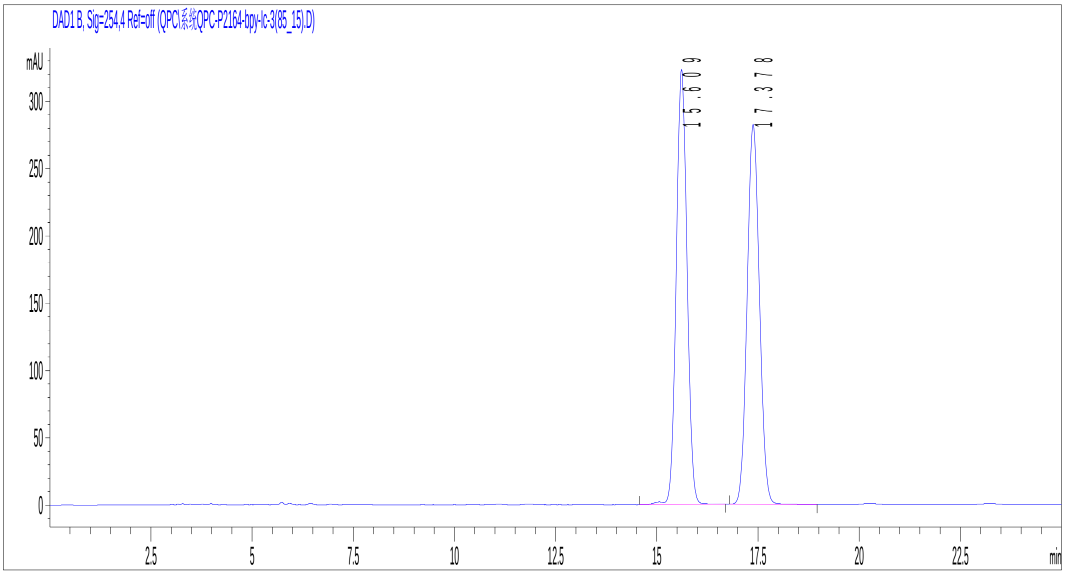

Peak	Ret Time	Type	Width	Area	Height	Area
#	[min]		[min]	mAU*s	[mAU]	%
1	15.609	VB R	0.2895	5997.2	323	50.182
2	17.378	BB	0.3302	5953.8	282.2	49.818
Totals				11951	605.2	

5g. Enantioenriched product, 83% ee

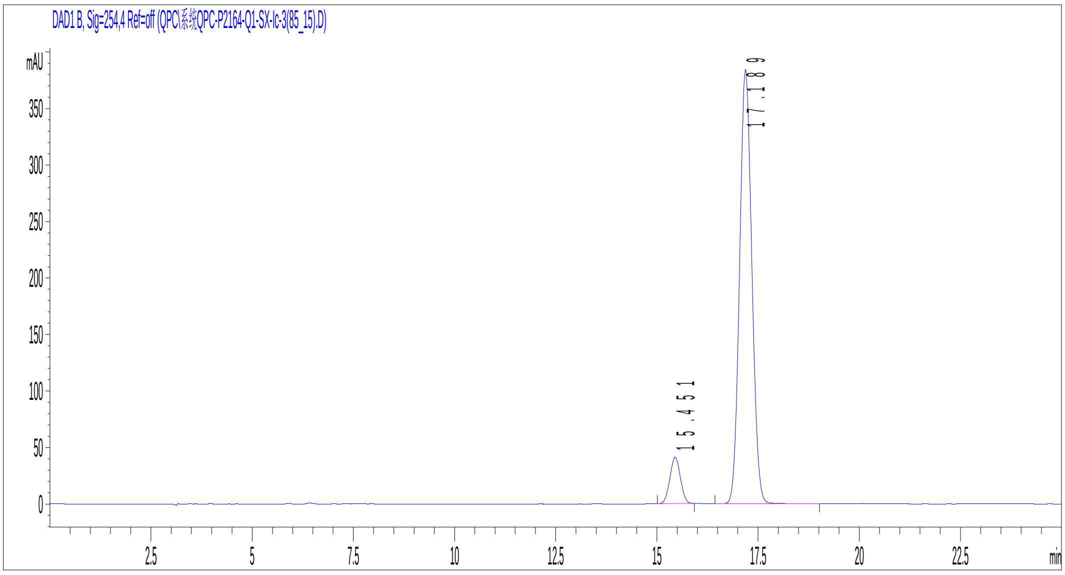

Peak	Ret Time	Type	Width	Area	Height	Area
#	[min]		[min]	mAU*s	[mAU]	%
1	15.451	BB	0.2851	740.4	40.9	8.400
2	17.189	BB	0.3271	8074	384.3	91.600
Totals				8814.4	425.2	

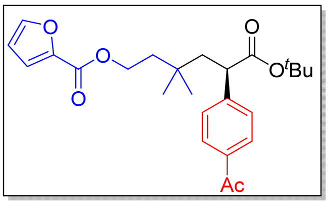

(*R*)-5-(4-acetylphenyl)-6-(*tert*-butoxy)-3,3-dimethyl-6-oxohexyl furan-2-carboxylate (5h)

The reaction was performed following the General Procedure 1 with $NiBr_2$ (2.2 mg, 0.01 mmol) and **L6** (5.4 mg, 0.011 mmol), *tert*-butyl acrylate (15.0 μL, 0.1 mmol, 1.0 equiv.), 1-(4-bromophenyl)ethan-1-one (39.8 mg, 0.2 mmol, 2.0 equiv.), 3-bromo-3-methylbutyl furan-2-carboxylate (110.8 mg, 0.4 mmol, 4.0 equiv.), Cy_2NMe (58.5 mg, 64 μL, 0.3 mmol), HEH (75.9 mg, 0.3 mmol) and 4CzIPN (8.0 mg, 0.01 mmol) in DMA (3.0 mL). The crude product was purified by flash chromatography on silica gel (eluted with petroleum ether:EtOAc = 10:1) to give the product (34.2 mg, 80% yield, 83% ee) as a white solid. Melting point: 64.5 – 66.0 °C; R_f = 0.57 (petroleum ether:EtOAc = 5:1). $[\alpha]_D^{25}$ = –23.19 (*c* = 0.667, $CHCl_3$). ^{1}H NMR (400 MHz, $CDCl_3$) δ: 7.89 (d, *J* = 8.4 Hz, 2H), 7.75 (dd, *J* = 3.8, 1.3 Hz, 1H), 7.54 (dd, *J* = 4.9, 1.3 Hz, 1H), 7.41 (d, *J* = 8.4 Hz, 2H), 7.08 (dd, *J* = 5.0, 3.7 Hz, 1H), 4.33 (t, *J* = 7.3 Hz, 2H), 3.63 (dd, *J* = 9.1, 3.5 Hz, 1H), 2.58 (s, 3H), 2.37 (dd, *J* = 14.2, 9.2 Hz, 1H), 1.72 (t, *J* = 7.3 Hz, 2H), 1.59 (dd, *J* = 14.2, 3.5 Hz, 1H), 1.37 (s, 9H), 0.99 (d, *J* = 2.7 Hz, 6H). ^{13}C{^{1}H} NMR (101 MHz, $CDCl_3$) δ: 197.91, 173.00, 162.33, 146.75, 135.91, 133.99, 133.41, 132.40, 128.82, 128.02, 127.83, 81.18, 62.34, 48.88, 45.50, 40.07, 33.17, 27.88, 27.28, 27.22, 26.73. The ee was determined by HPLC with a Daicel Chiralcel IC-3 column (^{i}PrOH/hexanes = 15/85, 1.0 mL/min, 254 nm, major t_r = 15.361 min (*S*), minor t_r = 17.087 min (*R*)). FTIR (neat, cm^{-1}) ν: 3104, 2962, 2927, 2871, 1711, 1684, 1605, 1418, 1262, 1096, 957, 842, 802, 751. HRMS: calcd for $C_{25}H_{33}O_6$ $[M+H]^+$ 429.2277, found 429.2273.

5h. Racemic product

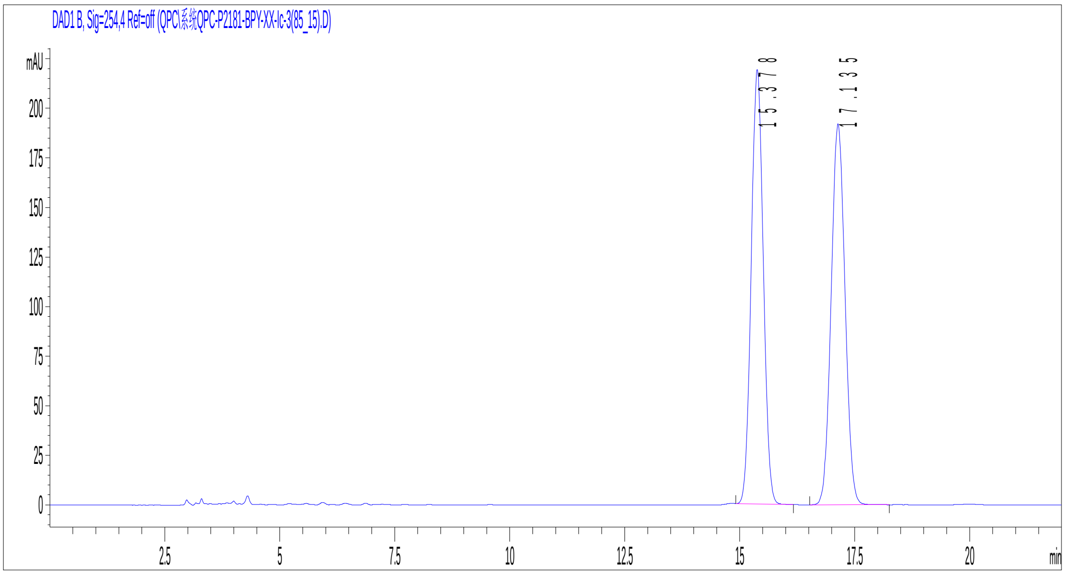

Peak	Ret Time	Type	Width	Area	Height	Area
#	[min]		[min]	mAU*s	[mAU]	%
1	15.378	BB	0.2847	3995.3	219.1	49.820
2	17.135	BB	0.3271	4024.1	192.1	50.180
Totals				8019.4	411.2	

5h. Enantioenriched product, 83% ee

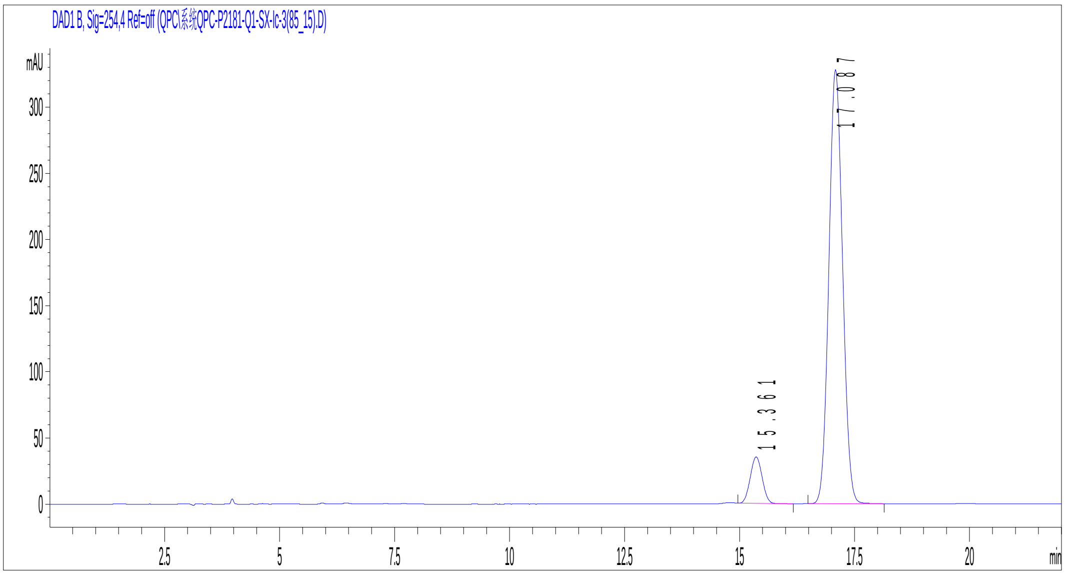

Peak	Ret Time	Type	Width	Area	Height	Area
#	[min]		[min]	mAU*s	[mAU]	%
1	15.361	BB	0.2824	635.1	35.2	8.556
2	17.087	BB	0.3235	6787.6	327.9	91.444
Totals				7422.7	363.1	

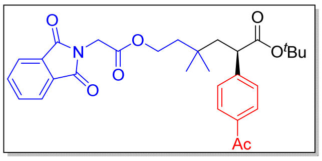

(*R*)-*tert*-butyl 2-(4-acetylphenyl)-6-(2-(1,3-dioxoisoindolin-2-yl)acetoxy)-4,4-dimethylhexanoate (5i)

The reaction was performed following the General Procedure 1 with $NiBr_2$ (2.2 mg, 0.01 mmol) and **L6** (5.4 mg, 0.011 mmol), *tert*-butyl acrylate (15.0 μL, 0.1 mmol, 1.0 equiv.), 1-(4-bromophenyl)ethan-1-one (39.8 mg, 0.2 mmol, 2.0 equiv.), 3-bromo-3-methylbutyl 2-(1,3-dioxoisoindolin-2-yl)acetate (148.1 mg, 0.4 mmol, 4.0 equiv.), Cy_2NMe (58.5 mg, 64 μL, 0.3 mmol), HEH (75.9 mg, 0.3 mmol) and 4CzIPN (8.0 mg, 0.01 mmol) in DMA(3.0 mL). The crude product was purified by flash chromatography on silica gel (eluted with petroleum ether:EtOAc = 5:1) to give the product (41.7 mg, 80% yield, 90% ee) as a white solid. Melting point: 103.8 – 108.4 °C; R_f = 0.52 (petroleum ether:EtOAc = 2:1). $[\alpha]_D^{25}$ = –11.53 (*c* = 0.587, $CHCl_3$). ^{1}H NMR (400 MHz, $CDCl_3$) δ: 7.94 – 7.86 (m, 4H), 7.76 (dd, *J* = 5.5, 3.0 Hz, 2H), 7.41 – 7.37 (m, 2H), 4.39 (s, 2H), 4.20 (t, *J* = 7.5 Hz, 2H), 3.59 (dd, *J* = 9.0, 3.4 Hz, 1H), 2.59 (s, 3H), 2.32 (dd, *J* = 14.2, 9.1 Hz, 1H), 1.62 (t, *J* = 7.5 Hz, 2H), 1.52 (dd, *J* = 14.2, 3.5 Hz, 1H), 1.36 (s, 9H), 0.91 (s, 6H). $^{13}C\{^1H\}$ NMR (101 MHz, $CDCl_3$) δ: 197.92, 172.92, 167.56, 167.37, 146.68, 135.93, 134.37, 132.06, 128.84, 127.99, 123.73, 81.19, 63.12, 48.79, 45.31, 39.76, 38.99, 33.05, 27.85, 27.13, 27.11, 26.74. The ee was determined by HPLC with a Daicel Chiralcel IA column (^{i}PrOH/hexanes = 25/75, 1.0 mL/min, 254 nm, major t_r = 12.014 min (*S*), minor t_r = 14.769 min (*R*)). FTIR (neat, cm^{-1}) ν: 2961, 2926, 2871, 1777, 1724, 1683, 1417, 1143, 1017, 956, 842, 735, 714. HRMS: calcd for $C_{30}H_{36}NO_7$ $[M+H]^+$ 522.2492, found 522.2492.

5i. Racemic product

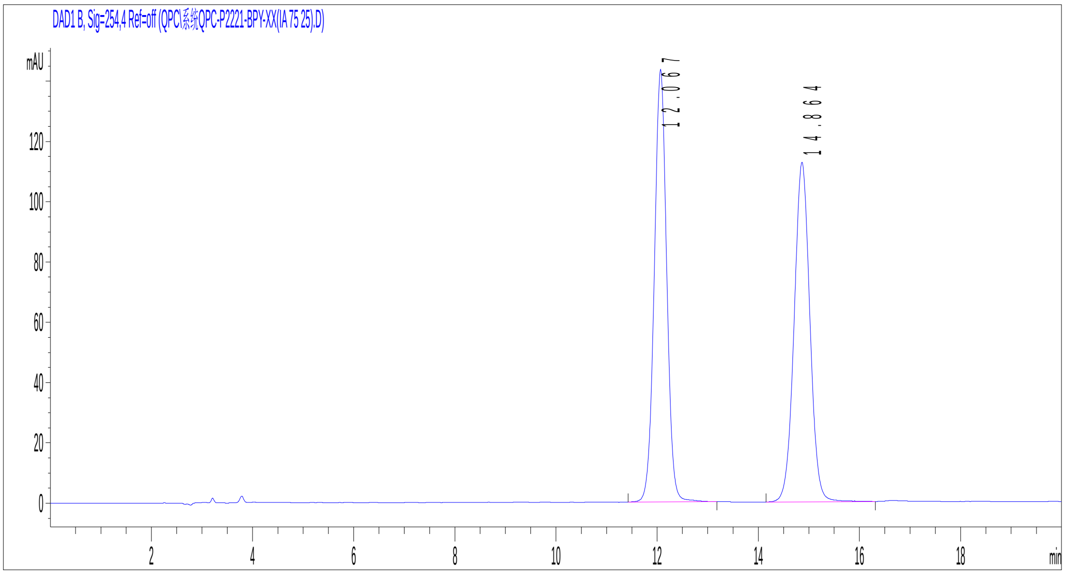

Peak	Ret Time	Type	Width	Area	Height	Area
#	[min]		[min]	mAU*s	[mAU]	%
1	12.067	BB	0.2629	2445	143.4	50.061
2	14.864	BB	0.3326	2439	112.7	49.939
Totals				4884	256.1	

5i. Enantioenriched product, 90% ee

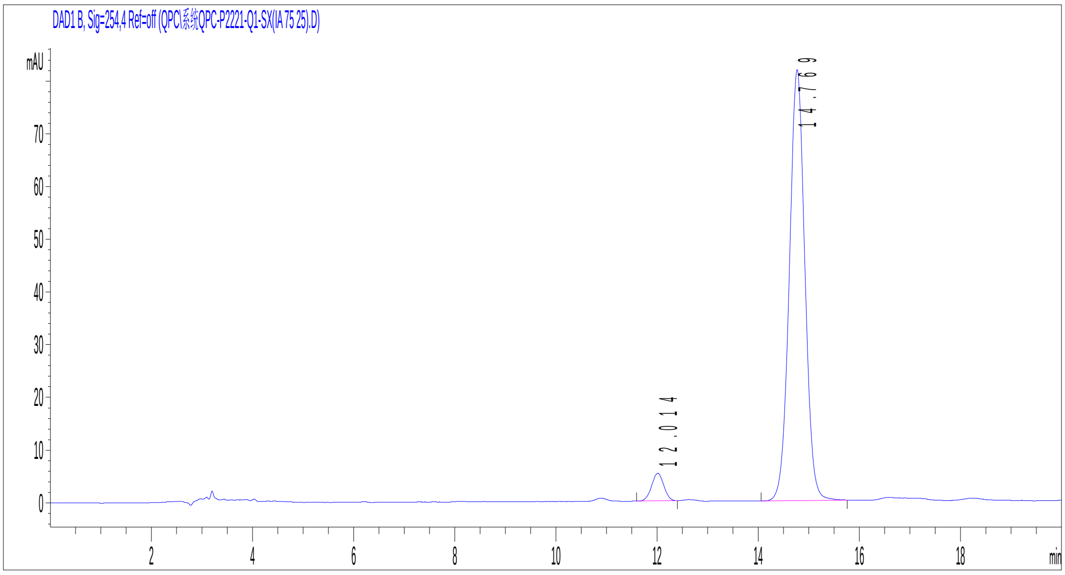

Peak	Ret Time	Type	Width	Area	Height	Area
#	[min]		[min]	mAU*s	[mAU]	%
1	12.014	BB	0.2569	87.7	5.2	4.781
2	14.769	BB	0.3293	1746.1	81.7	95.219
Totals				1833.8	86.9	

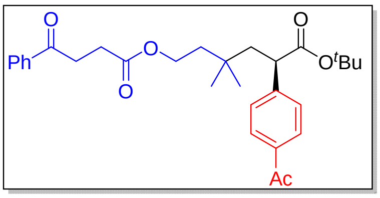

(*R*)-*tert*-butyl 2-(4-acetylphenyl)-4,4-dimethyl-6-((4-oxo-4-phenylbutanoyl)oxy)hexanoate (5j)

The reaction was performed following the General Procedure 1 with $NiBr_2$ (2.2 mg, 0.01 mmol) and **L6** (5.4 mg, 0.011 mmol), *tert*-butyl acrylate (15.0 μL, 0.1 mmol, 1.0 equiv.), 1-(4-bromophenyl)ethan-1-one (39.8 mg, 0.2 mmol, 2.0 equiv.), 3-bromo-3-methylbutyl 4-oxo-4-phenylbutanoate (137.3 mg, 0.4 mmol, 4.0 equiv.), Cy_2NMe (58.5 mg, 64 μL, 0.3 mmol), HEH (75.9 mg, 0.3 mmol) and 4CzIPN (8.0 mg, 0.01 mmol) in DMA (3.0 mL). The crude product was purified by flash chromatography on silica gel (eluted with petroleum ether:EtOAc = 5:1) to give the product (41.5 mg, 84% yield, 72% ee) as a white solid. Melting point: 89.8 – 90.2 °C; R_f = 0.72 (petroleum ether:EtOAc = 2:1). $[\alpha]_D^{25}$ = –16.96 (*c* = 0.91, $CHCl_3$). ^{1}H NMR (400 MHz, $CDCl_3$) δ: 7.98 (d, *J* = 7.0 Hz, 2H), 7.90 (d, *J* = 8.4 Hz, 2H), 7.58 (m, 1H), 7.47 (m, 2H), 7.40 (d, *J* = 8.3 Hz, 2H), 4.14 (t, *J* = 7.5 Hz, 2H), 3.61 (dd, *J* = 9.1, 3.4 Hz, 1H), 3.30 (t, *J* = 6.6 Hz, 2H), 2.71 (t, *J* = 6.7 Hz, 2H), 2.58 (s, 3H), 2.33 (dd, *J* = 14.2, 9.1 Hz, 1H), 1.62 (t, *J* = 7.5 Hz, 2H), 1.54 (dd, *J* = 14.2, 3.5 Hz, 1H), 1.36 (s, 9H), 0.93 (s, 6H). $^{13}C\{^1H\}$ NMR (101 MHz, $CDCl_3$) δ: 198.17, 197.91, 173.04, 173.00, 146.80, 136.58, 135.90, 133.36, 128.81, 128.73, 128.13, 128.01, 81.14, 61.89, 48.84, 45.34, 39.91, 33.43, 33.07, 28.35, 27.86, 27.20, 26.73. The ee was determined by HPLC with a Daicel Chiralcel IA column (^{i}PrOH/hexanes = 15/85, 1.0 mL/min, 250 nm, major t_r = 13.127 min (*S*), minor t_r = 14.076 min (*R*)). FTIR (neat, cm^{-1}) ν: 3059, 2962, 2927, 2871, 1729, 1685, 1367, 1142, 1017, 842, 802, 596. HRMS: calcd for $C_{30}H_{39}O_6$ $[M+H]^+$ 495.2747, found 495.2751.

5j. Racemic product

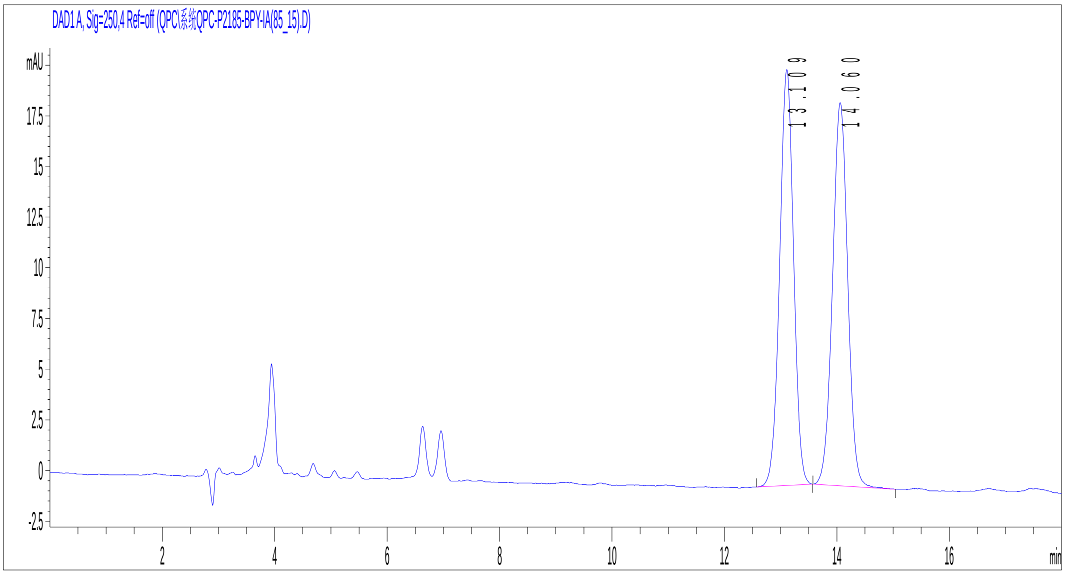

Peak	Ret Time	Type	Width	Area	Height	Area
#	[min]		[min]	mAU*s	[mAU]	%
1	13.109	BB	0.2669	353.3	20.5	49.918
2	14.06	BB	0.2886	354.4	18.9	50.082
Totals				707.7	39.4	

5j. Enantioenriched product, 72% ee

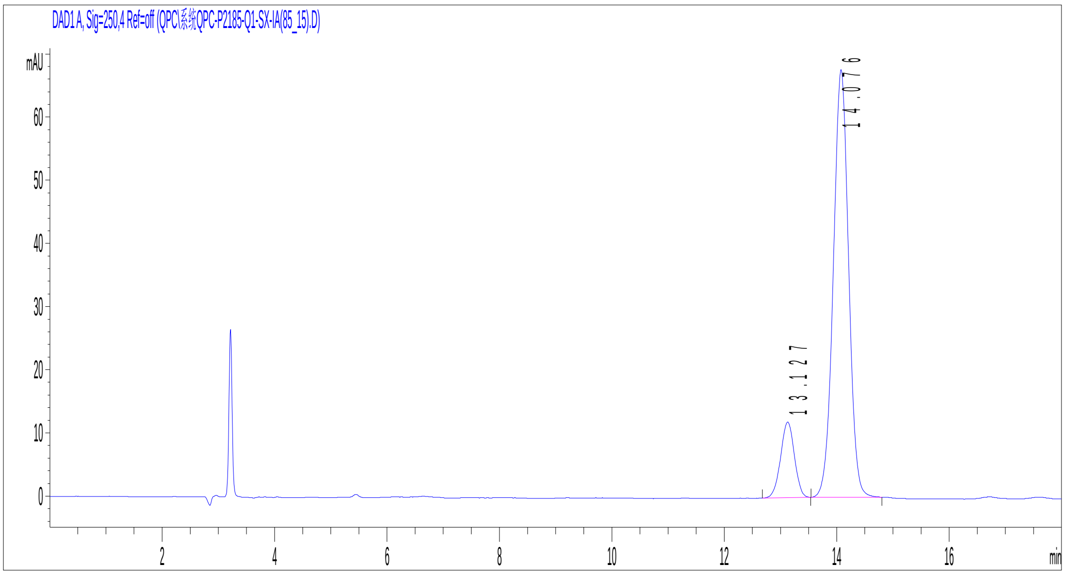

Peak	Ret Time	Type	Width	Area	Height	Area
#	[min]		[min]	mAU*s	[mAU]	%
1	13.127	BB	0.2635	204.8	12	13.814
2	14.076	BB	0.2903	1277.9	67.7	86.186
Totals				1482.7	79.7	

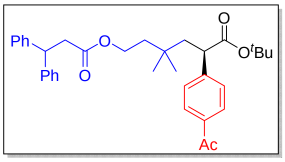

(*R*)-*tert*-butyl 2-(4-acetylphenyl)-6-((3,3-diphenylpropanoyl)oxy)-4,4-dimethylhexanoate (5k)

The reaction was performed following the General Procedure 1 with $NiBr_2$ (2.2 mg, 0.01 mmol) and **L6** (5.4 mg, 0.011 mmol), *tert*-butyl acrylate (15.0 μL, 0.1 mmol, 1.0 equiv.), 1-(4-bromophenyl)ethan-1-one (39.8 mg, 0.2 mmol, 2.0 equiv.), 3-bromo-3-methylbutyl 3,3-diphenylpropanoate (150.1 mg, 0.4 mmol, 4.0 equiv.), Cy_2NMe (58.5 mg, 64 μL, 0.3 mmol), HEH (75.9 mg, 0.3 mmol) and 4CzIPN (8.0 mg, 0.01 mmol) in DMA (3.0 mL). The crude product was purified by flash chromatography on silica gel (eluted with petroleum ether:EtOAc = 10:1) to give the product (45.0 mg, 83% yield, 82% ee) as a colorless oil. R_f = 0.48 (petroleum ether:EtOAc = 5:1). $[\alpha]_D^{25}$ = –18.80 (*c* = 0.718, $CHCl_3$). ^{1}H NMR (400 MHz, $CDCl_3$) δ: 7.89 (d, *J* = 8.5 Hz, 2H), 7.38 (d, *J* = 8.4 Hz, 2H), 7.30 – 7.24 (m, 4H), 7.23 – 7.15 (m, 6H), 4.51 (t, *J* = 8.1 Hz, 1H), 4.00 (t, *J* = 7.4 Hz, 2H), 3.55 (dd, *J* = 9.1, 3.4 Hz, 1H), 2.98 (d, *J* = 8.0 Hz, 2H), 2.58 (s, 3H), 2.27 (dd, *J* = 14.2, 9.2 Hz, 1H), 1.50 – 1.41 (m, 3H), 1.36 (s, 9H), 0.85 (d, *J* = 2.0 Hz, 6H). ^{13}C{^{1}H} NMR (101 MHz, $CDCl_3$) δ: 197.88, 173.00, 171.96, 146.81, 143.51, 143.49, 135.93, 128.82, 128.67, 128.00, 127.77, 126.67, 81.13, 61.69, 48.82, 47.12, 45.25, 40.90, 39.79, 32.99, 27.89, 27.14, 26.74. The ee was determined by HPLC with a Daicel Chiralcel IA column (^{i}PrOH/hexanes = 25/75, 1.0 mL/min, 254 nm, major t_r = 7.937 min (*S*), minor t_r = 8.396 min (*R*)). FTIR (neat, cm^{-1}) ν: 3028, 2961, 2924, 2870, 1729, 1685, 1367, 1266, 1143, 842, 799, 748, 701. HRMS: calcd for $C_{35}H_{42}NaO_5$ $[M+Na]^+$ 565.2930, found 565.2933.

5k. Racemic product

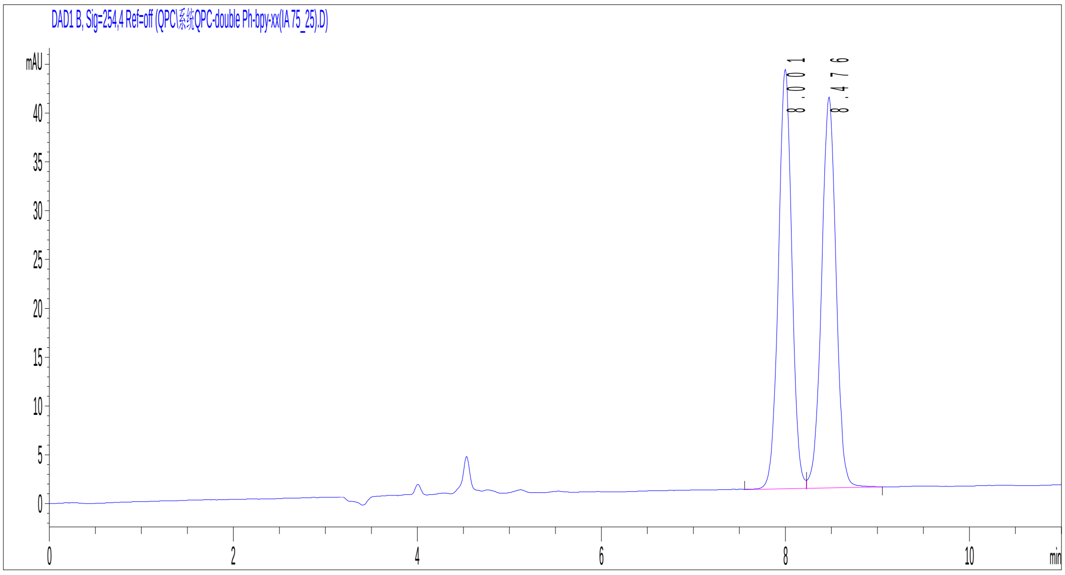

Peak	Ret Time	Type	Width	Area	Height	Area
#	[min]		[min]	mAU*s	[mAU]	%
1	8.001	BV	0.1563	435.3	42.9	49.807
2	8.476	VB	0.1699	438.7	40	50.193
Totals				874	82.9	

5k. Enantioenriched product, 82% ee

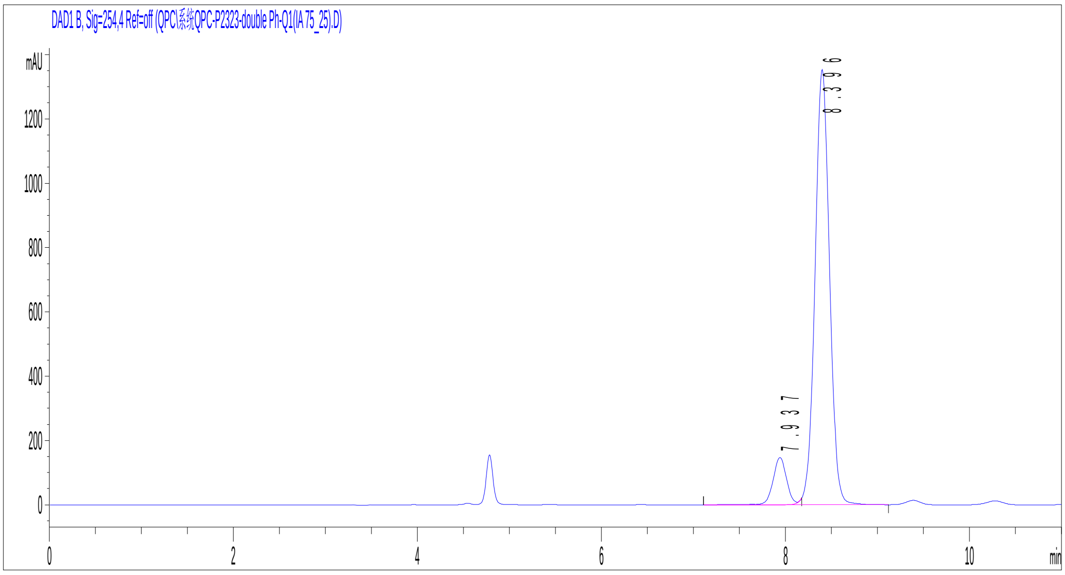

Peak	Ret Time	Type	Width	Area	Height	Area
#	[min]		[min]	mAU*s	[mAU]	%
1	7.937	BV E	0.1576	1505.8	146.9	9.231
2	8.396	VB R	0.1696	14807.3	1353.4	90.769
Totals				16313.1	1500.3	

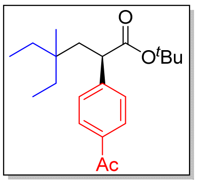

(*R*)-*tert*-butyl 2-(4-acetylphenyl)-4-ethyl-4-methylhexanoate (5l)

The reaction was performed following the General Procedure 1 with $NiBr_2$ (2.2 mg, 0.01 mmol) and **L6** (5.4 mg, 0.011 mmol), *tert*-butyl acrylate (15.0 μL, 0.1 mmol, 1.0 equiv.), 1-(4-bromophenyl)ethan-1-one (39.8 mg, 0.2 mmol, 2.0 equiv.), 3-bromo-3-methylpentane (72.4 mg, 0.4 mmol, 4.0 equiv.), Cy_2NMe (58.5 mg, 64 μL, 0.3 mmol), HEH (75.9 mg, 0.3 mmol) and 4CzIPN (8.0 mg, 0.01 mmol) in DMA (3.0 mL). The crude product was purified by flash chromatography on silica gel (eluted with petroleum ether:EtOAc = 50:1) to give the product (14.0 mg, 42% yield, 88% ee) as a white solid. Melting point: 71.8 – 76.6 ^{o}C; R_f = 0.56 (petroleum ether:EtOAc = 20:1). $[\alpha]_D^{25}$ = –2.75 (*c* = 0.41, $CHCl_3$). ^{1}H NMR (400 MHz, $CDCl_3$) δ: 7.90 (d, *J* = 8.2 Hz, 2H), 7.41 (d, *J* = 8.4 Hz, 2H), 3.55 (dd, *J* = 8.8, 3.5 Hz, 1H), 2.60 (s, 3H), 2.24 (dd, *J* = 14.3, 8.8 Hz, 1H), 1.48 (dd, *J* = 14.4, 3.3 Hz, 1H), 1.37 (s, 9H), 1.27 – 1.21 (m, 4H), 0.76 (m, 9H). ^{13}C{^{1}H} NMR (101 MHz, $CDCl_3$) δ: 197.99, 173.33, 147.42, 135.78, 128.73, 128.09, 80.91, 48.61, 42.10, 36.08, 31.05, 30.91, 27.89, 26.74, 23.95, 8.04. The ee was determined by HPLC with a Daicel Chiralcel IA column (^{i}PrOH/hexanes = 2/98, 1.0 mL/min, 254 nm, major t_r =4.748 min (*S*), minor t_r = 4.968 min (*R*)). FTIR (neat, cm^{-1}) ν: 2963, 2924, 2878, 2856, 1729, 1686, 1606, 1366, 1142, 1018, 956, 800, 751, 596. HRMS: calcd for $C_{21}H_{32}NaO_3$ $[M+Na]^+$ 355.2249, found 355.2253.

5l. Racemic product

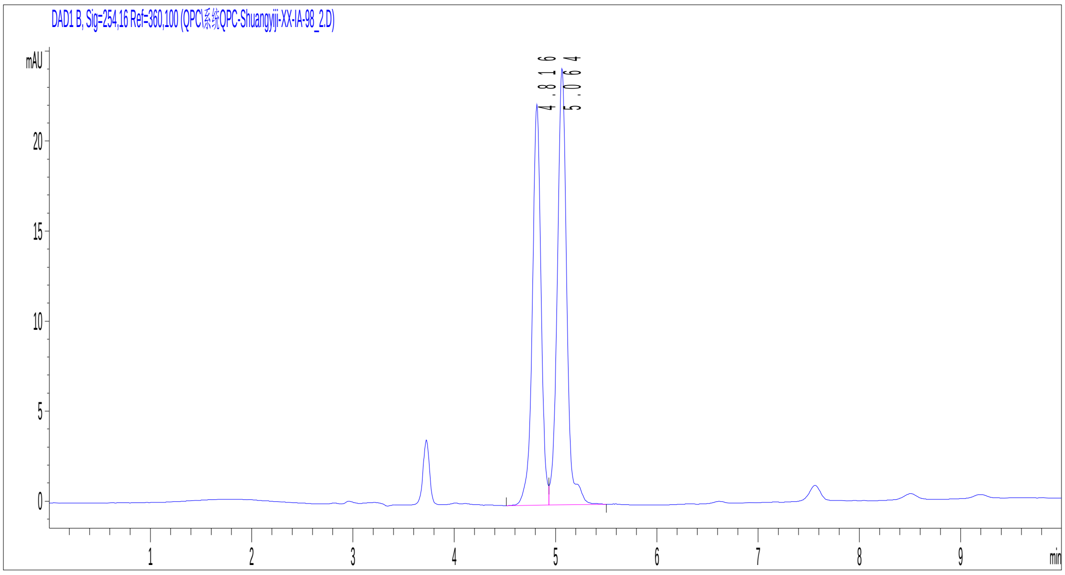

Peak	Ret Time	Type	Width	Area	Height	Area
#	[min]		[min]	mAU*s	[mAU]	%
1	4.816	BV	0.0919	133.4	22.3	45.902
2	5.064	VB	0.0996	157.2	24.3	54.098
Totals				290.6	46.6	

5l. Enantioenriched product, 88% ee

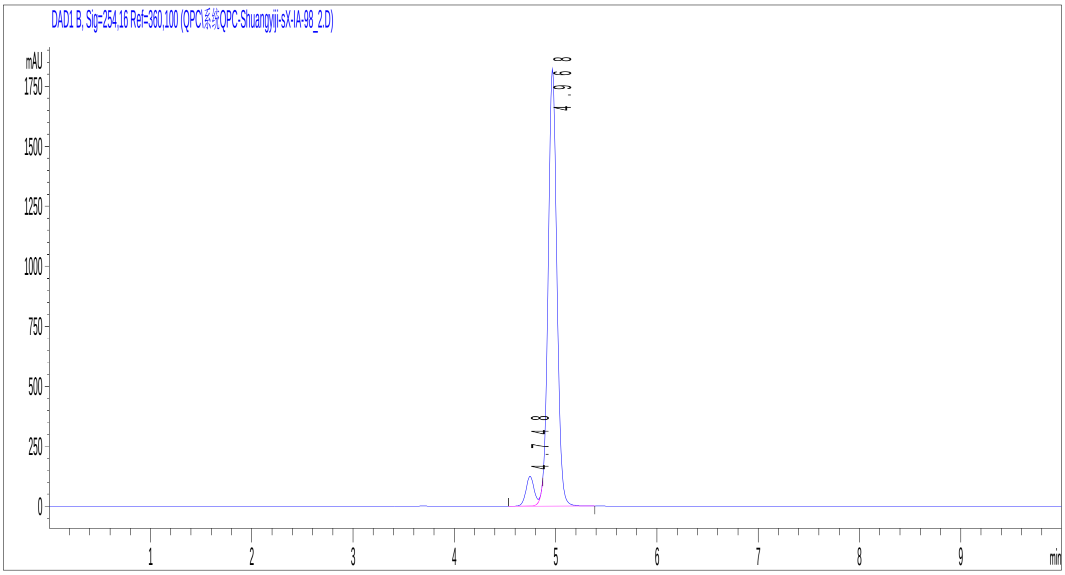

Peak	Ret Time	Type	Width	Area	Height	Area
#	[min]		[min]	mAU*s	[mAU]	%
1	4.748	BV E	0.087	697	125.1	5.961
2	4.968	VB R	0.0924	10997.1	1822.6	94.039
Totals				11694.1	1947.7	

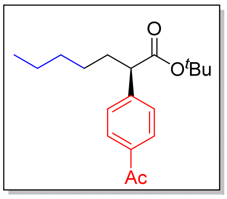

(*R*)-*tert*-butyl 2-(4-acetylphenyl)octanoate (6a)

The reaction was performed following the General Procedure 2 with $NiCl_2$·glyme (2.2 mg, 0.01 mmol) and **L6** (5.4 mg, 0.011 mmol), *tert*-butyl acrylate (15.0 μL, 0.1 mmol, 1.0 equiv.), 1-(4-iodophenyl)ethan-1-one (49.2 mg, 0.2 mmol, 2.0 equiv.), 1-iodobutane (73.6 mg, 0.4 mmol, 4.0 equiv.), Cy_2NMe (58.5 mg, 64.0 μL, 0.3 mmol), HEH (75.9 mg, 0.3 mmol) and 4CzIPN (8.0 mg, 0.01 mmol) in acetone/DMA (2.0/1.0, v/v, 3.0 mL). The crude product was purified by flash chromatography on silica gel (eluted with petroleum ether:EtOAc = 30:1) to give the product (4.6 mg, 15% yield, 76% ee) as a colorless oil. R_f = 0.33 (petroleum ether:EtOAc = 20:1). $[\alpha]_D^{25}$ = –10.50 (*c* = 0.200, $CHCl_3$). ^1H NMR (400 MHz, $CDCl_3$) δ: 7.89 (d, *J* = 8.5 Hz, 2H), 7.38 (d, *J* = 8.5 Hz, 2H), 3.47 (t, *J* = 7.7 Hz, 1H), 2.58 (s, 3H), 2.09 – 1.94 (m, 1H), 1.45 – 1.41 (m, 1H), 1.38 (s, 9H), 1.29 – 1.22 (m, 6H), 0.85 (m, 3H). $^{13}C\{^1H\}$ NMR (101 MHz, $CDCl_3$) δ: Unknown NMR (101 MHz, Chloroform-*d*) δ: 198.0, 172.78, 145.51, 135.97, 128.66, 128.23, 81.02, 52.89, 33.51, 31.62, 28.02, 27.28, 26.71, 22.52, 14.08. The ee was determined by HPLC with a Daicel Chiralcel IA column (^{i}PrOH/hexanes = 1/99, 1.0 mL/min, 250 nm, major t_r = 16.905 min (*S*),minor t_r = 21.29 min (*R*)). FTIR (neat, cm^{-1}) ν: 2960, 2927, 2061, 1727, 1636, 1459, 1367, 1263, 1147, 1018, 797, 597. HRMS: calcd for $C_{19}H_{29}O_3$ $[M+H]^+$ 305.2117, found 305.2120.

6a. Racemic product

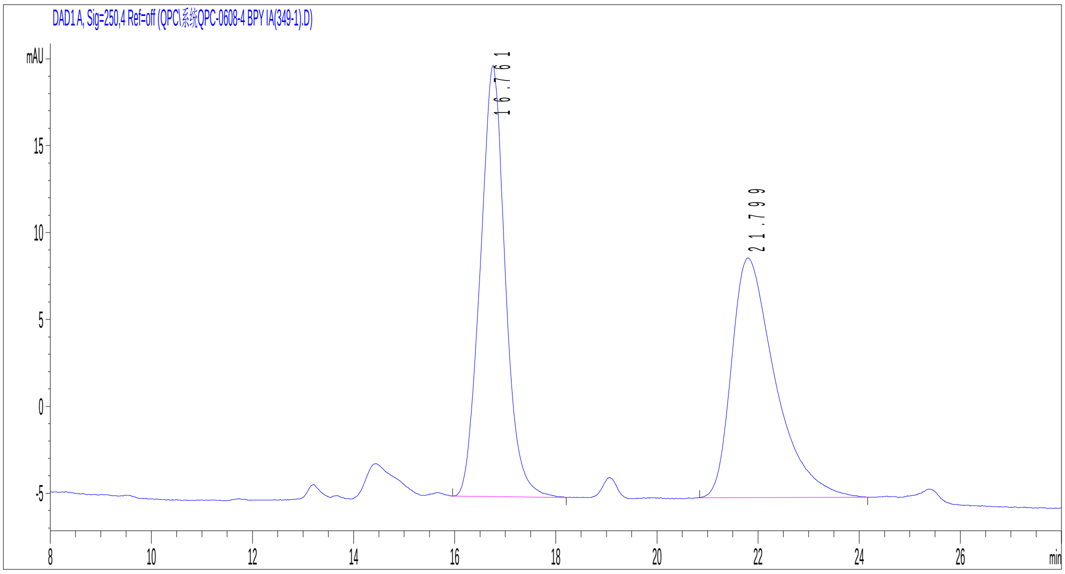

Peak	Ret Time	Type	Width	Area	Height	Area
#	[min]		[min]	mAU*s	[mAU]	%
1	16.761	BB	0.5078	854.7	24.8	50.908
2	21.799	BB	0.8348	824.2	13.8	49.092
Totals				1678.9	38.6	

6a. Enantioenriched product, 76% ee

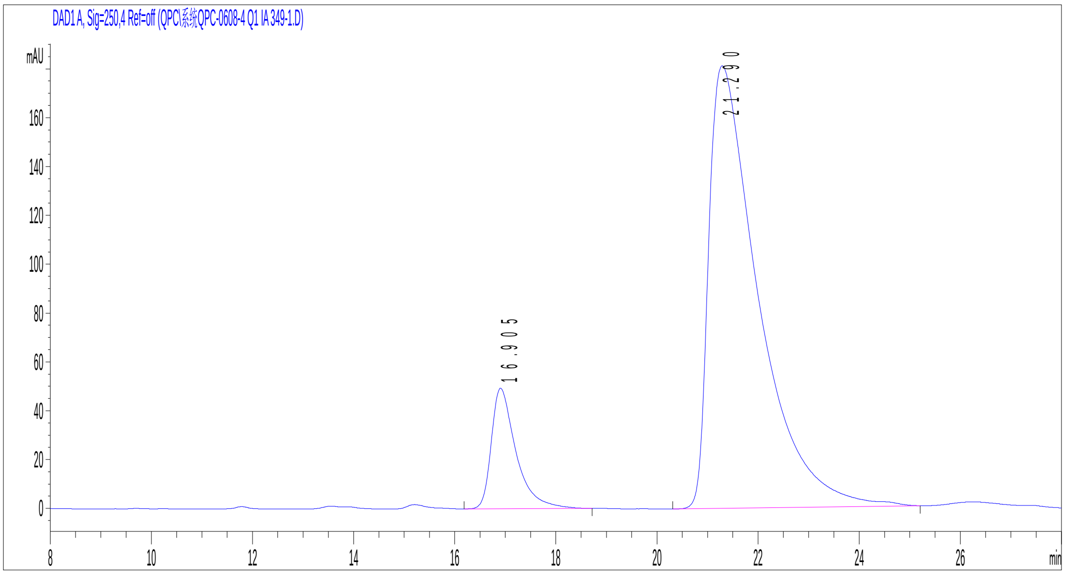

Peak	Ret Time	Type	Width	Area	Height	Area
#	[min]		[min]	mAU*s	[mAU]	%
1	16.905	BB	0.5041	1677.1	49.4	11.985
2	21.29	BB	0.9766	12316.9	181.3	88.015
Totals				13994	230.7	

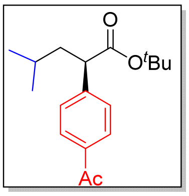

(*R*)-*tert*-butyl 2-(4-acetylphenyl)-4-methylpentanoate (6b)

The reaction was performed following the General Procedure 2 with $NiCl_2$·glyme (2.2 mg, 0.01 mmol) and **L6** (5.4 mg, 0.011 mmol), *tert*-butyl acrylate (15.0 μL, 0.1 mmol, 1.0 equiv.), 1-(4-iodophenyl)ethan-1-one (49.2 mg, 0.2 mmol, 2.0 equiv.), 2-iodopropane (68.0 mg, 39.9 μL, 0.4 mmol, 4.0 equiv.), Cy_2NMe (58.5 mg, 64.0 μL, 0.3 mmol), HEH (75.9 mg, 0.3 mmol) and 4CzIPN (8.0 mg, 0.01 mmol) in acetone/DMA (2.0/1.0, v/v, 3.0 mL). The crude product was purified by flash chromatography on silica gel (eluted with petroleum ether:EtOAc = 30:1) to give the product (21.8 mg, 75% yield, 92% ee) as a colorless oil. R_f = 0.39 (petroleum ether:EtOAc = 20:1). $[\alpha]_D^{25}$ = –21.43 (*c* = 0.700, $CHCl_3$). ^{1}H NMR (400 MHz, $CDCl_3$) δ: 7.91 (d, *J* = 8.4 Hz, 2H), 7.41 (d, *J* = 8.3 Hz, 2H), 3.61 (t, *J* = 7.8 Hz, 1H), 2.60 (s, 3H), 1.94 (dt, *J* = 13.6, 7.7 Hz, 1H), 1.68 – 1.57 (m, 1H), 1.45 (m, 1H), 1.39 (s, 9H), 0.91 (d, *J* = 6.6 Hz, 6H). ^{13}C{^{1}H} NMR (101 MHz, $CDCl_3$) δ: 198.02, 172.86, 145.55, 135.94, 128.69, 128.26, 81.04, 50.77, 42.44, 28.00, 26.76, 26.03, 22.69, 22.39. The ee was determined by HPLC with a Daicel Chiralcel IA column (^{i}PrOH/hexanes = 2/98, 1.0 mL/min, 254 nm, major t_r = 4.958 min (*S*), minor t_r = 5.121 min (*R*)). FTIR (neat, cm^{-1}) ν: 2959, 2871, 1728, 1685, 1635, 1607, 1415, 1367, 1267, 1147, 956, 843, 798, 597. HRMS: calcd for $C_{18}H_{27}O_3$ $[M+H]^+$ 291.1960, found 291.1960.

6b. Racemic product

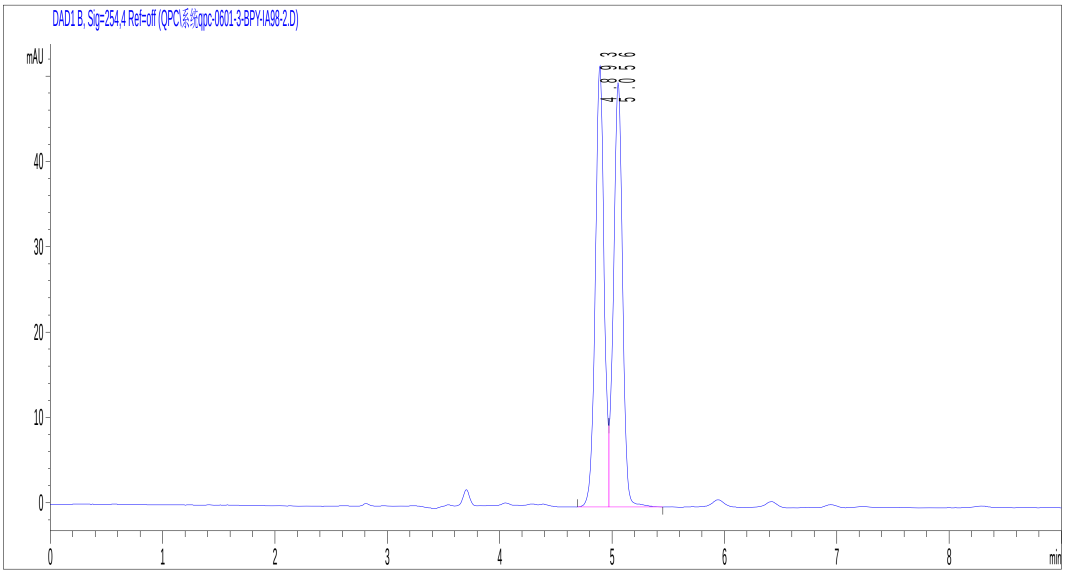

Peak	Ret Time	Type	Width	Area	Height	Area
#	[min]		[min]	mAU*s	[mAU]	%
1	4.893	BV	0.0831	279.9	51.8	50.129
2	5.056	VB	0.0854	278.4	49.7	49.871
Totals				558.3	101.5	

6b. Enantioenriched product, 92% ee

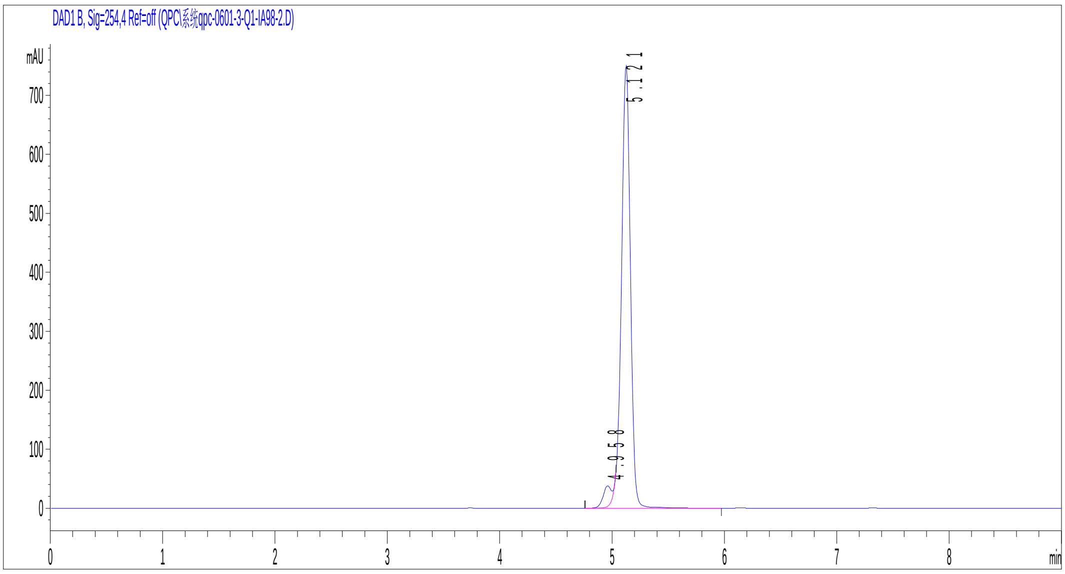

Peak	Ret Time	Type	Width	Area	Height	Area
#	[min]		[min]	mAU*s	[mAU]	%
1	4.958	BV E	0.0773	179.1	35.3	4.096
2	5.121	VB R	0.0851	4193.5	751.1	95.904
Totals				4372.6	786.4	

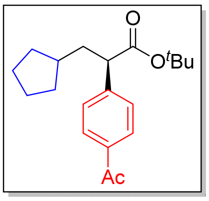

(R)-*tert*-butyl 2-(4-acetylphenyl)-3-cyclopentylpropanoate (6c)

The reaction was performed following the General Procedure 2 with $NiCl_2$·glyme (2.2 mg, 0.01 mmol) and **L6** (5.4 mg, 0.011 mmol), *tert*-butyl acrylate (15.0 μL, 0.1 mmol, 1.0 equiv.), 1-(4-iodophenyl)ethan-1-one (49.2 mg, 0.2 mmol, 2.0 equiv.), iodocyclopentane (78.4 mg, 46.3 μL, 0.4 mmol, 4.0 equiv.), Cy_2NMe (58.5 mg, 64.0 μL, 0.3 mmol), HEH (75.9 mg, 0.3 mmol) and 4CzIPN (8.0 mg, 0.01 mmol) in acetone/DMA (2.0/1.0, v/v, 3.0 mL). The crude product was purified by flash chromatography on silica gel (eluted with petroleum ether:EtOAc = 30:1) to give the product (21.5 mg, 68% yield, 90% ee) as a colorless oil. R_f = 0.38 (petroleum ether:EtOAc = 20:1). $[\alpha]_D^{25}$ = –30.43 (c = 0.447, $CHCl_3$). ^1H NMR (400 MHz, $CDCl_3$) δ: 7.91 (d, J = 8.5 Hz, 2H), 7.41 (d, J = 8.5 Hz, 2H), 3.56 (t, J = 7.8 Hz, 1H), 2.60 (s, 3H), 2.05 (dt, J = 13.4, 7.7 Hz, 1H), 1.82 – 1.70 (m, 3H), 1.61 (m, 3H), 1.50 – 1.42 (m, 2H), 1.39 (s, 9H), 1.11 (m, 2H). $^{13}C\{^1H\}$ NMR (101 MHz, $CDCl_3$) δ: 197.99, 172.88, 145.58, 135.95, 128.66, 128.28, 81.00, 52.08, 39.80, 38.00, 32.81, 32.42, 28.01, 26.73, 25.16. The ee was determined by HPLC with a Daicel Chiralcel IA column (^{i}PrOH/hexanes = 1/99, 1.0 mL/min, 254 nm, major t_r = 8.461 min (S), minor t_r = 10.891 min (R)). FTIR (neat, cm^{-1}) ν: 2952, 2867, 1727, 1685, 1607, 1415, 1367, 1266, 1141, 1018, 956, 798, 597. HRMS: calcd for $C_{20}H_{29}O_3$ $[M+H]^+$ 317.2117, found 317.2118.

6c. Racemic product

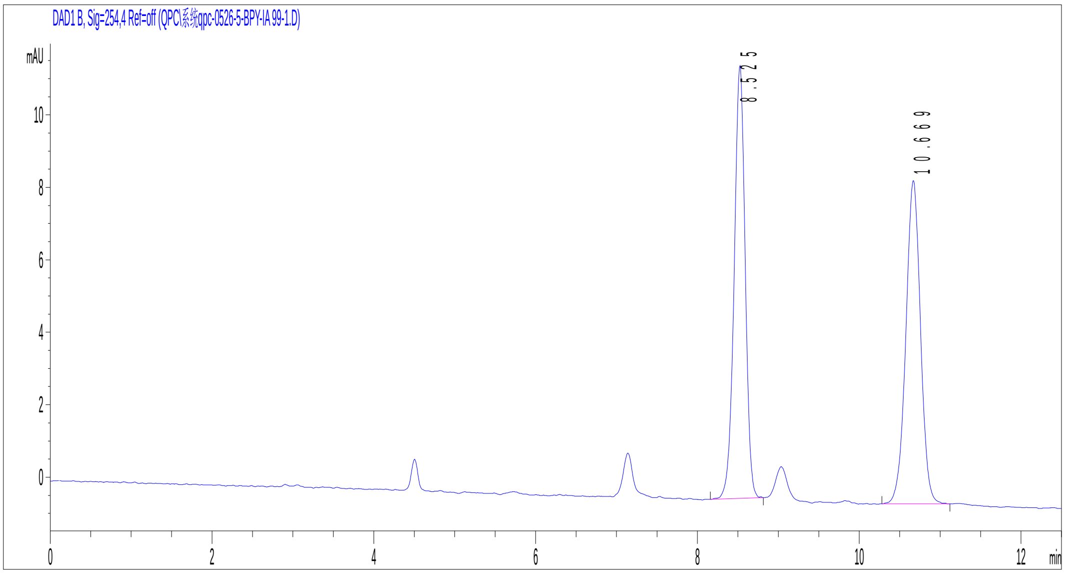

Peak	Ret Time	Type	Width	Area	Height	Area
#	[min]		[min]	mAU*s	[mAU]	%
1	8.525	BB	0.144	112.7	11.9	50.004
2	10.669	BB	0.1932	112.7	8.9	49.996
Totals				225.4	20.8	

6c. Enantioenriched product, 90% ee

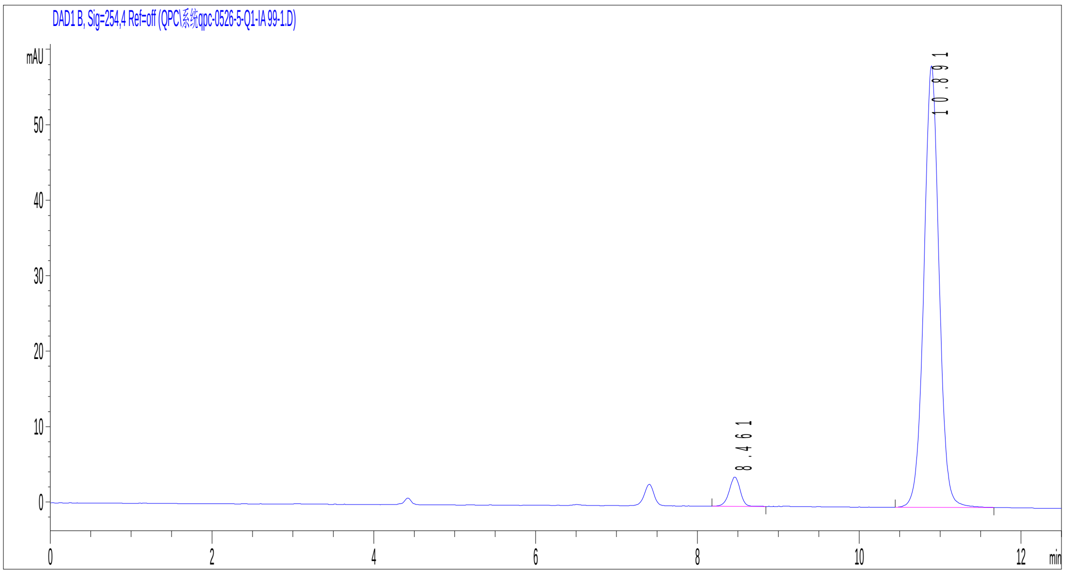

Peak	Ret Time	Type	Width	Area	Height	Area
#	[min]		[min]	mAU*s	[mAU]	%
1	8.461	BB	0.1465	37	3.9	4.617
2	10.891	BB	0.2003	763.5	58.5	95.383
Totals				800.5	62.4	

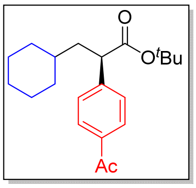

(*R*)-*tert*-butyl 2-(4-acetylphenyl)-3-cyclohexylpropanoate (6d)

The reaction was performed following the General Procedure 2 with $NiCl_2$·glyme (2.2 mg, 0.01 mmol) and **L6** (5.4 mg, 0.011 mmol), *tert*-butyl acrylate (15.0 μL, 0.1 mmol, 1.0 equiv.), 1-(4-iodophenyl)ethan-1-one (49.2 mg, 0.2 mmol, 2.0 equiv.), iodocyclohexane (84.0 mg, 51.7 μL, 0.4 mmol, 4.0 equiv.), Cy_2NMe (58.5 mg, 64.0 μL, 0.3 mmol), HEH (75.9 mg, 0.3 mmol) and 4CzIPN (8.0 mg, 0.01 mmol) in acetone/DMA (2.0/1.0, v/v, 3.0 mL). The crude product was purified by flash chromatography on silica gel (eluted with petroleum ether:EtOAc = 30:1) to give the product (23.1 mg, 70% yield, 90% ee) as a colorless oil. R_f = 0.33 (petroleum ether:EtOAc = 20:1). $[\alpha]_D^{25}$ = –28.63 (*c* = 1.617, $CHCl_3$). ^{1}H NMR (400 MHz, $CDCl_3$) δ: 7.91 (d, *J* = 8.3 Hz, 2H), 7.40 (d, *J* = 8.4 Hz, 2H), 3.65 (t, *J* = 7.8 Hz, 1H), 2.60 (s, 3H), 1.94 (dt, *J* = 14.2, 7.6 Hz, 1H), 1.74 – 1.58 (m, 6H), 1.39 (s, 9H), 1.14 (m, 4H), 0.97 – 0.86 (m, 2H). ^{13}C{^{1}H} NMR (101 MHz, $CDCl_3$) δ: 198.03, 172.96, 145.69, 135.93, 128.68, 128.26, 81.00, 50.02, 41.07, 35.48, 33.34, 33.08, 28.02, 26.75, 26.54, 26.24, 26.20. The ee was determined by HPLC with a Daicel Chiralcel IA column (^{i}PrOH/hexanes = 2/98, 1.0 mL/min, 254 nm, major t_r = 5.914 min (*S*), minor t_r = 6.373 min (*R*)). FTIR (neat, cm^{-1}) ν: 2979, 2920, 2849, 1718, 1681, 1636, 1446, 1365, 1271, 1148, 833, 600. HRMS: calcd for $C_{21}H_{31}O_3$ $[M+H]^+$ 331.2273, found 331.2273.

6d. Racemic product

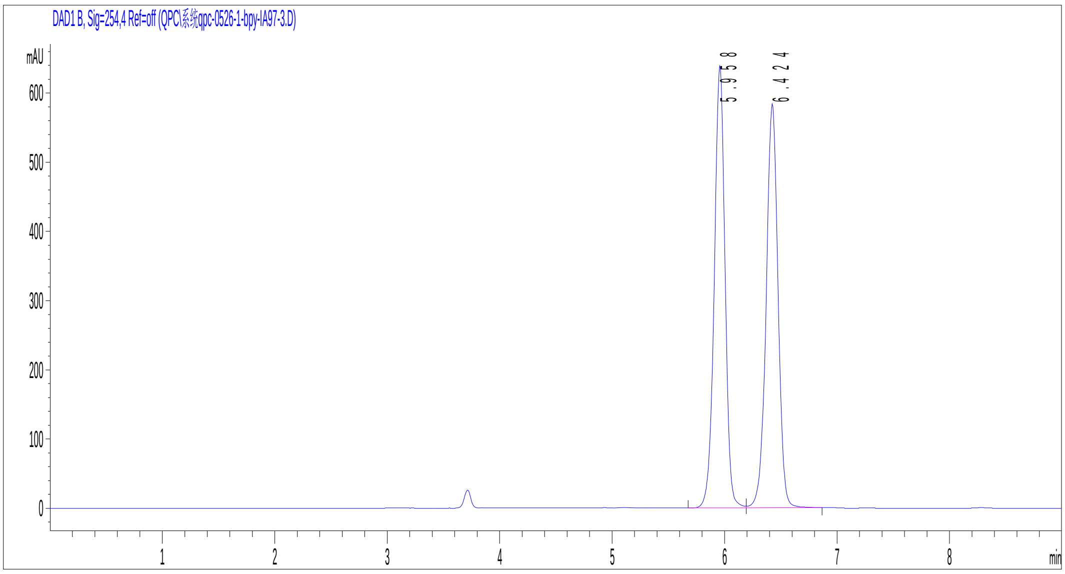

Peak	Ret Time	Type	Width	Area	Height	Area
#	[min]		[min]	mAU*s	[mAU]	%
1	5.958	BV	0.1014	4242.1	639.5	49.830
2	6.424	VB	0.1113	4271	584.1	50.170
Totals				8513.1	1223.6	

6d. Enantioenriched product, 90% ee

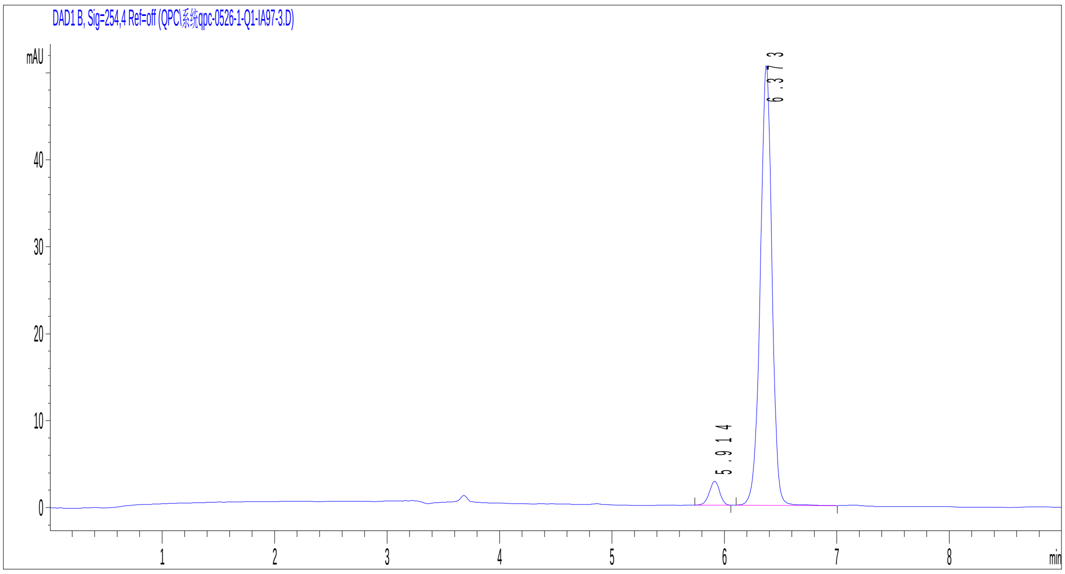

Peak	Ret Time	Type	Width	Area	Height	Area
#	[min]		[min]	mAU*s	[mAU]	%
1	5.914	BB	0.0982	17.6	2.8	4.608
2	6.373	BB	0.1098	363.4	50.6	95.392
Totals				381	53.4	

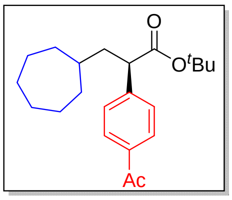

(*R*)-*tert*-butyl 2-(4-acetylphenyl)-3-cycloheptylpropanoate (6e)

The reaction was performed following the General Procedure 2 with $NiCl_2$·glyme (2.2 mg, 0.01 mmol) and **L6** (5.4 mg, 0.011 mmol), *tert*-butyl acrylate (15.0 μL, 0.1 mmol, 1.0 equiv.), 1-(4-iodophenyl)ethan-1-one (49.2 mg, 0.2 mmol, 2.0 equiv.), HEH (75.9 mg, 0.3 mmol) and 4CzIPN (8.0 mg, 0.01 mmol) in acetone/DMA (2.0/1.0, v/v, 3.0 mL). The crude product was purified by flash chromatography on silica gel (eluted with petroleum ether:EtOAc = 30:1) to give the product (14.8 mg, 43% yield, 90% ee) as a colorless oil. R_f = 0.37 (petroleum ether:EtOAc = 20:1). $[\alpha]_D^{25}$ = –23.28 (*c* = 0.570, $CHCl_3$). ^1H NMR (400 MHz, $CDCl_3$) δ: 7.91 (d, *J* = 8.3 Hz, 2H), 7.40 (d, *J* = 8.5 Hz, 2H), 3.61 (t, *J* = 7.8 Hz, 1H), 2.60 (s, 3H), 1.97 (dt, *J* = 15.1, 7.6 Hz, 1H), 1.72 (m, 2H), 1.69 – 1.62 (m, 4H), 1.54 – 1.42 (m, 4H), 1.39 (s, 9H), 1.33 (m, 2H), 1.25 – 1.17 (m, 2H). $^{13}C\{^1H\}$ NMR (101 MHz, $CDCl_3$) δ: 198.06, 172.96, 145.65, 135.93, 128.68, 128.29, 81.02, 50.63, 41.61, 36.84, 34.48, 34.28, 28.59, 28.03, 26.73, 26.31, 26.25. The ee was determined by HPLC with a Daicel Chiralcel IA column (^{i}PrOH/hexanes = 10/90, 1.0 mL/min, 250 nm, major t_r = 4.728 min (*S*), minor t_r = 4.931 min (*R*)). FTIR (neat, cm^{-1}) ν: 2926, 2855, 2070, 1725, 1636, 1560, 1459, 1414, 1366, 1266, 1143, 1018, 785. HRMS: calcd for $C_{22}H_{32}NaO_3$ $[M+Na]^+$ 367.2249, found 367.2246.

6e. Racemic product

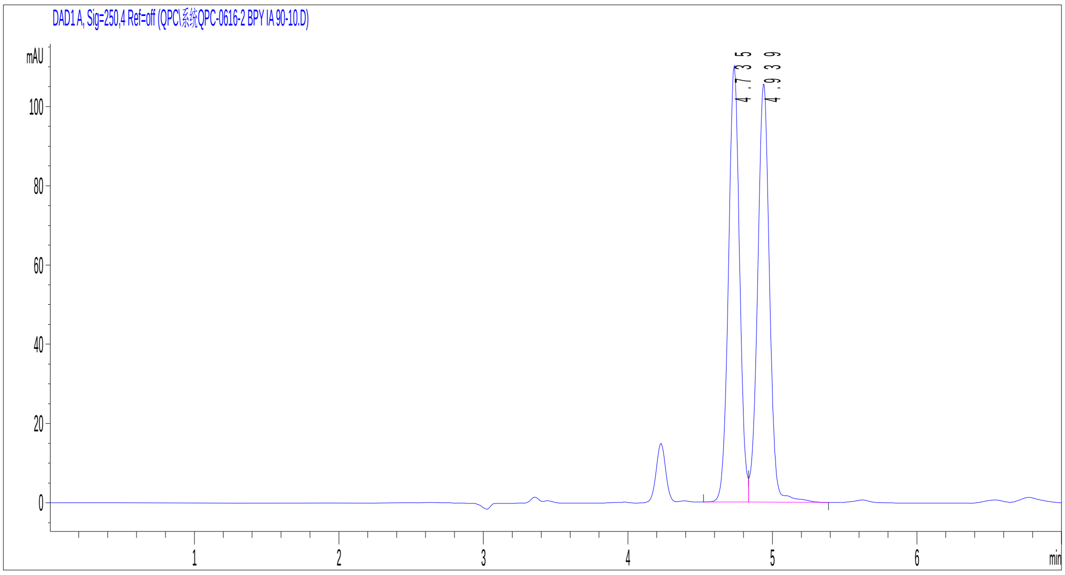

Peak	Ret Time	Type	Width	Area	Height	Area
#	[min]		[min]	mAU*s	[mAU]	%
1	4.735	BV	0.0822	587.5	110.2	49.220
2	4.939	VB	0.0868	606.1	105.9	50.780
Totals				1193.6	216.1	

6e. Enantioenriched product, 90% ee

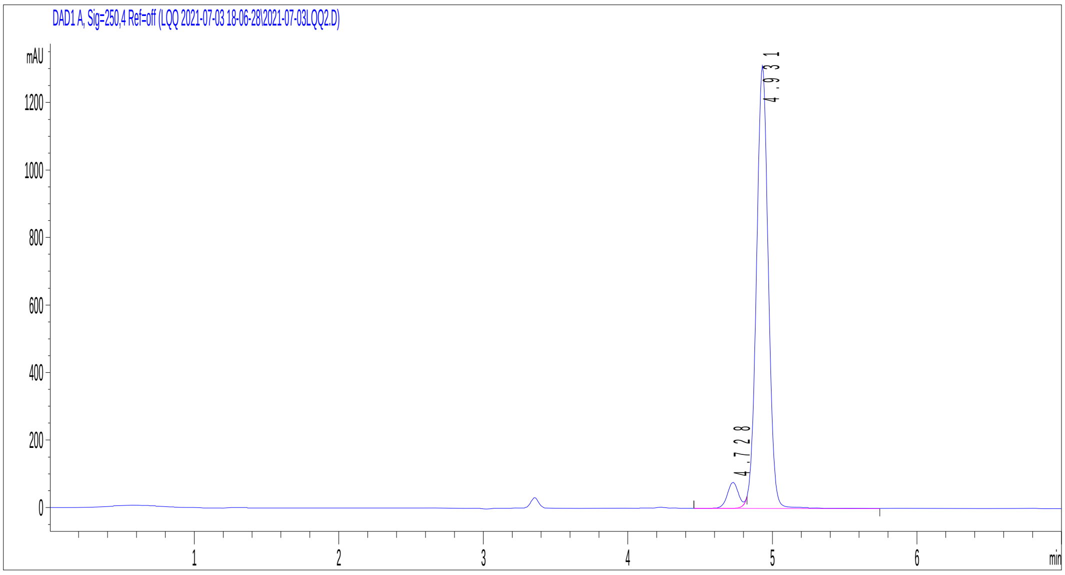

Peak	Ret Time	Type	Width	Area	Height	Area
#	[min]		[min]	mAU*s	[mAU]	%
1	4.728	BV E	0.0817	405.5	76.6	5.145
2	4.931	VB R	0.0885	7474.6	1311.3	94.855
Totals				7880.1	1387.9	

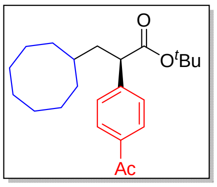

(*R*)-*tert*-butyl2-(4-acetylphenyl)-3-cyclooctylpropanoate (6f)

The reaction was performed following the General Procedure 2 with $NiCl_2$·glyme (2.2 mg, 0.01 mmol) and **L6** (5.4 mg, 0.011 mmol), *tert*-butyl acrylate (15.0 μL, 0.1 mmol, 1.0 equiv.), 1-(4-iodophenyl)ethan-1-one (49.2 mg, 0.2 mmol, 2.0 equiv.), iodocyclooctane (95.2 mg, 0.4 mmol, 4.0 equiv.), Cy_2NMe (58.5 mg, 64.0 μL, 0.3 mmol), HEH (75.9 mg, 0.3 mmol) and 4CzIPN (8.0 mg, 0.01 mmol) in acetone/DMA (2.0/1.0, v/v, 3.0 mL). The crude product was purified by flash chromatography on silica gel (eluted with petroleum ether:EtOAc = 30:1) to give the product (17.9 mg, 50% yield, 90% ee) as a colorless oil. R_f = 0.35 (petroleum ether:EtOAc = 20:1). $[\alpha]_D^{25}$ = –23.22 (*c* = 0.290, $CHCl_3$). ^{1}H NMR (400 MHz, $CDCl_3$) δ: 7.91 (d, *J* = 8.6 Hz, 2H), 7.41 (d, *J* = 8.5 Hz, 2H), 3.62 (t, *J* = 7.8 Hz, 1H), 2.60 (s, 3H), 2.02 – 1.92 (m, 1H), 1.62 (m, 6H), 1.57 – 1.46 (m, 4H), 1.46 – 1.40 (m, 4H), 1.40 (s, 9H), 1.33 – 1.24 (m, 2H). $^{13}C\{^1H\}$ NMR (101 MHz, $CDCl_3$) δ: 198.03, 172.94, 145.68, 135.94, 128.67, 128.27, 81.02, 50.65, 41.70, 34.82, 32.21, 32.13, 28.04, 27.31, 27.23, 26.74, 26.36, 25.46, 25.31. The ee was determined by HPLC with a Daicel Chiralcel IA column (^{i}PrOH/hexanes = 2/98, 1.0 mL/min, 254 nm, major t_r = 7.302 min (*S*), minor t_r = 7.76 min (*R*)). FTIR (neat, cm^{-1}) ν: 2924, 2855, 1728, 1685, 1636, 1367, 1265, 1145, 1017, 956, 796, 598. HRMS: calcd for $C_{23}H_{34}NaO_3$ $[M+Na]^+$ 381.2406, found 381.2402.

6f. Racemic product

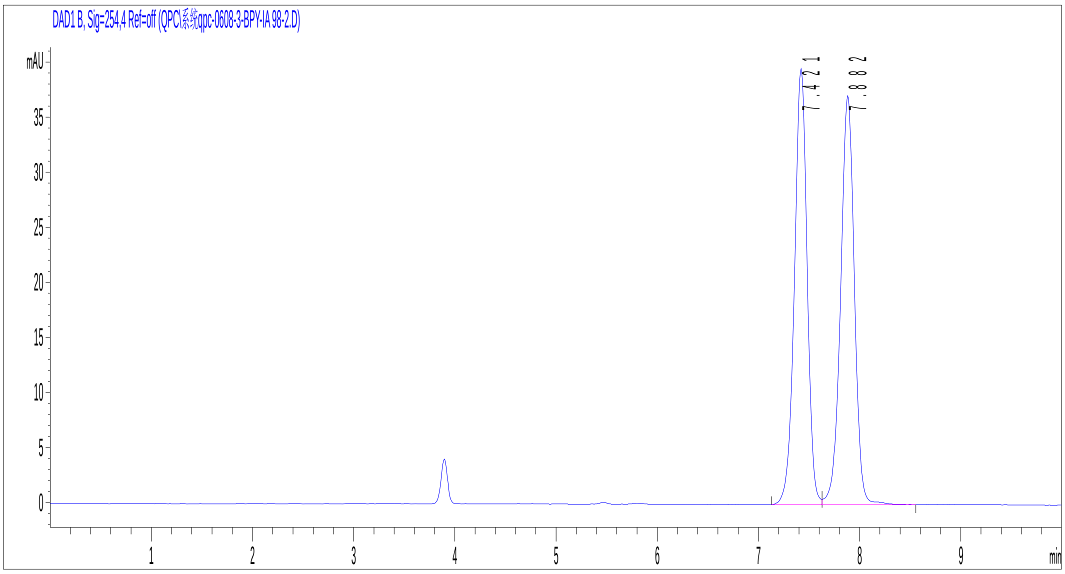

Peak	Ret Time	Type	Width	Area	Height	Area
#	[min]		[min]	mAU*s	[mAU]	%
1	7.421	BV	0.1356	344.7	39.6	49.537
2	7.882	VB	0.1443	351.1	37.1	50.463
Totals				695.8	76.7	

6f. Enantioenriched product, 90% ee

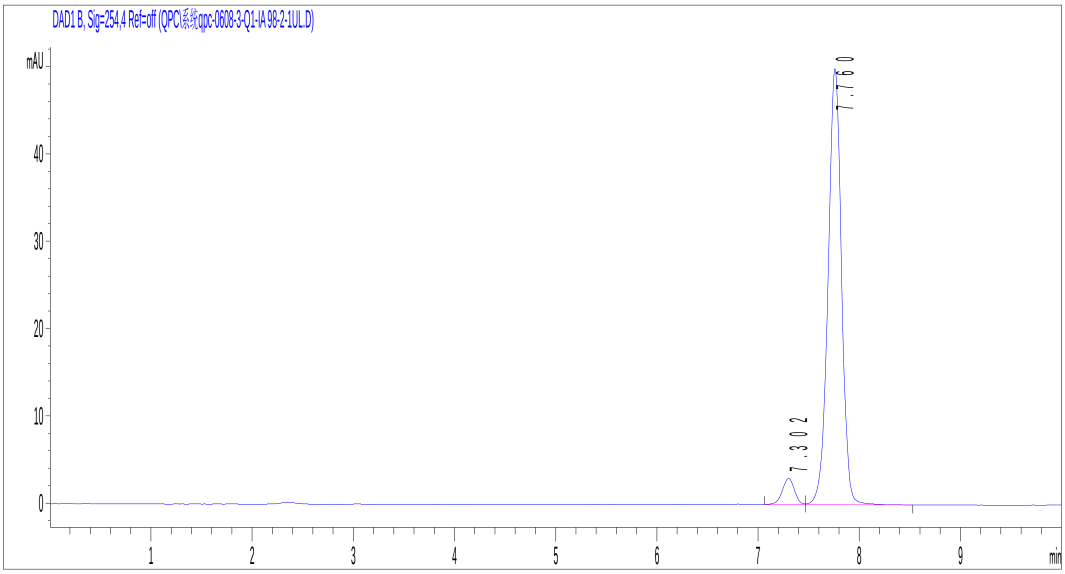

Peak	Ret Time	Type	Width	Area	Height	Area
#	[min]		[min]	mAU*s	[mAU]	%
1	7.302	BV	0.1289	25.1	3	5.164
2	7.76	VB	0.1416	460.4	49.9	94.836
Totals				485.5	52.9	

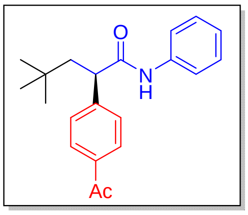

(*R*)-2-(4-acetylphenyl)-4,4-dimethyl-N-phenylpentanamide (6g)

The reaction was performed following the General Procedure 1 with $NiBr_2$ (2.2 mg, 0.01 mmol) and **L6** (5.4 mg, 0.011 mmol), N-phenylacrylamide (14.7 mg, 0.1 mmol, 1.0 equiv.), 1-(4-bromophenyl)ethan-1-one (39.8 mg, 0.2 mmol, 2.0 equiv.), ^{t}BuBr (54.8 mg, 44.9 μL, 0.4 mmol, 4.0 equiv.), Cy_2NMe (58.5 mg, 64.0 μL, 0.3 mmol), HEH (75.9 mg, 0.3 mmol) and 4CzIPN (8.0 mg, 0.01 mmol) in DMA (3.0 mL). The crude product was purified by flash chromatography on silica gel (eluted with petroleum ether:EtOAc = 10:1) to give the product (12.9 mg, 40% yield, 33% ee) as a colorless oil. R_f = 0.44 (petroleum ether:EtOAc = 5:1). $[\alpha]_D^{25}$ = 22.11 (c = 0.603, $CHCl_3$). ^{1}H NMR (400 MHz, $CDCl_3$) δ: 7.91 (d, J = 8.2 Hz, 2H), 7.49 (d, J = 8.1 Hz, 2H), 7.43 (m, 3H), 7.27 (t, J = 7.8 Hz, 2H), 7.07 (t, J = 7.3 Hz, 1H), 3.63 (dd, J = 7.7, 4.6 Hz, 1H), 2.59 (s, 3H), 2.49 (dd, J = 14.1, 7.8 Hz, 1H), 1.63 (dd, J = 14.1, 4.5 Hz, 1H), 0.93 (s, 9H). ^{13}C{^{1}H} NMR (101 MHz, $CDCl_3$) δ:197.97, 171.28, 147.11, 137.90, 136.08, 129.12, 129.07, 128.09, 124.56, 119.95, 51.13, 47.08, 31.24, 29.70, 26.77. The ee was determined by HPLC with a Daicel Chiralcel IA column (^{i}PrOH/hexanes = 20/80, 1.0 mL/min, 254 nm, major t_r = 7.849 min (*S*), minor t_r = 8.314 min (*R*)). FTIR (neat, cm^{-1}) ν: 2961, 2070, 1636, 1546, 1442, 1364, 1308, 1270, 1174, 1017, 753, 598. HRMS: calcd for $C_{21}H_{26}NO_2$ $[M+H]^+$ 324.1964, found 324.1961.

6g. Racemic product

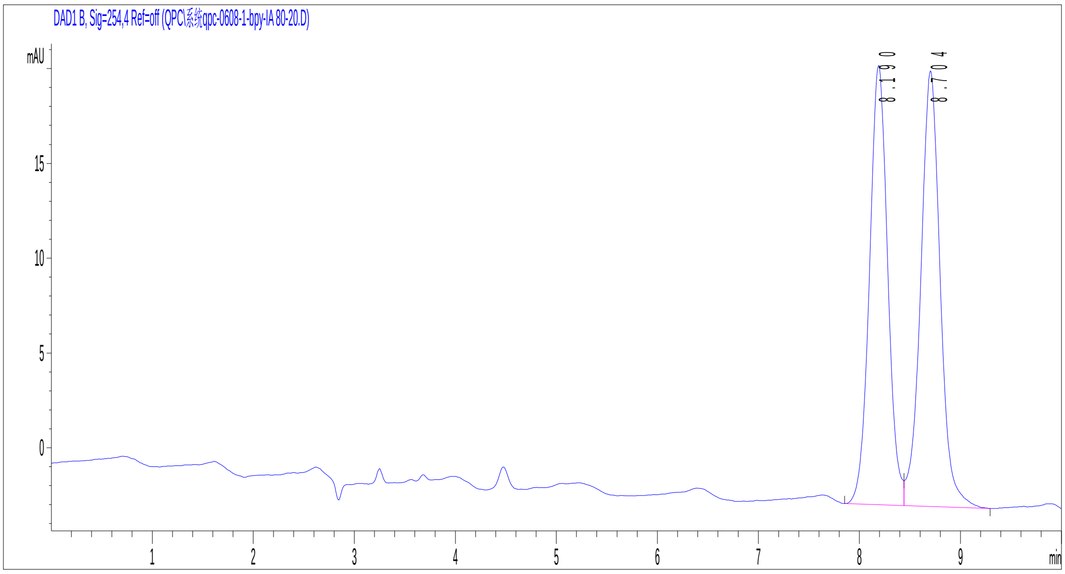

Peak	Ret Time	Type	Width	Area	Height	Area
#	[min]		[min]	mAU*s	[mAU]	%
1	8.19	BV	0.1945	291.1	23.2	48.472
2	8.704	VB	0.2052	309.4	23	51.528
Totals				600.5	46.2	

6g. Enantioenriched product, 33% ee

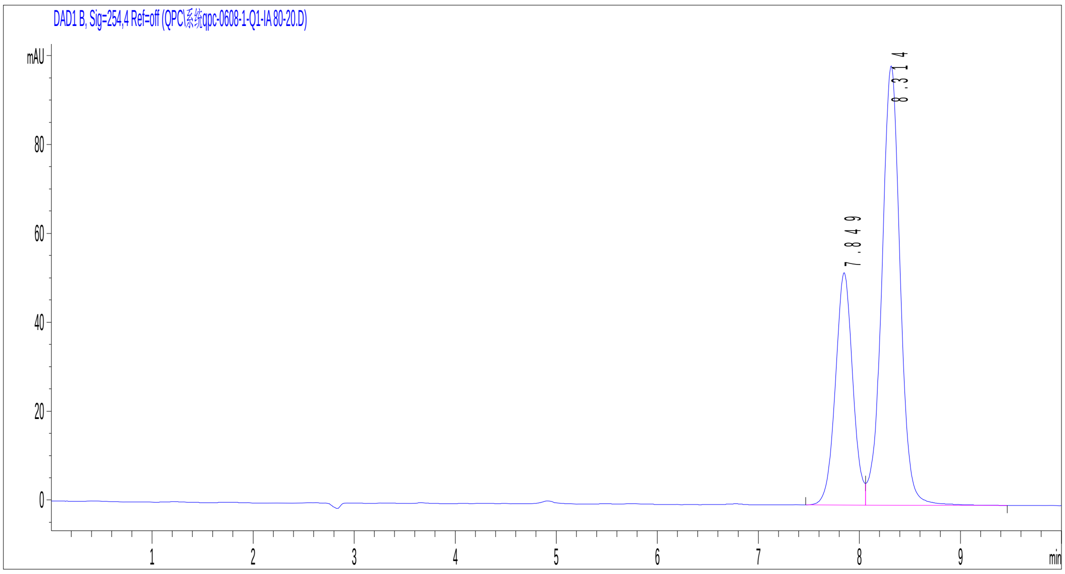

Peak	Ret Time	Type	Width	Area	Height	Area
#	[min]		[min]	mAU*s	[mAU]	%
1	7.849	BV	0.1813	624.1	52.3	33.290
2	8.314	VB	0.1936	1250.6	98.9	66.710
Totals				1874.7	151.2	

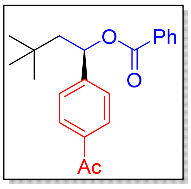

(*R*)-1-(4-acetylphenyl)-3,3-dimethylbutyl benzoate (6h)

The reaction was performed following the General Procedure 1 with $NiBr_2$ (2.2 mg, 0.01 mmol) and **L6** (5.4 mg, 0.011 mmol), vinyl benzoate (14.8 mg, 0.1 mmol, 1.0 equiv.), 1-(4-bromophenyl)ethan-1-one (39.8 mg, 0.2 mmol, 2.0 equiv.), ^{t}BuBr (54.8 mg, 44.9 μL, 0.4 mmol, 4.0 equiv.), Cy_2NMe (58.5 mg, 64.0 μL, 0.3 mmol), HEH (75.9 mg, 0.3 mmol) and 4CzIPN (8.0 mg, 0.01 mmol) in DMA (3.0 mL). The crude product was purified by flash chromatography on silica gel (eluted with petroleum ether:EtOAc = 20:1) to give the product (14.6 mg, 45% yield, 50% ee) as a colorless oil. R_f = 0.41 (petroleum ether:EtOAc = 10:1). $[\alpha]_D^{25}$ = –40.30 (*c* = 1.277, $CHCl_3$). ^{1}H NMR (400 MHz, $CDCl_3$) δ: 7.96 (d, *J* = 8.5 Hz, 2H), 7.52 (d, *J* = 8.3 Hz, 2H), 7.33 (t, *J* = 7.9 Hz, 2H), 7.20 (t, *J* = 7.4 Hz, 1H), 6.95 (d, *J* = 7.6 Hz, 2H), 3.96 (dd, *J* = 9.2, 3.7 Hz, 1H), 2.61 (s, 3H), 2.45 (dd, *J* = 14.1, 9.3 Hz, 1H), 1.67 (dd, *J* = 14.1, 3.7 Hz, 1H), 1.00 (s, 9H). ^{13}C{^{1}H} NMR (101 MHz, $CDCl_3$) δ: 197.76, 172.68, 150.78, 145.80, 136.31, 129.50, 129.01, 128.26, 126.03, 121.30, 48.46, 47.22, 31.33, 29.58, 26.73. The ee was determined by HPLC with a Daicel Chiralcel IA column (^{i}PrOH/hexanes = 10/90, 1.0 mL/min, 250 nm, major t_r = 7.46 min (*S*), minor t_r = 8.641 min (*R*)). FTIR (neat, cm^{-1}) ν: 2957, 2868, 1756, 1607, 1492, 1267, 1194, 1161, 1018, 910, 813, 749, 687. HRMS: calcd for $C_{21}H_{25}O_3$ $[M+H]^+$ 325.1804, found 325.1802.

6h. Racemic product

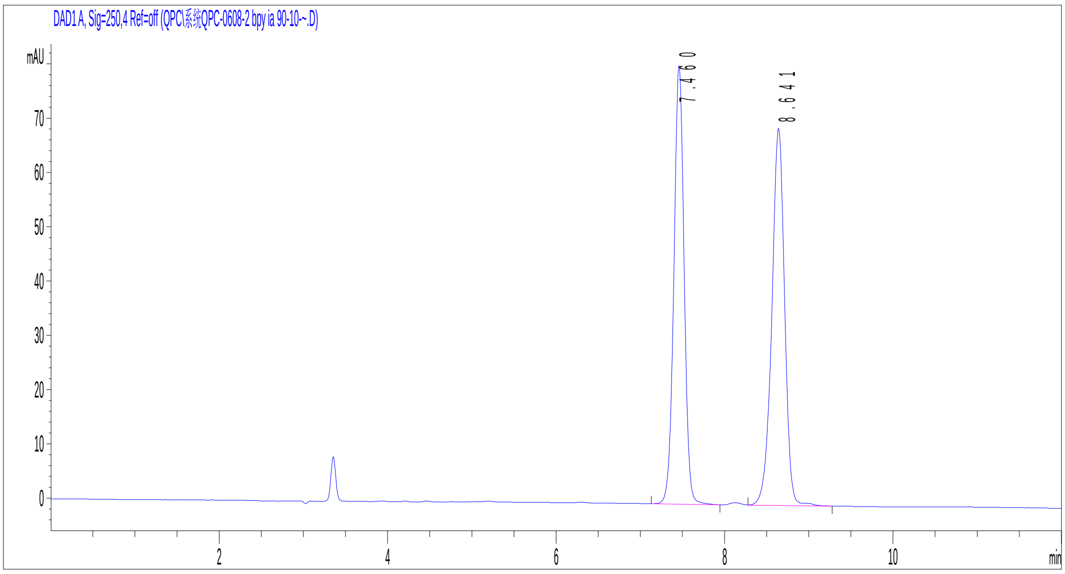

Peak	Ret Time	Type	Width	Area	Height	Area
#	[min]		[min]	mAU*s	[mAU]	%
1	7.46	BB	0.1302	679.9	80.8	48.122
2	8.641	BB	0.1611	732.9	69.5	51.878
Totals				1412.8	150.3	

6h. Enantioenriched product, 50% ee

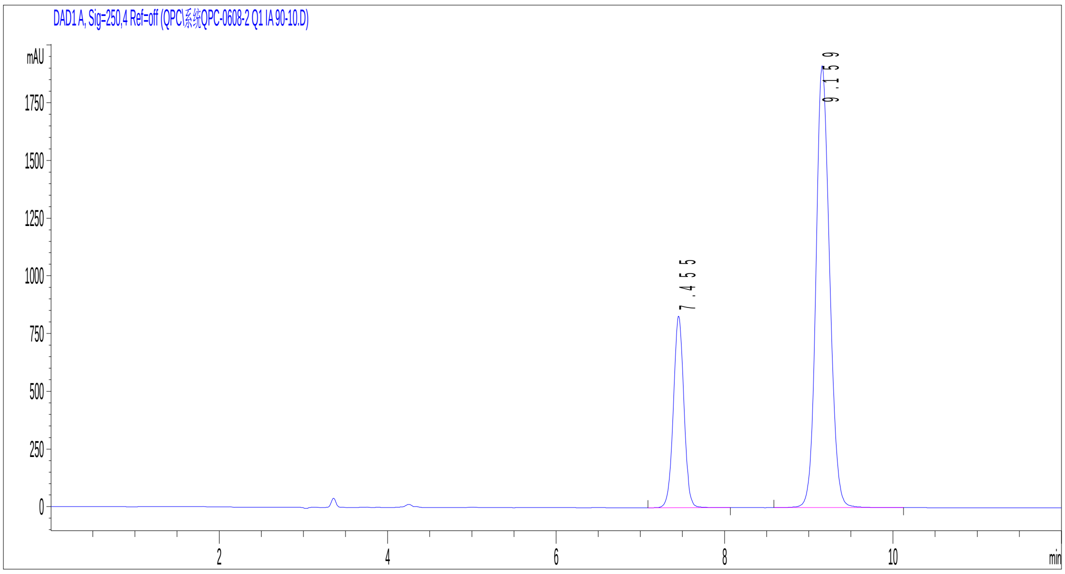

Peak	Ret Time	Type	Width	Area	Height	Area
#	[min]		[min]	mAU*s	[mAU]	%
1	7.455	BB	0.1333	7214.1	830.8	24.985
2	9.159	BB	0.174	21659.6	1914.8	75.015
Totals				28873.7	2745.6	

Supplementary References

1 Wei, X., Shu, W., Garcia-Dominguez, A., Merino, E. & Nevado, C. Asymmetric Ni-Catalyzed Radical Relayed Reductive Coupling. *J. Am. Chem. Soc.* **142**, 13515-13522 (2020).

2 Poremba, K. E., Kadunce, N. T., Suzuki, N., Cherney, A. H. & Reisman, S. E. Nickel-Catalyzed Asymmetric Reductive Cross-Coupling To Access 1,1-Diarylalkanes. *J. Am. Chem. Soc.* **139**, 5684-5687 (2017).

3 Woods, B. P., Orlandi, M., Huang, C.-Y., Sigman, M. S. & Doyle, A. G. Nickel-Catalyzed Enantioselective Reductive Cross-Coupling of Styrenyl Aziridines. *J. Am. Chem. Soc.* **139**, 5688-5691 (2017).

4 Meng, Q.-Y., Wang, S., Huff, G. S. & König, B. Ligand-Controlled Regioselective Hydrocarboxylation of Styrenes with CO_2 by Combining Visible Light and Nickel Catalysis. *J. Am. Chem. Soc.* **140**, 3198-3201 (2018).

5 Che, Y.-Y. *et al.* Palladium-Catalyzed Electrophilic Functionalization of Pyridine Derivatives through Phosphonium Salts. *Angew. Chem. Int. Ed.* **59**, 16414-16419 (2020).

6 Chen, H., Jia, X., Yu, Y., Qian, Q. & Gong, H. Nickel-Catalyzed Reductive Allylation of Tertiary Alkyl Halides with Allylic Carbonates. *Angew. Chem. Int. Ed.* **56**, 13103-13106 (2017).

7 Zhao, B. *et al.* Palladium-Catalyzed Dual Ligand-Enabled Alkylation of Silyl Enol Ether and Enamide under Irradiation: Scope, Mechanism, and Theoretical Elucidation of Hybrid Alkyl Pd(I)-Radical Species. *ACS Catal.* **10**, 1334-1343 (2020).

8 Guo, L. et al. General Method for Enantioselective Three-Component Carboarylation of Alkenes Enabled by Visible-Light Dual Photoredox/Nickel Catalysis. *J. Am. Chem. Soc.* **142** , 20390-20399 (2020).
